# Global burden of drug use disorders by region and country, 1990–2021

**DOI:** 10.3389/fpubh.2024.1470809

**Published:** 2024-10-29

**Authors:** Shuyan Zhang, Xiaoying Qi, Yingying Wang, Keyuan Fang

**Affiliations:** Department of Clinical Pharmacy, Beilun People’s Hospital, Ningbo, China

**Keywords:** Duds, GBD, burden, SDI, ASIR, DALY

## Abstract

**Background:**

This study used data from the Global Burden of Disease Study (GBD) database to systematically assess the magnitude of drug use disorders (DUD) burden between 1990 and 2021.

**Methods:**

This study used GBD data to analyze the trends in ASIR, DALYs and other DUD indicators from 1990 to 2021, and compared them among different regions and countries. The Estimated Annual Percentage Change (EAPC) and its 95% Confidence Interval (CI) were calculated to assess the temporal and geographical disparities. ASIR and DALYs were used to evaluate the burden of DUDs, and socio-demographic index (SDI) was used to measure the socio-economic development level of each country.

**Results:**

The global ASIR of DUDs showed a slight downward trend (EAPC = −0.26). The age-standardized DALY rate (per 100,000) significantly declined from 1990 to 2021 (EAPC = −1.44). Among the regions, the high SDI region exhibited the most substantial increase in ASIR (EAPC = 0.65). On a regional level, the high-income North America region had the highest EAPC for both age-standardized DALYs and ASIR (EAPC = 4.82, 1.02, respectively). Nationally, the United States of America reported the largest increase in age-standardized DALY rates and EAPC for ASIR (EAPC of 4.88, 1.05, respectively), while South Africa had the most significant decrease in EAPC (EAPC of −3.62, −1.52, respectively). In 2021, the highest ASIR was observed in high-income North America at 520.07; Central Asia had the highest age-standardized DALY rate. Globally, age-standardized DALYs and ASIR for DUDs were generally higher in men than in women, and the burden of DUDs decreased with age.

**Conclusion:**

The global burden of DUDs has shown complex and changing trends over the last decades, with large differences in burden between regions and countries. This highlights the need for targeted public health policies and interventions in High income North America region and Eastern Europe.

## 1 Introduction

Drug use disorders (DUDs) are characterized by the persistent and compulsive consumption of specific drugs for non-medical purposes, primarily to achieve particular psychological effects. These disorders can result in significant psychological, physiological consequences, and social problems (1–3). Such issues may include cognitive impairment, suicidal ideation, a reduction in quality of life, and an increased risk of infectious diseases (4–7). According to the Global Burden of Disease, Injuries, and Risk Factors Study (GBD) 2021, DUDs are among the top 20 causes of Disability-Adjusted Life Years (DALYs) in individuals aged 10–49 (8). The World Drug Report 2023 indicates that over 296 million people worldwide used drugs in 2021, with the number of individuals suffering from DUDs reaching 39.5 million—a 45% increase over the past decade (9). Despite this, only one in five individuals with drug-related disorders receives treatment, and regional disparities in access to such treatment continue to widen.

According to the United Nations Office on Drugs and Crime report, 284 million people aged 15–64 years worldwide will have used drugs in 2020.DUD seriously affects the physical and mental health of drug abusers, resulting in public health and safety problems such as AIDS (10). In particular, DUDs are recognized as chronic and relapsing brain diseases that can disrupt brain function by restoring reward pathways and changing synaptic plasticity (11). Although psychological withdrawal and medication may alleviate symptoms, DUDs remain a major global public health problem (12).

In the current global health scenario, having updated information on the burden of DUDs is important for public health policy and healthcare delivery. This will help countries to develop more effective policies that are targeted toward specific populations. Therefore, this study aims to comprehensively assess the magnitude and temporal trends of the global burden of DUDs from 1990 to 2021 using the most recent estimates of DUDs burden from GBD 2021. It also assesses the inequalities in the global burden of DUDs by age, sex, and age-standardized incidence rate (ASIR) and DALY (8).

## 2 Methods

### 2.1 Data collection and case definition

The GBD provides comprehensive estimates of risk exposure and health loss attributable to risk factors worldwide, utilizing all relevant available data. The methods for data collection, processing, and analysis in GBD 2021 are detailed elsewhere (13). GBD 2021 estimated relevant metrics across 23 age groups, from birth to 95 years and older. It includes data for males, females, and all sexes combined, covering 204 countries and territories, which are organized into 21 regions and seven super-regions. GBD regions consist of countries and territories that are geographically close and epidemiologically similar. These regions are further grouped into super-regions based on patterns of causes of death. Burden data for DUDs were obtained from the Institute for Health Metrics and Evaluation (IHME).^1^ This paper reports burden data on DUDs including age-standardized incidence and age-standardized DALY rate (per 100,000), Age standardized rates (ASRs) (per 100,000), and SDIs by age, sex, country and region from 1990 to 2021 (14). In GBD 2021, DUDs are defined based on the diagnostic criteria of the Diagnostic and Statistical Manual of Mental Disorders (DSM-IV-TR) or International Classification of Diseases (ICD-10), including Opioid Use Disorder, Cocaine Use Disorder, Cannabis Use Disorder, Amphetamine Use Disorder and Other DUDs (15). The latter category includes Hallucinogenic Dependence, Inhalant or Solvent Dependence, Sedative Dependence and Other DUDs. This study was based on a publicly available databases and does not require ethical approval.

### 2.2 Socio-economic development indicators

The SDI is a composite index that includes the lagged distribution of *per capita* income, average years of schooling, and female fertility rate under 25 years old. It is used as a comprehensive measure of socioeconomic development (8). The GBD study uses the human development index methodology to calculate the composite SDI, which is defined as the geometric mean of the three covariates mentioned above (16). The values of SDI range from 0 to 1, indicating different levels of socio-economic development, with higher values indicating better development. The sample was divided into 21 regions and 204 countries based on the SDI according to their social development status: Low SDI, Low-middle SDI, Middle SDI, High-middle SDI, and High SDI.

### 2.3 Statistical analysis

The statistically analyzed data were presented by age, sex, year, region, and country, accompanied by a 95% uncertainty interval (UI). The analysis included point estimates along with their corresponding 95% confidence intervals (CIs), which represent the 25th and 95th percentiles of the distribution, were extracted to account for estimation uncertainty. In GBD 2021 study, covariates were added and time smoothing was performed to improve the stability of results. ASR (17) were estimated based on global standardized population of GBD study (2021). To assess trends in ASR over specific time intervals, linear regression model and natural logarithmic transformation were fitted to the data. The goodness of fit of the model was evaluated by bias. The model assumes that the natural logarithm of ASIR (Y) is linear with respect to calendar year (X), with random bias (*ε*). *Y* = *α* + *β*X + ε where *β* represents the direction and magnitude of the ASIR trend. To evaluate the time trend, we introduced the Estimated Annual Percentage Change (EAPC) metric and calculated EAPC as EAPC = 100 × (exp (*β*) − 1) (18–20). A downward trend was defined when the upper limit of 95% CI of EAPC was less than 0; an upward trend of burden was defined when the lower limit of 95% CI of EAPC was greater than 0; otherwise, the trend was stable. Furthermore, associations between EAPC, DUDs-related burden and SDI were assessed using Pearson or Spearman rank tests. All statistical analyses and visualizations were conducted using R software (version 4.0.3),^2^ and a two-tailed *p* < 0.05 was considered statistically significant.

## 3 Results

### 3.1 Temporal trends in the burden of substance use disorders, 1990–2021

**Figure 1:**
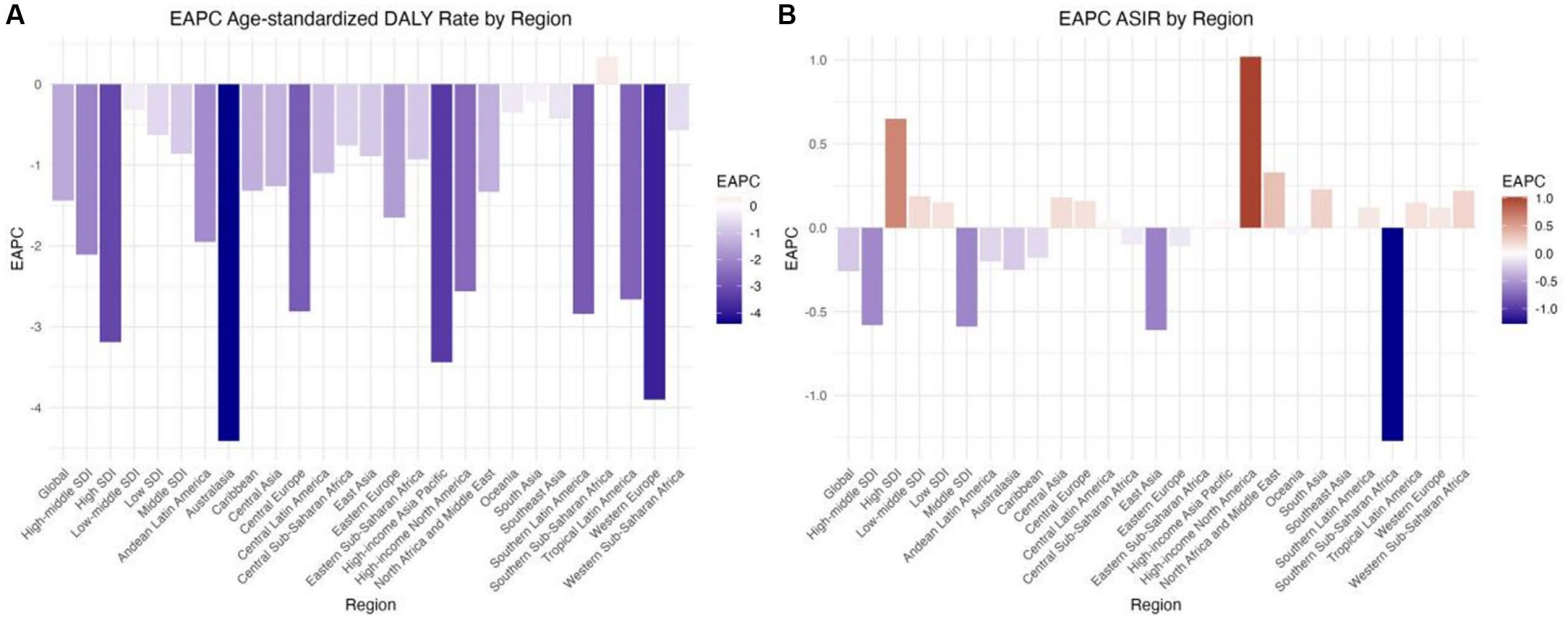
EAPC of ASRs of drug use disorders from 1990 to 2021, by locations. (A) Age-standardized DALY rate (B) ASIR. DALY, disability adjusted life-year; ASIR, age standardized incidence rate; ASRs, age standardized rates.

**Figure 2:**
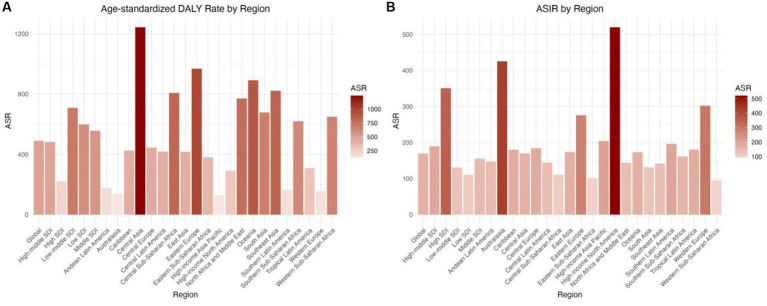
ASRs drug use disorders in 2021, by locations. (A) Age-standardized DALY rate (B) ASIR. DALY, disability adjusted life-year; ASIR, age standardized incidence rate; ASRs, age standardized rates.

**Figure 3:**
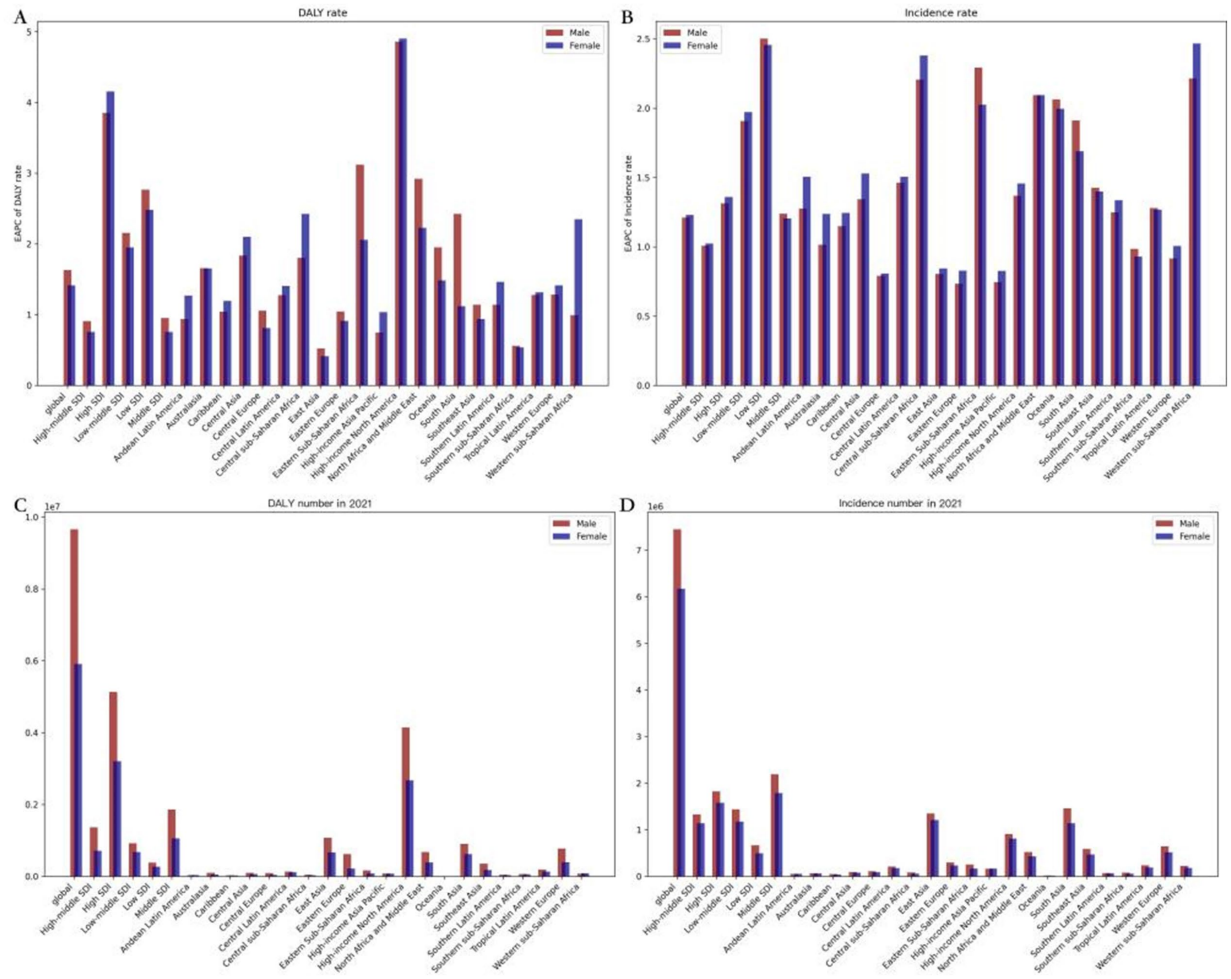
ASRs of drug use disorders, by locations and sexes. (A) EAPC of ASIR; (B) EAPC of age-standardized DALY rate; (C) Age-standardized DALY rate in 2021; (D) ASIR in 2021. DALY, disability adjusted life-year; ASIR, age standardized incidence rate; ASRs, age standardized rates.

Globally, the incidence of DUDs was 13,609,362.3 in 2021, which was higher than that in 2019 (11,163,596.9). The number of DALY cases was 15,562,161.5 in 2021, which was higher than that in 1990 (10,100,720.7) (Supplementary Table S1; Figures 1A,B). In addition, the ASIR for all ages in DUDs decreased from 206.18 (95% UI: 174.39; 238.74) in 1990 to 169.39 (95% UI: 145.14; 195.01) in 2021 with an EAPC of −0.26 (95% UI: −5.46; 5.22) (Table 1; Figures 1B, 2B, 3AD). Meanwhile, the age-standardized DALY rate (per 100,000) decreased from 742.17 (95% UI: 597.93; 879.88) in 1990 to 489.81 (95% UI: 391.39; 593.21), with an EAPC of −1.44 (−7.63; 5.18) (Table 1; Figures 1A, 2A, 3BC).

**Table 1:** Age-standardized DALY rate and ASIR and corresponding EAPC for global DUDs in 1990 and 2021.

Location	ASIR			Age-standardized DALY rate (per 100,000)		
	1990 No. (95%UI)	2021 No. (95%UI)	1990–2021 EAPC No. (95%CI)	1990 No. (95%UI)	2021 No. (95%UI)	1990–2021 EAPC No. (95%CI)
Global						
Male	222.54 (190.47, 256.77)	183.99 (159.71, 211.51)	−0.26 (−5.54, 5.31)	851.58 (686.58, 1023.05)	598.14 (479.64, 722.15)	−1.21 (−7.61, 5.64)
Female	189.27 (158.66, 221.96)	154.12 (130.49, 179.18)	−0.26 (−5.37, 5.11)	642.58 (519.51, 765.26)	392.51 (312.52, 473.79)	−1.70 (−7.67, 4.65)
Both	206.18 (174.39, 238.74)	169.39 (145.14, 195.01)	−0.26 (−5.46, 5.22)	742.17 (597.93, 879.88)	489.81 (391.39, 593.21)	−1.44 (−7.63, 5.18)
SDI						
High-middle SDI	213.21 (181.57, 246.44)	189.65 (161.53, 218.09)	−0.58 (−5.90, 5.04)	856.77 (690.30, 1031.82)	482.12 (379.01, 594.29)	−2.11 (−8.26, 4.45)
High SDI	284.65 (242.42, 330.56)	350.90 (307.36, 400.20)	0.65 (−5.34, 7.02)	548.06 (430.57, 664.63)	220.40 (173.92, 266.10)	−3.19 (−8.51, 2.44)
Low-middle SDI	124.75 (105.84, 145.45)	130.55 (110.96, 151.41)	0.19 (−4.80, 5.44)	794.83 (643.62, 954.95)	709.62 (559.48, 858.77)	−0.32 (−6.95, 6.79)
Low SDI	107.37 (88.96, 125.35)	110.82 (92.59, 128.79)	0.15 (−4.67, 5.21)	719.83 (575.38, 878.66)	597.97 (468.77, 731.27)	−0.63 (−7.08, 6.26)
Middle SDI	178.51 (152.88, 204.74)	155.19 (131.25, 179.27)	−0.59 (−5.71, 4.81)	750.17 (611.72, 916.52)	555.50 (440.55, 679.88)	−0.86 (−7.22, 5.93)
Regions						
Andean Latin America	195.61 (166.30, 225.46)	147.25 (123.64, 171.11)	−0.20 (−5.27, 5.13)	303.16 (238.57, 372.33)	177.54 (130.63, 230.85)	−1.95 (−7.15, 3.54)
Australasia	529.52 (453.67, 616.27)	425.48 (369.38, 483.04)	−0.25 (−6.36, 6.26)	508.43 (383.46, 631.96)	139.51 (107.13, 171.93)	−4.41 (−9.22, 0.66)
Caribbean	199.55 (166.32, 238.29)	180.09 (147.21, 220.40)	−0.18 (−5.44, 5.36)	639.20 (505.32, 776.75)	424.57 (332.08, 524.68)	−1.32 (−7.39, 5.15)
Central Asia	173.93 (144.74, 204.34)	169.72 (143.57, 197.09)	0.18 (−5.04, 5.68)	1574.74 (1267.28, 1868.55)	1243.96 (979.87, 1537.34)	−1.26 (−8.38, 6.42)
Central Europe	197.80 (164.95, 233.27)	184.24 (155.27, 214.63)	0.16 (−5.14, 5.75)	934.23 (736.11, 1129.34)	444.98 (348.90, 543.68)	−2.81 (−8.84, 3.61)
Central Latin America	162.99 (137.28, 191.04)	144.04 (121.38, 167.38)	0.04 (−5.01, 5.35)	552.50 (437.50, 663.36)	417.92 (320.09, 518.20)	−1.10 (−7.16, 5.35)
Central Sub-Saharan Africa	128.28(106.92,150.81)	110.05(91.77,129.82)	−0.10(−4.88,4.91)	957.08(730.98,1235.06)	807.46(606.17,1044.96)	−0.76(−7.48,6.45)
East Asia	229.24 (194.35, 266.18)	173.93 (146.09, 204.63)	−0.61 (−5.82, 4.88)	631.09 (487.49, 794.25)	416.58 (317.97, 550.67)	−0.89 (−6.97, 5.59)
Eastern Europe	302.11(257.48,349.99)	275.72(238.80,312.90)	−0.11(−5.79,5.92)	1341.94(1052.31,1614.53)	968.69(755.39,1183.22)	−1.65(−8.51,5.72)
Eastern Sub-Saharan Africa	114.08 (95.47, 135.50)	101.09 (83.77, 119.60)	0.02 (−4.68, 4.95)	481.45 (374.63, 608.71)	379.81 (288.86, 492.14)	−0.93 (−6.91, 5.44)
High-income Asia Pacific	222.75 (180.91, 269.46)	204.38 (168.19, 247.27)	0.04 (−5.36, 5.75)	366.11 (281.03, 456.93)	128.42 (100.73, 159.17)	−3.44 (−8.23, 1.60)
High-income North America	436.37 (373.43, 512.11)	520.07 (454.13, 592.82)	1.02 (−5.34, 7.81)	578.69 (438.12, 709.61)	291.42 (228.20, 354.80)	−2.56 (−8.18, 3.41)
North Africa and Middle East	145.74 (119.98, 173.60)	143.52 (120.87, 169.07)	0.33 (−4.74, 5.66)	1149.62 (884.03, 1419.31)	770.51 (569.14, 975.54)	−1.33 (−7.98, 5.79)
Oceania	185.47 (152.23, 230.70)	173.25 (141.60, 212.00)	−0.04 (−5.27, 5.48)	998.63 (782.43, 1285.58)	891.76 (686.65, 1156.78)	−0.35 (−7.20, 7.02)
South Asia	139.85 (116.75, 164.68)	131.41 (109.78, 153.28)	0.23 (−4.73, 5.46)	736.91 (585.48, 911.41)	678.71 (520.99, 835.91)	−0.22 (−6.82, 6.85)
Southeast Asia	155.47 (130.90, 183.14)	141.48 (116.93, 166.01)	−0.01 (−5.04, 5.28)	943.06 (774.81, 1144.82)	822.39 (651.11, 1002.89)	−0.43 (−7.19, 6.82)
Southern Latin America	211.62(175.58,249.36)	196.13(167.54,227.34)	0.12(−5.24,5.78)	432.65(333.62,532.54)	165.45(126.13,203.86)	−2.84(−7.93,2.53)
Southern Sub-Saharan Africa	260.10 (221.76, 303.29)	161.51 (137.31, 186.47)	−1.27 (−6.35, 4.08)	564.09 (453.13, 678.12)	620.42 (504.67, 742.66)	0.34 (−6.22, 7.36)
Tropical Latin America	203.95(173.73,242.27)	180.40(153.43,207.80)	0.15(−5.15,5.73)	700.52(576.95,824.96)	307.55(250.01,364.38)	−2.66(−8.34,3.38)
Western Europe	321.12 (272.30, 373.02)	302.00 (262.87, 348.16)	0.12 (−5.67, 6.26)	491.11 (385.72, 598.00)	156.51 (123.02, 191.55)	−3.90 (−8.87, 1.33)
Western Sub-Saharan Africa	106.83 (89.61, 126.14)	94.68 (79.60, 111.36)	0.22 (−4.42, 5.09)	769.65 (623.31, 940.52)	649.65 (523.69, 798.64)	−0.57 (−7.09, 6.42)

The ASIR varied substantially among SDI subgroups from 1990 to 2021. The High SDI region had the largest increase in ASIR (EAPC = 0.65), and the Middle SDI region had the largest decrease (EAPC = −0.59) (Table 1; Figure 1B). The age-standardized DALYs also differed significantly among SDI subgroups, with all SDI regions showing a decreasing trend. The greatest reduction was observed in the High SDI region (EAPC = −3.19), and the least reduction was observed in the Low-middle SDI region (EAPC = −0.32) (Table 1; Figure 1A). In different country regions, both prevalence and age-standardized DALY rate decreased initially as the SDI increased up to 0.75, then increased as the SDI continued to rise. When only the SDI was considered, Qatar had a significantly higher ASIR and age-standardized DALY rate than expected, and the Republic of Korea had a significantly lower rate than expected (Supplementary Figure S5).

Of the 21 geographic regions, both male and female EAPCs were highest in the High-income North America region (EAPCs of 4.82, 4.80, and 4.85, respectively) and lowest in Southern sub Saharan Africa (EAPCs of-3.22, −3.32, and −3.11, respectively) (Figures 3B,C; Supplementary Figure S3). The age-standardized incidence rate was highest for both sexes in High-income North America (EAPC 1.02, 1.23, and 0.85 respectively) and lowest for both sexes in Southern sub-Saharan Africa (EAPC-3.22, −3.32, and −3.11 respectively) (Supplementary Figure S4; Supplementary Table S3). The age-standardized incidence rate was highest for males and lowest for females (EAPC of −1.27, −1.23, and −1.34, respectively) (Supplementary Figure S4; Supplementary Table S3). Of the 204 countries, the United States of America had the largest increase in DALY, with EAPCs of 4.88, 4.86, and 4.93 for male and female respectively, while South Africa had the largest decrease, with EAPCs of −3.62, −3.81, and −3.43 for female, respectively, (Supplementary Figure S3; Supplementary Table S1) In terms of incidence, the United States of America had the highest increase in both male and female populations(Supplementary Figure S4; Supplementary Table S3), with EAPCs of 1.05, 1.28, and 1.12 respectively, while South Africa had the largest decrease in both male and female populations, with EAPCs of −1.52, −1.64, and −1.42, respectively, (Supplementary Figure S4; Supplementary Table S3). Low-middle SDI regions have the highest EAPCs for ASIR and these are higher for males than for females, while high-income North America regions have the highest EAPCs and these are higher for females than for males for age-standardized DALY rates. The EAPC is highest in the High-income North America region and this is higher for females than for males (Figures 3A,B). Overall, from 1990 to 2021, the global EAPC for the age-standardized DALY rate was lower for women than for men (EAPCs of 1.414 and 1.629 respectively), and the EAPA for the ASIR was higher for women than for men (EAPCs of 1.229 and 1.21 respectively) (Figures 3A,B).

### 3.2 Distribution of the burden of DUDs at the national and regional levels, 2021

The highest ASIR was reported in high-income North America (520.07 [454.13; 592.82]) and the lowest in Western Sub-Saharan Africa (94.68 [79.60; 111.36]); the highest age-standardized DALYs rate was found in Central Asia (1,243.96 [979.87; 1,537.34]) and the lowest in high-income Asia Pacific (128.42 [100.73; 159.17]). According to the World Health Organization (WHO), among 204 countries worldwide, the United States of America had the highest age-standardized DALY rate (per 100,000) and ASIR for DUDs in 2021 without considering gender(1,944.08 and 531.19, respectively), while Nigeria and Kenya had the lowest rates (33.93 and 86.25, respectively) (Supplementary Figures S3A, S4A; Supplementary Tables S1, S3).

The global age-standardized incidence and DALY of DUDs decreased with age in 2021. Zimbabwe had the highest proportion of DUDs incidence and DALY among individuals aged 15–19 and 25–29 years (1,736.673 and 4,838.232, respectively). The highest proportion of DUDs incidence and DALY was observed in Zimbabwe’s 15–29 year olds within the corresponding age groups (Supplementary Tables S1, S2; Supplementary Figure S2). However, the highest proportion was observed in the 30–49 year olds within the corresponding age groups, namely Australia for those aged 30–40 years and Paraguay for those aged 45–49 years (Supplementary Tables S1, S4; Supplementary Figure S1). Regarding sex, the age-standardized DALY rate and ASIR of DUDs were higher in males than females across all 21 regions. Globally, the age-standardized DALY rate was significantly higher in males at 1,077,455. 21(851,106.38; 1,324,784. 32) than in females at 1,018,345. 20 (788,246.88; 1,258,056. 70). Similarly, the global ASIR was also higher in males at 598.14 (479.64; 722.15) than in females at 392.51 (312.52; 473.79) (Table 1; Figures 3C,D).

## 4 Discussion

This study found that the global ASIR and age-standardized DALY rate for DUDs both declined. There was a gender difference in the global burden of DUDs. There are significant regional variations in the burden of DUDs. The burden of DUDs decreases with increasing age. The trends in ASIR varied significantly across different SDI regions.

Globally, the disease burden associated with DUDs is quantified using the age-standardized DALY rate and ASIR, which have remained relatively consistent over the past three decades. It is widely recognized that examining temporal trends and attributable risk factors can provide valuable insights for the development of prevention and control policies. Furthermore, DALYs serve as a measure of disease burden that health policymakers address promptly to benefit the nation, society, families, and individuals as a whole.

This study conducted a systematic analysis of the global burden of DUDs and its trends from 1990 to 2021, using data sourced from the GBD database. Both the global ASIR and age-standardized DALY rate for DUDs demonstrated a decline over this period, suggesting a potential positive trend in mitigating the health impacts of DUDs worldwide. The decreasing rate of age-standardized DALYs implies a reduction in the severity of DUDs when assessed. This could be attributed to advancements in medical interventions that have curtailed premature death and disability among individuals with DUDs (21). The prevalence of DUDs is influenced by factors such as region, country, age, and gender (2), and heightened awareness of DUDs, coupled with more effective social support systems, may also contribute to reduced rates of DALYs (22). Furthermore, the High income North America region warrants special attention due to its highest age-standardized DALY rate (per 100,000) and ASIR for DUDs in 2021, potentially attributable to higher rates of marijuana, opioid, and cocaine dependence in the region (23).

Gender differences in the global burden of DUDs have also been reported, with males generally having a higher burden than females (24). Biological sex differences (including brain structure and function, endocrine function, and metabolic function) and gender roles are all contributors to this disparity (25). In addition, there are substantial regional variations in the burden of DUDs. For example, High income North America has the highest EAPC for age-standardized DALY rate DALYs and ASIR, which may be due to the high prevalence of substance abuse and relatively high drug availability in this region (26). On the other hand, the low EAPC values in sub-Saharan Africa may reflect the limited healthcare resources and lower diagnosis rates in this region (27).

The present study also found that the burden of DUDs decreased with age. Marijuana is considered a relatively safe recreational drug, but repeated use during adolescence can affect resting brain connectivity, intelligence and cognitive functioning (28). The prevalence of DUDs and DALYs are higher among adolescents in many countries, so preventive measures and interventions should be implemented for younger populations to reduce the health impact of DUDs (29). In some countries such as Zimbabwe, the burden of DUDs is high among adolescents and young adults, which may be related to socioeconomic stress, poor education opportunities and employment prospects (30). The slight decrease in ASIR may be due to global prevention and treatment strategies for DUDs (31). However, the trends of ASIR were different across SDI regions. The significant decrease in ASIR in Middle SDI regions may be related to sustained public health policies, medical resource allocation and disease prevention and control. All SDI regions showed a decreasing trend in DALY rate, especially in High SDI region, indicating that economic development and social progress play an important role in reducing disease burden (13).

This study has several strengths. First, the data used in this study were derived from the GBD (1990–2021), which is a high-quality and reliable source of information. Second, this study comprehensively analyzed the global burden of DUDs over the past three decades in different regions, periods, and factors. Third, the results of this study have important implications for public health policy formulation. For instance, countries and regions facing a high burden of DUDs should first strengthen regulations on addictive substances, followed by increasing health interventions aimed at addressing drug addiction among young people. However, there are some limitations to this study. First, the burden of DUDs may be underestimated due to limited data availability, especially in low-income countries. Second, although age, sex, country, and SDI were considered in this study, genetics, living environment, and individual psychological characteristics were not taken into account when assessing the burden of DUDs. Finally, the processing methods and modeling techniques used in this study may affect the accuracy of data assessment to some extent.

## 5 Conclusion

In conclusion, the global burden of DUDs based on GBD data showed a slight decrease in ASIR, a significant reduction in DALYs, and marked differences in gender, age, region, and SDI. These results highlight the need for targeted prevention and intervention strategies, and that socioeconomic development is an important factor in reducing disease burden. This study highlights the serious threat that DUDs pose to global health and emphasizes the importance of developing effective strategies to address this issue. Preventive and therapeutic measures should be tailored to the specific needs of different regions and populations. International cooperation and knowledge sharing are also essential for tackling the global DUD epidemic.

## Data Availability

The original contributions presented in the study are included in the article/Supplementary material, further inquiries can be directed to the corresponding author.

## Footnotes

^1^ https://vizhub.healthdata.org/gbd-results/

^2^ https://www.R-project.org/
