# Supplementary material for: Global burden of drug use disorders by region and country, 1990–2021
Source: Front Public Health. 2024 Oct 29;12:1470809. doi: 10.3389/fpubh.2024.1470809 (PMC11554507; doi:10.3389/fpubh.2024.1470809)

**Figure S1: Incidence of drug use disorders in 204 countries in 2021,by countries and ages.**

**
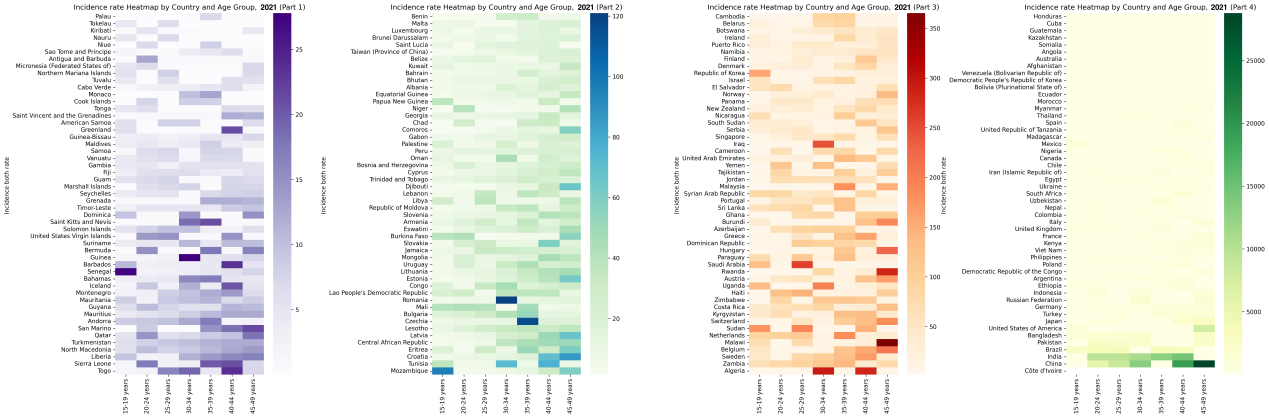
**

**Figure S2: DALY of drug use disorders in 204 countries in 2021, by countries and ages.**

**
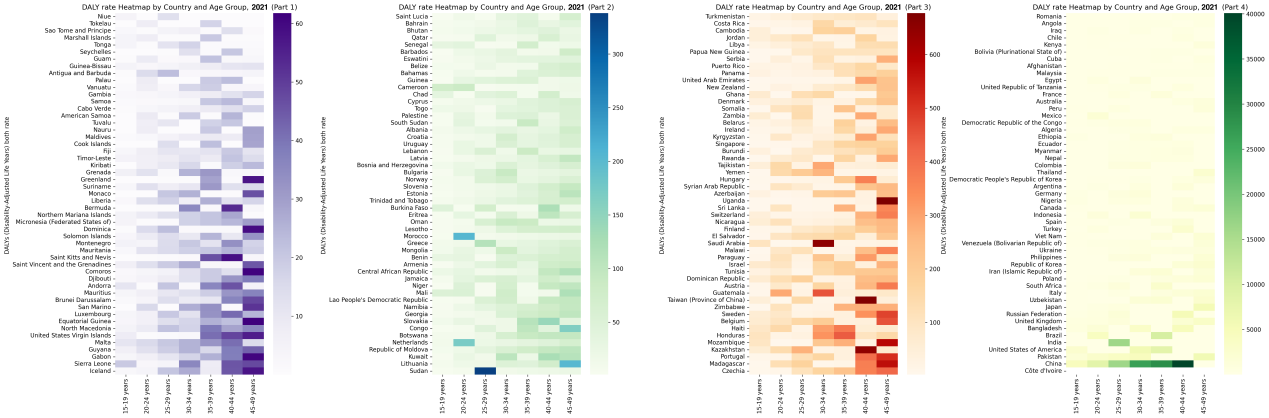
**

**Figure S3: Age-standarized DALY rate of drug use disorders in 204 countries from 1990 to 2021, by locations.** (A) Both (B) Female (C) Male.


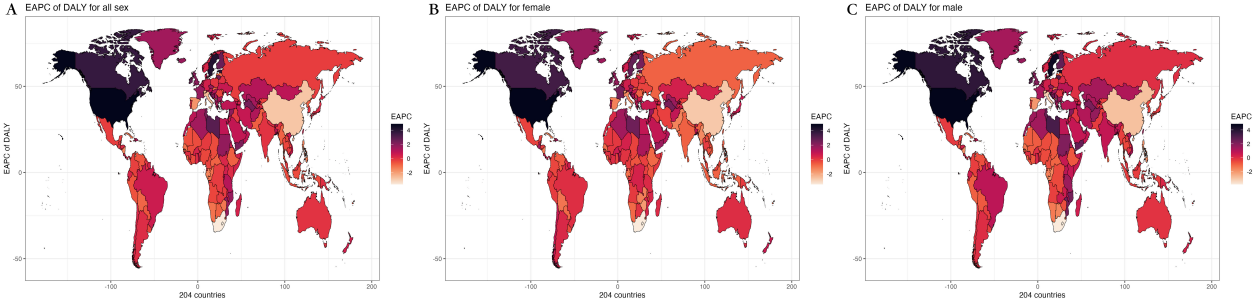


**Figure S4: Age-standarized incidence rate of drug use disorders in 204 countries from 1990 to 2021, by locations.** (A) Both (B) Female (C) Male.


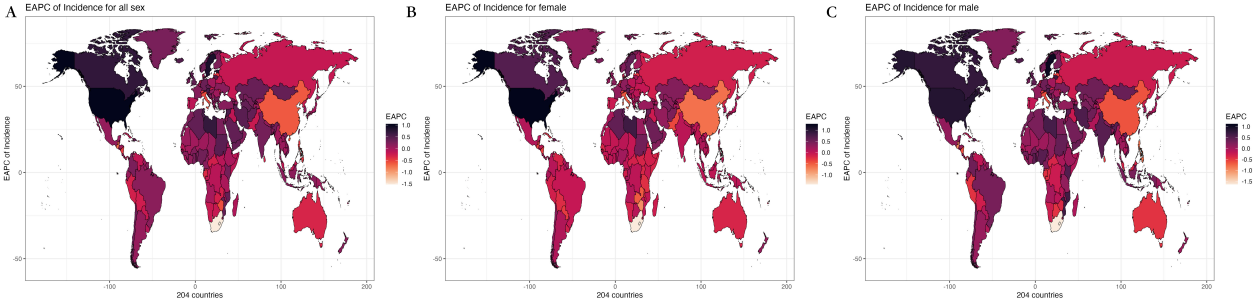


**Figure S5: Scatter plot of correlation analysis between ASRs of drug use disorders from 1990 to 2021 and the level of SDI, by locations. (A) ASIR (B) Age-standardized DALY rate. DALY = disability adjusted life-year. ASIR = age standardized incidence rate. ASRs = age standardized rates.**


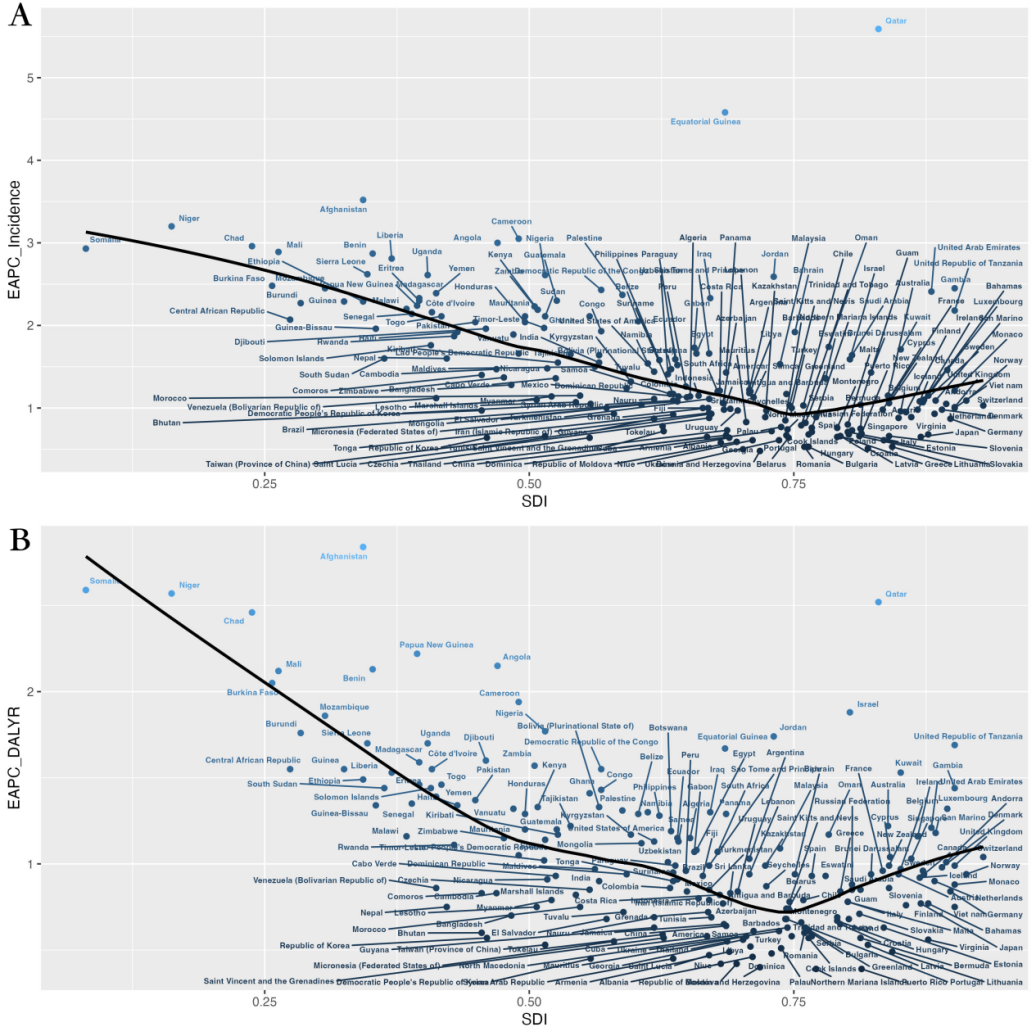

Supplement: Supplementary file 1 [file Data_Sheet_1.docx]
