# Supplementary material for: Global burden of drug use disorders by region and country, 1990–2021
Source: Front Public Health. 2024 Oct 29;12:1470809. doi: 10.3389/fpubh.2024.1470809 (PMC11554507; doi:10.3389/fpubh.2024.1470809)
Supplement: Supplementary file 2 [file Table_1.docx]

**Supplementary Table 1 Age-standarized DALY rate of drug use disorders in 204 countries from 1990 to 2021, by locations and sex.**

| nation | sex | DALY No.(95%UI) | | | Age-standardized DALY rate (per 100,000) No.95%UI | | |
| --- | --- | --- | --- | --- | --- | --- | --- |
|  |  | 1990 | 2021 | 1990-2021 EAPC No.(95%CI) | 1990 | 2021 | 1990-2021 EAPC No.(95%CI) |
| United States of America | male | 799,072.49(618,169.90; 1,000,165.22) | 3,929,671.93(3,388,391.44; 4,454,368.44) | 5.53(-9.90; 23.61) | 586.10(452.07; 734.52) | 2,374.75(2,043.17; 2,705.27) | 4.86(-3.29; 13.69) |
| Sweden | male | 7,663.17(6,396.08; 9,158.76) | 31,379.68(26,127.21; 36,911.49) | 4.98(-5.77; 16.96) | 176.43(146.20; 211.82) | 638.21(528.86; 752.94) | 4.55(-2.27; 11.85) |
| Estonia | male | 3,417.52(2,619.34; 4,336.99) | 6,877.60(5,543.50; 8,251.01) | 3.75(-5.37; 13.75) | 442.82(339.42; 562.63) | 1,121.93(899.60; 1,356.54) | 4.29(-3.06; 12.20) |
| South Africa | male | 100,084.24(70,071.27; 137,268.72) | 52,206.46(43,468.88; 61,804.97) | -2.20(-12.67; 9.52) | 543.02(385.48; 736.69) | 178.85(149.03; 211.25) | -3.81(-8.88; 1.54) |
| Canada | male | 53,311.36(41,516.01; 66,779.97) | 210,163.76(184,622.79; 236,014.57) | 4.43(-8.10; 18.66) | 360.22(280.25; 454.17) | 1,150.84(1,009.67; 1,298.42) | 3.81(-3.56; 11.74) |
| Libya | male | 3,425.58(2,619.74; 4,337.75) | 14,569.54(11,517.07; 18,520.62) | 6.33(-3.78; 17.51) | 181.36(140.27; 227.89) | 345.77(274.66; 439.96) | 3.21(-2.90; 9.70) |
| Italy | male | 112,679.11(91,173.64; 136,855.18) | 47,611.44(36,751.46; 57,986.71) | -3.43(-13.69; 8.04) | 382.91(310.49; 466.50) | 180.04(137.08; 222.26) | -3.11(-8.21; 2.28) |
| Slovenia | male | 1,134.66(828.57; 1,499.49) | 2,465.46(2,004.82; 2,943.90) | 2.82(-5.24; 11.56) | 111.32(81.64; 147.33) | 266.34(215.05; 320.98) | 3.11(-2.73; 9.31) |
| Mauritius | male | 714.39(484.20; 1,001.80) | 1,957.79(1,656.77; 2,258.49) | 3.38(-4.49; 11.89) | 112.93(77.24; 156.58) | 279.74(236.35; 324.85) | 3.09(-2.80; 9.34) |
| Lithuania | male | 5,395.85(4,357.45; 6,521.62) | 8,180.84(6,772.36; 9,677.21) | 1.56(-7.55; 11.56) | 288.10(232.92; 348.26) | 639.62(528.13; 756.75) | 2.94(-3.77; 10.11) |
| China | male | 2,013,500.57(1,589,010.94; 2,485,619.53) | 1,021,678.46(795,974.73; 1,249,554.17) | -2.42(-15.53; 12.73) | 301.56(240.20; 368.73) | 139.44(108.82; 171.93) | -2.85(-7.73; 2.30) |
| Switzerland | male | 26,092.80(20,531.06; 31,436.95) | 15,304.54(12,891.79; 17,940.16) | -2.21(-11.54; 8.11) | 687.63(542.76; 830.15) | 346.68(289.49; 408.77) | -2.82(-8.56; 3.28) |
| Ireland | male | 3,805.38(2,892.91; 4,835.87) | 13,147.89(11,051.72; 15,276.79) | 3.85(-5.95; 14.67) | 212.88(160.99; 270.21) | 571.70(478.89; 663.95) | 2.79(-3.81; 9.85) |
| Finland | male | 10,093.18(8,587.33; 11,875.89) | 18,833.94(16,167.75; 21,655.15) | 2.46(-7.55; 13.56) | 370.67(312.42; 440.02) | 732.53(628.40; 847.02) | 2.64(-4.21; 9.97) |
| Yemen | male | 3,588.62(2,607.98; 4,833.27) | 23,978.27(17,285.54; 32,539.41) | 6.48(-4.13; 18.26) | 74.33(54.93; 99.10) | 162.54(119.17; 221.78) | 2.58(-2.72; 8.17) |
| Bosnia and Herzegovina | male | 1,316.09(926.31; 1,814.67) | 1,210.82(879.98; 1,558.35) | 1.28(-5.98; 9.10) | 50.99(36.02; 69.77) | 77.38(56.40; 101.40) | 2.53(-2.03; 7.30) |
| Puerto Rico | male | 5,624.61(4,187.68; 7,367.81) | 6,426.16(5,360.64; 7,590.45) | -2.81(-11.48; 6.70) | 319.78(238.53; 418.52) | 409.01(341.29; 484.09) | -2.51(-8.66; 4.06) |
| Tunisia | male | 3,972.25(2,962.06; 5,106.59) | 13,486.50(10,300.58; 16,929.90) | 4.04(-5.78; 14.88) | 101.29(76.14; 128.44) | 218.70(166.62; 274.14) | 2.51(-3.09; 8.43) |
| Mozambique | male | 3,058.50(2,224.68; 4,089.51) | 10,402.47(7,634.54; 13,664.16) | 5.09(-4.56; 15.70) | 66.99(49.59; 86.90) | 94.37(70.43; 124.11) | 2.29(-2.44; 7.26) |
| Brunei Darussalam | male | 326.21(249.30; 415.34) | 377.82(288.15; 481.18) | 0.16(-5.87; 6.57) | 236.99(187.96; 293.58) | 131.28(99.63; 166.96) | -2.20(-7.07; 2.93) |
| Turkmenistan | male | 2,138.65(1,521.59; 2,914.58) | 7,402.63(5,667.58; 9,501.80) | 3.88(-5.33; 13.98) | 120.97(86.90; 161.09) | 260.32(199.11; 335.04) | 2.19(-3.57; 8.29) |
| Lebanon | male | 2,472.64(1,906.25; 3,118.01) | 5,244.35(3,977.89; 6,584.39) | 4.74(-4.22; 14.54) | 173.44(133.69; 218.59) | 167.39(126.98; 210.74) | 2.14(-3.19; 7.75) |
| North Macedonia | male | 847.91(589.70; 1,172.07) | 1,557.03(1,171.71; 1,956.15) | 2.43(-5.16; 10.62) | 78.37(54.49; 108.23) | 134.03(100.47; 169.60) | 2.10(-3.02; 7.48) |
| Sudan | male | 7,319.49(5,446.68; 9,757.90) | 41,132.84(30,527.80; 52,261.92) | 5.02(-5.97; 17.29) | 90.48(67.85; 120.44) | 200.04(149.42; 254.25) | 2.07(-3.41; 7.86) |
| Spain | male | 77,838.93(62,440.56; 95,558.57) | 62,227.35(49,808.77; 75,029.91) | -1.71(-12.41; 10.29) | 387.17(312.05; 475.39) | 290.93(231.36; 355.55) | -1.98(-7.63; 4.01) |
| United Kingdom | male | 108,113.42(85,178.44; 134,466.08) | 243,507.48(211,181.27; 274,816.61) | 2.50(-9.93; 16.63) | 373.94(294.47; 465.13) | 746.69(645.92; 845.41) | 1.93(-4.87; 9.21) |
| Syrian Arab Republic | male | 4,668.02(3,518.17; 5,989.64) | 7,464.02(5,747.87; 9,166.56) | 2.28(-6.81; 12.26) | 87.51(67.63; 110.46) | 121.20(92.98; 146.90) | 1.91(-3.06; 7.14) |
| Nicaragua | male | 1,946.00(1,368.70; 2,582.98) | 2,112.85(1,563.59; 2,764.89) | 0.67(-7.05; 9.03) | 123.80(88.68; 162.40) | 60.21(45.01; 77.84) | -1.86(-5.97; 2.43) |
| Philippines | male | 51,193.22(36,938.98; 67,490.39) | 42,926.87(30,307.63; 55,630.09) | 0.30(-10.24; 12.09) | 162.78(118.03; 213.22) | 69.71(49.79; 89.73) | -1.84(-6.09; 2.61) |
| Albania | male | 1,472.60(1,042.98; 2,046.71) | 2,649.11(2,082.96; 3,272.59) | 1.05(-6.92; 9.71) | 81.72(58.45; 112.69) | 185.38(145.91; 229.92) | 1.83(-3.57; 7.53) |
| United Arab Emirates | male | 3,870.77(2,970.33; 4,943.05) | 20,562.98(15,125.64; 25,946.04) | 9.26(-1.56; 21.28) | 249.67(192.44; 315.24) | 239.76(177.59; 303.08) | 1.80(-3.91; 7.84) |
| Congo | male | 1,751.15(1,243.78; 2,326.44) | 2,225.36(1,583.19; 2,945.97) | 1.29(-6.52; 9.75) | 179.10(129.57; 235.19) | 87.64(62.88; 115.62) | -1.73(-6.22; 2.96) |
| Malta | male | 388.07(294.30; 496.22) | 685.61(561.52; 807.94) | 2.22(-4.58; 9.50) | 206.70(157.00; 265.17) | 329.88(269.35; 390.05) | 1.70(-4.32; 8.11) |
| Serbia | male | 4,761.53(3,598.24; 6,103.53) | 7,250.59(5,715.54; 8,819.15) | 1.18(-7.79; 11.02) | 102.03(76.86; 130.98) | 158.05(123.29; 193.04) | 1.70(-3.55; 7.24) |
| Kiribati | male | 47.72(32.78; 62.39) | 311.84(222.27; 426.02) | 3.66(-2.38; 10.06) | 131.25(90.82; 167.68) | 529.21(378.25; 711.59) | 1.66(-4.79; 8.54) |
| Botswana | male | 1,149.20(806.98; 1,552.72) | 1,086.62(813.62; 1,383.94) | 1.09(-6.02; 8.74) | 210.52(149.67; 278.42) | 89.99(68.12; 113.26) | -1.64(-6.15; 3.10) |
| Croatia | male | 3,674.47(2,730.98; 4,732.99) | 5,183.01(4,189.24; 6,154.65) | 0.94(-7.71; 10.41) | 146.87(108.76; 189.23) | 260.67(208.58; 311.30) | 1.64(-4.16; 7.78) |
| Czechia | male | 6,618.01(4,715.09; 9,058.43) | 8,729.76(6,937.74; 10,498.09) | 1.38(-7.78; 11.45) | 132.45(93.16; 182.23) | 185.60(146.49; 225.85) | 1.64(-3.76; 7.33) |
| Afghanistan | male | 4,740.80(3,577.44; 6,224.76) | 23,517.04(18,252.37; 30,437.58) | 5.43(-5.05; 17.08) | 115.09(87.14; 150.60) | 175.95(136.00; 229.54) | 1.64(-3.69; 7.27) |
| Qatar | male | 546.43(389.04; 731.72) | 2,597.50(1,861.75; 3,453.29) | 7.66(-0.88; 16.93) | 144.68(105.77; 188.67) | 81.22(58.71; 107.21) | -1.60(-6.03; 3.04) |
| Iceland | male | 435.97(348.69; 530.61) | 1,074.63(915.63; 1,238.25) | 2.51(-4.63; 10.18) | 316.33(253.31; 385.11) | 603.72(513.35; 698.66) | 1.57(-4.94; 8.52) |
| United Republic of Tanzania | male | 7,642.48(5,661.32; 10,172.64) | 32,953.16(22,373.11; 45,702.30) | 4.29(-6.40; 16.20) | 79.28(59.98; 102.52) | 146.87(99.14; 205.85) | 1.49(-3.64; 6.90) |
| Djibouti | male | 139.69(97.89; 190.71) | 873.39(567.56; 1,223.82) | 5.46(-1.71; 13.15) | 60.62(44.60; 80.79) | 125.73(82.36; 174.26) | 1.49(-3.50; 6.73) |
| Gabon | male | 874.42(627.54; 1,156.35) | 790.84(563.22; 1,030.60) | 0.59(-6.16; 7.82) | 200.87(147.41; 259.61) | 96.89(68.96; 125.33) | -1.48(-6.07; 3.33) |
| Bulgaria | male | 5,406.89(3,967.13; 7,158.30) | 5,524.83(4,398.12; 6,688.03) | 0.36(-8.26; 9.79) | 129.17(93.69; 173.16) | 185.93(146.40; 228.52) | 1.48(-3.89; 7.14) |
| Morocco | male | 14,794.23(10,758.31; 19,182.42) | 38,637.78(29,400.03; 51,321.51) | 3.17(-7.57; 15.16) | 127.23(93.83; 162.32) | 197.54(150.73; 261.80) | 1.47(-3.96; 7.20) |
| Namibia | male | 1,413.16(979.16; 1,919.28) | 1,137.04(857.40; 1,449.19) | 0.61(-6.51; 8.28) | 228.26(159.65; 304.42) | 102.63(78.76; 129.09) | -1.41(-6.07; 3.49) |
| Algeria | male | 14,414.72(10,990.43; 18,140.19) | 48,496.52(36,325.66; 60,499.99) | 3.87(-7.18; 16.22) | 130.13(101.48; 162.84) | 210.95(157.60; 261.78) | 1.40(-4.10; 7.21) |
| Senegal | male | 3,206.31(2,236.47; 4,418.52) | 2,837.93(1,916.42; 3,784.67) | 1.56(-6.51; 10.33) | 113.35(81.23; 153.77) | 39.85(27.21; 52.12) | -1.33(-5.07; 2.56) |
| Kazakhstan | male | 22,183.52(17,005.54; 28,437.20) | 43,468.46(34,666.46; 52,513.29) | 2.10(-8.65; 14.11) | 272.60(211.36; 346.46) | 449.76(358.27; 542.02) | 1.31(-4.96; 8.00) |
| Greenland | male | 108.00(81.57; 139.29) | 107.84(86.16; 132.70) | 0.88(-3.95; 5.94) | 289.82(219.02; 371.06) | 347.30(277.12; 429.30) | 1.31(-4.70; 7.69) |
| Luxembourg | male | 778.43(637.06; 936.39) | 1,103.36(916.27; 1,295.15) | 0.21(-6.85; 7.81) | 373.15(304.86; 448.87) | 327.06(269.27; 384.84) | -1.26(-7.06; 4.91) |
| Slovakia | male | 4,812.34(3,651.93; 6,069.28) | 3,590.23(2,748.12; 4,443.82) | -1.28(-9.37; 7.53) | 181.15(137.11; 228.05) | 136.60(103.91; 168.77) | -1.26(-6.21; 3.94) |
| Greece | male | 8,818.72(6,804.70; 11,034.71) | 17,891.43(15,094.25; 20,627.90) | 0.69(-9.14; 11.57) | 171.94(132.51; 215.22) | 395.16(330.91; 459.30) | 1.26(-4.91; 7.83) |
| France | male | 54,007.60(42,657.07; 67,116.16) | 78,031.49(64,361.72; 91,883.16) | 1.49(-9.76; 14.15) | 182.83(144.37; 227.14) | 244.88(200.96; 290.31) | 1.26(-4.40; 7.25) |
| Comoros | male | 107.44(75.37; 150.44) | 449.29(315.75; 619.61) | 3.49(-2.88; 10.29) | 58.89(42.21; 78.58) | 125.20(87.66; 172.00) | 1.26(-3.72; 6.49) |
| Hungary | male | 5,987.59(4,090.24; 8,489.95) | 6,495.52(5,027.44; 8,097.18) | 0.73(-8.08; 10.39) | 121.53(82.53; 172.12) | 151.22(114.66; 189.64) | 1.25(-3.91; 6.68) |
| Taiwan (Province of China) | male | 22,414.51(17,623.66; 27,814.00) | 31,481.09(25,567.78; 38,163.28) | 1.56(-8.83; 13.14) | 191.62(151.95; 234.57) | 243.66(195.16; 299.55) | 1.22(-4.41; 7.18) |
| Bhutan | male | 141.81(100.99; 190.00) | 333.58(237.48; 439.93) | 3.17(-2.90; 9.61) | 48.85(35.60; 64.18) | 75.55(53.74; 99.00) | 1.19(-3.26; 5.86) |
| Uzbekistan | male | 9,987.93(7,097.25; 13,707.17) | 20,642.12(15,238.45; 26,039.99) | 3.32(-6.84; 14.59) | 101.65(73.79; 136.52) | 114.37(84.69; 143.84) | 1.18(-3.71; 6.31) |
| Viet Nam | male | 54,676.12(39,980.70; 71,927.70) | 113,424.41(84,913.28; 145,835.03) | 2.88(-8.87; 16.15) | 166.67(123.20; 217.20) | 211.38(158.12; 270.00) | 1.15(-4.34; 6.95) |
| Sri Lanka | male | 17,800.86(13,660.60; 22,503.21) | 13,046.49(10,089.61; 16,246.96) | -0.57(-9.91; 9.74) | 199.77(155.53; 250.20) | 118.51(90.82; 148.76) | -1.12(-5.92; 3.92) |
| Cuba | male | 8,671.39(5,888.56; 11,721.13) | 4,397.03(3,137.46; 5,835.28) | -1.49(-9.74; 7.51) | 139.47(95.89; 187.28) | 80.19(56.47; 108.34) | -1.11(-5.53; 3.51) |
| Mongolia | male | 1,287.99(931.47; 1,716.83) | 2,462.22(1,842.24; 3,094.53) | 3.03(-5.04; 11.78) | 125.73(91.55; 165.63) | 148.91(111.93; 187.89) | 1.11(-4.04; 6.54) |
| Trinidad and Tobago | male | 705.93(480.93; 969.98) | 831.71(635.89; 1,036.32) | 1.72(-5.18; 9.12) | 114.74(79.60; 155.99) | 116.01(88.17; 145.41) | 1.11(-3.78; 6.26) |
| Kuwait | male | 1,222.52(873.82; 1,634.39) | 4,666.27(3,735.66; 5,750.43) | 4.71(-4.13; 14.36) | 99.49(72.66; 129.88) | 150.95(121.00; 185.63) | 1.08(-4.10; 6.53) |
| Bahrain | male | 449.61(308.37; 613.23) | 1,106.51(819.32; 1,402.94) | 3.90(-3.43; 11.78) | 124.56(86.77; 164.09) | 87.27(63.98; 111.24) | -1.07(-5.58; 3.65) |
| Sierra Leone | male | 1,718.08(1,170.48; 2,348.21) | 1,593.91(1,084.90; 2,172.57) | 2.84(-4.77; 11.05) | 117.21(80.95; 158.81) | 39.19(27.14; 52.49) | -1.07(-4.81; 2.81) |
| Iran (Islamic Republic of) | male | 93,947.16(74,598.39; 116,016.06) | 248,897.55(209,283.52; 286,773.35) | 3.57(-9.03; 17.92) | 369.17(299.51; 450.47) | 512.28(430.69; 590.62) | 1.05(-5.33; 7.86) |
| Central African Republic | male | 1,294.52(938.62; 1,699.83) | 1,601.60(1,116.89; 2,157.48) | 1.16(-6.32; 9.24) | 114.40(84.14; 147.97) | 69.94(49.21; 93.65) | -1.00(-5.29; 3.48) |
| Malawi | male | 2,545.33(1,803.31; 3,427.17) | 9,279.61(6,194.16; 13,247.93) | 3.41(-5.97; 13.72) | 67.21(49.09; 87.86) | 124.10(81.58; 176.47) | 0.98(-3.97; 6.18) |
| Belgium | male | 10,830.52(8,193.63; 13,891.92) | 15,241.33(12,619.80; 17,878.49) | 1.20(-8.43; 11.84) | 208.83(157.42; 267.75) | 282.26(232.51; 333.96) | 0.96(-4.77; 7.04) |
| Turkey | male | 26,806.44(18,658.67; 36,919.31) | 46,075.13(36,636.95; 57,232.98) | 2.43(-8.42; 14.55) | 91.45(64.61; 124.21) | 102.21(80.95; 127.46) | 0.94(-3.81; 5.93) |
| Peru | male | 25,392.61(16,408.52; 37,197.40) | 17,502.46(13,494.21; 21,941.30) | 0.90(-8.87; 11.72) | 240.21(157.66; 352.45) | 90.18(69.49; 112.72) | -0.92(-5.48; 3.87) |
| Eritrea | male | 878.23(628.01; 1,175.83) | 3,928.86(2,561.21; 5,752.62) | 4.28(-4.33; 13.65) | 73.62(54.57; 95.84) | 127.74(84.28; 188.54) | 0.91(-4.07; 6.13) |
| Eswatini | male | 650.23(457.76; 887.95) | 550.67(422.18; 692.61) | 0.95(-5.49; 7.83) | 215.55(154.09; 288.89) | 106.69(84.15; 131.56) | -0.90(-5.62; 4.07) |
| Bangladesh | male | 28,199.46(21,134.20; 35,598.15) | 61,484.12(42,463.25; 80,423.85) | 2.74(-8.41; 15.23) | 59.53(44.78; 75.40) | 74.62(51.53; 96.83) | 0.90(-3.53; 5.53) |
| Mauritania | male | 953.51(685.08; 1,291.14) | 749.74(526.18; 968.60) | 1.68(-5.10; 8.95) | 117.32(85.02; 155.46) | 40.96(29.34; 52.23) | -0.89(-4.68; 3.05) |
| Ukraine | male | 98,242.09(79,607.64; 119,932.57) | 91,213.47(70,783.05; 113,155.28) | 0.23(-11.03; 12.91) | 370.17(298.92; 454.97) | 430.88(337.95; 533.76) | 0.88(-5.30; 7.46) |
| Uganda | male | 4,754.12(3,264.37; 6,647.87) | 18,246.50(13,141.06; 24,868.30) | 3.85(-6.22; 15.01) | 69.98(50.32; 95.00) | 119.58(84.51; 162.83) | 0.88(-4.02; 6.04) |
| Mali | male | 2,422.38(1,710.64; 3,267.15) | 3,395.38(2,426.67; 4,529.48) | 2.71(-5.62; 11.77) | 77.08(55.34; 103.08) | 36.23(26.33; 47.20) | -0.85(-4.50; 2.94) |
| Brazil | male | 141,620.16(97,214.48; 196,844.70) | 180,547.97(141,220.75; 221,922.38) | 2.25(-9.88; 16.01) | 186.31(128.51; 255.32) | 157.73(123.17; 194.44) | 0.85(-4.35; 6.33) |
| Latvia | male | 6,771.46(4,981.18; 8,906.15) | 3,472.40(2,852.42; 4,057.98) | -2.12(-10.08; 6.55) | 511.72(374.82; 676.04) | 404.52(330.77; 475.41) | -0.84(-6.84; 5.54) |
| Bermuda | male | 68.46(50.73; 87.88) | 47.35(37.58; 56.71) | -1.12(-5.08; 3.00) | 204.13(151.27; 263.03) | 156.16(121.25; 188.64) | -0.82(-5.94; 4.57) |
| Guatemala | male | 6,028.16(4,819.77; 7,399.41) | 12,398.25(10,291.31; 14,452.58) | 2.72(-6.89; 13.31) | 196.70(161.03; 237.13) | 163.54(136.49; 191.05) | -0.81(-5.97; 4.63) |
| Bolivia (Plurinational State of) | male | 5,451.72(3,722.10; 7,549.10) | 6,557.54(4,842.59; 8,478.95) | 1.86(-7.05; 11.62) | 193.77(134.75; 264.25) | 105.60(77.85; 135.61) | -0.80(-5.51; 4.13) |
| Ethiopia | male | 14,844.85(11,210.84; 18,947.70) | 26,521.04(19,663.71; 34,199.10) | 2.22(-8.06; 13.65) | 79.70(62.00; 99.67) | 55.88(41.76; 70.11) | -0.79(-4.86; 3.46) |
| Côte d'Ivoire | male | 5,188.66(3,638.78; 6,912.83) | 4,954.96(3,313.30; 6,624.16) | 2.07(-6.60; 11.54) | 99.38(71.07; 129.12) | 38.75(26.22; 50.59) | -0.79(-4.53; 3.09) |
| Chad | male | 1,791.18(1,248.22; 2,435.34) | 2,394.52(1,640.84; 3,267.61) | 2.80(-5.20; 11.46) | 83.80(59.52; 113.35) | 36.68(25.83; 48.95) | -0.79(-4.46; 3.02) |
| Egypt | male | 18,937.52(12,867.47; 26,332.88) | 51,841.81(36,878.43; 66,935.19) | 3.21(-7.82; 15.56) | 72.35(49.61; 99.34) | 96.85(69.00; 124.51) | 0.78(-3.91; 5.70) |
| Saint Kitts and Nevis | male | 25.52(17.52; 34.62) | 28.03(20.35; 36.35) | 0.91(-2.56; 4.50) | 126.00(87.73; 168.32) | 87.15(62.15; 114.75) | -0.76(-5.28; 3.99) |
| India | male | 279,377.14(222,251.44; 346,832.59) | 748,504.65(597,726.13; 901,306.35) | 2.92(-10.60; 18.49) | 70.29(56.62; 86.10) | 97.14(78.39; 116.07) | 0.71(-3.98; 5.63) |
| Dominican Republic | male | 3,200.60(2,255.92; 4,405.78) | 4,218.52(2,917.58; 5,655.76) | 2.50(-6.05; 11.82) | 93.51(68.07; 124.85) | 70.78(49.06; 94.88) | 0.70(-3.68; 5.29) |
| Barbados | male | 173.34(121.45; 239.29) | 128.52(94.02; 167.17) | -0.37(-5.29; 4.80) | 130.35(91.21; 177.65) | 90.94(65.98; 120.38) | -0.69(-5.25; 4.10) |
| Oman | male | 950.99(666.13; 1,306.36) | 3,241.56(2,393.61; 4,122.09) | 5.65(-2.89; 14.95) | 79.80(56.98; 105.91) | 84.11(62.84; 106.48) | 0.69(-3.85; 5.45) |
| Gambia | male | 434.75(295.57; 604.09) | 428.08(293.76; 562.11) | 2.34(-3.93; 9.01) | 109.56(76.25; 148.50) | 40.70(28.30; 52.62) | -0.68(-4.48; 3.26) |
| Saudi Arabia | male | 8,042.91(5,697.62; 10,947.07) | 24,874.49(17,931.03; 33,109.47) | 4.82(-5.66; 16.47) | 91.69(66.56; 122.16) | 85.33(60.99; 113.77) | 0.68(-3.88; 5.46) |
| Kenya | male | 5,530.95(3,970.30; 7,513.21) | 14,180.32(10,648.44; 17,406.55) | 3.83(-6.00; 14.69) | 62.92(46.07; 83.91) | 67.38(51.56; 81.07) | 0.68(-3.64; 5.19) |
| Equatorial Guinea | male | 209.96(151.07; 280.33) | 686.01(504.32; 947.36) | 4.99(-1.89; 12.36) | 140.50(101.09; 186.11) | 89.78(64.72; 121.62) | -0.67(-5.22; 4.09) |
| Cyprus | male | 760.36(587.83; 949.87) | 1,640.12(1,298.12; 1,999.24) | 2.76(-4.85; 10.98) | 182.22(140.73; 227.06) | 227.26(177.15; 278.65) | 0.67(-4.85; 6.51) |
| Netherlands | male | 15,735.42(11,671.04; 20,485.82) | 18,790.23(15,318.02; 22,828.50) | 0.51(-9.28; 11.36) | 190.37(140.86; 248.02) | 231.13(186.07; 282.58) | 0.65(-4.90; 6.52) |
| United States Virgin Islands | male | 141.49(113.91; 173.21) | 121.95(92.04; 164.07) | -1.18(-6.12; 4.03) | 279.32(224.93; 341.30) | 363.56(268.77; 505.76) | -0.63(-6.67; 5.80) |
| Zimbabwe | male | 8,819.08(6,157.27; 12,411.86) | 7,666.87(5,894.61; 9,524.18) | 0.48(-8.45; 10.29) | 219.97(157.60; 297.36) | 131.51(103.12; 159.96) | -0.63(-5.57; 4.56) |
| Venezuela (Bolivarian Republic of) | male | 9,109.70(6,303.37; 12,405.96) | 6,797.73(4,817.12; 8,889.60) | 0.88(-8.02; 10.64) | 100.06(69.52; 133.94) | 55.45(39.34; 73.11) | -0.63(-4.72; 3.64) |
| Liberia | male | 736.05(523.47; 991.48) | 1,051.74(716.58; 1,418.46) | 3.55(-3.69; 11.34) | 104.56(74.65; 139.88) | 41.26(28.94; 54.25) | -0.62(-4.42; 3.34) |
| Rwanda | male | 1,876.66(1,327.20; 2,491.03) | 7,402.94(4,868.58; 10,339.95) | 3.94(-5.26; 14.04) | 66.90(49.01; 86.26) | 126.54(83.82; 174.52) | 0.62(-4.34; 5.82) |
| South Sudan | male | 1,644.57(1,141.27; 2,268.40) | 3,395.60(2,343.63; 4,999.08) | 1.53(-6.71; 10.51) | 61.71(44.06; 83.14) | 98.70(67.36; 143.78) | 0.62(-4.08; 5.55) |
| Nepal | male | 4,160.09(2,918.54; 5,710.74) | 10,883.90(7,987.65; 14,095.71) | 2.44(-7.01; 12.85) | 51.04(36.24; 69.11) | 74.61(55.69; 95.93) | 0.59(-3.84; 5.21) |
| Democratic People's Republic of Korea | male | 19,574.12(14,154.91; 25,661.44) | 19,165.26(14,424.92; 25,029.95) | 0.78(-9.06; 11.69) | 195.13(142.50; 254.78) | 129.00(96.34; 167.70) | -0.58(-5.49; 4.59) |
| Austria | male | 11,867.72(9,065.96; 14,792.99) | 15,035.63(12,345.51; 17,622.15) | 0.73(-8.88; 11.35) | 281.64(214.51; 351.69) | 345.14(281.00; 405.50) | 0.58(-5.37; 6.91) |
| Niger | male | 2,345.89(1,653.77; 3,172.52) | 3,442.03(2,345.46; 4,652.86) | 3.01(-5.36; 12.11) | 80.60(57.84; 108.08) | 37.04(26.00; 48.78) | -0.58(-4.27; 3.26) |
| Somalia | male | 1,632.09(1,169.62; 2,226.02) | 7,809.50(4,938.82; 12,163.79) | 4.45(-4.85; 14.66) | 59.70(43.38; 80.46) | 93.46(57.35; 147.38) | 0.58(-4.07; 5.45) |
| Micronesia (Federated States of) | male | 69.96(51.98; 91.02) | 52.05(36.43; 67.29) | -0.10(-4.14; 4.10) | 151.11(114.18; 194.90) | 93.28(65.38; 120.44) | -0.57(-5.16; 4.25) |
| Jamaica | male | 1,689.14(1,129.28; 2,400.67) | 1,534.42(1,092.12; 2,045.40) | 0.42(-6.97; 8.40) | 143.60(97.37; 199.99) | 98.15(69.95; 131.09) | -0.56(-5.20; 4.31) |
| Cameroon | male | 5,707.13(3,994.66; 7,538.54) | 6,158.97(4,333.68; 8,145.16) | 3.63(-5.38; 13.51) | 144.55(102.26; 190.12) | 44.96(32.10; 58.67) | -0.55(-4.47; 3.53) |
| Montenegro | male | 533.61(373.91; 713.43) | 289.08(206.16; 374.38) | -0.82(-6.53; 5.24) | 158.67(111.68; 211.57) | 95.37(67.82; 123.65) | -0.54(-5.18; 4.32) |
| Nigeria | male | 35,644.74(25,473.01; 47,510.64) | 31,445.60(23,034.52; 40,976.46) | 2.13(-8.33; 13.77) | 91.03(65.57; 119.91) | 35.23(26.07; 44.92) | -0.54(-4.20; 3.26) |
| Democratic Republic of the Congo | male | 15,806.69(11,477.90; 20,993.97) | 29,446.08(20,541.47; 40,223.01) | 2.63(-7.79; 14.23) | 104.20(77.28; 135.19) | 74.92(52.63; 102.09) | -0.53(-4.90; 4.05) |
| Andorra | male | 62.37(44.37; 83.23) | 54.76(39.15; 71.23) | 0.65(-3.48; 4.95) | 176.34(126.67; 236.41) | 128.54(91.24; 169.92) | 0.53(-4.44; 5.75) |
| Republic of Moldova | male | 5,607.74(4,201.17; 7,292.87) | 3,756.72(2,919.66; 4,528.08) | -0.80(-8.95; 8.07) | 258.85(194.09; 335.54) | 200.65(155.03; 244.53) | -0.51(-5.84; 5.13) |
| Thailand | male | 70,325.44(46,296.72; 101,522.50) | 44,917.07(31,555.02; 60,289.88) | -0.51(-11.04; 11.26) | 215.94(144.12; 307.28) | 146.94(101.26; 201.75) | -0.48(-5.54; 4.86) |
| Zambia | male | 2,982.47(2,160.32; 3,959.93) | 10,922.56(7,628.01; 15,479.69) | 3.85(-5.72; 14.39) | 99.72(74.70; 129.83) | 138.53(95.86; 197.04) | 0.48(-4.55; 5.78) |
| Maldives | male | 89.76(61.98; 124.76) | 400.56(287.67; 519.77) | 5.95(-0.45; 12.77) | 92.53(65.40; 126.50) | 92.40(65.96; 119.22) | 0.48(-4.15; 5.33) |
| Northern Mariana Islands | male | 39.71(29.24; 51.92) | 27.34(19.58; 36.33) | -1.69(-5.06; 1.80) | 137.98(103.56; 178.68) | 110.62(78.04; 149.70) | -0.46(-5.21; 4.54) |
| Bahamas | male | 192.40(135.28; 260.73) | 206.22(152.02; 268.65) | 0.77(-4.68; 6.53) | 138.65(97.69; 182.97) | 100.63(74.17; 131.05) | -0.46(-5.14; 4.45) |
| Armenia | male | 2,331.80(1,603.33; 3,270.38) | 1,705.80(1,203.91; 2,193.89) | 0.20(-7.29; 8.29) | 132.52(92.57; 183.97) | 114.19(81.13; 146.07) | 0.46(-4.38; 5.55) |
| Guinea-Bissau | male | 336.62(240.35; 439.30) | 332.77(229.09; 439.05) | 2.42(-3.60; 8.81) | 92.06(66.51; 119.43) | 38.38(26.81; 49.80) | -0.46(-4.20; 3.42) |
| Denmark | male | 11,744.52(9,620.27; 13,935.67) | 15,127.95(12,626.67; 17,499.24) | 0.47(-9.14; 11.09) | 424.77(348.07; 502.74) | 522.53(437.99; 608.69) | 0.45(-5.91; 7.25) |
| Niue | male | 1.43(1.07; 1.87) | 0.74(0.54; 0.94) | -1.57(-1.98; -1.16) | 134.21(100.64; 174.93) | 90.90(66.05; 116.90) | -0.44(-5.01; 4.35) |
| Ecuador | male | 5,466.72(3,839.13; 7,304.26) | 9,963.81(7,982.66; 12,299.19) | 2.69(-6.70; 13.02) | 116.66(83.46; 154.09) | 106.26(85.25; 131.42) | 0.44(-4.32; 5.45) |
| Madagascar | male | 3,389.33(2,493.74; 4,514.85) | 11,593.49(7,988.26; 15,724.03) | 3.49(-6.11; 14.07) | 70.04(53.01; 90.86) | 100.09(69.71; 134.75) | 0.44(-4.27; 5.37) |
| Portugal | male | 7,410.75(5,536.65; 9,551.96) | 8,656.21(7,122.17; 10,314.48) | -0.70(-9.67; 9.16) | 146.90(109.49; 189.37) | 179.67(147.62; 213.90) | -0.43(-5.70; 5.13) |
| Grenada | male | 54.79(38.24; 75.40) | 87.65(67.29; 110.04) | 1.58(-3.11; 6.49) | 137.13(97.20; 184.17) | 152.19(116.76; 190.64) | 0.42(-4.73; 5.85) |
| Antigua and Barbuda | male | 37.52(26.20; 51.22) | 47.65(35.82; 61.54) | 1.08(-2.98; 5.31) | 122.95(86.74; 165.54) | 100.12(74.90; 130.23) | -0.41(-5.13; 4.54) |
| Belize | male | 101.74(70.14; 141.30) | 205.94(150.98; 283.48) | 2.84(-2.70; 8.69) | 113.82(79.77; 154.93) | 86.97(64.21; 118.45) | -0.41(-4.94; 4.34) |
| Germany | male | 117,299.52(95,250.73; 143,022.54) | 138,069.28(113,570.56; 162,447.89) | 0.20(-11.42; 13.36) | 273.02(220.53; 334.24) | 328.36(269.99; 385.68) | 0.38(-5.50; 6.63) |
| Uruguay | male | 3,494.63(2,302.27; 4,863.91) | 2,279.62(1,687.36; 2,905.89) | -0.11(-7.84; 8.27) | 232.00(152.75; 324.24) | 137.20(100.82; 176.30) | -0.38(-5.35; 4.86) |
| Saint Vincent and the Grenadines | male | 65.85(44.94; 90.51) | 57.80(42.31; 75.26) | -0.16(-4.31; 4.17) | 120.48(84.52; 162.61) | 97.82(71.18; 128.57) | -0.38(-5.05; 4.51) |
| Angola | male | 4,814.57(3,462.97; 6,326.77) | 9,902.11(7,056.32; 12,972.20) | 2.99(-6.40; 13.34) | 109.46(80.30; 141.85) | 80.60(56.93; 104.53) | -0.38(-4.83; 4.29) |
| Honduras | male | 2,668.63(2,061.67; 3,330.21) | 7,092.45(5,173.86; 9,381.83) | 3.46(-5.66; 13.45) | 150.93(120.49; 186.00) | 151.35(112.22; 198.92) | 0.37(-4.74; 5.75) |
| Kyrgyzstan | male | 4,137.64(3,273.14; 5,194.43) | 7,130.19(5,682.14; 8,860.70) | 1.51(-7.44; 11.33) | 210.46(169.92; 259.30) | 208.08(167.11; 257.17) | -0.35(-5.75; 5.36) |
| Suriname | male | 226.56(158.04; 308.93) | 367.29(287.20; 462.55) | 1.73(-4.38; 8.23) | 110.53(78.23; 148.98) | 126.10(98.26; 159.21) | 0.35(-4.62; 5.57) |
| Togo | male | 1,084.84(782.75; 1,440.29) | 1,408.24(961.52; 1,895.81) | 3.55(-3.99; 11.68) | 81.25(60.42; 105.18) | 38.95(27.55; 51.61) | 0.35(-3.44; 4.29) |
| Myanmar | male | 20,478.36(14,708.13; 27,024.83) | 21,820.13(14,999.64; 29,074.51) | 0.67(-9.28; 11.72) | 101.31(74.39; 132.46) | 76.48(52.88; 101.29) | -0.34(-4.75; 4.27) |
| Cabo Verde | male | 187.65(130.17; 255.26) | 145.54(104.51; 187.23) | 2.83(-2.42; 8.36) | 146.10(102.91; 195.08) | 46.11(33.14; 58.85) | -0.34(-4.29; 3.78) |
| Colombia | male | 24,274.65(17,800.34; 31,631.07) | 27,971.48(21,505.14; 35,996.40) | 1.16(-9.07; 12.54) | 150.08(111.50; 193.91) | 104.75(80.54; 134.74) | -0.33(-5.04; 4.61) |
| Nauru | male | 6.87(5.02; 8.85) | 5.21(3.62; 6.86) | 0.00(-1.71; 1.74) | 140.33(103.56; 178.92) | 90.82(63.97; 118.90) | -0.33(-4.91; 4.47) |
| Republic of Korea | male | 31,465.55(21,194.52; 43,521.94) | 28,043.48(19,560.23; 36,344.35) | -0.15(-10.26; 11.11) | 120.63(83.32; 165.80) | 105.06(72.36; 138.96) | -0.32(-5.04; 4.64) |
| Lesotho | male | 1,255.39(882.78; 1,704.40) | 784.44(584.64; 1,028.61) | 0.61(-6.15; 7.86) | 161.30(115.06; 217.18) | 91.72(69.76; 117.08) | -0.32(-4.92; 4.50) |
| New Zealand | male | 5,449.25(3,916.97; 7,414.19) | 8,841.19(7,020.53; 10,647.14) | 1.03(-8.05; 11.01) | 302.17(217.10; 410.98) | 337.90(265.82; 409.08) | 0.31(-5.56; 6.55) |
| Paraguay | male | 2,067.74(1,393.25; 2,901.47) | 3,125.99(2,240.06; 4,119.94) | 2.21(-6.01; 11.15) | 109.34(74.61; 151.65) | 78.80(56.72; 103.44) | -0.31(-4.75; 4.34) |
| Indonesia | male | 75,396.67(52,248.79; 104,222.87) | 87,323.78(61,575.02; 117,202.46) | 1.10(-10.20; 13.82) | 81.46(57.45; 111.18) | 56.87(40.19; 76.24) | -0.30(-4.42; 3.99) |
| Pakistan | male | 60,421.07(42,026.48; 83,541.33) | 80,492.86(62,935.01; 99,128.43) | 2.44(-8.95; 15.26) | 125.50(88.99; 170.76) | 72.49(56.40; 89.26) | -0.29(-4.71; 4.34) |
| Tonga | male | 43.71(30.84; 60.28) | 41.78(29.99; 54.76) | 0.50(-3.34; 4.49) | 99.04(71.43; 134.95) | 85.60(61.47; 110.57) | 0.29(-4.26; 5.05) |
| Azerbaijan | male | 4,230.07(2,962.10; 5,761.06) | 6,603.96(4,904.50; 8,425.78) | 2.02(-6.91; 11.82) | 113.29(80.20; 153.10) | 112.68(82.94; 143.51) | 0.28(-4.54; 5.34) |
| Lao People's Democratic Republic | male | 1,372.97(989.41; 1,834.08) | 2,451.90(1,754.16; 3,244.13) | 2.51(-5.49; 11.19) | 77.63(56.92; 102.43) | 61.17(44.59; 80.19) | -0.28(-4.47; 4.09) |
| Japan | male | 72,562.36(52,313.42; 96,808.33) | 48,152.34(35,543.05; 61,573.50) | -0.87(-11.41; 10.92) | 113.00(80.70; 151.76) | 87.48(63.21; 114.52) | -0.26(-4.80; 4.49) |
| Fiji | male | 381.26(267.51; 518.18) | 349.41(240.72; 459.16) | 0.38(-5.56; 6.70) | 96.06(68.87; 128.95) | 73.61(50.91; 96.56) | -0.26(-4.63; 4.31) |
| Sao Tome and Principe | male | 54.41(37.41; 74.68) | 68.40(51.94; 88.46) | 2.52(-1.89; 7.14) | 121.39(85.53; 162.19) | 66.34(50.46; 83.91) | -0.26(-4.54; 4.21) |
| Mexico | male | 50,964.11(37,769.02; 66,578.26) | 63,857.79(51,045.21; 78,711.54) | 1.31(-9.71; 13.68) | 126.54(94.70; 161.11) | 95.33(76.18; 117.43) | -0.25(-4.87; 4.60) |
| Haiti | male | 2,928.04(2,001.63; 4,095.97) | 6,359.65(4,464.22; 8,362.70) | 3.04(-5.92; 12.86) | 104.59(72.55; 145.55) | 97.28(69.72; 126.78) | 0.25(-4.41; 5.13) |
| Costa Rica | male | 1,725.47(1,254.24; 2,329.86) | 2,331.06(1,866.73; 2,867.95) | 1.88(-6.02; 10.43) | 115.10(84.80; 151.89) | 93.88(75.06; 115.99) | 0.25(-4.37; 5.09) |
| Guyana | male | 424.61(295.01; 578.04) | 300.22(210.46; 395.36) | -0.19(-5.95; 5.93) | 108.32(76.63; 145.22) | 73.99(52.33; 97.37) | -0.23(-4.61; 4.34) |
| Ghana | male | 7,639.01(5,273.38; 10,402.49) | 6,347.65(4,426.56; 8,630.91) | 3.36(-5.67; 13.26) | 127.70(90.02; 172.49) | 40.43(28.34; 54.55) | 0.23(-3.64; 4.25) |
| Saint Lucia | male | 86.69(59.50; 118.06) | 92.32(68.51; 120.21) | 0.96(-3.75; 5.89) | 128.71(90.95; 173.88) | 96.93(71.37; 126.53) | -0.22(-4.92; 4.71) |
| Israel | male | 4,422.79(3,442.72; 5,551.60) | 9,310.44(7,613.35; 11,205.50) | 2.29(-7.01; 12.52) | 186.68(145.91; 233.26) | 203.75(166.48; 245.51) | 0.21(-5.22; 5.94) |
| Burundi | male | 1,469.03(1,042.20; 1,962.58) | 5,655.73(3,805.26; 8,052.05) | 3.69(-5.23; 13.44) | 68.53(50.40; 89.28) | 102.63(68.70; 148.63) | 0.21(-4.52; 5.16) |
| Norway | male | 8,611.22(7,219.07; 10,181.34) | 16,844.02(14,182.42; 19,372.23) | 0.92(-8.86; 11.74) | 385.00(322.47; 455.50) | 605.77(506.93; 700.48) | 0.20(-6.35; 7.20) |
| Dominica | male | 51.38(35.66; 71.92) | 46.34(34.42; 60.45) | -0.30(-4.22; 3.79) | 135.40(95.25; 187.53) | 128.37(95.26; 167.40) | -0.20(-5.14; 5.01) |
| Singapore | male | 2,207.84(1,493.54; 3,092.20) | 3,042.42(2,183.18; 4,084.33) | 1.87(-6.32; 10.76) | 114.12(77.80; 159.34) | 103.45(73.74; 139.32) | 0.20(-4.53; 5.16) |
| Timor-Leste | male | 357.11(240.54; 508.34) | 546.66(374.95; 766.71) | 1.95(-4.52; 8.85) | 88.88(60.98; 124.98) | 78.21(54.40; 106.74) | 0.20(-4.25; 4.86) |
| Samoa | male | 96.63(70.20; 132.22) | 101.86(72.58; 133.67) | 0.45(-4.28; 5.40) | 112.81(83.97; 149.57) | 95.70(68.49; 124.65) | -0.19(-4.83; 4.67) |
| Solomon Islands | male | 176.68(127.62; 234.65) | 284.83(203.94; 373.13) | 2.20(-3.63; 8.40) | 114.88(84.56; 149.65) | 82.95(59.62; 107.12) | -0.19(-4.68; 4.52) |
| Benin | male | 1,393.84(1,011.23; 1,871.48) | 2,217.82(1,519.10; 2,965.69) | 3.79(-4.21; 12.46) | 83.92(61.50; 110.45) | 39.55(27.75; 52.05) | -0.19(-3.97; 3.74) |
| Iraq | male | 13,194.11(9,880.45; 16,988.09) | 43,499.93(33,419.98; 56,939.43) | 3.29(-7.58; 15.45) | 180.76(137.13; 229.47) | 199.32(154.06; 262.01) | -0.16(-5.53; 5.51) |
| Papua New Guinea | male | 1,766.64(1,217.99; 2,412.59) | 4,248.64(2,952.14; 5,760.34) | 3.43(-5.18; 12.82) | 84.25(59.04; 114.43) | 77.30(53.91; 103.96) | 0.16(-4.28; 4.80) |
| American Samoa | male | 29.14(21.70; 37.87) | 23.20(16.95; 29.61) | -0.29(-3.53; 3.05) | 119.52(90.24; 153.22) | 96.91(70.91; 123.13) | -0.13(-4.79; 4.75) |
| Marshall Islands | male | 26.53(19.56; 34.48) | 27.84(19.69; 36.18) | 1.16(-2.30; 4.73) | 131.69(98.59; 168.71) | 91.30(65.29; 117.99) | -0.13(-4.72; 4.68) |
| Guam | male | 105.68(77.38; 139.58) | 70.81(50.95; 91.45) | -0.17(-4.52; 4.38) | 123.69(92.37; 162.30) | 86.67(61.95; 112.64) | -0.13(-4.68; 4.63) |
| Palau | male | 10.65(7.78; 14.18) | 8.19(5.68; 10.70) | -0.05(-2.28; 2.23) | 120.45(88.44; 158.71) | 80.55(54.75; 106.79) | -0.13(-4.60; 4.55) |
| Chile | male | 12,138.68(8,278.12; 16,616.90) | 13,794.06(10,365.66; 17,594.45) | 0.97(-8.57; 11.50) | 173.58(118.44; 235.53) | 139.83(104.42; 178.72) | -0.12(-5.13; 5.15) |
| Malaysia | male | 12,021.82(8,586.12; 15,932.31) | 15,861.43(11,378.73; 20,667.09) | 2.36(-7.45; 13.21) | 133.43(96.44; 175.73) | 83.82(60.22; 108.29) | -0.12(-4.62; 4.60) |
| El Salvador | male | 1,933.74(1,396.04; 2,582.37) | 2,932.63(2,332.91; 3,584.06) | 1.13(-6.92; 9.87) | 86.76(63.56; 113.27) | 95.62(76.67; 116.64) | 0.11(-4.51; 4.96) |
| Panama | male | 1,187.10(826.65; 1,603.02) | 1,912.50(1,474.70; 2,406.32) | 1.97(-5.74; 10.31) | 96.52(68.25; 128.19) | 85.82(66.30; 107.61) | 0.11(-4.43; 4.86) |
| Australia | male | 52,389.54(41,594.50; 65,061.70) | 86,879.67(74,408.55; 100,170.58) | 1.03(-10.25; 13.73) | 581.78(460.74; 722.79) | 672.37(571.25; 780.72) | -0.10(-6.66; 6.92) |
| Poland | male | 33,397.61(25,427.40; 43,613.63) | 29,438.32(23,644.50; 35,529.29) | -0.12(-10.28; 11.19) | 176.87(133.52; 233.09) | 165.73(131.97; 201.49) | -0.10(-5.27; 5.36) |
| Monaco | male | 27.48(19.55; 36.33) | 25.46(18.63; 32.11) | 0.49(-2.85; 3.94) | 200.24(143.09; 265.87) | 166.64(120.84; 214.32) | 0.10(-5.10; 5.59) |
| Tuvalu | male | 4.98(3.73; 6.55) | 6.06(4.38; 8.07) | 1.62(-0.27; 3.54) | 118.09(89.75; 154.36) | 89.38(64.98; 118.20) | -0.09(-4.67; 4.70) |
| Tajikistan | male | 2,695.41(1,882.79; 3,673.61) | 5,839.11(4,098.44; 7,469.25) | 2.71(-6.16; 12.42) | 113.04(82.20; 149.75) | 110.11(77.80; 139.86) | -0.07(-4.85; 4.95) |
| Romania | male | 11,924.56(9,421.36; 14,752.33) | 10,959.28(9,166.97; 12,876.59) | -0.81(-9.98; 9.29) | 100.87(79.50; 125.06) | 116.94(97.83; 138.47) | -0.06(-4.90; 5.02) |
| Vanuatu | male | 67.12(49.27; 92.00) | 124.58(90.53; 163.30) | 2.67(-2.36; 7.95) | 96.38(71.12; 130.55) | 80.19(58.64; 104.36) | 0.06(-4.41; 4.74) |
| Guinea | male | 1,618.72(1,153.57; 2,187.53) | 1,886.54(1,316.28; 2,517.55) | 2.47(-5.27; 10.84) | 71.05(51.23; 95.03) | 35.97(25.53; 47.45) | -0.06(-3.74; 3.77) |
| Belarus | male | 18,634.91(13,785.76; 24,502.75) | 16,896.23(13,540.93; 20,460.34) | -0.22(-9.84; 10.43) | 351.52(260.24; 461.25) | 354.24(286.34; 428.57) | 0.05(-5.88; 6.36) |
| Georgia | male | 4,080.16(2,927.35; 5,353.17) | 2,195.46(1,727.51; 2,735.74) | -1.35(-8.96; 6.89) | 148.22(106.77; 192.69) | 126.89(99.14; 158.75) | 0.05(-4.87; 5.22) |
| Jordan | male | 2,018.65(1,422.75; 2,720.14) | 5,843.95(4,009.41; 7,935.20) | 4.63(-4.41; 14.52) | 109.06(77.89; 143.74) | 79.57(54.80; 106.47) | 0.05(-4.41; 4.73) |
| Seychelles | male | 55.46(38.92; 75.17) | 76.72(59.66; 93.33) | 1.58(-2.97; 6.35) | 142.19(101.11; 191.26) | 126.13(95.91; 155.71) | -0.04(-5.01; 5.20) |
| Burkina Faso | male | 2,395.68(1,743.11; 3,138.66) | 3,490.62(2,386.92; 4,668.78) | 3.64(-4.80; 12.83) | 79.19(58.77; 102.82) | 41.30(29.38; 54.19) | 0.04(-3.78; 4.02) |
| Russian Federation | male | 454,993.47(364,079.57; 561,049.87) | 489,209.62(420,568.54; 557,042.48) | -0.14(-12.90; 14.50) | 587.31(469.68; 724.79) | 684.51(584.79; 783.84) | -0.03(-6.64; 7.04) |
| San Marino | male | 25.18(17.74; 33.89) | 21.51(15.04; 28.13) | 0.48(-2.69; 3.76) | 202.25(142.44; 273.20) | 154.24(107.32; 203.13) | 0.03(-5.09; 5.43) |
| Cook Islands | male | 10.99(7.77; 14.62) | 7.09(5.09; 9.18) | -0.78(-2.78; 1.27) | 112.41(80.51; 149.03) | 87.60(62.47; 113.53) | 0.03(-4.52; 4.81) |
| Palestine | male | 819.24(570.10; 1,120.75) | 1,967.79(1,368.95; 2,614.71) | 3.70(-4.18; 12.22) | 95.75(68.75; 126.78) | 72.61(50.14; 96.17) | -0.03(-4.40; 4.54) |
| Argentina | male | 23,519.86(15,629.74; 33,398.89) | 28,595.29(19,990.84; 38,315.74) | 1.34(-8.93; 12.77) | 149.11(99.14; 211.62) | 121.76(85.04; 163.62) | 0.02(-4.86; 5.15) |
| Tokelau | male | 0.77(0.55; 1.03) | 0.61(0.45; 0.78) | -0.28(-0.85; 0.30) | 107.70(77.73; 142.22) | 89.41(65.40; 113.80) | 0.01(-4.57; 4.80) |
| Cambodia | male | 3,691.60(2,525.08; 5,191.38) | 6,881.01(4,843.92; 9,377.57) | 2.64(-6.38; 12.54) | 84.73(59.04; 116.84) | 75.62(53.88; 102.19) | 0.01(-4.40; 4.62) |
| United States of America | female | 508,551.62(373,902.40; 656,572.21) | 2,555,018.47(2,060,825.51; 3,035,865.98) | 5.69(-9.37; 23.24) | 371.29(271.97; 482.39) | 1,511.82(1,217.96; 1,805.58) | 4.93(-2.77; 13.25) |
| Canada | female | 35,653.25(26,630.14; 45,794.16) | 112,303.71(94,695.15; 131,855.15) | 4.19(-7.70; 17.62) | 241.55(180.23; 310.75) | 602.60(503.00; 708.30) | 3.45(-3.24; 10.59) |
| South Africa | female | 86,748.54(59,020.32; 119,075.45) | 43,254.78(34,696.01; 52,168.41) | -1.96(-12.29; 9.58) | 446.46(308.40; 605.31) | 139.13(112.05; 167.69) | -3.43(-8.29; 1.69) |
| Slovenia | female | 527.97(375.27; 697.71) | 791.80(591.51; 997.69) | 2.57(-4.36; 10.01) | 51.45(36.34; 68.44) | 84.47(60.51; 107.44) | 2.94(-1.75; 7.86) |
| Sweden | female | 3,864.87(2,980.57; 4,913.92) | 10,611.80(8,537.72; 12,494.44) | 3.28(-6.23; 13.76) | 89.47(68.38; 114.88) | 209.86(167.57; 249.09) | 2.78(-2.79; 8.68) |
| China | female | 1,564,991.61(1,204,625.56; 1,958,237.93) | 639,530.02(471,656.75; 805,252.95) | -2.11(-14.84; 12.54) | 247.52(194.37; 305.23) | 91.77(66.46; 116.92) | -2.66(-7.15; 2.04) |
| Libya | female | 1,949.84(1,382.68; 2,583.33) | 5,837.70(4,398.72; 7,243.96) | 5.86(-3.30; 15.88) | 125.62(91.64; 161.94) | 150.58(114.29; 187.11) | 2.57(-2.67; 8.09) |
| France | female | 30,229.51(21,923.97; 39,348.26) | 60,368.64(49,288.86; 70,799.78) | 2.96(-8.22; 15.51) | 99.21(71.49; 130.46) | 156.49(125.96; 186.93) | 2.43(-2.87; 8.03) |
| Turkmenistan | female | 1,220.54(840.12; 1,678.30) | 4,227.22(3,215.51; 5,308.08) | 3.58(-5.06; 12.99) | 66.97(46.78; 91.21) | 164.85(125.52; 206.93) | 2.41(-2.90; 8.01) |
| Finland | female | 3,821.67(3,039.50; 4,781.30) | 7,965.65(6,663.76; 9,186.04) | 2.41(-6.75; 12.47) | 143.54(112.05; 181.84) | 289.26(242.11; 336.87) | 2.40(-3.48; 8.65) |
| Ireland | female | 2,120.17(1,527.89; 2,774.65) | 6,644.26(5,429.57; 7,910.46) | 3.76(-5.36; 13.75) | 118.86(85.76; 156.03) | 260.39(210.00; 313.67) | 2.39(-3.40; 8.53) |
| Estonia | female | 1,520.69(1,123.89; 1,965.39) | 1,883.34(1,427.49; 2,322.62) | 1.41(-6.25; 9.69) | 187.06(138.32; 244.00) | 322.73(239.95; 401.57) | 2.35(-3.62; 8.68) |
| Tunisia | female | 3,334.39(2,314.45; 4,586.49) | 7,077.79(5,105.89; 9,093.08) | 3.44(-5.69; 13.46) | 83.94(59.24; 112.50) | 113.71(81.20; 147.05) | 1.88(-3.03; 7.04) |
| United Kingdom | female | 61,251.11(46,036.26; 78,018.95) | 127,480.97(105,405.40; 147,735.41) | 2.33(-9.46; 15.66) | 202.90(150.99; 259.27) | 367.15(300.44; 426.95) | 1.84(-4.23; 8.29) |
| Qatar | female | 121.62(78.47; 175.43) | 1,121.82(769.89; 1,521.23) | 9.30(1.57; 17.61) | 82.60(54.67; 117.21) | 105.08(72.93; 144.75) | 1.80(-3.03; 6.88) |
| Zimbabwe | female | 9,520.46(6,101.75; 13,871.48) | 6,400.65(4,615.48; 8,407.32) | -0.33(-9.03; 9.20) | 212.41(138.77; 301.24) | 86.56(63.52; 111.78) | -1.76(-6.23; 2.93) |
| Yemen | female | 2,914.84(2,069.65; 3,882.98) | 15,996.43(11,460.84; 21,012.31) | 5.70(-4.43; 16.90) | 59.77(43.61; 77.84) | 104.79(77.11; 135.05) | 1.74(-3.08; 6.80) |
| Greenland | female | 64.29(48.45; 81.47) | 90.06(70.70; 108.64) | 1.74(-2.94; 6.64) | 216.71(165.30; 271.77) | 316.42(248.76; 384.86) | 1.73(-4.20; 8.03) |
| Syrian Arab Republic | female | 4,766.88(3,541.00; 6,209.03) | 10,418.21(7,827.69; 13,136.92) | 2.65(-6.78; 13.04) | 91.03(68.82; 116.25) | 144.00(108.23; 180.45) | 1.73(-3.41; 7.14) |
| Uzbekistan | female | 5,297.78(3,570.19; 7,546.96) | 18,209.89(12,871.23; 23,493.28) | 3.82(-6.27; 14.99) | 52.51(35.89; 73.47) | 101.12(71.79; 130.71) | 1.73(-3.04; 6.75) |
| Bosnia and Herzegovina | female | 888.13(572.34; 1,283.40) | 773.81(532.40; 1,040.55) | 0.69(-6.07; 7.94) | 36.42(23.62; 52.61) | 47.24(32.63; 64.08) | 1.68(-2.34; 5.85) |
| Switzerland | female | 10,862.78(8,687.80; 13,234.52) | 8,421.87(6,807.28; 9,950.41) | -0.92(-9.83; 8.86) | 302.31(240.62; 370.14) | 189.10(151.60; 227.04) | -1.65(-6.88; 3.87) |
| Iceland | female | 275.64(216.14; 339.47) | 733.31(601.43; 849.86) | 2.58(-4.25; 9.89) | 206.02(161.27; 253.23) | 407.12(330.73; 475.36) | 1.62(-4.57; 8.21) |
| Algeria | female | 10,297.64(7,713.04; 13,721.58) | 27,215.06(19,868.10; 34,717.17) | 4.02(-6.48; 15.70) | 94.15(71.04; 122.24) | 122.72(89.18; 156.63) | 1.57(-3.40; 6.78) |
| Czechia | female | 4,015.13(2,772.45; 5,563.10) | 4,946.27(3,798.14; 6,088.16) | 1.29(-7.31; 10.69) | 79.57(54.15; 112.01) | 98.65(74.39; 124.74) | 1.52(-3.23; 6.50) |
| Malta | female | 208.11(143.83; 284.14) | 251.10(188.90; 311.26) | 1.92(-3.82; 8.01) | 110.32(76.16; 152.08) | 117.74(87.91; 147.99) | 1.50(-3.47; 6.72) |
| Dominican Republic | female | 1,670.16(1,120.54; 2,304.81) | 4,025.38(2,744.42; 5,393.19) | 2.92(-5.60; 12.21) | 44.98(31.10; 61.07) | 68.50(46.72; 91.86) | 1.49(-2.88; 6.06) |
| Latvia | female | 3,591.17(2,573.60; 4,807.10) | 1,311.93(996.68; 1,622.46) | -2.79(-9.79; 4.76) | 252.66(179.02; 340.72) | 137.84(103.59; 172.41) | -1.47(-6.38; 3.70) |
| Bahrain | female | 206.90(136.05; 304.84) | 678.32(444.88; 923.25) | 5.59(-1.36; 13.03) | 88.94(60.11; 125.97) | 105.24(69.11; 143.42) | 1.47(-3.34; 6.53) |
| Guatemala | female | 2,678.78(1,928.69; 3,607.57) | 7,907.70(6,062.94; 9,824.00) | 5.09(-4.29; 15.39) | 77.16(56.18; 101.71) | 89.40(68.97; 111.18) | 1.45(-3.20; 6.33) |
| Myanmar | female | 17,245.55(12,038.51; 23,316.18) | 14,292.05(9,796.38; 19,653.37) | -0.15(-9.62; 10.32) | 84.92(59.81; 112.62) | 46.96(32.22; 64.57) | -1.41(-5.30; 2.63) |
| Sao Tome and Principe | female | 33.13(21.62; 47.48) | 44.15(28.44; 61.47) | 1.24(-2.67; 5.30) | 71.10(46.13; 100.67) | 41.94(27.39; 57.70) | -1.40(-5.17; 2.52) |
| Sudan | female | 6,693.34(4,995.39; 8,809.01) | 26,569.26(19,580.12; 34,161.12) | 4.26(-6.24; 15.92) | 77.83(59.17; 100.69) | 124.66(93.28; 157.71) | 1.40(-3.58; 6.63) |
| Italy | female | 43,470.19(32,007.65; 56,498.25) | 26,436.55(19,405.02; 33,183.95) | -1.66(-11.56; 9.34) | 145.60(107.04; 190.73) | 96.23(69.05; 122.13) | -1.39(-5.98; 3.41) |
| Austria | female | 5,283.15(3,942.10; 6,809.42) | 6,712.24(5,309.11; 8,082.59) | 1.56(-7.35; 11.33) | 126.68(94.47; 163.17) | 153.78(120.72; 187.02) | 1.39(-3.81; 6.86) |
| Micronesia (Federated States of) | female | 60.02(42.55; 81.59) | 32.15(22.05; 42.94) | -0.92(-4.46; 2.74) | 138.80(99.88; 187.79) | 61.63(42.68; 81.50) | -1.32(-5.48; 3.02) |
| Cuba | female | 6,756.19(4,502.38; 9,283.94) | 4,133.19(2,850.74; 5,518.93) | -1.65(-9.83; 7.27) | 111.18(74.86; 153.03) | 75.98(52.90; 101.83) | -1.31(-5.67; 3.25) |
| Pakistan | female | 69,488.78(45,321.01; 102,106.09) | 80,376.13(58,964.80; 105,578.81) | 1.71(-9.58; 14.40) | 155.87(103.19; 225.34) | 72.17(53.83; 93.29) | -1.29(-5.60; 3.22) |
| Oman | female | 498.21(327.84; 719.08) | 1,984.83(1,297.43; 2,727.55) | 5.24(-2.76; 13.90) | 77.42(51.98; 107.80) | 100.00(65.89; 136.00) | 1.27(-3.49; 6.25) |
| Spain | female | 32,091.02(23,432.02; 42,022.62) | 31,636.14(24,585.14; 39,208.29) | -0.98(-11.13; 10.33) | 159.54(116.82; 209.65) | 144.23(110.08; 183.36) | -1.24(-6.26; 4.04) |
| Brunei Darussalam | female | 145.22(112.88; 186.57) | 227.27(162.68; 293.08) | 1.10(-4.46; 6.97) | 120.03(95.53; 151.38) | 91.38(65.19; 117.04) | -1.21(-5.75; 3.56) |
| Zambia | female | 2,716.83(1,768.88; 3,815.55) | 3,959.43(2,834.40; 5,226.43) | 2.09(-6.34; 11.29) | 81.36(54.07; 112.74) | 44.63(32.16; 58.06) | -1.21(-5.05; 2.79) |
| Democratic People's Republic of Korea | female | 20,183.23(14,647.59; 26,782.14) | 13,812.67(10,220.53; 17,413.62) | -0.55(-9.96; 9.84) | 179.42(130.87; 235.88) | 97.04(71.37; 123.70) | -1.20(-5.80; 3.63) |
| Lebanon | female | 2,074.26(1,539.79; 2,692.70) | 3,473.88(2,509.83; 4,536.55) | 3.69(-4.78; 12.90) | 140.25(104.81; 181.96) | 114.99(82.57; 149.98) | 1.18(-3.71; 6.33) |
| Djibouti | female | 63.07(41.61; 91.23) | 242.90(171.85; 326.92) | 5.45(-0.41; 11.66) | 32.13(22.07; 45.59) | 40.02(28.60; 53.75) | 1.17(-2.65; 5.14) |
| Nicaragua | female | 1,583.01(1,057.47; 2,199.52) | 2,428.54(1,628.18; 3,285.75) | 1.31(-6.59; 9.88) | 95.97(65.38; 131.35) | 66.67(44.79; 89.59) | -1.15(-5.38; 3.28) |
| Portugal | female | 4,110.87(2,838.46; 5,718.70) | 5,184.18(3,978.20; 6,370.06) | 1.00(-7.61; 10.42) | 78.48(54.03; 110.36) | 97.81(74.30; 122.44) | 1.13(-3.59; 6.07) |
| Croatia | female | 2,215.74(1,551.97; 2,950.82) | 2,065.92(1,584.03; 2,549.05) | 0.50(-7.20; 8.84) | 87.13(60.51; 116.61) | 94.39(70.34; 118.76) | 1.13(-3.58; 6.07) |
| North Macedonia | female | 491.87(332.73; 682.60) | 638.81(466.54; 813.20) | 1.44(-5.22; 8.57) | 47.28(32.01; 65.64) | 56.17(40.33; 72.67) | 1.13(-3.08; 5.54) |
| Greece | female | 3,837.59(2,880.93; 4,919.78) | 5,712.51(4,542.49; 6,903.85) | 0.64(-8.07; 10.17) | 73.20(54.97; 94.52) | 118.15(92.13; 144.91) | 1.11(-3.82; 6.29) |
| Andorra | female | 23.82(16.16; 32.72) | 32.54(22.51; 42.48) | 1.63(-2.00; 5.39) | 79.30(54.28; 108.24) | 79.65(55.18; 105.47) | 1.09(-3.41; 5.81) |
| Togo | female | 509.65(330.90; 727.10) | 1,514.12(1,011.38; 2,116.30) | 4.27(-3.38; 12.53) | 33.04(22.08; 45.99) | 36.98(24.99; 50.79) | 1.08(-2.65; 4.96) |
| Jordan | female | 1,440.39(966.74; 2,039.64) | 6,327.04(4,212.17; 8,663.28) | 5.50(-3.69; 15.56) | 90.75(61.36; 125.61) | 103.95(70.14; 141.61) | 1.01(-3.77; 6.03) |
| Bolivia (Plurinational State of) | female | 4,782.58(3,272.40; 6,560.36) | 5,835.66(4,312.94; 7,509.08) | 1.46(-7.31; 11.05) | 160.66(111.25; 217.16) | 93.48(69.12; 120.61) | -1.00(-5.58; 3.80) |
| Belgium | female | 6,823.02(4,973.28; 8,910.82) | 8,633.43(7,079.45; 10,197.11) | 1.37(-7.74; 11.38) | 129.58(93.44; 171.41) | 145.39(117.94; 172.62) | 0.98(-4.10; 6.33) |
| Afghanistan | female | 10,708.49(7,741.92; 14,563.33) | 30,635.65(23,719.53; 37,928.35) | 4.34(-6.30; 16.18) | 228.08(166.36; 305.47) | 231.26(181.85; 282.43) | 0.97(-4.60; 6.87) |
| Kuwait | female | 842.25(545.52; 1,228.69) | 2,847.09(1,883.10; 3,852.08) | 5.83(-2.61; 15.00) | 99.81(65.31; 142.60) | 102.36(66.19; 140.29) | 0.96(-3.81; 5.96) |
| Comoros | female | 62.46(41.90; 88.61) | 154.69(109.10; 206.98) | 2.95(-2.31; 8.50) | 31.32(21.19; 43.48) | 41.06(29.36; 54.17) | 0.95(-2.88; 4.94) |
| Gabon | female | 331.96(226.38; 468.05) | 428.80(295.83; 579.60) | 1.71(-4.51; 8.33) | 78.80(53.90; 109.28) | 45.47(31.40; 60.47) | -0.94(-4.81; 3.09) |
| Fiji | female | 367.96(256.55; 498.32) | 248.70(176.61; 334.66) | -0.22(-5.80; 5.69) | 97.58(69.25; 129.43) | 54.20(38.48; 72.90) | -0.93(-4.98; 3.28) |
| Egypt | female | 19,825.22(12,614.06; 29,122.45) | 51,464.49(34,641.53; 69,921.91) | 3.31(-7.73; 15.67) | 78.40(50.78; 112.49) | 101.54(69.46; 136.10) | 0.93(-3.82; 5.91) |
| Saudi Arabia | female | 6,196.30(4,254.94; 8,587.14) | 20,860.21(13,893.74; 29,233.69) | 5.14(-5.21; 16.62) | 103.92(73.62; 138.84) | 105.29(70.02; 145.12) | 0.92(-3.86; 5.95) |
| Bangladesh | female | 30,060.21(20,667.67; 40,896.69) | 48,624.78(35,720.38; 64,351.69) | 3.25(-7.73; 15.53) | 68.82(48.50; 91.20) | 54.66(40.28; 71.98) | 0.89(-3.24; 5.20) |
| Guam | female | 83.67(56.87; 114.98) | 45.57(30.74; 60.95) | -0.56(-4.46; 3.50) | 121.92(84.30; 164.57) | 58.77(39.44; 78.90) | -0.88(-5.01; 3.44) |
| Senegal | female | 1,904.21(1,240.06; 2,731.65) | 3,006.11(1,950.17; 4,198.71) | 1.85(-6.29; 10.71) | 62.03(40.91; 86.30) | 40.55(27.09; 55.44) | -0.88(-4.64; 3.02) |
| Chile | female | 6,359.45(4,249.20; 8,856.81) | 10,132.95(7,337.84; 13,127.27) | 1.91(-7.43; 12.18) | 86.96(58.79; 120.58) | 101.75(74.11; 132.09) | 0.86(-3.88; 5.84) |
| Solomon Islands | female | 155.58(110.99; 209.86) | 192.19(132.57; 257.87) | 1.77(-3.65; 7.50) | 109.53(79.70; 144.43) | 58.06(40.50; 76.64) | -0.84(-4.95; 3.46) |
| Turkey | female | 30,595.84(20,346.36; 43,766.57) | 46,734.58(32,064.72; 63,794.38) | 2.25(-8.59; 14.38) | 106.19(71.58; 149.30) | 106.99(72.66; 147.39) | 0.84(-3.96; 5.88) |
| Republic of Moldova | female | 3,109.78(2,245.20; 4,101.74) | 2,118.60(1,585.01; 2,665.37) | -1.27(-8.85; 6.95) | 131.83(95.10; 173.51) | 107.19(78.42; 137.62) | -0.83(-5.54; 4.13) |
| Benin | female | 768.19(512.02; 1,084.69) | 2,419.60(1,564.77; 3,295.50) | 4.33(-3.79; 13.14) | 37.70(25.38; 51.96) | 39.72(25.98; 53.79) | 0.83(-2.96; 4.78) |
| Paraguay | female | 3,231.91(1,934.49; 4,818.66) | 2,966.36(2,078.11; 4,002.16) | 1.57(-6.55; 10.39) | 169.26(103.19; 249.29) | 77.21(54.33; 103.62) | -0.82(-5.22; 3.79) |
| New Zealand | female | 3,058.99(2,204.86; 4,107.10) | 5,313.95(4,266.90; 6,358.53) | 1.61(-7.06; 11.08) | 167.46(120.64; 224.97) | 206.70(164.38; 248.73) | 0.81(-4.61; 6.55) |
| Namibia | female | 1,098.95(724.53; 1,568.55) | 1,145.64(815.96; 1,502.79) | 1.34(-5.83; 9.07) | 171.26(115.97; 237.94) | 92.10(65.95; 119.43) | -0.80(-5.37; 4.00) |
| Ethiopia | female | 9,095.83(6,245.73; 12,459.59) | 17,831.79(13,035.16; 23,383.57) | 2.06(-7.82; 13.01) | 43.60(30.56; 58.35) | 35.67(26.02; 46.05) | -0.80(-4.43; 2.97) |
| United Arab Emirates | female | 1,030.47(711.22; 1,429.44) | 3,586.83(2,522.31; 4,708.37) | 6.90(-1.88; 16.45) | 164.90(118.29; 217.90) | 125.00(89.72; 162.29) | 0.78(-4.18; 6.00) |
| Sierra Leone | female | 1,127.88(737.05; 1,590.38) | 1,798.29(1,200.43; 2,457.29) | 2.68(-5.02; 11.00) | 70.58(46.02; 98.61) | 42.00(28.43; 56.35) | -0.77(-4.56; 3.17) |
| Nauru | female | 5.31(3.77; 7.09) | 3.40(2.36; 4.53) | -0.33(-1.62; 0.98) | 117.17(85.15; 155.11) | 61.57(42.60; 81.26) | -0.76(-4.95; 3.60) |
| Puerto Rico | female | 2,314.18(1,698.36; 2,991.81) | 1,771.02(1,375.09; 2,221.91) | -1.03(-8.49; 7.04) | 120.90(88.69; 156.09) | 105.40(81.49; 132.22) | -0.75(-5.49; 4.23) |
| Japan | female | 49,984.43(36,189.59; 66,449.09) | 46,419.51(34,879.57; 59,000.75) | 0.20(-10.42; 12.07) | 77.03(55.61; 103.21) | 83.01(60.76; 106.72) | 0.75(-3.78; 5.50) |
| Burkina Faso | female | 1,244.35(821.04; 1,756.49) | 3,721.99(2,453.09; 5,135.20) | 4.11(-4.43; 13.41) | 31.67(21.10; 44.15) | 36.25(23.96; 49.09) | 0.75(-2.96; 4.59) |
| Israel | female | 2,626.23(1,918.99; 3,416.16) | 4,734.29(3,624.92; 5,862.03) | 2.71(-5.96; 12.17) | 107.13(78.17; 139.37) | 102.73(78.43; 127.72) | 0.73(-4.02; 5.72) |
| Sri Lanka | female | 9,512.62(6,513.85; 13,015.04) | 7,023.64(5,075.52; 9,002.05) | 0.13(-8.70; 9.81) | 106.69(74.23; 143.70) | 60.12(42.86; 77.37) | -0.69(-4.85; 3.65) |
| Niue | female | 1.16(0.82; 1.54) | 0.48(0.33; 0.62) | -1.68(-2.50; -0.84) | 114.90(80.44; 152.76) | 59.22(40.19; 78.04) | -0.69(-4.83; 3.64) |
| Tuvalu | female | 5.08(3.65; 6.74) | 3.46(2.34; 4.77) | 0.04(-1.26; 1.36) | 105.06(75.74; 138.98) | 57.89(39.37; 79.44) | -0.69(-4.81; 3.61) |
| Armenia | female | 1,358.56(897.37; 1,888.74) | 1,791.71(1,241.96; 2,344.35) | 0.51(-7.06; 8.69) | 73.93(49.92; 101.57) | 112.21(75.64; 149.86) | 0.69(-4.15; 5.78) |
| Panama | female | 825.34(556.20; 1,147.90) | 1,722.72(1,247.16; 2,270.61) | 2.63(-5.03; 10.90) | 69.62(47.40; 96.38) | 79.19(57.32; 104.52) | 0.69(-3.79; 5.39) |
| Costa Rica | female | 1,195.37(807.34; 1,666.86) | 2,096.29(1,490.76; 2,725.30) | 2.65(-5.21; 11.17) | 79.99(55.24; 109.97) | 78.14(55.53; 102.93) | 0.69(-3.78; 5.37) |
| Equatorial Guinea | female | 99.87(67.44; 137.38) | 319.39(218.70; 427.69) | 5.08(-1.04; 11.57) | 53.82(36.71; 73.32) | 46.57(32.45; 61.50) | 0.69(-3.26; 4.81) |
| Russian Federation | female | 176,281.92(136,349.30; 220,329.19) | 170,328.92(136,674.85; 202,496.85) | -0.77(-12.50; 12.52) | 217.42(166.78; 273.03) | 228.32(180.73; 274.23) | -0.68(-6.17; 5.14) |
| Norway | female | 3,537.47(2,781.58; 4,386.06) | 8,908.46(7,156.71; 10,579.66) | 1.50(-7.69; 11.60) | 162.29(127.97; 201.20) | 317.59(254.37; 378.45) | 0.68(-5.22; 6.94) |
| Romania | female | 8,150.10(6,284.53; 10,279.14) | 5,483.84(4,335.59; 6,683.56) | -1.40(-9.85; 7.84) | 67.56(51.84; 85.80) | 54.76(42.53; 67.52) | -0.68(-4.72; 3.53) |
| Bhutan | female | 166.92(110.16; 242.14) | 213.02(152.80; 285.55) | 2.63(-2.96; 8.54) | 63.36(43.65; 88.28) | 53.41(38.53; 71.04) | 0.68(-3.41; 4.96) |
| Gambia | female | 247.61(161.63; 352.44) | 461.45(312.94; 637.76) | 2.49(-3.84; 9.24) | 61.13(40.92; 84.16) | 40.31(27.15; 55.74) | -0.67(-4.43; 3.23) |
| Suriname | female | 123.17(84.45; 170.22) | 223.49(161.77; 298.24) | 2.33(-3.29; 8.27) | 63.72(44.30; 87.36) | 74.81(54.07; 99.65) | 0.67(-3.76; 5.31) |
| Denmark | female | 4,526.00(3,682.76; 5,505.38) | 7,033.70(5,759.35; 8,291.19) | 0.86(-8.03; 10.62) | 165.38(133.53; 203.11) | 228.85(185.33; 273.84) | 0.66(-4.88; 6.53) |
| Indonesia | female | 63,409.37(43,510.26; 87,834.99) | 55,042.44(38,774.22; 75,619.91) | 0.59(-10.22; 12.71) | 67.44(47.33; 92.49) | 37.54(26.61; 51.41) | -0.66(-4.35; 3.18) |
| Hungary | female | 4,012.60(2,751.48; 5,585.98) | 3,079.81(2,209.40; 4,028.25) | 0.19(-7.86; 8.95) | 78.77(53.04; 111.68) | 67.10(47.37; 89.73) | 0.66(-3.67; 5.17) |
| India | female | 445,783.90(306,602.71; 628,024.80) | 480,819.54(361,894.31; 607,832.29) | 1.52(-11.41; 16.34) | 115.43(81.09; 157.32) | 65.22(49.63; 82.01) | -0.65(-4.88; 3.78) |
| Cook Islands | female | 9.94(6.79; 13.93) | 5.25(3.60; 6.91) | -0.68(-2.41; 1.07) | 111.18(76.73; 153.31) | 57.08(38.70; 76.89) | -0.65(-4.76; 3.64) |
| Marshall Islands | female | 20.52(14.36; 27.22) | 17.60(12.38; 23.74) | 0.70(-2.28; 3.77) | 112.32(80.25; 145.10) | 61.93(43.80; 83.07) | -0.64(-4.83; 3.73) |
| Germany | female | 49,882.34(39,415.52; 62,321.98) | 54,845.46(43,851.06; 64,995.49) | 0.56(-10.24; 12.65) | 119.10(93.68; 150.20) | 133.74(105.04; 161.82) | 0.64(-4.35; 5.89) |
| Bulgaria | female | 3,100.27(2,172.12; 4,186.80) | 2,231.19(1,686.81; 2,804.63) | -0.39(-8.09; 7.95) | 72.96(50.25; 99.96) | 70.92(52.03; 90.67) | 0.64(-3.73; 5.21) |
| Albania | female | 812.73(536.60; 1,165.02) | 869.01(668.99; 1,090.55) | -0.09(-6.91; 7.22) | 47.34(32.07; 66.66) | 61.14(46.58; 77.78) | 0.64(-3.59; 5.06) |
| Palau | female | 8.59(5.76; 11.88) | 4.64(3.21; 6.11) | -1.12(-2.76; 0.55) | 108.16(73.05; 146.85) | 60.45(41.50; 80.44) | -0.63(-4.79; 3.72) |
| Barbados | female | 165.66(110.92; 228.67) | 132.34(94.56; 172.68) | -0.21(-5.17; 5.00) | 117.83(79.20; 161.97) | 87.76(62.73; 115.62) | -0.62(-5.15; 4.12) |
| Monaco | female | 15.46(10.64; 20.83) | 14.99(10.62; 19.70) | 1.10(-1.72; 4.00) | 104.84(70.96; 143.00) | 89.82(63.22; 118.36) | 0.62(-3.99; 5.45) |
| San Marino | female | 13.04(8.79; 17.77) | 13.47(9.61; 17.48) | 1.42(-1.30; 4.23) | 104.34(70.74; 142.87) | 86.46(61.15; 114.06) | 0.62(-3.95; 5.42) |
| Viet Nam | female | 34,625.68(24,067.47; 47,032.27) | 33,537.21(24,907.94; 42,652.08) | 0.94(-9.45; 12.53) | 100.82(71.45; 136.32) | 64.20(47.22; 82.25) | -0.59(-4.81; 3.83) |
| Venezuela (Bolivarian Republic of) | female | 8,940.79(5,948.54; 12,906.03) | 9,799.56(6,727.96; 12,977.40) | 1.14(-8.12; 11.33) | 98.53(66.56; 137.97) | 70.99(49.10; 94.28) | -0.58(-4.90; 3.95) |
| Philippines | female | 23,213.75(16,380.28; 31,242.30) | 26,841.03(18,416.58; 35,780.02) | 1.58(-8.66; 12.97) | 77.48(55.60; 102.97) | 45.68(31.49; 60.49) | -0.58(-4.47; 3.46) |
| Tokelau | female | 0.77(0.55; 1.02) | 0.38(0.26; 0.51) | -1.17(-2.22; -0.10) | 108.00(78.51; 144.00) | 58.62(39.90; 78.41) | -0.57(-4.70; 3.75) |
| Kazakhstan | female | 12,000.36(8,839.91; 15,761.94) | 17,961.57(13,730.53; 22,180.47) | 1.32(-8.51; 12.21) | 141.02(104.12; 183.85) | 183.43(139.74; 226.70) | 0.56(-4.77; 6.19) |
| Seychelles | female | 46.87(33.85; 62.67) | 34.73(26.45; 44.25) | 0.37(-3.30; 4.18) | 125.61(91.48; 165.26) | 69.24(52.32; 89.42) | -0.55(-4.87; 3.97) |
| Singapore | female | 1,375.51(920.92; 1,873.51) | 2,576.39(1,767.97; 3,419.38) | 2.26(-5.78; 10.99) | 73.42(49.59; 100.06) | 86.37(60.44; 115.95) | 0.55(-4.01; 5.33) |
| Rwanda | female | 1,076.52(723.69; 1,518.03) | 2,621.87(1,857.94; 3,540.94) | 3.73(-4.44; 12.59) | 34.75(23.56; 48.07) | 39.71(28.64; 52.28) | 0.55(-3.24; 4.49) |
| Mongolia | female | 711.58(506.35; 973.00) | 1,859.12(1,313.93; 2,446.76) | 2.56(-5.19; 10.94) | 70.80(51.22; 95.39) | 110.34(77.77; 144.90) | 0.53(-4.29; 5.59) |
| Azerbaijan | female | 2,483.87(1,698.51; 3,494.75) | 5,705.32(4,043.41; 7,478.82) | 1.96(-6.83; 11.59) | 61.91(42.54; 85.70) | 98.54(70.62; 129.86) | 0.53(-4.17; 5.47) |
| Niger | female | 1,331.34(864.59; 1,861.74) | 3,869.45(2,558.58; 5,372.23) | 4.11(-4.46; 13.45) | 43.09(28.62; 58.75) | 40.03(26.59; 54.67) | 0.53(-3.26; 4.48) |
| Slovakia | female | 2,748.38(2,051.21; 3,552.17) | 1,968.06(1,437.22; 2,491.86) | -0.54(-8.11; 7.66) | 101.94(75.32; 132.55) | 73.02(52.72; 93.76) | -0.52(-4.88; 4.04) |
| Vanuatu | female | 55.24(37.36; 77.97) | 82.01(55.06; 111.28) | 2.21(-2.37; 7.00) | 82.19(57.83; 111.37) | 53.03(36.41; 71.12) | -0.52(-4.56; 3.68) |
| Kenya | female | 4,971.26(3,317.71; 7,001.66) | 7,502.77(5,451.49; 9,534.71) | 2.72(-6.39; 12.72) | 54.53(36.43; 74.71) | 31.67(23.35; 39.34) | -0.52(-4.05; 3.13) |
| Democratic Republic of the Congo | female | 6,829.35(4,620.67; 9,312.85) | 16,753.19(11,589.74; 22,316.37) | 3.60(-6.37; 14.63) | 42.38(28.98; 57.17) | 41.72(28.90; 54.88) | 0.52(-3.31; 4.51) |
| Eswatini | female | 531.06(356.61; 776.43) | 556.37(406.02; 730.25) | 0.99(-5.46; 7.87) | 146.25(100.76; 206.85) | 96.02(70.92; 123.01) | -0.50(-5.13; 4.36) |
| Taiwan (Province of China) | female | 20,187.98(14,830.58; 26,811.08) | 10,438.88(7,836.16; 13,348.52) | -0.08(-9.26; 10.04) | 182.67(135.39; 239.02) | 85.96(63.02; 110.86) | -0.49(-5.00; 4.25) |
| Samoa | female | 67.61(47.04; 92.14) | 59.16(40.65; 78.80) | 0.50(-3.68; 4.86) | 94.66(67.07; 127.22) | 60.30(41.67; 79.78) | -0.48(-4.65; 3.87) |
| Mozambique | female | 2,204.06(1,491.20; 3,078.75) | 5,289.33(3,769.55; 7,199.20) | 3.21(-5.60; 12.84) | 38.45(26.20; 52.81) | 38.26(27.57; 50.90) | 0.47(-3.28; 4.37) |
| Tajikistan | female | 1,478.04(1,020.67; 2,036.29) | 4,995.15(3,425.89; 6,629.28) | 3.08(-5.68; 12.64) | 60.72(43.07; 81.69) | 97.06(66.79; 127.65) | 0.44(-4.25; 5.35) |
| Central African Republic | female | 635.82(425.09; 884.95) | 1,062.15(719.06; 1,440.56) | 2.00(-5.14; 9.67) | 52.59(35.75; 72.19) | 40.88(28.33; 55.16) | -0.44(-4.21; 3.49) |
| Serbia | female | 2,539.30(1,830.94; 3,364.46) | 2,277.79(1,730.27; 2,825.35) | 0.07(-7.68; 8.46) | 54.33(39.27; 72.66) | 48.99(36.69; 62.30) | 0.43(-3.56; 4.59) |
| Nepal | female | 7,592.92(4,880.31; 11,290.86) | 9,584.82(6,768.27; 12,892.49) | 1.92(-7.36; 12.13) | 85.57(55.51; 124.80) | 55.06(39.40; 72.63) | -0.41(-4.49; 3.84) |
| Lithuania | female | 2,359.06(1,809.67; 2,997.64) | 1,876.01(1,448.65; 2,291.03) | -0.76(-8.26; 7.34) | 118.29(90.27; 150.83) | 128.37(98.07; 158.74) | 0.40(-4.54; 5.60) |
| Northern Mariana Islands | female | 30.77(20.38; 43.74) | 13.31(9.33; 17.51) | -2.09(-4.93; 0.84) | 115.69(77.94; 159.92) | 58.96(41.52; 78.22) | -0.39(-4.55; 3.95) |
| Republic of Korea | female | 18,930.86(12,856.09; 26,357.27) | 23,784.91(16,943.68; 30,939.31) | 0.45(-9.56; 11.57) | 74.32(51.17; 102.41) | 93.02(65.97; 122.44) | 0.39(-4.24; 5.24) |
| Malawi | female | 2,075.75(1,448.64; 2,838.07) | 3,658.37(2,634.43; 4,888.74) | 2.24(-6.13; 11.35) | 50.81(35.98; 68.76) | 40.86(30.10; 53.33) | -0.39(-4.17; 3.54) |
| Lao People's Democratic Republic | female | 1,353.68(941.32; 1,851.65) | 2,097.49(1,363.15; 2,949.23) | 2.08(-5.74; 10.55) | 70.86(49.69; 96.55) | 52.60(34.54; 72.50) | -0.38(-4.42; 3.82) |
| Dominica | female | 29.17(21.04; 37.89) | 31.38(24.19; 39.64) | 0.35(-3.20; 4.03) | 81.50(58.90; 105.70) | 91.34(70.32; 115.99) | 0.37(-4.25; 5.22) |
| Argentina | female | 14,146.32(9,424.17; 20,073.12) | 21,730.72(14,933.40; 29,152.33) | 1.72(-8.33; 12.87) | 86.36(57.40; 122.44) | 88.97(60.95; 119.58) | 0.37(-4.21; 5.18) |
| Palestine | female | 823.26(538.23; 1,196.90) | 2,567.03(1,621.91; 3,653.77) | 4.16(-4.02; 13.03) | 97.05(64.77; 135.69) | 97.27(61.75; 135.08) | 0.36(-4.32; 5.27) |
| Saint Kitts and Nevis | female | 19.23(13.86; 25.26) | 26.79(19.13; 34.46) | 1.33(-2.09; 4.86) | 93.97(68.53; 123.28) | 82.92(59.12; 107.22) | -0.35(-4.84; 4.35) |
| Morocco | female | 17,013.67(11,536.49; 23,807.39) | 25,973.05(19,263.55; 33,069.91) | 1.90(-8.34; 13.28) | 140.60(97.90; 191.67) | 133.58(99.25; 170.02) | 0.35(-4.64; 5.61) |
| American Samoa | female | 23.81(16.37; 32.60) | 13.75(9.38; 18.21) | -0.38(-3.10; 2.41) | 101.17(70.00; 136.91) | 59.50(41.11; 79.47) | -0.35(-4.51; 4.00) |
| Cambodia | female | 3,280.51(2,230.34; 4,580.97) | 4,254.34(2,877.12; 5,803.70) | 1.85(-6.65; 11.12) | 66.32(46.68; 90.77) | 46.38(31.61; 62.61) | -0.35(-4.26; 3.72) |
| Madagascar | female | 1,984.38(1,333.87; 2,830.29) | 5,538.71(3,951.46; 7,197.88) | 3.49(-5.38; 13.20) | 39.02(26.27; 54.56) | 41.76(30.64; 53.62) | 0.34(-3.49; 4.33) |
| Netherlands | female | 7,870.57(5,472.01; 10,484.03) | 8,772.94(6,703.93; 10,958.08) | 0.43(-8.63; 10.39) | 98.36(67.94; 131.70) | 104.81(79.66; 131.00) | 0.33(-4.42; 5.31) |
| El Salvador | female | 1,740.34(1,183.85; 2,449.50) | 2,543.26(1,753.68; 3,349.36) | 1.52(-6.44; 10.15) | 70.93(49.04; 97.34) | 69.96(48.70; 91.94) | 0.33(-4.01; 4.86) |
| United Republic of Tanzania | female | 5,673.08(3,736.42; 8,116.61) | 13,433.13(9,376.22; 18,179.52) | 3.17(-6.51; 13.84) | 49.94(33.14; 69.70) | 49.07(34.88; 65.41) | 0.33(-3.62; 4.44) |
| Uruguay | female | 2,578.24(1,680.03; 3,672.25) | 1,906.91(1,385.45; 2,442.35) | 0.06(-7.51; 8.25) | 164.99(106.72; 235.17) | 107.06(76.66; 138.04) | -0.32(-5.07; 4.65) |
| Tonga | female | 44.05(29.96; 60.23) | 30.41(21.08; 41.23) | 0.14(-3.37; 3.78) | 102.35(69.79; 138.29) | 60.00(41.63; 80.72) | -0.32(-4.50; 4.03) |
| Guinea-Bissau | female | 156.38(106.24; 218.88) | 351.32(235.86; 476.13) | 3.03(-3.07; 9.51) | 36.27(24.98; 50.11) | 35.73(23.62; 47.75) | 0.32(-3.35; 4.14) |
| Trinidad and Tobago | female | 477.70(326.25; 659.52) | 557.77(411.34; 726.58) | 0.91(-5.53; 7.80) | 79.00(54.50; 107.98) | 78.99(58.00; 103.29) | 0.29(-4.18; 4.96) |
| Georgia | female | 2,520.33(1,766.25; 3,434.94) | 1,985.85(1,351.71; 2,606.69) | -1.33(-8.85; 6.81) | 83.11(58.07; 114.03) | 111.81(76.37; 147.89) | 0.27(-4.55; 5.33) |
| Guyana | female | 254.42(175.10; 347.97) | 302.67(220.82; 395.39) | 0.35(-5.46; 6.51) | 64.41(44.73; 87.38) | 72.95(53.45; 95.23) | 0.27(-4.12; 4.85) |
| Côte d'Ivoire | female | 2,172.75(1,440.12; 3,078.22) | 4,873.48(3,196.80; 6,756.92) | 3.13(-5.59; 12.67) | 43.95(29.83; 60.76) | 38.82(25.83; 53.64) | 0.27(-3.48; 4.17) |
| Australia | female | 27,169.68(20,873.52; 34,375.50) | 44,680.98(36,527.21; 52,497.60) | 1.52(-9.20; 13.50) | 303.98(233.28; 384.62) | 334.35(269.92; 394.18) | 0.26(-5.64; 6.53) |
| Cyprus | female | 298.07(221.02; 392.32) | 577.85(424.82; 734.50) | 2.52(-4.06; 9.54) | 74.62(55.30; 98.03) | 77.03(55.85; 98.03) | 0.26(-4.19; 4.92) |
| Haiti | female | 2,078.03(1,481.91; 2,753.74) | 5,253.35(3,891.87; 6,807.28) | 3.08(-5.71; 12.69) | 68.67(49.99; 90.00) | 75.39(55.99; 97.57) | 0.26(-4.15; 4.87) |
| Luxembourg | female | 316.92(241.60; 406.97) | 655.39(520.22; 795.60) | 1.81(-4.83; 8.91) | 151.71(114.60; 196.16) | 191.99(152.41; 233.73) | 0.25(-5.10; 5.89) |
| Brazil | female | 94,722.69(65,819.42; 129,362.53) | 125,915.92(91,725.94; 159,619.73) | 1.73(-9.99; 14.97) | 123.07(86.03; 167.11) | 105.59(77.05; 134.05) | 0.24(-4.51; 5.23) |
| Lesotho | female | 966.04(648.42; 1,351.55) | 947.71(701.18; 1,211.19) | 0.95(-6.01; 8.43) | 118.69(79.44; 164.32) | 101.40(75.92; 128.26) | 0.24(-4.48; 5.18) |
| Maldives | female | 66.25(44.34; 92.27) | 106.34(71.95; 141.84) | 2.95(-1.95; 8.09) | 70.44(49.45; 95.62) | 47.47(31.90; 63.23) | -0.24(-4.18; 3.87) |
| Ecuador | female | 4,509.48(3,110.02; 6,187.63) | 7,834.14(5,555.46; 10,180.49) | 2.38(-6.75; 12.40) | 92.73(65.07; 125.39) | 82.08(58.13; 106.53) | 0.22(-4.28; 4.93) |
| Kiribati | female | 37.75(27.39; 49.98) | 83.49(64.85; 106.34) | 2.18(-2.42; 6.99) | 102.02(75.02; 132.30) | 133.46(103.43; 168.80) | 0.21(-4.77; 5.45) |
| Bahamas | female | 144.42(100.24; 200.32) | 182.13(126.76; 241.84) | 1.15(-4.19; 6.78) | 101.83(71.44; 139.12) | 83.69(58.08; 111.16) | -0.21(-4.72; 4.50) |
| Colombia | female | 18,584.62(13,176.63; 25,091.69) | 23,843.46(17,163.18; 31,042.12) | 1.21(-8.88; 12.41) | 109.12(78.91; 145.00) | 88.03(63.08; 114.59) | -0.20(-4.75; 4.57) |
| Malaysia | female | 8,025.23(5,376.58; 11,311.52) | 8,617.90(5,732.96; 11,795.83) | 2.06(-7.14; 12.17) | 92.58(63.38; 128.05) | 50.17(33.52; 68.20) | -0.18(-4.18; 4.00) |
| Ukraine | female | 39,717.05(30,141.99; 51,238.92) | 29,295.02(21,832.51; 37,125.30) | -0.87(-10.96; 10.36) | 135.90(101.96; 177.44) | 127.18(93.01; 160.86) | -0.16(-5.08; 5.02) |
| Bermuda | female | 44.99(31.57; 60.65) | 29.57(21.95; 37.87) | -0.45(-3.92; 3.15) | 128.39(89.97; 173.06) | 97.30(71.13; 127.01) | -0.16(-4.81; 4.73) |
| Botswana | female | 843.63(548.24; 1,220.92) | 1,115.56(799.27; 1,467.03) | 2.66(-4.59; 10.45) | 137.73(90.22; 193.81) | 86.75(62.29; 113.22) | 0.16(-4.41; 4.94) |
| Guinea | female | 919.64(610.02; 1,278.42) | 2,168.87(1,454.82; 2,982.50) | 2.79(-5.10; 11.34) | 34.61(23.58; 47.43) | 34.89(23.19; 47.16) | 0.16(-3.48; 3.93) |
| Kyrgyzstan | female | 2,034.19(1,527.64; 2,635.49) | 4,391.72(3,206.17; 5,602.80) | 1.58(-6.93; 10.85) | 95.70(72.97; 122.38) | 126.01(92.31; 159.94) | -0.15(-5.07; 5.03) |
| Timor-Leste | female | 270.01(179.64; 378.51) | 340.06(229.18; 476.06) | 1.87(-4.13; 8.25) | 73.42(50.30; 101.09) | 48.78(34.11; 66.76) | -0.15(-4.12; 3.99) |
| Iraq | female | 7,429.52(5,418.49; 9,880.91) | 23,210.90(17,350.69; 30,520.38) | 3.29(-6.97; 14.69) | 104.39(76.36; 135.75) | 114.85(86.53; 148.79) | -0.14(-4.96; 4.93) |
| United States Virgin Islands | female | 56.33(41.51; 73.47) | 33.41(24.68; 43.12) | -0.64(-4.25; 3.11) | 102.30(74.83; 132.96) | 86.78(64.10; 113.97) | -0.14(-4.71; 4.64) |
| Nigeria | female | 13,239.87(9,133.73; 17,979.35) | 34,531.60(23,217.35; 45,675.00) | 3.49(-7.17; 15.38) | 36.70(25.61; 49.47) | 32.81(22.12; 42.78) | 0.14(-3.44; 3.85) |
| Mexico | female | 41,442.93(29,250.64; 55,203.54) | 55,693.36(40,074.19; 71,464.11) | 1.44(-9.48; 13.67) | 98.22(70.01; 129.17) | 78.95(56.80; 101.22) | -0.13(-4.58; 4.52) |
| Chad | female | 1,091.58(724.86; 1,521.37) | 2,647.68(1,754.95; 3,601.37) | 3.23(-4.89; 12.06) | 45.03(30.17; 61.51) | 37.76(25.47; 50.45) | -0.13(-3.84; 3.73) |
| Grenada | female | 34.53(24.55; 45.95) | 44.79(33.07; 58.17) | 0.83(-3.13; 4.94) | 86.53(61.72; 114.03) | 83.77(61.81; 108.22) | -0.10(-4.61; 4.63) |
| Antigua and Barbuda | female | 26.29(18.12; 36.10) | 38.67(27.16; 51.29) | 1.43(-2.38; 5.39) | 80.51(55.69; 109.32) | 78.08(55.08; 104.47) | 0.10(-4.35; 4.75) |
| Burundi | female | 973.05(642.97; 1,376.50) | 2,212.49(1,589.45; 2,998.59) | 3.04(-4.89; 11.63) | 40.41(27.19; 56.04) | 38.89(28.60; 52.22) | -0.10(-3.84; 3.79) |
| Uganda | female | 3,803.66(2,529.94; 5,470.43) | 7,987.31(5,567.97; 10,887.24) | 3.12(-6.08; 13.23) | 53.05(36.07; 74.67) | 42.14(29.69; 55.85) | -0.09(-3.91; 3.88) |
| South Sudan | female | 1,031.27(663.97; 1,452.60) | 1,630.52(1,139.47; 2,189.79) | 1.73(-5.80; 9.87) | 43.51(28.75; 60.80) | 39.92(28.29; 53.01) | -0.09(-3.85; 3.82) |
| Somalia | female | 1,048.32(706.06; 1,464.44) | 3,327.29(2,345.88; 4,380.62) | 3.82(-4.57; 12.95) | 37.58(25.48; 51.79) | 38.86(28.43; 50.18) | 0.09(-3.66; 3.98) |
| Iran (Islamic Republic of) | female | 44,990.50(34,881.51; 57,139.27) | 72,226.42(56,232.39; 87,471.72) | 2.30(-8.98; 14.96) | 182.95(145.13; 229.30) | 160.22(124.05; 194.30) | -0.08(-5.24; 5.35) |
| Congo | female | 642.10(442.82; 870.78) | 1,220.37(832.73; 1,600.72) | 3.19(-4.17; 11.12) | 61.45(42.44; 82.50) | 45.38(31.63; 58.92) | 0.08(-3.83; 4.15) |
| Saint Lucia | female | 58.45(40.78; 80.53) | 74.27(51.93; 96.96) | 1.10(-3.37; 5.77) | 84.19(59.50; 114.96) | 78.70(55.56; 103.22) | 0.07(-4.41; 4.76) |
| Ghana | female | 4,806.48(3,083.69; 6,858.20) | 8,582.35(5,718.68; 11,618.26) | 3.27(-6.02; 13.48) | 74.64(48.76; 103.99) | 48.74(32.44; 65.48) | -0.07(-4.04; 4.07) |
| Mauritania | female | 504.90(336.31; 720.29) | 861.08(551.23; 1,195.27) | 2.71(-4.26; 10.20) | 58.86(39.65; 81.95) | 42.80(27.59; 58.44) | 0.06(-3.79; 4.07) |
| Liberia | female | 439.04(289.47; 625.02) | 1,068.87(716.07; 1,481.80) | 3.85(-3.41; 11.66) | 55.39(36.96; 76.74) | 41.63(27.82; 57.32) | 0.06(-3.76; 4.03) |
| Jamaica | female | 1,031.87(714.74; 1,429.90) | 1,292.24(905.27; 1,712.96) | 0.81(-6.44; 8.63) | 84.72(59.01; 116.09) | 81.43(57.06; 107.46) | -0.05(-4.53; 4.64) |
| Cabo Verde | female | 101.83(68.13; 144.52) | 134.18(86.27; 181.59) | 2.29(-2.80; 7.65) | 66.81(45.48; 93.17) | 43.99(28.37; 59.41) | -0.05(-3.92; 3.97) |
| Peru | female | 14,593.82(9,601.84; 20,914.64) | 16,666.88(12,310.86; 21,463.00) | 1.62(-8.16; 12.45) | 131.39(89.46; 186.03) | 85.71(63.39; 109.77) | -0.04(-4.58; 4.71) |
| Saint Vincent and the Grenadines | female | 38.86(26.12; 54.72) | 40.37(28.22; 55.04) | 0.20(-3.59; 4.14) | 73.55(49.97; 102.04) | 71.13(49.77; 97.59) | -0.03(-4.37; 4.51) |
| Mali | female | 1,445.19(986.45; 1,980.81) | 3,741.65(2,402.28; 5,306.47) | 3.47(-5.01; 12.70) | 40.57(27.64; 55.77) | 37.25(24.27; 51.52) | 0.03(-3.67; 3.87) |
| Thailand | female | 24,740.31(17,001.38; 33,770.47) | 22,117.11(15,495.78; 28,654.59) | 0.39(-9.57; 11.44) | 77.88(54.54; 105.25) | 61.54(42.52; 81.20) | -0.02(-4.25; 4.40) |
| Papua New Guinea | female | 1,392.91(968.38; 1,919.48) | 2,717.02(1,797.34; 3,668.74) | 3.29(-4.87; 12.15) | 73.59(51.97; 99.19) | 53.09(35.68; 70.36) | -0.02(-4.08; 4.21) |
| Cameroon | female | 2,916.67(1,908.02; 4,097.39) | 6,333.25(4,080.59; 8,910.46) | 3.99(-5.07; 13.91) | 67.19(44.71; 93.05) | 43.13(28.12; 58.90) | 0.02(-3.84; 4.03) |
| Belarus | female | 8,309.58(6,152.79; 10,824.69) | 7,660.08(6,116.09; 9,293.45) | -0.11(-9.01; 9.66) | 145.95(107.11; 192.43) | 146.09(112.38; 179.98) | -0.01(-5.08; 5.32) |
| Honduras | female | 1,344.98(944.25; 1,813.98) | 4,036.89(2,889.57; 5,383.51) | 3.42(-5.14; 12.75) | 69.35(49.87; 91.46) | 72.61(52.20; 95.92) | 0.01(-4.36; 4.57) |
| Poland | female | 16,564.76(12,064.66; 22,546.36) | 11,707.80(8,851.80; 14,635.38) | 0.03(-9.28; 10.28) | 87.17(62.96; 119.57) | 63.86(47.04; 82.22) | -0.01(-4.25; 4.41) |
| Montenegro | female | 245.55(170.54; 337.95) | 149.82(108.77; 194.26) | -0.07(-5.17; 5.32) | 76.26(52.84; 104.87) | 48.46(34.98; 63.34) | 0.01(-3.98; 4.16) |
| Angola | female | 2,167.47(1,460.61; 2,995.74) | 6,169.05(4,197.98; 8,498.31) | 4.03(-5.00; 13.92) | 50.58(34.56; 68.78) | 42.92(29.30; 58.03) | 0.01(-3.84; 4.00) |
| Mauritius | female | 562.38(372.39; 793.76) | 560.55(411.56; 734.03) | 0.39(-6.02; 7.23) | 93.32(62.15; 129.92) | 80.67(58.17; 106.12) | 0.00(-4.47; 4.68) |
| Belize | female | 63.31(42.59; 87.79) | 177.37(121.69; 239.05) | 3.50(-1.93; 9.22) | 75.45(51.10; 102.40) | 74.19(51.39; 99.32) | 0.00(-4.39; 4.60) |
| Eritrea | female | 521.94(343.53; 734.00) | 1,305.00(922.93; 1,707.71) | 3.16(-4.26; 11.16) | 41.00(27.68; 56.67) | 42.30(30.23; 54.86) | 0.00(-3.83; 3.99) |
| United States of America | both | 1,307,624.11(988,964.13; 1,654,165.25) | 6,484,690.40(5,471,717.29; 7,481,321.26) | 5.59(-10.32; 24.33) | 479.21(361.89; 608.89) | 1,944.08(1,632.99; 2,249.41) | 4.88(-3.07; 13.49) |
| Sweden | both | 11,528.05(9,426.87; 14,021.57) | 41,991.48(34,777.96; 49,150.85) | 4.52(-6.47; 16.79) | 133.94(108.94; 162.97) | 430.88(354.39; 505.09) | 4.10(-2.29; 10.91) |
| Estonia | both | 4,938.21(3,765.23; 6,189.12) | 8,760.94(7,066.45; 10,562.38) | 3.17(-6.13; 13.40) | 314.90(240.13; 395.89) | 733.92(586.79; 889.92) | 3.91(-2.98; 11.28) |
| Canada | both | 88,964.62(68,715.34; 112,211.71) | 322,467.47(279,922.35; 364,203.71) | 4.34(-8.58; 19.09) | 301.00(232.06; 381.56) | 877.19(759.19; 998.21) | 3.67(-3.41; 11.27) |
| South Africa | both | 186,832.78(128,949.13; 255,742.29) | 95,461.25(77,951.10; 112,747.97) | -2.08(-13.11; 10.35) | 492.92(344.06; 667.71) | 157.73(129.31; 185.46) | -3.62(-8.59; 1.62) |
| Slovenia | both | 1,662.63(1,223.75; 2,210.75) | 3,257.26(2,591.37; 3,889.53) | 2.74(-5.58; 11.81) | 81.76(60.12; 108.99) | 178.61(141.47; 215.11) | 3.11(-2.33; 8.85) |
| Libya | both | 5,375.43(4,081.87; 6,848.51) | 20,407.25(16,607.01; 25,162.83) | 6.18(-4.26; 17.75) | 156.72(120.35; 196.54) | 251.47(204.48; 310.75) | 2.96(-2.82; 9.07) |
| China | both | 3,578,492.19(2,821,561.76; 4,421,610.17) | 1,661,208.48(1,278,813.66; 2,030,011.63) | -2.29(-15.85; 13.44) | 275.72(220.07; 337.63) | 116.47(89.13; 143.59) | -2.78(-7.49; 2.17) |
| Ireland | both | 5,925.55(4,437.41; 7,537.80) | 19,792.15(16,466.93; 22,921.86) | 3.82(-6.38; 15.12) | 166.09(124.68; 210.94) | 414.53(342.12; 481.53) | 2.64(-3.63; 9.32) |
| Finland | both | 13,914.85(11,733.13; 16,548.87) | 26,799.59(23,007.65; 30,716.38) | 2.45(-7.90; 13.95) | 259.06(217.19; 312.60) | 517.31(441.03; 595.09) | 2.59(-3.90; 9.52) |
| Italy | both | 156,149.30(124,020.06; 193,518.38) | 74,047.99(56,080.22; 90,333.03) | -2.85(-13.56; 9.19) | 265.21(209.85; 329.51) | 138.92(104.24; 173.13) | -2.55(-7.43; 2.59) |
| Switzerland | both | 36,955.59(29,912.01; 44,143.64) | 23,726.41(19,859.50; 27,775.09) | -1.79(-11.57; 9.07) | 501.43(405.72; 600.70) | 269.87(224.64; 317.70) | -2.46(-7.98; 3.40) |
| Lithuania | both | 7,754.91(6,223.79; 9,489.15) | 10,056.85(8,329.33; 11,910.94) | 0.98(-8.27; 11.16) | 201.70(161.68; 247.40) | 386.60(317.58; 458.25) | 2.39(-3.77; 8.95) |
| Turkmenistan | both | 3,359.19(2,359.79; 4,546.86) | 11,629.85(8,851.16; 14,632.03) | 3.78(-5.87; 14.41) | 93.56(66.39; 123.87) | 215.12(164.10; 270.38) | 2.35(-3.22; 8.25) |
| Tunisia | both | 7,306.64(5,393.58; 9,575.53) | 20,564.29(15,802.74; 25,526.17) | 3.80(-6.41; 15.12) | 92.57(68.54; 118.85) | 165.06(127.32; 203.38) | 2.24(-3.06; 7.83) |
| Yemen | both | 6,503.46(4,790.10; 8,504.99) | 39,974.70(29,242.70; 52,827.54) | 6.16(-4.93; 18.54) | 67.02(50.71; 87.10) | 133.48(98.84; 174.39) | 2.23(-2.85; 7.58) |
| Bosnia and Herzegovina | both | 2,204.22(1,523.99; 3,047.66) | 1,984.62(1,441.71; 2,515.53) | 1.06(-6.66; 9.41) | 43.93(30.62; 60.63) | 62.65(45.37; 81.24) | 2.21(-2.11; 6.73) |
| Puerto Rico | both | 7,938.80(5,963.45; 10,320.93) | 8,197.18(6,920.84; 9,685.82) | -2.40(-11.28; 7.37) | 216.12(162.46; 280.46) | 251.83(212.35; 298.31) | -2.09(-7.75; 3.93) |
| Mauritius | both | 1,276.77(867.84; 1,772.73) | 2,518.34(2,107.89; 2,946.89) | 2.30(-5.72; 11.01) | 103.45(70.60; 142.41) | 180.59(150.11; 213.30) | 1.96(-3.42; 7.65) |
| United Kingdom | both | 169,364.53(131,801.58; 212,179.96) | 370,988.45(318,124.07; 421,932.29) | 2.44(-10.37; 17.08) | 288.21(223.58; 362.27) | 554.29(472.29; 632.12) | 1.91(-4.59; 8.84) |
| Brunei Darussalam | both | 471.43(368.02; 593.54) | 605.09(455.36; 754.54) | 0.47(-6.03; 7.42) | 181.15(145.73; 222.81) | 112.67(84.86; 140.97) | -1.85(-6.58; 3.12) |
| Syrian Arab Republic | both | 9,434.90(7,157.25; 12,006.50) | 17,882.23(13,826.30; 21,895.19) | 2.48(-7.46; 13.50) | 89.19(68.98; 111.73) | 134.46(103.85; 164.48) | 1.85(-3.23; 7.19) |
| North Macedonia | both | 1,339.78(941.82; 1,837.86) | 2,195.84(1,648.89; 2,748.27) | 2.10(-5.81; 10.67) | 63.16(44.42; 86.42) | 96.50(71.81; 121.50) | 1.80(-2.97; 6.81) |
| Sudan | both | 14,012.83(10,733.36; 17,941.27) | 67,702.10(52,052.84; 83,607.75) | 4.68(-6.76; 17.53) | 83.96(64.83; 107.51) | 161.57(124.37; 198.88) | 1.78(-3.48; 7.31) |
| Spain | both | 109,929.96(86,672.46; 135,993.75) | 93,863.50(75,485.15; 114,315.04) | -1.48(-12.58; 11.02) | 274.45(216.51; 339.56) | 218.13(170.99; 268.00) | -1.75(-7.13; 3.95) |
| Lebanon | both | 4,546.91(3,515.47; 5,765.89) | 8,718.23(6,503.56; 11,045.44) | 4.29(-5.13; 14.65) | 156.38(121.46; 196.28) | 141.39(104.26; 179.73) | 1.73(-3.40; 7.14) |
| France | both | 84,237.11(65,044.74; 105,672.16) | 138,400.13(115,480.89; 161,523.85) | 2.08(-9.78; 15.50) | 141.10(108.30; 177.54) | 200.59(165.19; 237.62) | 1.70(-3.79; 7.51) |
| Malta | both | 596.18(446.91; 765.44) | 936.71(759.68; 1,105.19) | 2.13(-4.96; 9.75) | 159.07(118.65; 204.53) | 226.98(182.96; 268.43) | 1.68(-3.97; 7.66) |
| Mozambique | both | 5,262.56(3,770.95; 6,999.20) | 15,691.79(11,574.65; 20,502.95) | 4.40(-5.58; 15.44) | 51.34(37.56; 67.51) | 64.16(48.75; 82.74) | 1.67(-2.65; 6.18) |
| United Arab Emirates | both | 4,901.24(3,750.19; 6,165.61) | 24,149.81(17,987.69; 30,404.48) | 8.91(-2.03; 21.08) | 225.99(174.58; 281.00) | 200.82(149.90; 252.32) | 1.62(-3.89; 7.45) |
| Iceland | both | 711.62(574.14; 863.58) | 1,807.94(1,533.53; 2,059.96) | 2.54(-5.15; 10.85) | 262.37(211.42; 318.87) | 510.21(432.42; 583.44) | 1.61(-4.76; 8.40) |
| Czechia | both | 10,633.14(7,612.23; 14,558.85) | 13,676.03(10,920.76; 16,658.24) | 1.35(-8.24; 11.94) | 106.45(74.87; 146.28) | 143.42(113.30; 176.56) | 1.61(-3.53; 7.01) |
| Nicaragua | both | 3,529.01(2,450.12; 4,723.61) | 4,541.39(3,236.05; 5,852.36) | 0.98(-7.50; 10.24) | 109.28(77.37; 143.71) | 63.46(45.27; 80.97) | -1.51(-5.68; 2.85) |
| Albania | both | 2,285.33(1,584.83; 3,157.66) | 3,518.12(2,754.23; 4,366.39) | 0.70(-7.52; 9.65) | 64.90(45.70; 89.12) | 125.50(97.97; 155.66) | 1.50(-3.49; 6.74) |
| Croatia | both | 5,890.21(4,317.46; 7,696.30) | 7,248.93(5,835.67; 8,610.64) | 0.79(-8.16; 10.61) | 117.59(85.86; 153.50) | 179.36(142.59; 214.50) | 1.48(-3.91; 7.18) |
| Algeria | both | 24,712.36(19,034.26; 30,887.24) | 75,711.58(58,693.11; 93,667.72) | 3.93(-7.55; 16.84) | 112.27(88.30; 138.79) | 167.13(129.49; 207.11) | 1.47(-3.80; 7.03) |
| Greenland | both | 172.29(131.60; 218.30) | 197.90(158.82; 237.69) | 1.23(-4.21; 6.99) | 257.27(197.34; 324.01) | 332.53(267.32; 400.83) | 1.46(-4.51; 7.80) |
| Gabon | both | 1,206.38(866.94; 1,590.26) | 1,219.64(911.89; 1,553.61) | 0.92(-6.27; 8.67) | 140.77(103.57; 181.24) | 69.92(52.73; 89.23) | -1.41(-5.69; 3.05) |
| Uzbekistan | both | 15,285.71(10,721.04; 20,921.42) | 38,852.01(27,983.51; 48,594.19) | 3.53(-7.26; 15.58) | 76.89(55.12; 103.85) | 107.79(77.72; 134.72) | 1.41(-3.42; 6.48) |
| Philippines | both | 74,406.97(53,547.50; 98,266.65) | 69,767.90(48,831.06; 90,873.22) | 0.76(-10.29; 13.17) | 120.14(87.20; 156.84) | 57.95(41.23; 74.90) | -1.36(-5.46; 2.91) |
| Djibouti | both | 202.76(142.99; 280.93) | 1,116.30(767.52; 1,498.75) | 5.50(-1.93; 13.48) | 47.37(34.27; 63.72) | 84.04(58.17; 112.06) | 1.36(-3.21; 6.15) |
| Serbia | both | 7,300.82(5,476.33; 9,397.52) | 9,528.37(7,559.46; 11,531.67) | 0.84(-8.35; 10.96) | 78.67(59.02; 100.73) | 105.93(83.54; 127.73) | 1.34(-3.48; 6.40) |
| Bulgaria | both | 8,507.16(6,175.50; 11,250.69) | 7,756.02(6,166.99; 9,369.25) | 0.11(-8.82; 9.91) | 101.25(72.71; 135.81) | 130.03(101.41; 161.12) | 1.24(-3.76; 6.49) |
| Zimbabwe | both | 18,339.55(12,469.32; 26,071.12) | 14,067.52(10,693.23; 17,644.91) | 0.10(-9.38; 10.57) | 216.84(151.63; 295.78) | 106.19(81.88; 130.44) | -1.22(-5.91; 3.71) |
| Comoros | both | 169.90(118.19; 233.38) | 603.98(443.61; 792.58) | 3.33(-3.33; 10.45) | 44.55(31.64; 59.50) | 82.95(61.11; 109.32) | 1.22(-3.34; 5.99) |
| Greece | both | 12,656.31(9,655.64; 15,698.95) | 23,603.94(19,830.10; 27,178.65) | 0.67(-9.41; 11.86) | 122.58(93.91; 152.64) | 256.32(212.32; 296.43) | 1.20(-4.53; 7.27) |
| Cuba | both | 15,427.58(10,526.52; 20,902.76) | 8,530.23(6,113.80; 11,237.54) | -1.55(-10.42; 8.19) | 125.46(86.55; 168.67) | 78.16(55.30; 102.49) | -1.18(-5.57; 3.41) |
| Afghanistan | both | 15,449.30(11,516.19; 20,295.60) | 54,152.69(43,238.47; 66,734.01) | 4.74(-6.50; 17.32) | 175.69(131.24; 229.13) | 203.76(162.87; 250.12) | 1.18(-4.28; 6.94) |
| Kiribati | both | 85.47(63.45; 109.77) | 395.33(297.04; 521.03) | 3.16(-3.07; 9.80) | 116.50(87.38; 147.31) | 322.76(242.47; 419.61) | 1.16(-4.76; 7.45) |
| Congo | both | 2,393.25(1,728.34; 3,189.12) | 3,445.73(2,565.08; 4,362.05) | 1.89(-6.40; 10.91) | 118.06(86.40; 154.59) | 66.07(49.55; 83.27) | -1.15(-5.38; 3.27) |
| Namibia | both | 2,512.12(1,729.41; 3,425.61) | 2,282.68(1,698.50; 2,860.56) | 0.94(-6.88; 9.42) | 198.66(137.91; 263.89) | 96.73(73.34; 119.31) | -1.13(-5.74; 3.71) |
| Senegal | both | 5,110.52(3,554.14; 7,049.78) | 5,844.04(3,974.18; 7,879.93) | 1.72(-7.06; 11.34) | 86.48(61.59; 115.48) | 40.25(27.73; 53.38) | -1.09(-4.84; 2.80) |
| Kazakhstan | both | 34,183.88(26,055.92; 43,494.78) | 61,430.03(49,033.50; 74,072.77) | 1.86(-9.19; 14.26) | 205.54(157.25; 260.23) | 314.08(249.39; 378.82) | 1.08(-4.82; 7.34) |
| Dominican Republic | both | 4,870.77(3,415.78; 6,627.87) | 8,243.89(5,800.76; 10,930.36) | 2.69(-6.52; 12.81) | 68.40(49.05; 90.35) | 69.62(48.97; 92.23) | 1.08(-3.30; 5.66) |
| United Republic of Tanzania | both | 13,315.55(9,478.89; 17,901.06) | 46,386.29(33,158.47; 61,106.19) | 3.87(-7.10; 16.14) | 63.75(46.59; 84.67) | 95.07(68.53; 125.50) | 1.07(-3.59; 5.97) |
| Hungary | both | 10,000.19(6,927.66; 14,099.52) | 9,575.33(7,309.64; 12,025.06) | 0.53(-8.63; 10.62) | 100.43(69.13; 142.84) | 109.92(82.51; 140.09) | 1.05(-3.78; 6.12) |
| Kuwait | both | 2,064.77(1,430.99; 2,811.13) | 7,513.36(5,828.98; 9,342.42) | 5.15(-4.20; 15.41) | 99.64(70.55; 133.54) | 128.51(98.92; 162.26) | 1.01(-4.00; 6.27) |
| Latvia | both | 10,362.63(7,623.39; 13,466.46) | 4,784.32(3,905.12; 5,665.64) | -2.32(-10.57; 6.69) | 379.56(277.82; 499.88) | 272.59(220.70; 323.57) | -0.99(-6.59; 4.95) |
| Slovakia | both | 7,560.72(5,795.78; 9,591.73) | 5,558.29(4,250.61; 6,844.57) | -1.00(-9.52; 8.33) | 141.95(108.20; 180.35) | 105.55(80.68; 130.45) | -0.98(-5.68; 3.96) |
| Sri Lanka | both | 27,313.47(20,350.31; 35,008.68) | 20,070.13(15,191.84; 25,317.44) | -0.29(-10.06; 10.55) | 153.25(115.20; 194.70) | 88.35(66.28; 111.91) | -0.97(-5.49; 3.76) |
| Bhutan | both | 308.72(213.34; 424.07) | 546.60(402.96; 707.78) | 2.93(-3.62; 9.93) | 55.46(39.41; 74.22) | 65.05(48.01; 83.53) | 0.97(-3.33; 5.46) |
| Bangladesh | both | 58,259.67(42,206.40; 75,315.83) | 110,108.89(81,723.69; 141,970.31) | 3.06(-8.68; 16.30) | 63.83(47.14; 81.64) | 64.28(48.17; 82.75) | 0.97(-3.32; 5.45) |
| Belgium | both | 17,653.54(13,158.09; 22,840.46) | 23,874.76(19,933.02; 27,918.96) | 1.26(-8.81; 12.44) | 170.10(126.42; 219.69) | 214.45(177.84; 253.00) | 0.95(-4.51; 6.72) |
| Mongolia | both | 1,999.57(1,440.17; 2,675.00) | 4,321.35(3,218.70; 5,432.58) | 2.91(-5.70; 12.30) | 98.18(71.79; 128.23) | 129.55(96.31; 163.39) | 0.94(-4.06; 6.20) |
| Morocco | both | 31,807.90(22,649.66; 42,357.37) | 64,610.83(50,122.18; 80,784.70) | 2.56(-8.62; 15.10) | 133.95(96.84; 173.53) | 165.53(128.50; 206.81) | 0.93(-4.30; 6.45) |
| Micronesia (Federated States of) | both | 129.98(95.61; 169.47) | 84.20(60.08; 108.82) | -0.46(-4.96; 4.25) | 145.12(108.84; 186.89) | 77.86(55.88; 100.16) | -0.90(-5.30; 3.71) |
| Oman | both | 1,449.20(999.53; 1,993.61) | 5,226.39(3,781.67; 6,776.66) | 5.52(-3.49; 15.36) | 78.93(56.07; 106.10) | 89.80(64.70; 115.19) | 0.89(-3.73; 5.74) |
| Bolivia (Plurinational State of) | both | 10,234.30(7,078.09; 13,733.33) | 12,393.19(9,211.97; 15,650.09) | 1.68(-7.83; 12.17) | 176.76(123.98; 234.20) | 99.48(74.10; 125.62) | -0.88(-5.53; 3.99) |
| Turkey | both | 57,402.28(38,825.30; 79,411.87) | 92,809.71(67,604.76; 117,398.23) | 2.34(-9.16; 15.29) | 98.74(67.74; 134.55) | 104.49(76.03; 132.91) | 0.88(-3.89; 5.90) |
| Sierra Leone | both | 2,845.96(1,933.11; 3,924.26) | 3,392.21(2,354.65; 4,563.26) | 2.83(-5.51; 11.91) | 92.73(63.60; 126.11) | 40.61(28.44; 53.56) | -0.86(-4.63; 3.06) |
| Egypt | both | 38,762.74(25,438.25; 54,941.75) | 103,306.30(71,061.82; 135,564.92) | 3.26(-8.44; 16.46) | 75.31(50.10; 105.29) | 99.09(68.94; 128.83) | 0.86(-3.86; 5.80) |
| Democratic People's Republic of Korea | both | 39,757.35(29,321.90; 51,611.45) | 32,977.93(25,136.30; 42,224.87) | 0.15(-10.14; 11.62) | 187.36(139.04; 242.14) | 113.50(85.73; 146.30) | -0.84(-5.61; 4.18) |
| Myanmar | both | 37,723.90(27,134.41; 49,404.83) | 36,112.18(24,967.26; 47,949.34) | 0.32(-10.07; 11.91) | 93.08(68.13; 121.32) | 61.29(42.51; 81.05) | -0.83(-4.99; 3.52) |
| Central African Republic | both | 1,930.34(1,368.74; 2,524.69) | 2,663.75(1,888.95; 3,418.12) | 1.45(-6.55; 10.13) | 82.18(59.64; 106.36) | 54.61(38.63; 69.96) | -0.83(-4.88; 3.40) |
| Austria | both | 17,150.87(12,986.10; 21,560.56) | 21,747.87(17,849.27; 25,514.27) | 0.99(-8.99; 12.06) | 205.46(155.36; 258.08) | 251.89(204.95; 297.04) | 0.83(-4.82; 6.82) |
| Qatar | both | 668.05(469.26; 890.19) | 3,719.32(2,640.72; 4,932.59) | 8.01(-0.90; 17.73) | 126.70(91.25; 165.67) | 87.42(63.03; 116.31) | -0.82(-5.34; 3.93) |
| Trinidad and Tobago | both | 1,183.63(818.11; 1,623.94) | 1,389.48(1,067.74; 1,723.71) | 1.42(-5.96; 9.38) | 96.94(67.84; 130.80) | 97.73(73.59; 122.05) | 0.82(-3.89; 5.75) |
| Botswana | both | 1,992.82(1,375.07; 2,701.01) | 2,202.18(1,619.07; 2,787.48) | 1.80(-6.05; 10.30) | 171.30(119.71; 228.97) | 88.09(65.96; 110.86) | -0.80(-5.33; 3.96) |
| Luxembourg | both | 1,095.34(891.96; 1,326.67) | 1,758.74(1,444.38; 2,069.81) | 0.72(-6.82; 8.87) | 264.11(213.71; 320.07) | 261.12(213.41; 307.89) | -0.79(-6.39; 5.15) |
| Saudi Arabia | both | 14,239.22(10,187.67; 19,337.69) | 45,734.70(32,214.91; 60,908.36) | 4.96(-6.14; 17.38) | 96.77(70.91; 128.08) | 93.75(65.88; 124.07) | 0.79(-3.87; 5.68) |
| Ethiopia | both | 23,940.68(17,576.36; 31,148.43) | 44,352.83(33,204.86; 57,274.04) | 2.16(-8.60; 14.20) | 61.20(45.86; 79.30) | 45.56(34.27; 57.71) | -0.78(-4.65; 3.25) |
| Pakistan | both | 129,909.85(88,620.50; 184,693.97) | 160,868.99(123,985.22; 201,662.39) | 2.08(-9.91; 15.66) | 139.90(95.60; 195.10) | 72.36(56.70; 88.86) | -0.77(-5.12; 3.78) |
| Iran (Islamic Republic of) | both | 138,937.67(110,014.51; 173,533.53) | 321,123.96(268,927.05; 373,935.04) | 3.23(-9.57; 17.84) | 277.50(224.30; 339.16) | 339.36(282.07; 397.38) | 0.74(-5.21; 7.07) |
| Ukraine | both | 137,959.14(110,425.13; 169,559.52) | 120,508.49(95,752.25; 147,203.64) | -0.05(-11.53; 12.92) | 248.36(196.12; 308.62) | 279.62(219.77; 344.82) | 0.70(-5.04; 6.79) |
| Eswatini | both | 1,181.29(827.62; 1,614.02) | 1,107.04(834.41; 1,401.37) | 0.98(-6.14; 8.64) | 176.92(124.81; 238.56) | 100.77(78.29; 125.21) | -0.67(-5.34; 4.24) |
| Eritrea | both | 1,400.17(979.48; 1,885.75) | 5,233.86(3,626.71; 7,176.51) | 3.92(-4.94; 13.60) | 56.36(40.59; 74.63) | 85.17(59.14; 117.54) | 0.67(-3.89; 5.44) |
| Togo | both | 1,594.49(1,136.28; 2,140.93) | 2,922.36(1,994.24; 3,891.47) | 3.89(-4.40; 12.89) | 55.37(39.94; 72.55) | 37.80(26.10; 49.61) | 0.67(-3.08; 4.57) |
| Barbados | both | 339.00(234.54; 468.08) | 260.87(189.77; 336.35) | -0.29(-5.91; 5.66) | 124.21(86.13; 170.12) | 89.49(64.57; 116.73) | -0.66(-5.21; 4.11) |
| Andorra | both | 86.19(61.16; 115.08) | 87.30(62.28; 112.89) | 0.95(-3.65; 5.77) | 131.42(93.00; 175.54) | 105.00(74.81; 137.71) | 0.66(-4.11; 5.67) |
| Gambia | both | 682.36(466.39; 944.30) | 889.53(619.98; 1,169.83) | 2.47(-4.53; 9.97) | 84.93(58.61; 113.58) | 40.46(28.72; 52.18) | -0.65(-4.42; 3.28) |
| Viet Nam | both | 89,301.80(64,952.22; 116,707.99) | 146,961.63(111,275.02; 185,576.44) | 2.25(-9.68; 15.74) | 132.62(97.27; 171.83) | 140.56(107.02; 176.80) | 0.64(-4.41; 5.96) |
| Rwanda | both | 2,953.18(2,101.86; 3,933.49) | 10,024.81(7,111.81; 13,396.36) | 3.90(-5.60; 14.35) | 50.05(36.30; 65.34) | 80.54(57.06; 106.74) | 0.63(-3.87; 5.33) |
| Brazil | both | 236,342.85(162,996.37; 324,191.34) | 306,463.89(234,174.76; 376,925.19) | 2.05(-10.55; 16.41) | 154.16(107.66; 208.61) | 131.43(99.94; 162.15) | 0.62(-4.38; 5.88) |
| Paraguay | both | 5,299.65(3,354.78; 7,676.22) | 6,092.34(4,391.33; 7,937.68) | 1.86(-6.99; 11.54) | 139.30(89.87; 199.27) | 78.01(56.42; 101.02) | -0.60(-5.02; 4.02) |
| Sao Tome and Principe | both | 87.54(60.11; 118.82) | 112.56(82.79; 144.71) | 2.11(-2.79; 7.25) | 95.22(66.31; 127.86) | 53.84(39.92; 68.23) | -0.59(-4.65; 3.63) |
| Armenia | both | 3,690.36(2,546.50; 5,115.91) | 3,497.52(2,481.31; 4,456.25) | 0.36(-7.83; 9.29) | 102.58(71.38; 140.87) | 113.38(79.07; 145.88) | 0.59(-4.25; 5.68) |
| Bermuda | both | 113.45(83.74; 146.86) | 76.93(60.18; 93.02) | -0.88(-5.30; 3.74) | 165.72(122.10; 215.08) | 126.52(97.57; 155.03) | -0.58(-5.49; 4.58) |
| Saint Kitts and Nevis | both | 44.75(31.51; 59.15) | 54.81(39.79; 69.70) | 1.09(-3.05; 5.41) | 109.97(78.55; 144.63) | 85.06(61.34; 108.84) | -0.58(-5.08; 4.14) |
| Venezuela (Bolivarian Republic of) | both | 18,050.49(12,349.11; 25,002.58) | 16,597.29(11,744.44; 21,426.11) | 1.03(-8.72; 11.83) | 99.39(68.90; 136.12) | 63.70(45.37; 82.72) | -0.58(-4.81; 3.83) |
| Fiji | both | 749.22(541.46; 1,009.61) | 598.11(421.18; 787.77) | 0.10(-6.35; 7.00) | 96.89(70.70; 128.23) | 64.06(45.16; 84.29) | -0.58(-4.80; 3.83) |
| Republic of Moldova | both | 8,717.52(6,481.92; 11,259.56) | 5,875.32(4,602.94; 7,151.44) | -0.96(-9.52; 8.42) | 192.96(144.00; 248.61) | 154.35(119.94; 191.23) | -0.57(-5.64; 4.78) |
| Peru | both | 39,986.43(26,421.81; 57,301.52) | 34,169.34(26,341.98; 42,675.53) | 1.20(-9.23; 12.83) | 184.78(124.15; 262.42) | 87.87(68.13; 109.57) | -0.55(-5.10; 4.22) |
| Niue | both | 2.59(1.92; 3.38) | 1.22(0.88; 1.58) | -1.61(-1.96; -1.26) | 125.33(93.09; 164.07) | 75.32(53.91; 97.89) | -0.55(-4.94; 4.03) |
| Taiwan (Province of China) | both | 42,602.48(32,833.14; 54,040.20) | 41,919.97(33,592.24; 50,851.21) | 0.94(-9.66; 12.78) | 187.19(145.34; 234.80) | 165.50(130.50; 203.15) | 0.54(-4.67; 6.03) |
| Netherlands | both | 23,605.99(17,224.43; 30,791.44) | 27,563.17(22,125.72; 33,464.41) | 0.49(-9.66; 11.78) | 145.23(105.23; 189.73) | 168.58(133.69; 205.61) | 0.53(-4.70; 6.04) |
| Cyprus | both | 1,058.43(809.57; 1,318.72) | 2,217.97(1,744.52; 2,714.68) | 2.69(-5.22; 11.27) | 129.66(99.47; 161.80) | 152.61(119.09; 186.84) | 0.53(-4.59; 5.93) |
| Nauru | both | 12.18(9.07; 15.68) | 8.61(6.08; 11.26) | -0.14(-2.36; 2.14) | 129.04(97.56; 163.81) | 76.29(54.26; 98.73) | -0.52(-4.92; 4.09) |
| Denmark | both | 16,270.52(13,417.44; 19,330.71) | 22,161.65(18,557.73; 25,555.66) | 0.58(-9.39; 11.65) | 298.18(244.92; 355.03) | 378.43(314.58; 437.72) | 0.50(-5.55; 6.93) |
| New Zealand | both | 8,508.24(6,120.50; 11,384.05) | 14,155.13(11,367.97; 16,868.77) | 1.25(-8.31; 11.81) | 234.45(168.76; 313.85) | 273.84(217.67; 327.59) | 0.50(-5.18; 6.52) |
| Jordan | both | 3,459.04(2,396.34; 4,646.72) | 12,170.99(8,556.13; 16,214.01) | 5.02(-4.78; 15.83) | 100.49(70.11; 132.69) | 90.59(63.86; 119.88) | 0.49(-4.12; 5.33) |
| Uganda | both | 8,557.77(5,921.12; 11,845.84) | 26,233.80(19,419.64; 34,434.39) | 3.58(-6.82; 15.14) | 61.17(43.47; 83.18) | 77.94(57.52; 102.99) | 0.49(-3.97; 5.16) |
| Solomon Islands | both | 332.26(238.39; 433.64) | 477.02(347.46; 626.13) | 2.01(-4.33; 8.78) | 112.03(82.66; 143.99) | 70.61(51.45; 91.58) | -0.48(-4.80; 4.04) |
| Mauritania | both | 1,458.41(1,031.35; 1,995.81) | 1,610.82(1,088.54; 2,167.79) | 2.13(-5.43; 10.29) | 86.99(62.46; 115.91) | 42.00(28.27; 55.52) | -0.48(-4.30; 3.49) |
| Germany | both | 167,181.86(134,981.72; 204,665.35) | 192,914.74(158,897.12; 225,741.95) | 0.31(-11.64; 13.87) | 198.05(159.26; 243.58) | 235.19(192.38; 277.40) | 0.46(-5.09; 6.34) |
| Guam | both | 189.34(137.30; 253.80) | 116.39(82.25; 151.97) | -0.34(-5.17; 4.74) | 123.03(90.49; 163.23) | 73.34(51.31; 96.78) | -0.46(-4.83; 4.11) |
| Azerbaijan | both | 6,713.94(4,669.11; 9,190.12) | 12,309.28(9,076.40; 15,759.03) | 2.03(-7.51; 12.56) | 86.73(60.94; 117.07) | 105.87(77.21; 135.95) | 0.45(-4.31; 5.45) |
| Chad | both | 2,882.77(2,019.76; 3,943.69) | 5,042.20(3,434.73; 6,757.68) | 3.02(-5.73; 12.57) | 63.07(44.58; 84.52) | 37.39(25.90; 49.29) | -0.45(-4.15; 3.39) |
| Mali | both | 3,867.57(2,739.77; 5,221.40) | 7,137.02(4,933.03; 9,650.46) | 3.04(-6.04; 13.00) | 57.64(41.25; 77.38) | 36.87(25.47; 48.51) | -0.45(-4.13; 3.36) |
| United States Virgin Islands | both | 197.82(157.82; 242.40) | 155.36(121.42; 203.39) | -1.01(-6.18; 4.45) | 185.73(148.45; 228.23) | 221.66(170.49; 294.74) | -0.44(-5.98; 5.43) |
| Indonesia | both | 138,806.04(95,946.45; 191,986.46) | 142,366.22(100,397.10; 192,681.23) | 0.88(-10.85; 14.16) | 74.30(52.35; 100.79) | 47.44(33.63; 64.09) | -0.44(-4.37; 3.65) |
| Malawi | both | 4,621.08(3,300.43; 6,164.50) | 12,937.97(9,407.04; 17,366.35) | 2.96(-6.70; 13.62) | 58.64(42.77; 77.32) | 79.89(57.75; 106.44) | 0.44(-4.05; 5.13) |
| Maldives | both | 156.01(108.21; 214.88) | 506.90(364.03; 660.97) | 4.92(-1.66; 11.95) | 81.37(58.07; 110.36) | 76.24(53.87; 98.94) | 0.43(-4.01; 5.06) |
| Costa Rica | both | 2,920.85(2,086.23; 3,949.51) | 4,427.35(3,430.48; 5,592.32) | 2.21(-6.34; 11.54) | 97.41(70.86; 129.45) | 85.71(66.16; 107.90) | 0.42(-4.12; 5.18) |
| Somalia | both | 2,680.41(1,906.33; 3,629.32) | 11,136.80(7,646.89; 16,033.19) | 4.23(-5.40; 14.84) | 48.44(35.04; 64.51) | 65.97(44.87; 95.54) | 0.42(-3.87; 4.91) |
| Israel | both | 7,049.02(5,371.01; 8,967.54) | 14,044.73(11,250.32; 16,816.25) | 2.43(-7.27; 13.16) | 146.46(111.70; 186.52) | 153.65(122.92; 184.31) | 0.41(-4.74; 5.83) |
| Suriname | both | 349.73(241.69; 474.34) | 590.78(452.98; 745.29) | 1.92(-4.66; 8.95) | 87.51(61.58; 117.91) | 100.07(76.21; 126.53) | 0.41(-4.31; 5.37) |
| Côte d'Ivoire | both | 7,361.41(5,169.30; 9,898.14) | 9,828.45(6,733.77; 13,019.06) | 2.48(-6.88; 12.78) | 72.49(51.86; 95.36) | 38.76(26.67; 50.76) | -0.40(-4.14; 3.49) |
| Madagascar | both | 5,373.71(3,914.91; 7,306.68) | 17,132.20(12,463.98; 22,366.71) | 3.50(-6.48; 14.55) | 54.40(40.82; 72.01) | 69.86(51.13; 91.07) | 0.40(-3.95; 4.94) |
| Burkina Faso | both | 3,640.03(2,622.36; 4,800.29) | 7,212.61(4,952.82; 9,520.33) | 3.88(-5.29; 13.95) | 52.87(39.08; 68.91) | 38.59(26.98; 50.61) | 0.39(-3.37; 4.30) |
| Montenegro | both | 779.16(557.84; 1,030.55) | 438.90(319.00; 565.91) | -0.56(-6.70; 5.97) | 118.72(85.08; 157.12) | 72.47(52.23; 94.27) | -0.37(-4.74; 4.20) |
| Uruguay | both | 6,072.87(4,013.43; 8,515.26) | 4,186.53(3,082.13; 5,300.22) | -0.03(-8.35; 9.04) | 198.16(130.69; 278.79) | 122.11(89.36; 155.52) | -0.36(-5.22; 4.76) |
| Bahamas | both | 336.82(236.37; 455.18) | 388.35(280.96; 507.91) | 0.93(-5.15; 7.40) | 119.94(85.22; 160.31) | 92.01(66.36; 120.85) | -0.36(-4.95; 4.46) |
| Northern Mariana Islands | both | 70.48(50.71; 93.29) | 40.65(29.31; 53.45) | -1.84(-5.64; 2.12) | 126.81(93.41; 166.12) | 86.24(61.67; 113.89) | -0.36(-4.88; 4.38) |
| Panama | both | 2,012.44(1,396.01; 2,708.72) | 3,635.22(2,764.65; 4,527.50) | 2.25(-6.11; 11.36) | 83.17(58.83; 110.29) | 82.51(62.54; 102.86) | 0.36(-4.15; 5.08) |
| Nigeria | both | 48,884.61(34,441.10; 64,961.24) | 65,977.20(46,950.88; 86,392.67) | 2.65(-8.55; 15.23) | 64.47(46.09; 85.19) | 33.93(24.41; 43.68) | -0.36(-3.97; 3.39) |
| Marshall Islands | both | 47.05(34.93; 61.32) | 45.43(32.59; 59.13) | 0.96(-2.98; 5.06) | 122.08(92.09; 156.80) | 76.99(56.01; 99.43) | -0.35(-4.76; 4.27) |
| Ecuador | both | 9,976.20(6,992.77; 13,524.68) | 17,797.96(13,958.66; 22,248.78) | 2.55(-7.38; 13.56) | 104.50(74.85; 139.80) | 94.00(73.58; 116.86) | 0.35(-4.30; 5.22) |
| Norway | both | 12,148.69(9,965.63; 14,508.43) | 25,752.48(21,407.85; 29,953.86) | 1.09(-9.10; 12.41) | 276.44(227.24; 330.49) | 466.20(387.62; 544.62) | 0.34(-5.94; 7.05) |
| Thailand | both | 95,065.75(64,177.13; 132,877.40) | 67,034.18(47,528.65; 88,162.62) | -0.26(-11.19; 12.00) | 146.23(100.56; 203.25) | 103.34(72.40; 136.78) | -0.34(-5.07; 4.62) |
| Angola | both | 6,982.04(4,974.07; 9,215.54) | 16,071.16(11,900.02; 20,681.43) | 3.35(-6.56; 14.30) | 80.27(58.31; 105.26) | 60.50(44.59; 77.78) | -0.34(-4.51; 4.02) |
| Singapore | both | 3,583.34(2,455.95; 4,963.80) | 5,618.81(4,018.64; 7,430.74) | 2.03(-6.76; 11.65) | 94.05(64.60; 129.69) | 94.91(66.68; 126.65) | 0.34(-4.31; 5.21) |
| Jamaica | both | 2,721.02(1,844.59; 3,745.44) | 2,826.66(2,071.66; 3,728.06) | 0.58(-7.41; 9.27) | 113.44(78.45; 154.84) | 89.88(65.52; 118.55) | -0.33(-4.89; 4.46) |
| Samoa | both | 164.25(118.83; 218.92) | 161.02(116.79; 208.34) | 0.47(-4.71; 5.93) | 104.58(77.49; 136.26) | 78.49(57.44; 101.01) | -0.33(-4.76; 4.31) |
| Tuvalu | both | 10.06(7.49; 13.12) | 9.52(6.75; 12.58) | 0.91(-1.44; 3.31) | 111.49(83.80; 144.21) | 74.55(53.49; 98.20) | -0.33(-4.71; 4.26) |
| Lao People's Democratic Republic | both | 2,726.65(1,951.53; 3,679.24) | 4,549.39(3,211.98; 6,037.02) | 2.30(-6.29; 11.68) | 74.12(54.06; 98.38) | 56.93(40.92; 74.67) | -0.32(-4.44; 3.97) |
| Benin | both | 2,162.03(1,554.26; 2,900.45) | 4,637.42(3,260.71; 6,228.14) | 4.05(-4.70; 13.61) | 58.57(42.25; 76.98) | 39.58(28.24; 52.56) | 0.31(-3.48; 4.24) |
| Palau | both | 19.24(13.95; 25.63) | 12.84(8.77; 16.74) | -0.50(-3.15; 2.23) | 114.73(84.06; 151.13) | 72.25(48.71; 95.44) | -0.30(-4.66; 4.25) |
| Cook Islands | both | 20.92(14.52; 28.06) | 12.33(8.88; 15.92) | -0.72(-3.29; 1.92) | 111.78(78.21; 148.77) | 71.35(50.48; 91.88) | -0.30(-4.64; 4.24) |
| Romania | both | 20,074.67(16,095.28; 24,830.72) | 16,443.12(13,629.75; 19,271.15) | -1.04(-10.56; 9.50) | 84.33(67.04; 105.19) | 86.52(71.04; 102.34) | -0.28(-4.80; 4.46) |
| Grenada | both | 89.32(63.31; 120.33) | 132.45(101.03; 166.23) | 1.32(-3.75; 6.66) | 111.82(80.56; 148.30) | 119.18(91.04; 149.39) | 0.28(-4.62; 5.42) |
| Kyrgyzstan | both | 6,171.84(4,840.55; 7,783.10) | 11,521.91(9,242.40; 14,181.75) | 1.55(-7.87; 11.94) | 151.87(120.30; 188.53) | 166.43(134.29; 204.05) | -0.27(-5.45; 5.20) |
| Chile | both | 18,498.13(12,526.83; 25,201.19) | 23,927.01(17,769.61; 30,523.25) | 1.32(-8.78; 12.54) | 129.25(87.74; 174.93) | 121.03(89.38; 154.42) | 0.27(-4.62; 5.40) |
| Liberia | both | 1,175.09(822.83; 1,582.66) | 2,120.61(1,467.49; 2,848.20) | 3.71(-4.24; 12.32) | 78.40(55.45; 105.19) | 41.44(29.48; 55.64) | -0.27(-4.08; 3.69) |
| South Sudan | both | 2,675.85(1,851.29; 3,670.80) | 5,026.12(3,608.01; 7,036.91) | 1.63(-6.99; 11.06) | 53.16(37.54; 71.41) | 66.87(48.19; 94.65) | 0.27(-4.03; 4.76) |
| Saint Vincent and the Grenadines | both | 104.71(72.80; 143.01) | 98.17(71.41; 127.80) | -0.03(-4.71; 4.87) | 97.21(68.45; 130.17) | 84.79(61.41; 111.00) | -0.26(-4.77; 4.48) |
| Monaco | both | 42.94(30.47; 56.50) | 40.46(29.54; 51.29) | 0.71(-3.10; 4.67) | 152.36(107.17; 202.86) | 128.07(92.24; 165.49) | 0.26(-4.68; 5.47) |
| Tokelau | both | 1.53(1.13; 2.04) | 0.99(0.71; 1.27) | -0.69(-0.99; -0.39) | 108.42(79.84; 143.42) | 74.47(52.98; 95.36) | -0.26(-4.64; 4.33) |
| Haiti | both | 5,006.07(3,571.85; 6,877.08) | 11,613.00(8,525.28; 15,083.00) | 3.06(-6.50; 13.60) | 85.80(62.04; 115.94) | 85.99(63.57; 110.51) | 0.26(-4.28; 5.01) |
| Colombia | both | 42,859.27(31,550.85; 55,810.78) | 51,814.94(39,787.36; 66,076.79) | 1.18(-9.63; 13.29) | 129.06(96.00; 166.06) | 96.46(73.86; 123.08) | -0.25(-4.89; 4.61) |
| Belize | both | 165.05(114.72; 226.55) | 383.31(273.74; 512.89) | 3.11(-3.08; 9.69) | 94.81(67.39; 127.21) | 80.53(57.42; 107.07) | -0.25(-4.71; 4.42) |
| Cameroon | both | 8,623.80(6,083.04; 11,387.41) | 12,492.22(8,637.13; 16,651.80) | 3.84(-5.88; 14.57) | 103.76(73.08; 135.93) | 43.97(30.73; 57.49) | -0.24(-4.13; 3.81) |
| American Samoa | both | 52.95(38.46; 69.76) | 36.96(26.68; 47.16) | -0.31(-4.01; 3.53) | 110.42(81.45; 144.24) | 78.45(56.67; 99.53) | -0.22(-4.66; 4.43) |
| Honduras | both | 4,013.61(3,042.57; 5,073.70) | 11,129.33(8,389.85; 14,210.07) | 3.45(-6.11; 13.97) | 109.01(85.67; 135.28) | 109.67(83.47; 138.69) | 0.22(-4.56; 5.24) |
| El Salvador | both | 3,674.08(2,596.32; 4,946.13) | 5,475.88(4,188.36; 6,867.80) | 1.32(-7.35; 10.81) | 78.45(56.30; 103.58) | 81.50(62.50; 101.45) | 0.22(-4.26; 4.90) |
| San Marino | both | 38.22(26.77; 51.18) | 34.98(25.20; 45.11) | 0.83(-2.84; 4.65) | 153.61(107.75; 206.64) | 119.60(86.43; 156.97) | 0.21(-4.67; 5.33) |
| Ghana | both | 12,445.50(8,584.31; 16,945.31) | 14,929.99(10,015.38; 19,936.52) | 3.45(-6.40; 14.35) | 99.99(70.01; 134.31) | 44.81(30.18; 59.47) | 0.21(-3.72; 4.30) |
| Georgia | both | 6,600.49(4,733.74; 8,725.53) | 4,181.31(3,140.65; 5,262.28) | -1.32(-9.54; 7.65) | 114.56(82.57; 151.62) | 119.48(87.84; 151.18) | 0.20(-4.67; 5.32) |
| Vanuatu | both | 122.36(87.44; 165.47) | 206.60(144.98; 271.31) | 2.47(-3.06; 8.31) | 89.11(65.56; 119.15) | 66.53(47.13; 86.28) | -0.20(-4.47; 4.27) |
| Mexico | both | 92,407.03(67,509.93; 121,235.74) | 119,551.15(91,663.69; 149,850.47) | 1.37(-10.25; 14.49) | 111.92(82.52; 144.72) | 86.88(66.56; 108.77) | -0.19(-4.73; 4.56) |
| Burundi | both | 2,442.08(1,720.76; 3,314.55) | 7,868.22(5,638.83; 10,623.13) | 3.46(-5.75; 13.58) | 53.72(38.77; 71.11) | 71.49(50.65; 96.40) | 0.19(-4.17; 4.75) |
| Russian Federation | both | 631,275.39(502,326.76; 778,747.88) | 659,538.54(561,314.81; 757,397.06) | -0.30(-13.31; 14.67) | 400.23(317.40; 494.71) | 456.02(385.23; 526.69) | -0.18(-6.38; 6.43) |
| Seychelles | both | 102.33(73.30; 136.34) | 111.45(86.52; 135.95) | 1.11(-3.78; 6.25) | 134.16(97.79; 176.33) | 100.58(76.69; 124.08) | -0.18(-4.91; 4.79) |
| Antigua and Barbuda | both | 63.81(44.43; 85.93) | 86.32(64.25; 109.92) | 1.22(-3.42; 6.08) | 101.02(70.88; 134.82) | 89.05(66.09; 113.69) | -0.18(-4.77; 4.63) |
| Japan | both | 122,546.79(88,791.74; 161,754.90) | 94,571.85(70,378.15; 120,981.43) | -0.40(-11.61; 12.23) | 95.21(68.22; 127.27) | 85.30(62.08; 111.73) | 0.18(-4.36; 4.93) |
| Palestine | both | 1,642.51(1,118.35; 2,289.17) | 4,534.82(3,019.45; 6,147.71) | 3.93(-4.79; 13.46) | 96.25(66.59; 130.44) | 84.69(56.46; 114.03) | 0.18(-4.35; 4.93) |
| Bahrain | both | 656.51(447.70; 896.72) | 1,784.83(1,282.59; 2,292.82) | 4.47(-3.38; 12.96) | 110.28(76.68; 145.83) | 93.48(67.05; 120.03) | -0.17(-4.78; 4.66) |
| Argentina | both | 37,666.18(25,644.15; 52,274.68) | 50,326.01(35,767.03; 66,734.95) | 1.49(-9.33; 13.60) | 117.16(79.71; 162.33) | 105.17(74.47; 140.02) | 0.16(-4.58; 5.14) |
| Democratic Republic of the Congo | both | 22,636.04(16,414.11; 29,982.88) | 46,199.27(33,346.39; 60,512.05) | 2.94(-7.94; 15.11) | 72.31(53.21; 93.88) | 58.25(42.28; 75.62) | -0.16(-4.30; 4.16) |
| Kenya | both | 10,502.21(7,326.62; 14,339.39) | 21,683.09(16,466.06; 26,836.87) | 3.36(-6.84; 14.68) | 58.62(41.02; 78.58) | 48.60(37.50; 58.62) | 0.16(-3.82; 4.30) |
| Iraq | both | 20,623.63(15,780.35; 26,197.76) | 66,710.83(52,492.02; 84,974.83) | 3.29(-7.99; 15.96) | 143.41(110.89; 180.46) | 159.03(125.92; 202.94) | -0.15(-5.30; 5.27) |
| Zambia | both | 5,699.30(4,031.53; 7,699.74) | 14,882.00(10,778.18; 19,886.40) | 3.16(-6.65; 14.01) | 90.50(64.71; 118.87) | 90.76(66.66; 120.63) | -0.15(-4.74; 4.65) |
| Tajikistan | both | 4,173.45(2,930.53; 5,634.34) | 10,834.25(7,787.53; 13,768.03) | 2.87(-6.62; 13.33) | 86.78(62.39; 115.71) | 103.68(75.10; 130.88) | 0.15(-4.58; 5.12) |
| Portugal | both | 11,521.62(8,508.10; 15,177.42) | 13,840.38(11,179.16; 16,538.41) | -0.08(-9.54; 10.37) | 112.36(82.81; 148.66) | 138.20(110.96; 165.59) | 0.11(-4.91; 5.40) |
| Cambodia | both | 6,972.10(4,802.20; 9,653.48) | 11,135.35(7,753.82; 15,060.49) | 2.30(-7.17; 12.72) | 74.86(52.88; 101.18) | 60.94(43.08; 81.77) | -0.11(-4.30; 4.26) |
| Niger | both | 3,677.23(2,571.57; 4,974.86) | 7,311.48(5,087.03; 10,038.45) | 3.47(-5.67; 13.51) | 61.33(43.37; 81.66) | 38.58(26.92; 51.90) | -0.11(-3.85; 3.77) |
| Malaysia | both | 20,047.05(14,037.17; 27,098.36) | 24,479.33(17,277.75; 32,258.86) | 2.25(-7.97; 13.60) | 113.03(80.48; 151.04) | 67.79(48.14; 88.82) | -0.10(-4.40; 4.39) |
| Guinea-Bissau | both | 493.00(355.10; 645.32) | 684.09(474.91; 893.11) | 2.71(-4.04; 9.94) | 61.99(45.15; 80.07) | 36.97(26.06; 48.15) | -0.10(-3.80; 3.74) |
| Papua New Guinea | both | 3,159.54(2,222.84; 4,233.45) | 6,965.66(4,861.90; 9,321.23) | 3.38(-5.72; 13.35) | 79.08(57.32; 104.33) | 65.57(46.23; 87.11) | 0.09(-4.18; 4.55) |
| Nepal | both | 11,753.01(7,807.39; 16,731.33) | 20,468.72(15,064.43; 26,465.41) | 2.18(-7.85; 13.31) | 68.89(46.57; 96.16) | 63.95(47.60; 81.63) | 0.09(-4.16; 4.52) |
| Saint Lucia | both | 145.14(102.72; 196.76) | 166.59(120.40; 212.53) | 1.02(-4.27; 6.60) | 105.87(75.66; 141.95) | 87.99(62.82; 112.50) | -0.08(-4.67; 4.74) |
| Guinea | both | 2,538.36(1,804.10; 3,412.07) | 4,055.41(2,884.34; 5,363.52) | 2.67(-5.83; 11.94) | 51.41(36.73; 68.40) | 35.54(25.19; 46.40) | 0.08(-3.59; 3.88) |
| Belarus | both | 26,944.48(20,191.09; 35,024.14) | 24,556.30(19,974.27; 29,342.59) | -0.19(-10.17; 10.90) | 246.96(184.46; 322.99) | 249.96(203.26; 299.17) | 0.06(-5.53; 5.98) |
| Guatemala | both | 8,706.94(6,890.59; 10,842.79) | 20,305.95(16,931.04; 23,914.76) | 3.58(-6.59; 14.84) | 135.28(108.53; 166.42) | 124.14(104.01; 145.53) | -0.06(-4.98; 5.11) |
| Poland | both | 49,962.37(37,535.88; 66,231.77) | 41,146.13(32,491.35; 50,033.59) | -0.06(-10.53; 11.65) | 132.39(98.40; 176.84) | 115.61(90.21; 142.29) | -0.04(-4.86; 5.02) |
| Timor-Leste | both | 627.12(420.58; 876.36) | 886.73(602.48; 1,221.56) | 1.92(-5.03; 9.38) | 81.33(55.92; 112.00) | 63.34(44.08; 85.88) | 0.04(-4.20; 4.46) |
| Cabo Verde | both | 289.48(201.92; 397.04) | 279.72(196.75; 365.50) | 2.77(-3.11; 9.01) | 101.85(72.24; 136.10) | 44.78(31.60; 58.30) | 0.03(-3.89; 4.11) |
| Australia | both | 79,559.23(62,590.49; 98,922.53) | 131,560.64(111,227.23; 151,449.22) | 1.20(-10.49; 14.42) | 443.22(347.71; 551.03) | 502.09(420.60; 582.79) | 0.02(-6.27; 6.72) |
| Lesotho | both | 2,221.43(1,552.45; 3,025.71) | 1,732.15(1,290.72; 2,209.87) | 0.79(-6.75; 8.94) | 139.08(97.82; 187.51) | 96.68(73.24; 122.71) | -0.02(-4.68; 4.86) |
| Guyana | both | 679.03(477.87; 914.52) | 602.90(435.33; 781.16) | 0.05(-6.41; 6.96) | 86.03(61.01; 113.72) | 73.59(53.67; 95.06) | -0.01(-4.39; 4.58) |
| Tonga | both | 87.76(61.66; 120.11) | 72.20(51.42; 95.27) | 0.33(-4.05; 4.91) | 100.95(71.38; 135.56) | 72.51(51.89; 95.98) | -0.01(-4.38; 4.56) |
| Equatorial Guinea | both | 309.84(221.32; 415.58) | 1,005.40(741.03; 1,306.54) | 5.02(-2.25; 12.84) | 92.00(65.88; 121.34) | 68.49(50.91; 89.21) | -0.01(-4.31; 4.50) |
| Dominica | both | 80.55(57.80; 109.70) | 77.72(59.03; 98.54) | -0.06(-4.51; 4.59) | 109.17(78.81; 146.91) | 110.45(83.99; 140.26) | 0.00(-4.80; 5.05) |
| Republic of Korea | both | 50,396.41(34,862.09; 69,429.62) | 51,828.39(36,683.98; 67,414.45) | 0.10(-10.61; 12.09) | 97.55(68.06; 132.63) | 99.31(69.68; 130.25) | 0.00(-4.68; 4.91) |
| India | both | 725,161.05(529,674.97; 957,956.00) | 1,229,324.19(963,582.06; 1,506,573.26) | 2.19(-11.70; 18.25) | 92.07(68.35; 119.09) | 81.52(64.47; 98.94) | 0.00(-4.48; 4.69) |

**Supplementary Table 2 DALY of Drug use disorders in 204 countries in 2021, by countries and ages.**

|  | 15-19 years | 20-24 years | 25-29 years | 30-34 years | 35-39 years | 40-44 years | 45-49 years | 50-54 years | 55-59 years | 60-64 years | 65-69 years | 70-74 years | 75-79 years | 80-84 | 85-89 | 90-94 | 95+ years |
| --- | --- | --- | --- | --- | --- | --- | --- | --- | --- | --- | --- | --- | --- | --- | --- | --- | --- |
| Afghanistan | 161.73 | 434.53 | 481.56 | 392.36 | 340.42 | 316.37 | 273.58 | 232.93 | 180.93 | 141.94 | 114.86 | 96.94 | 87.22 | 87.23 | 82.69 | 86.53 | 92.69 |
| Albania | 119.53 | 359.11 | 355.18 | 259.63 | 181.76 | 128.51 | 104.09 | 91.50 | 76.52 | 59.48 | 58.03 | 61.04 | 62.23 | 54.68 | 58.40 | 75.63 | 92.30 |
| Algeria | 123.62 | 369.04 | 420.97 | 346.40 | 297.62 | 254.73 | 206.43 | 161.19 | 121.34 | 95.24 | 72.87 | 62.91 | 63.06 | 68.83 | 113.43 | 142.34 | 207.59 |
| American Samoa | 94.15 | 229.76 | 227.44 | 132.46 | 95.47 | 88.04 | 66.65 | 60.33 | 40.85 | 42.21 | 32.78 | 54.56 | 35.83 | 39.56 | 26.23 | 28.67 | 35.02 |
| Andorra | 141.05 | 285.56 | 284.05 | 210.32 | 159.23 | 124.41 | 96.82 | 73.34 | 52.06 | 37.14 | 30.38 | 26.71 | 25.17 | 25.12 | 26.58 | 28.80 | 32.41 |
| Angola | 40.89 | 122.91 | 138.28 | 133.46 | 114.61 | 98.98 | 70.89 | 57.83 | 49.36 | 41.44 | 36.92 | 33.38 | 28.65 | 25.92 | 25.62 | 25.77 | 23.16 |
| Antigua and Barbuda | 127.01 | 228.01 | 222.75 | 173.70 | 139.48 | 109.31 | 89.94 | 68.57 | 53.08 | 39.68 | 30.66 | 23.60 | 22.03 | 21.47 | 19.31 | 18.39 | 18.13 |
| Argentina | 150.09 | 261.82 | 259.92 | 208.01 | 161.81 | 123.73 | 100.52 | 83.68 | 67.77 | 55.36 | 47.04 | 40.91 | 35.88 | 33.65 | 32.92 | 36.18 | 38.51 |
| Armenia | 90.02 | 303.40 | 332.26 | 241.41 | 171.53 | 126.84 | 105.18 | 93.43 | 78.08 | 60.12 | 48.15 | 38.64 | 37.60 | 36.93 | 36.33 | 37.31 | 47.26 |
| Australia | 348.24 | 928.82 | 1,135.00 | 1,111.20 | 1,117.97 | 983.34 | 773.15 | 525.10 | 315.35 | 181.16 | 104.21 | 70.59 | 64.61 | 58.58 | 68.57 | 59.37 | 130.46 |
| Austria | 290.96 | 694.14 | 703.54 | 557.14 | 396.24 | 284.32 | 230.86 | 163.01 | 123.21 | 79.10 | 61.30 | 53.15 | 54.78 | 55.96 | 81.10 | 97.66 | 117.94 |
| Azerbaijan | 86.47 | 285.16 | 314.35 | 229.95 | 157.61 | 114.83 | 94.36 | 82.80 | 70.57 | 55.79 | 44.46 | 37.49 | 34.68 | 34.68 | 30.66 | 31.41 | 37.40 |
| Bahrain | 138.25 | 233.62 | 225.40 | 184.36 | 151.38 | 116.25 | 89.38 | 66.29 | 48.60 | 35.27 | 26.42 | 20.49 | 18.43 | 18.03 | 16.61 | 16.19 | 15.53 |
| Bangladesh | 73.11 | 246.78 | 265.25 | 195.04 | 145.95 | 115.98 | 96.06 | 77.58 | 61.30 | 46.46 | 36.59 | 32.95 | 31.77 | 33.33 | 35.30 | 39.59 | 51.73 |
| Barbados | 48.91 | 144.66 | 145.52 | 114.31 | 89.99 | 78.98 | 76.74 | 72.16 | 77.58 | 61.12 | 66.58 | 53.89 | 50.49 | 50.09 | 51.45 | 62.55 | 74.58 |
| Belarus | 141.30 | 233.66 | 225.15 | 180.15 | 137.33 | 104.36 | 81.92 | 60.77 | 47.47 | 33.78 | 25.75 | 19.53 | 17.66 | 16.50 | 15.65 | 15.58 | 14.78 |
| Belgium | 173.24 | 492.04 | 596.58 | 550.88 | 446.62 | 364.79 | 308.03 | 282.11 | 237.33 | 186.91 | 135.13 | 94.79 | 99.82 | 124.26 | 125.65 | 137.37 | 156.11 |
| Belize | 219.47 | 456.46 | 524.87 | 468.23 | 411.07 | 323.94 | 250.57 | 171.10 | 125.89 | 86.92 | 74.17 | 67.02 | 72.09 | 83.54 | 110.43 | 135.84 | 168.54 |
| Benin | 130.06 | 209.30 | 203.63 | 156.50 | 124.60 | 95.15 | 73.89 | 55.12 | 42.60 | 31.13 | 23.10 | 18.66 | 16.73 | 15.23 | 14.59 | 14.50 | 14.31 |
| Bermuda | 31.63 | 97.62 | 101.27 | 78.98 | 61.16 | 49.44 | 41.77 | 36.51 | 30.52 | 28.17 | 26.65 | 22.83 | 21.49 | 21.11 | 21.92 | 21.07 | 20.22 |
| Bhutan | 139.59 | 284.78 | 294.36 | 249.87 | 232.44 | 193.26 | 163.15 | 112.61 | 84.35 | 56.68 | 42.69 | 34.41 | 36.85 | 43.45 | 42.32 | 47.11 | 45.71 |
| Bolivia (Plurinational State of) | 45.15 | 145.39 | 148.20 | 118.04 | 91.02 | 81.29 | 77.86 | 71.16 | 76.28 | 59.96 | 69.68 | 59.64 | 58.52 | 50.85 | 55.24 | 69.61 | 77.24 |
| Bosnia and Herzegovina | 139.66 | 249.88 | 240.15 | 192.61 | 146.96 | 116.88 | 95.96 | 79.70 | 70.67 | 64.77 | 55.61 | 44.93 | 40.36 | 32.57 | 23.29 | 24.42 | 20.08 |
| Botswana | 55.87 | 170.09 | 187.41 | 140.15 | 99.65 | 66.28 | 50.95 | 42.25 | 34.29 | 26.56 | 21.98 | 20.08 | 21.04 | 22.09 | 21.26 | 19.96 | 19.21 |
| Brazil | 62.31 | 179.68 | 201.06 | 163.58 | 123.23 | 107.32 | 109.35 | 118.38 | 111.59 | 91.90 | 84.32 | 68.92 | 61.46 | 64.64 | 64.95 | 69.30 | 157.77 |
| Brunei Darussalam | 195.21 | 358.60 | 350.42 | 262.30 | 196.82 | 146.45 | 115.39 | 85.24 | 66.14 | 52.80 | 43.83 | 34.99 | 30.98 | 28.31 | 29.06 | 30.53 | 31.94 |
| Bulgaria | 118.80 | 279.06 | 280.29 | 233.25 | 186.86 | 142.87 | 118.20 | 96.07 | 75.04 | 59.81 | 50.61 | 44.57 | 45.55 | 43.82 | 40.20 | 35.36 | 42.99 |
| Burkina Faso | 157.72 | 392.74 | 381.09 | 257.61 | 179.37 | 124.51 | 100.20 | 85.56 | 70.75 | 51.97 | 43.35 | 37.82 | 33.82 | 31.24 | 35.17 | 45.33 | 54.89 |
| Burundi | 23.77 | 87.63 | 96.14 | 78.80 | 64.11 | 51.86 | 42.12 | 35.08 | 30.50 | 29.45 | 31.39 | 30.49 | 29.24 | 28.19 | 28.14 | 27.20 | 22.24 |
| Cambodia | 34.68 | 82.30 | 100.38 | 207.06 | 201.81 | 243.90 | 40.42 | 32.19 | 28.98 | 24.51 | 24.65 | 24.61 | 26.88 | 227.65 | 33.08 | 27.91 | 27.56 |
| Cameroon | 29.89 | 118.01 | 113.69 | 88.80 | 69.56 | 57.23 | 48.77 | 43.37 | 37.07 | 31.12 | 27.13 | 22.88 | 20.57 | 19.92 | 19.56 | 20.32 | 18.50 |
| Canada | 54.43 | 185.86 | 185.87 | 120.70 | 82.59 | 60.34 | 47.55 | 39.76 | 35.35 | 30.03 | 27.92 | 24.90 | 24.46 | 24.01 | 27.49 | 28.20 | 38.67 |
| Cabo Verde | 29.09 | 106.27 | 113.55 | 87.45 | 67.95 | 55.66 | 47.20 | 41.53 | 35.99 | 34.32 | 34.18 | 29.79 | 27.17 | 26.11 | 26.48 | 25.24 | 21.99 |
| Central African Republic | 619.76 | 1,796.54 | 2,267.95 | 2,052.71 | 1,699.83 | 1,381.50 | 1,148.05 | 916.77 | 641.20 | 352.10 | 193.77 | 113.82 | 84.34 | 78.79 | 86.32 | 107.70 | 144.95 |
| Chad | 40.16 | 115.08 | 124.46 | 116.20 | 100.51 | 87.63 | 64.99 | 54.31 | 46.47 | 38.41 | 32.19 | 26.83 | 22.24 | 20.65 | 19.43 | 19.60 | 18.59 |
| Chile | 25.84 | 91.03 | 97.25 | 75.65 | 59.53 | 46.79 | 39.56 | 34.22 | 29.34 | 27.40 | 26.72 | 23.08 | 21.52 | 20.99 | 20.68 | 19.50 | 18.62 |
| China | 194.20 | 318.89 | 298.80 | 229.38 | 174.39 | 135.02 | 109.23 | 91.60 | 70.49 | 57.10 | 48.51 | 42.74 | 38.06 | 37.06 | 39.33 | 53.78 | 71.44 |
| Colombia | 84.57 | 308.14 | 332.09 | 246.71 | 187.42 | 146.12 | 108.47 | 89.48 | 72.54 | 62.56 | 56.66 | 49.23 | 47.72 | 45.11 | 46.08 | 43.56 | 31.17 |
| Comoros | 158.77 | 253.18 | 244.03 | 189.33 | 145.54 | 107.86 | 85.20 | 65.16 | 51.78 | 40.07 | 33.16 | 24.85 | 22.43 | 20.01 | 18.09 | 18.40 | 18.26 |
| Congo | 34.93 | 82.51 | 107.45 | 253.59 | 248.06 | 305.34 | 43.05 | 32.91 | 29.62 | 24.94 | 24.26 | 25.10 | 28.99 | 248.56 | 39.33 | 32.75 | 33.35 |
| Costa Rica | 43.51 | 129.30 | 147.32 | 144.72 | 125.67 | 111.13 | 80.85 | 66.89 | 56.87 | 47.64 | 40.51 | 36.77 | 30.98 | 28.62 | 29.04 | 31.24 | 31.09 |
| Côte d'Ivoire | 84.62 | 203.00 | 205.27 | 126.42 | 91.31 | 81.88 | 62.65 | 55.70 | 40.16 | 36.57 | 28.66 | 45.06 | 24.30 | 23.18 | 21.55 | 26.26 | 28.17 |
| Croatia | 101.67 | 208.85 | 217.29 | 173.94 | 141.96 | 112.82 | 90.81 | 69.63 | 56.09 | 42.81 | 36.13 | 24.12 | 22.35 | 19.81 | 17.38 | 17.74 | 19.49 |
| Cuba | 169.76 | 423.88 | 480.17 | 423.86 | 331.18 | 213.03 | 162.90 | 127.54 | 102.09 | 73.44 | 62.43 | 57.46 | 57.90 | 64.56 | 113.96 | 189.53 | 205.36 |
| Cyprus | 113.54 | 205.88 | 201.47 | 157.05 | 120.91 | 91.60 | 72.56 | 56.03 | 42.92 | 31.70 | 23.62 | 18.96 | 16.36 | 15.75 | 14.44 | 14.57 | 14.37 |
| Czechia | 127.07 | 431.61 | 462.60 | 311.02 | 250.44 | 181.32 | 149.90 | 97.64 | 73.33 | 48.83 | 39.07 | 36.69 | 39.77 | 49.80 | 64.53 | 104.05 | 133.69 |
| Democratic Republic of the Congo | 177.22 | 407.08 | 396.62 | 286.39 | 203.23 | 145.40 | 119.06 | 105.32 | 84.89 | 65.59 | 54.20 | 46.75 | 44.57 | 44.86 | 57.49 | 69.51 | 74.81 |
| Denmark | 26.65 | 95.07 | 99.55 | 77.97 | 60.66 | 48.45 | 41.03 | 35.72 | 30.70 | 29.00 | 29.22 | 24.97 | 23.32 | 23.21 | 22.44 | 21.19 | 19.82 |
| Djibouti | 86.67 | 303.74 | 321.93 | 230.38 | 175.17 | 136.78 | 108.88 | 91.27 | 76.97 | 65.43 | 61.98 | 52.85 | 49.31 | 42.81 | 36.60 | 32.52 | 29.14 |
| Dominica | 39.83 | 118.94 | 132.72 | 129.35 | 109.18 | 95.58 | 67.72 | 57.00 | 48.50 | 40.33 | 34.73 | 30.03 | 25.16 | 23.16 | 21.86 | 22.64 | 20.61 |
| Dominican Republic | 286.98 | 811.20 | 816.78 | 734.23 | 746.75 | 629.45 | 602.47 | 436.41 | 317.72 | 181.21 | 126.39 | 96.26 | 95.89 | 111.38 | 161.04 | 207.91 | 220.24 |
| Ecuador | 36.17 | 87.27 | 101.52 | 255.93 | 243.81 | 311.48 | 44.74 | 33.96 | 30.48 | 27.15 | 27.35 | 28.10 | 30.69 | 280.54 | 39.28 | 33.28 | 32.57 |
| Egypt | 179.31 | 277.55 | 258.44 | 211.20 | 178.05 | 143.55 | 114.40 | 78.24 | 59.72 | 42.62 | 34.77 | 28.32 | 28.81 | 30.18 | 27.00 | 25.97 | 22.02 |
| El Salvador | 94.29 | 179.73 | 178.74 | 141.15 | 111.35 | 84.45 | 66.73 | 51.98 | 39.61 | 29.62 | 22.71 | 18.27 | 16.22 | 15.24 | 14.12 | 13.80 | 14.69 |
| Equatorial Guinea | 109.54 | 234.56 | 234.30 | 188.87 | 144.93 | 113.16 | 92.60 | 78.20 | 69.20 | 65.95 | 51.93 | 39.60 | 36.32 | 29.30 | 21.76 | 26.28 | 28.71 |
| Eritrea | 72.74 | 260.03 | 275.26 | 192.00 | 146.86 | 127.94 | 113.35 | 95.98 | 77.54 | 60.58 | 45.50 | 35.31 | 31.35 | 32.14 | 30.09 | 24.00 | 28.05 |
| Estonia | 83.37 | 189.76 | 208.44 | 170.56 | 145.12 | 113.21 | 89.60 | 68.00 | 54.18 | 41.49 | 34.38 | 24.04 | 20.83 | 19.02 | 16.97 | 17.43 | 18.68 |
| Ethiopia | 44.90 | 138.49 | 157.17 | 157.12 | 131.94 | 111.99 | 78.72 | 63.91 | 53.82 | 44.75 | 39.48 | 35.01 | 31.17 | 30.61 | 30.14 | 30.52 | 27.89 |
| Micronesia (Federated States of) | 35.36 | 85.92 | 106.24 | 265.85 | 256.13 | 318.44 | 46.34 | 33.50 | 30.94 | 26.03 | 24.74 | 23.98 | 29.90 | 197.79 | 39.89 | 32.26 | 32.37 |
| Fiji | 559.26 | 1,856.20 | 2,331.26 | 1,952.86 | 1,387.71 | 774.98 | 497.38 | 395.74 | 317.54 | 236.90 | 167.36 | 118.06 | 91.00 | 65.33 | 61.52 | 77.31 | 114.03 |
| Finland | 82.04 | 196.14 | 216.51 | 176.37 | 137.73 | 126.91 | 131.86 | 139.47 | 135.66 | 112.35 | 105.25 | 82.91 | 72.53 | 75.90 | 76.42 | 61.43 | 111.72 |
| France | 29.25 | 75.46 | 93.07 | 111.42 | 98.04 | 105.37 | 39.86 | 30.72 | 28.34 | 24.50 | 24.37 | 24.95 | 27.36 | 81.64 | 30.54 | 29.33 | 25.40 |
| Gabon | 80.65 | 192.46 | 185.29 | 114.89 | 79.34 | 63.92 | 51.29 | 45.91 | 35.05 | 30.90 | 26.69 | 33.44 | 23.69 | 24.92 | 22.29 | 22.74 | 29.39 |
| Georgia | 438.03 | 1,275.29 | 1,325.43 | 1,147.68 | 926.35 | 733.77 | 576.09 | 453.77 | 344.92 | 217.94 | 150.77 | 110.94 | 93.48 | 104.24 | 145.73 | 182.30 | 228.99 |
| Germany | 186.01 | 393.42 | 441.71 | 399.99 | 355.24 | 296.60 | 250.50 | 189.45 | 149.18 | 111.60 | 103.57 | 115.51 | 155.31 | 222.57 | 358.83 | 554.37 | 629.43 |
| Ghana | 43.11 | 133.61 | 152.97 | 156.25 | 137.75 | 121.47 | 85.85 | 69.39 | 58.74 | 50.33 | 45.26 | 40.65 | 32.85 | 29.23 | 27.14 | 27.23 | 26.21 |
| Greece | 27.27 | 101.42 | 103.99 | 81.31 | 63.92 | 50.18 | 42.45 | 37.08 | 31.71 | 29.97 | 28.71 | 25.48 | 24.13 | 23.30 | 22.97 | 22.32 | 20.50 |
| Greenland | 89.06 | 307.90 | 336.34 | 250.04 | 185.35 | 143.58 | 119.62 | 106.50 | 89.78 | 70.16 | 53.96 | 48.08 | 46.97 | 47.55 | 40.57 | 36.87 | 39.28 |
| Grenada | 210.52 | 473.09 | 552.37 | 530.16 | 498.16 | 397.00 | 305.96 | 203.14 | 138.81 | 83.57 | 62.76 | 51.67 | 51.47 | 53.09 | 65.95 | 95.95 | 123.06 |
| Guam | 32.74 | 104.82 | 121.02 | 90.88 | 69.85 | 57.53 | 48.02 | 41.62 | 38.28 | 31.93 | 28.39 | 24.20 | 20.96 | 19.56 | 21.23 | 21.51 | 24.77 |
| Guatemala | 180.13 | 565.88 | 700.39 | 668.39 | 556.10 | 365.70 | 262.81 | 160.18 | 112.99 | 67.26 | 53.80 | 46.00 | 45.60 | 52.57 | 68.75 | 89.18 | 105.80 |
| Guinea | 282.37 | 659.77 | 763.81 | 687.09 | 635.67 | 556.65 | 506.98 | 402.03 | 274.86 | 155.22 | 90.69 | 57.53 | 42.49 | 38.31 | 35.87 | 34.60 | 47.48 |
| Guinea-Bissau | 151.44 | 262.16 | 279.12 | 246.43 | 228.65 | 170.33 | 140.37 | 94.82 | 75.17 | 50.00 | 37.15 | 29.39 | 30.71 | 27.69 | 20.97 | 20.32 | 17.90 |
| Guyana | 85.00 | 217.79 | 213.25 | 130.12 | 89.81 | 76.42 | 62.48 | 52.56 | 40.36 | 40.59 | 32.25 | 49.21 | 31.14 | 30.24 | 21.40 | 19.25 | 21.91 |
| Haiti | 142.44 | 272.32 | 283.04 | 234.92 | 217.66 | 180.37 | 161.05 | 125.26 | 102.22 | 75.04 | 65.69 | 40.37 | 35.87 | 30.14 | 26.95 | 33.34 | 43.82 |
| Honduras | 24.76 | 86.88 | 91.19 | 71.27 | 55.99 | 44.62 | 37.25 | 32.57 | 28.21 | 26.08 | 26.56 | 23.20 | 21.84 | 22.08 | 21.28 | 20.71 | 19.56 |
| Hungary | 25.76 | 91.50 | 94.44 | 74.29 | 57.55 | 46.26 | 38.84 | 34.22 | 29.83 | 27.62 | 27.14 | 23.94 | 21.62 | 21.14 | 20.96 | 20.36 | 18.73 |
| Iceland | 99.53 | 188.80 | 187.90 | 149.73 | 121.42 | 91.70 | 71.83 | 52.27 | 40.52 | 29.09 | 22.84 | 18.39 | 16.61 | 15.91 | 14.48 | 15.15 | 14.81 |
| India | 122.36 | 212.95 | 209.55 | 171.21 | 144.39 | 112.30 | 89.41 | 63.14 | 49.48 | 35.41 | 27.85 | 22.17 | 20.91 | 20.08 | 17.81 | 17.02 | 15.78 |
| Indonesia | 107.81 | 216.15 | 232.36 | 194.27 | 173.76 | 157.65 | 145.79 | 133.09 | 120.01 | 108.13 | 104.11 | 68.97 | 57.34 | 45.32 | 33.94 | 33.61 | 49.86 |
| Iran (Islamic Republic of) | 116.02 | 330.64 | 338.79 | 229.36 | 154.21 | 104.52 | 81.77 | 70.07 | 57.41 | 43.62 | 35.22 | 29.43 | 26.46 | 24.26 | 25.93 | 28.38 | 33.38 |
| Iraq | 365.69 | 1,109.76 | 1,704.53 | 1,267.79 | 766.07 | 708.56 | 497.28 | 278.62 | 266.02 | 168.42 | 144.06 | 141.28 | 172.76 | 206.54 | 325.99 | 451.57 | 456.64 |
| Ireland | 53.20 | 169.15 | 179.09 | 150.66 | 117.52 | 110.79 | 104.64 | 94.14 | 104.46 | 81.19 | 88.51 | 72.24 | 65.75 | 54.76 | 54.15 | 68.24 | 81.75 |
| Israel | 47.32 | 147.55 | 141.48 | 91.85 | 63.96 | 41.73 | 29.09 | 22.59 | 22.08 | 26.52 | 31.47 | 27.92 | 34.36 | 31.96 | 36.70 | 38.65 | 56.34 |
| Italy | 271.24 | 817.01 | 926.04 | 734.44 | 601.95 | 483.47 | 374.25 | 276.45 | 203.93 | 152.06 | 118.13 | 97.57 | 97.54 | 105.64 | 102.81 | 114.13 | 111.63 |
| Jamaica | 118.03 | 328.78 | 371.58 | 311.19 | 280.90 | 260.17 | 221.24 | 178.58 | 134.68 | 102.80 | 81.33 | 70.27 | 68.72 | 73.53 | 74.61 | 88.25 | 122.99 |
| Japan | 341.97 | 880.35 | 1,106.99 | 974.32 | 803.40 | 611.02 | 479.02 | 345.12 | 257.16 | 151.93 | 107.80 | 87.19 | 84.03 | 94.87 | 125.07 | 156.17 | 188.86 |
| Jordan | 153.25 | 383.43 | 396.50 | 332.03 | 255.47 | 203.60 | 168.93 | 123.02 | 93.46 | 60.96 | 48.94 | 43.30 | 46.39 | 56.34 | 80.14 | 109.01 | 127.55 |
| Kazakhstan | 143.42 | 361.62 | 388.70 | 299.77 | 232.41 | 176.73 | 135.86 | 93.62 | 65.49 | 46.76 | 39.43 | 35.56 | 35.74 | 39.31 | 50.00 | 64.70 | 82.15 |
| Kenya | 158.18 | 234.27 | 217.29 | 171.91 | 134.99 | 102.03 | 82.11 | 62.73 | 48.62 | 35.82 | 28.24 | 23.01 | 20.46 | 18.86 | 17.29 | 17.41 | 16.88 |
| Kiribati | 85.14 | 229.90 | 235.52 | 179.99 | 133.92 | 102.82 | 81.03 | 65.37 | 51.49 | 39.29 | 30.63 | 24.36 | 21.42 | 19.70 | 19.46 | 20.32 | 21.28 |
| Kuwait | 71.34 | 249.81 | 268.03 | 192.74 | 140.09 | 108.49 | 87.88 | 69.21 | 53.68 | 40.70 | 30.63 | 25.49 | 21.73 | 21.81 | 24.82 | 26.61 | 30.48 |
| Kyrgyzstan | 151.36 | 539.44 | 775.97 | 840.22 | 703.87 | 480.03 | 332.62 | 268.80 | 225.56 | 181.39 | 143.02 | 133.05 | 146.16 | 174.26 | 184.08 | 187.86 | 240.02 |
| Lao People's Democratic Republic | 23.32 | 65.19 | 81.64 | 118.69 | 113.95 | 136.58 | 37.83 | 29.42 | 27.88 | 25.21 | 25.76 | 25.66 | 30.54 | 222.14 | 48.13 | 58.01 | 71.05 |
| Latvia | 465.52 | 845.57 | 947.68 | 412.37 | 383.30 | 519.00 | 331.10 | 315.84 | 110.29 | 176.12 | 51.71 | 280.26 | 106.98 | 192.42 | 70.57 | 104.73 | 146.38 |
| Lebanon | 93.85 | 306.81 | 350.33 | 268.56 | 203.69 | 170.04 | 142.45 | 121.44 | 92.97 | 72.32 | 58.61 | 54.73 | 55.05 | 67.30 | 86.52 | 97.58 | 134.84 |
| Lesotho | 109.66 | 358.72 | 435.59 | 380.05 | 289.96 | 220.72 | 180.42 | 159.63 | 141.15 | 116.31 | 89.22 | 71.24 | 68.85 | 67.86 | 62.54 | 65.65 | 106.42 |
| Liberia | 58.54 | 164.31 | 165.72 | 111.24 | 80.41 | 59.83 | 46.63 | 38.39 | 33.62 | 28.94 | 26.51 | 23.56 | 23.07 | 22.58 | 25.57 | 25.97 | 35.22 |
| Libya | 199.13 | 680.90 | 741.65 | 607.31 | 471.96 | 331.63 | 269.24 | 253.13 | 206.97 | 155.19 | 112.95 | 82.16 | 62.16 | 48.12 | 42.70 | 50.50 | 67.50 |
| Lithuania | 109.57 | 376.12 | 404.86 | 288.92 | 215.79 | 181.26 | 148.15 | 116.79 | 89.10 | 69.44 | 56.25 | 47.29 | 46.37 | 55.34 | 57.96 | 69.04 | 98.09 |
| Luxembourg | 73.02 | 181.26 | 200.92 | 162.71 | 127.47 | 117.88 | 130.51 | 146.84 | 146.44 | 115.85 | 108.26 | 88.78 | 77.50 | 83.20 | 85.87 | 67.22 | 108.61 |
| North Macedonia | 33.96 | 103.86 | 106.87 | 83.09 | 64.23 | 50.16 | 41.97 | 36.86 | 31.73 | 28.78 | 27.67 | 23.59 | 21.78 | 22.31 | 23.18 | 22.54 | 21.23 |
| Madagascar | 159.55 | 471.51 | 549.41 | 507.31 | 485.95 | 455.93 | 389.04 | 296.36 | 216.60 | 165.50 | 126.87 | 104.26 | 99.30 | 97.72 | 98.27 | 110.30 | 148.46 |
| Malawi | 224.24 | 765.64 | 1,010.14 | 1,109.64 | 886.90 | 546.82 | 360.95 | 275.81 | 211.31 | 157.26 | 115.81 | 83.15 | 65.28 | 51.94 | 48.29 | 57.59 | 81.01 |
| Malaysia | 289.52 | 698.66 | 639.31 | 532.56 | 430.06 | 328.92 | 276.26 | 205.37 | 150.71 | 102.03 | 89.06 | 83.51 | 94.84 | 119.20 | 174.79 | 240.21 | 278.98 |
| Maldives | 34.70 | 83.73 | 109.33 | 204.82 | 189.04 | 223.24 | 44.00 | 32.80 | 30.06 | 25.45 | 24.66 | 24.81 | 27.97 | 185.60 | 34.42 | 30.23 | 29.03 |
| Mali | 35.39 | 86.25 | 106.46 | 242.10 | 228.86 | 288.48 | 43.50 | 34.04 | 31.12 | 26.32 | 26.41 | 26.94 | 30.19 | 211.14 | 35.71 | 28.69 | 24.87 |
| Malta | 58.73 | 198.40 | 202.72 | 133.64 | 96.17 | 71.32 | 57.12 | 47.67 | 41.48 | 35.68 | 32.05 | 27.48 | 27.54 | 25.89 | 28.67 | 28.66 | 37.64 |
| Marshall Islands | 65.70 | 213.90 | 225.54 | 156.36 | 114.61 | 92.04 | 65.29 | 52.29 | 40.61 | 33.84 | 33.36 | 27.33 | 27.58 | 28.59 | 32.57 | 31.65 | 43.91 |
| Mauritania | 25.21 | 90.57 | 94.96 | 75.27 | 58.72 | 46.40 | 38.97 | 33.56 | 28.95 | 26.23 | 25.50 | 22.64 | 21.02 | 21.55 | 22.28 | 21.95 | 19.25 |
| Mauritius | 221.56 | 654.35 | 579.54 | 530.04 | 376.65 | 306.88 | 215.74 | 132.45 | 98.34 | 64.73 | 52.23 | 48.79 | 48.80 | 57.67 | 71.67 | 75.77 | 82.68 |
| Mexico | 90.55 | 225.24 | 221.46 | 133.28 | 95.57 | 85.83 | 67.36 | 57.22 | 40.31 | 39.40 | 32.68 | 54.05 | 35.59 | 37.66 | 28.19 | 28.40 | 31.61 |
| Republic of Moldova | 27.93 | 101.40 | 108.25 | 85.32 | 65.86 | 53.55 | 45.20 | 38.97 | 33.24 | 31.08 | 30.47 | 26.57 | 25.50 | 26.72 | 27.45 | 28.05 | 25.10 |
| Mongolia | 128.42 | 378.09 | 426.98 | 359.36 | 347.05 | 293.87 | 230.60 | 180.36 | 140.60 | 117.04 | 105.13 | 81.16 | 76.76 | 75.06 | 64.13 | 59.73 | 67.15 |
| Montenegro | 108.13 | 208.31 | 217.98 | 174.80 | 144.17 | 113.57 | 93.80 | 71.34 | 56.63 | 43.54 | 35.86 | 26.35 | 24.14 | 21.69 | 20.92 | 22.15 | 27.31 |
| Morocco | 92.51 | 233.78 | 227.39 | 134.31 | 94.64 | 82.38 | 64.79 | 56.82 | 41.71 | 39.40 | 33.03 | 50.94 | 34.23 | 36.24 | 27.03 | 26.00 | 29.47 |
| Mozambique | 146.16 | 326.63 | 349.82 | 281.76 | 213.94 | 159.87 | 122.42 | 88.42 | 61.04 | 43.25 | 35.23 | 32.32 | 31.70 | 32.16 | 35.34 | 40.32 | 45.90 |
| Myanmar | 123.55 | 338.30 | 354.94 | 265.33 | 198.86 | 152.53 | 124.20 | 109.06 | 91.51 | 74.15 | 56.45 | 43.81 | 44.44 | 50.55 | 49.09 | 44.97 | 41.43 |
| Namibia | 72.89 | 198.37 | 214.68 | 160.24 | 112.90 | 76.47 | 59.25 | 47.98 | 38.28 | 29.34 | 24.41 | 21.57 | 20.69 | 19.58 | 20.78 | 25.48 | 22.42 |
| Nepal | 123.36 | 355.86 | 399.12 | 327.57 | 284.05 | 253.53 | 217.31 | 179.82 | 141.52 | 110.28 | 89.35 | 75.21 | 70.64 | 74.09 | 76.04 | 91.03 | 141.74 |
| Netherlands | 34.89 | 85.06 | 108.96 | 184.48 | 170.78 | 174.63 | 50.13 | 36.00 | 32.02 | 30.11 | 31.50 | 32.37 | 30.92 | 76.98 | 27.87 | 24.76 | 19.84 |
| New Zealand | 66.40 | 186.20 | 183.13 | 117.65 | 80.90 | 59.61 | 47.02 | 39.54 | 34.76 | 29.25 | 26.92 | 23.63 | 23.14 | 22.79 | 25.40 | 26.49 | 36.53 |
| Nicaragua | 76.84 | 190.51 | 210.37 | 172.91 | 132.81 | 118.92 | 121.54 | 129.43 | 125.42 | 109.24 | 102.89 | 81.66 | 72.71 | 76.16 | 77.29 | 77.92 | 168.32 |
| Niger | 92.62 | 227.93 | 220.97 | 131.53 | 92.31 | 81.20 | 64.51 | 55.52 | 40.35 | 39.10 | 31.51 | 46.97 | 35.66 | 39.71 | 29.03 | 28.22 | 32.49 |
| Nigeria | 46.58 | 143.48 | 146.50 | 119.70 | 91.58 | 79.73 | 75.59 | 67.90 | 73.48 | 57.72 | 64.69 | 52.98 | 52.57 | 45.91 | 49.31 | 62.00 | 69.15 |
| Democratic People's Republic of Korea | 191.74 | 420.94 | 423.93 | 352.92 | 284.67 | 230.81 | 188.43 | 129.61 | 96.26 | 64.77 | 49.79 | 42.12 | 43.33 | 46.56 | 59.96 | 76.73 | 90.31 |
| Northern Mariana Islands | 270.24 | 724.31 | 733.04 | 584.39 | 484.67 | 380.43 | 274.38 | 218.85 | 142.64 | 95.07 | 58.74 | 39.48 | 38.51 | 35.35 | 33.49 | 31.70 | 59.61 |
| Norway | 59.02 | 153.40 | 170.30 | 138.65 | 111.88 | 83.08 | 64.57 | 50.59 | 40.47 | 31.48 | 24.71 | 17.63 | 16.08 | 14.37 | 13.44 | 14.02 | 17.33 |
| Oman | 26.04 | 94.25 | 101.41 | 79.01 | 60.99 | 49.40 | 41.74 | 35.95 | 30.00 | 26.61 | 25.58 | 21.43 | 19.89 | 20.30 | 20.46 | 20.24 | 18.94 |
| Pakistan | 20.97 | 77.43 | 84.05 | 68.96 | 55.12 | 44.27 | 37.34 | 32.48 | 28.66 | 27.34 | 27.21 | 24.39 | 24.19 | 25.83 | 27.11 | 27.94 | 24.22 |
| Palestine | 94.87 | 224.25 | 218.17 | 131.02 | 91.64 | 78.59 | 61.78 | 54.77 | 39.96 | 38.05 | 31.44 | 47.06 | 31.80 | 33.25 | 24.78 | 25.06 | 29.50 |
| Panama | 95.80 | 279.08 | 285.59 | 203.57 | 139.19 | 97.80 | 77.85 | 67.57 | 55.99 | 43.85 | 36.57 | 31.80 | 29.74 | 27.54 | 30.30 | 37.29 | 31.32 |
| Papua New Guinea | 98.16 | 263.48 | 258.60 | 151.07 | 101.91 | 88.93 | 63.68 | 61.78 | 44.46 | 50.76 | 37.39 | 59.09 | 35.55 | 41.04 | 22.95 | 29.61 | 35.85 |
| Paraguay | 402.95 | 1,055.61 | 1,184.83 | 888.80 | 794.19 | 583.20 | 594.62 | 596.48 | 552.89 | 248.58 | 168.75 | 91.86 | 77.55 | 64.05 | 81.21 | 105.97 | 148.51 |
| Peru | 72.49 | 241.92 | 253.66 | 182.22 | 139.81 | 110.13 | 93.58 | 76.20 | 60.21 | 45.31 | 35.36 | 29.88 | 26.51 | 25.20 | 30.02 | 29.69 | 32.19 |
| Philippines | 56.65 | 158.03 | 159.58 | 127.14 | 100.60 | 88.99 | 86.94 | 79.66 | 88.84 | 71.86 | 82.29 | 68.24 | 66.93 | 56.82 | 60.10 | 75.10 | 89.24 |
| Poland | 85.18 | 224.22 | 216.58 | 130.27 | 88.83 | 70.50 | 58.30 | 46.92 | 38.03 | 32.19 | 30.80 | 31.70 | 30.15 | 27.39 | 19.90 | 15.22 | 15.37 |
| Portugal | 76.22 | 240.56 | 253.00 | 177.33 | 124.87 | 97.34 | 77.98 | 63.00 | 48.22 | 36.18 | 26.63 | 20.62 | 17.61 | 17.04 | 16.43 | 16.17 | 19.09 |
| Puerto Rico | 102.82 | 200.68 | 207.63 | 169.63 | 134.81 | 103.16 | 83.28 | 67.46 | 55.14 | 42.67 | 36.67 | 25.37 | 23.61 | 20.32 | 17.96 | 18.69 | 20.95 |
| Qatar | 84.82 | 197.81 | 189.87 | 113.97 | 79.52 | 67.13 | 52.85 | 46.67 | 34.95 | 32.44 | 26.06 | 40.53 | 23.75 | 22.60 | 18.52 | 19.66 | 21.02 |
| Romania | 80.46 | 217.78 | 228.61 | 167.50 | 120.11 | 87.76 | 67.75 | 52.61 | 40.68 | 31.48 | 24.68 | 19.62 | 16.65 | 15.02 | 14.98 | 16.21 | 18.11 |
| Russian Federation | 108.16 | 221.08 | 219.14 | 173.14 | 134.44 | 106.71 | 87.21 | 72.10 | 62.38 | 57.91 | 47.89 | 37.74 | 32.46 | 27.21 | 17.59 | 19.21 | 18.58 |
| Rwanda | 54.35 | 170.10 | 172.94 | 114.98 | 81.13 | 59.62 | 47.50 | 39.48 | 34.63 | 29.48 | 25.58 | 21.86 | 22.65 | 23.25 | 27.43 | 29.10 | 35.64 |
| Saint Lucia | 134.33 | 355.15 | 354.93 | 241.97 | 162.55 | 107.57 | 82.97 | 64.02 | 51.60 | 39.77 | 33.74 | 29.34 | 26.29 | 24.20 | 25.92 | 30.26 | 36.98 |
| Saint Vincent and the Grenadines | 130.79 | 329.23 | 360.82 | 309.46 | 235.46 | 189.78 | 157.86 | 110.80 | 79.45 | 54.29 | 43.88 | 39.02 | 40.13 | 45.24 | 58.80 | 75.36 | 93.07 |
| Samoa | 171.67 | 410.29 | 531.76 | 578.64 | 609.42 | 502.26 | 404.76 | 246.84 | 164.29 | 92.62 | 62.98 | 48.28 | 50.75 | 53.96 | 47.47 | 44.91 | 31.81 |
| Sao Tome and Principe | 74.15 | 232.43 | 251.47 | 180.45 | 132.59 | 104.77 | 87.39 | 70.72 | 57.44 | 42.77 | 36.09 | 33.58 | 28.99 | 27.03 | 26.82 | 33.04 | 45.11 |
| Saudi Arabia | 113.92 | 265.16 | 271.62 | 206.31 | 154.59 | 114.80 | 91.42 | 74.84 | 57.85 | 43.54 | 34.74 | 29.13 | 26.88 | 26.41 | 25.23 | 25.98 | 27.82 |
| Senegal | 129.95 | 398.93 | 430.52 | 315.99 | 223.15 | 180.52 | 155.96 | 154.17 | 129.66 | 98.78 | 73.26 | 55.56 | 41.99 | 31.52 | 26.73 | 25.81 | 31.05 |
| Serbia | 79.95 | 189.64 | 200.49 | 173.23 | 136.98 | 111.56 | 107.57 | 103.62 | 89.93 | 62.40 | 47.33 | 41.15 | 37.24 | 31.85 | 33.59 | 38.44 | 41.62 |
| Seychelles | 276.86 | 914.35 | 1,249.10 | 1,288.99 | 1,043.20 | 670.88 | 397.15 | 283.56 | 217.50 | 162.97 | 123.32 | 95.66 | 81.71 | 66.93 | 55.47 | 64.64 | 87.10 |
| Sierra Leone | 49.76 | 90.44 | 102.45 | 240.97 | 238.75 | 274.40 | 41.85 | 32.42 | 29.62 | 24.83 | 24.08 | 24.02 | 26.89 | 213.62 | 36.55 | 31.05 | 31.26 |
| Singapore | 124.06 | 216.90 | 209.94 | 165.68 | 134.22 | 106.50 | 84.82 | 63.74 | 50.02 | 35.98 | 28.51 | 24.30 | 23.44 | 23.71 | 21.53 | 20.94 | 18.63 |
| Slovakia | 133.81 | 226.07 | 220.95 | 176.42 | 140.25 | 107.70 | 84.83 | 61.42 | 45.96 | 32.83 | 24.77 | 19.86 | 17.14 | 16.72 | 15.98 | 16.29 | 15.97 |
| Slovenia | 134.14 | 219.25 | 210.00 | 167.42 | 133.76 | 102.77 | 82.12 | 59.61 | 43.91 | 31.64 | 23.84 | 18.81 | 16.44 | 15.28 | 14.50 | 14.41 | 14.56 |
| Solomon Islands | 131.39 | 236.62 | 213.97 | 127.29 | 89.58 | 76.27 | 60.51 | 52.31 | 38.34 | 36.13 | 30.11 | 47.01 | 31.14 | 32.31 | 24.54 | 26.63 | 29.80 |
| Somalia | 143.61 | 315.26 | 329.70 | 256.05 | 194.03 | 144.85 | 108.67 | 79.54 | 56.25 | 40.83 | 34.41 | 31.30 | 29.07 | 28.63 | 29.90 | 31.81 | 34.60 |
| South Africa | 35.21 | 159.76 | 128.88 | 96.37 | 73.82 | 60.09 | 51.86 | 48.31 | 46.34 | 49.69 | 49.17 | 38.78 | 36.04 | 38.82 | 42.25 | 46.25 | 38.01 |
| Republic of Korea | 73.38 | 257.27 | 276.13 | 197.18 | 145.20 | 114.46 | 93.28 | 74.19 | 57.15 | 43.18 | 32.10 | 25.12 | 21.85 | 20.46 | 19.09 | 19.98 | 24.77 |
| South Sudan | 27.40 | 98.40 | 103.01 | 81.84 | 63.13 | 50.61 | 42.89 | 37.24 | 32.13 | 29.86 | 28.86 | 25.49 | 24.10 | 24.19 | 24.89 | 24.56 | 22.63 |
| Spain | 88.11 | 253.18 | 298.65 | 260.46 | 188.31 | 122.28 | 91.02 | 77.19 | 62.29 | 46.21 | 39.34 | 37.64 | 32.62 | 31.25 | 38.68 | 52.36 | 47.51 |
| Sri Lanka | 87.35 | 242.03 | 276.36 | 190.69 | 160.53 | 149.46 | 99.24 | 78.31 | 73.71 | 56.44 | 50.11 | 45.22 | 46.15 | 48.08 | 57.17 | 62.18 | 81.31 |
| Sudan | 27.72 | 100.37 | 105.82 | 82.85 | 63.30 | 51.19 | 43.16 | 37.35 | 32.24 | 29.57 | 27.59 | 23.84 | 22.05 | 21.53 | 21.65 | 21.70 | 20.30 |
| Suriname | 115.55 | 258.76 | 259.22 | 193.39 | 143.79 | 106.08 | 86.26 | 70.19 | 54.62 | 41.46 | 33.38 | 27.34 | 25.10 | 23.64 | 23.13 | 26.41 | 35.12 |
| Eswatini | 128.13 | 288.38 | 294.20 | 217.51 | 154.64 | 109.89 | 88.73 | 78.09 | 63.50 | 48.33 | 40.09 | 35.15 | 32.16 | 29.86 | 31.67 | 36.09 | 34.82 |
| Sweden | 197.58 | 483.72 | 525.67 | 402.44 | 272.48 | 189.45 | 140.73 | 115.97 | 92.12 | 66.04 | 56.15 | 49.38 | 45.95 | 37.14 | 42.19 | 51.18 | 60.97 |
| Switzerland | 89.44 | 212.65 | 204.94 | 124.16 | 85.18 | 73.55 | 58.53 | 50.44 | 37.39 | 33.81 | 27.73 | 40.15 | 26.12 | 26.37 | 21.30 | 22.37 | 25.40 |
| Syrian Arab Republic | 34.74 | 81.52 | 97.32 | 189.17 | 178.52 | 221.74 | 41.59 | 31.48 | 29.96 | 24.30 | 22.85 | 22.33 | 25.83 | 146.38 | 30.43 | 24.85 | 18.71 |
| Taiwan (Province of China) | 116.44 | 283.62 | 327.43 | 294.88 | 245.44 | 234.53 | 222.14 | 215.69 | 202.97 | 154.58 | 138.56 | 107.04 | 99.80 | 113.43 | 118.54 | 137.51 | 385.99 |
| Tajikistan | 35.24 | 83.70 | 101.30 | 175.47 | 178.38 | 221.95 | 42.65 | 33.22 | 29.45 | 24.60 | 24.13 | 23.80 | 27.09 | 249.65 | 33.72 | 28.92 | 26.87 |
| United Republic of Tanzania | 301.39 | 518.41 | 513.85 | 429.41 | 357.00 | 302.27 | 255.27 | 180.24 | 129.07 | 81.86 | 65.08 | 58.27 | 65.55 | 81.55 | 118.13 | 154.92 | 171.91 |
| Thailand | 78.79 | 233.47 | 235.76 | 163.99 | 127.11 | 110.01 | 93.27 | 77.46 | 65.98 | 55.07 | 50.28 | 44.41 | 44.48 | 46.87 | 50.93 | 48.97 | 73.88 |
| Bahamas | 122.30 | 353.41 | 410.01 | 338.20 | 283.51 | 236.80 | 197.38 | 162.13 | 123.88 | 96.54 | 76.86 | 67.05 | 62.78 | 64.06 | 62.75 | 74.06 | 108.34 |
| Gambia | 120.19 | 229.52 | 236.86 | 216.26 | 183.26 | 144.56 | 117.11 | 79.89 | 60.25 | 40.24 | 29.37 | 22.22 | 19.91 | 19.88 | 17.61 | 16.28 | 15.14 |
| Timor-Leste | 285.18 | 1,085.55 | 1,293.06 | 988.30 | 731.79 | 558.17 | 451.85 | 352.27 | 283.17 | 164.87 | 107.99 | 73.37 | 54.51 | 52.03 | 64.63 | 87.74 | 104.30 |
| Togo | 297.62 | 615.98 | 702.88 | 553.05 | 460.75 | 406.22 | 331.75 | 225.54 | 151.76 | 92.54 | 71.76 | 62.50 | 63.50 | 72.62 | 106.88 | 145.87 | 191.55 |
| Tonga | 117.67 | 329.43 | 345.99 | 248.66 | 191.49 | 162.53 | 146.25 | 128.68 | 108.08 | 97.08 | 82.99 | 76.02 | 97.01 | 138.60 | 157.32 | 142.28 | 206.12 |
| Trinidad and Tobago | 90.68 | 351.75 | 433.34 | 371.41 | 345.53 | 295.12 | 215.56 | 143.47 | 87.48 | 59.57 | 48.58 | 42.12 | 39.20 | 34.35 | 32.75 | 33.21 | 33.52 |
| Tunisia | 82.03 | 277.55 | 311.18 | 231.78 | 160.13 | 114.09 | 91.43 | 79.52 | 65.37 | 51.86 | 39.53 | 30.96 | 27.48 | 26.73 | 24.13 | 24.11 | 29.43 |
| Turkey | 93.09 | 318.17 | 334.26 | 219.59 | 143.06 | 97.90 | 71.83 | 57.55 | 48.16 | 42.43 | 37.71 | 32.78 | 29.36 | 27.82 | 27.62 | 29.84 | 34.58 |
| Turkmenistan | 64.74 | 193.69 | 190.91 | 123.64 | 85.80 | 62.03 | 48.30 | 39.87 | 35.44 | 29.92 | 27.15 | 23.21 | 22.97 | 21.93 | 24.96 | 24.71 | 33.50 |
| Uganda | 23.27 | 92.43 | 98.02 | 77.56 | 60.15 | 47.53 | 40.60 | 35.42 | 30.46 | 28.71 | 27.67 | 23.03 | 21.58 | 21.06 | 21.44 | 20.81 | 19.69 |
| Ukraine | 99.64 | 220.33 | 215.43 | 127.81 | 90.11 | 76.65 | 60.05 | 53.68 | 39.17 | 37.72 | 30.08 | 47.76 | 29.14 | 30.56 | 23.36 | 29.72 | 32.12 |
| United Arab Emirates | 96.81 | 215.84 | 207.19 | 125.94 | 87.38 | 73.63 | 58.70 | 52.57 | 39.13 | 36.25 | 29.88 | 44.57 | 27.99 | 28.04 | 21.82 | 23.87 | 26.38 |
| United Kingdom | 121.49 | 232.72 | 243.53 | 202.69 | 173.25 | 131.80 | 106.53 | 75.91 | 57.29 | 40.23 | 29.30 | 23.57 | 22.10 | 21.45 | 19.11 | 18.57 | 17.68 |
| United States of America | 115.00 | 362.20 | 422.25 | 349.96 | 294.03 | 250.37 | 201.43 | 157.33 | 122.00 | 95.71 | 77.13 | 66.76 | 64.18 | 70.47 | 72.62 | 88.25 | 117.36 |
| Uruguay | 88.28 | 287.62 | 303.73 | 214.70 | 155.21 | 122.97 | 101.62 | 83.05 | 65.05 | 51.94 | 40.39 | 34.58 | 32.89 | 35.93 | 39.18 | 43.94 | 54.58 |
| Uzbekistan | 177.78 | 583.57 | 649.19 | 459.92 | 321.70 | 233.32 | 187.31 | 159.87 | 140.55 | 116.93 | 87.43 | 75.27 | 69.66 | 74.23 | 63.58 | 67.44 | 82.21 |
| Vanuatu | 90.18 | 222.94 | 219.01 | 128.66 | 90.93 | 81.11 | 62.05 | 53.21 | 38.42 | 36.94 | 29.85 | 45.71 | 31.03 | 31.52 | 23.93 | 25.56 | 30.32 |
| Venezuela (Bolivarian Republic of) | 43.78 | 93.69 | 108.91 | 224.76 | 219.19 | 261.38 | 45.41 | 36.04 | 32.32 | 27.28 | 25.29 | 24.50 | 26.83 | 207.45 | 34.29 | 28.52 | 27.67 |
| Viet nam | 171.71 | 663.42 | 844.05 | 717.63 | 514.69 | 333.23 | 247.42 | 210.03 | 167.34 | 119.51 | 82.83 | 61.35 | 51.22 | 43.55 | 41.52 | 53.92 | 67.76 |
| United States Virgin Islands | 125.48 | 511.48 | 550.19 | 434.78 | 320.16 | 272.96 | 238.51 | 181.91 | 137.28 | 101.71 | 70.69 | 77.25 | 72.22 | 62.88 | 61.65 | 73.18 | 153.77 |
| Yemen | 423.29 | 1,034.23 | 1,231.55 | 1,200.94 | 1,194.13 | 1,107.52 | 932.60 | 536.97 | 356.55 | 176.71 | 105.79 | 70.91 | 57.99 | 51.97 | 51.33 | 56.04 | 69.96 |
| Zambia | 39.72 | 100.84 | 127.67 | 289.06 | 279.18 | 330.84 | 56.20 | 42.27 | 38.31 | 34.14 | 35.22 | 36.18 | 38.28 | 231.02 | 41.53 | 35.23 | 33.14 |
| Zimbabwe | 953.43 | 3,555.88 | 4,838.23 | 4,576.98 | 4,000.27 | 3,365.11 | 2,893.38 | 2,355.98 | 1,699.39 | 901.81 | 440.11 | 205.08 | 121.82 | 103.05 | 101.13 | 109.85 | 124.69 |
| Monaco | 183.62 | 589.15 | 693.93 | 550.16 | 441.79 | 199.78 | 214.02 | 96.35 | 77.60 | 51.24 | 34.42 | 29.27 | 28.24 | 29.37 | 27.21 | 30.70 | 30.50 |
| San Marino | 199.29 | 318.18 | 297.99 | 230.97 | 177.17 | 135.97 | 108.00 | 92.86 | 73.01 | 60.42 | 51.50 | 46.06 | 40.51 | 39.18 | 42.88 | 55.18 | 63.66 |
| Saint Kitts and Nevis | 87.91 | 286.20 | 310.12 | 225.80 | 161.34 | 119.91 | 101.43 | 90.34 | 77.84 | 64.63 | 48.32 | 40.46 | 39.10 | 39.99 | 36.43 | 38.05 | 50.75 |
| Cook Islands | 79.16 | 199.29 | 195.55 | 116.26 | 81.03 | 69.82 | 55.38 | 47.98 | 35.50 | 33.52 | 27.52 | 42.93 | 27.18 | 26.72 | 21.72 | 22.78 | 25.15 |
| Nauru | 60.48 | 161.83 | 175.17 | 139.80 | 108.27 | 79.90 | 62.74 | 48.99 | 38.23 | 28.64 | 22.18 | 17.17 | 14.85 | 13.16 | 11.95 | 12.34 | 12.67 |
| Niue | 117.08 | 373.73 | 412.30 | 347.42 | 245.87 | 153.15 | 73.52 | 50.78 | 51.65 | 68.53 | 71.34 | 63.53 | 67.06 | 92.31 | 142.82 | 200.14 | 306.28 |
| Palau | 83.20 | 256.36 | 309.87 | 266.77 | 248.05 | 222.62 | 188.88 | 154.42 | 119.01 | 93.99 | 75.55 | 66.76 | 63.50 | 67.31 | 65.19 | 74.18 | 100.07 |
| Tokelau | 30.64 | 86.37 | 116.17 | 281.86 | 277.19 | 335.87 | 48.80 | 36.92 | 33.95 | 29.24 | 29.84 | 31.18 | 33.37 | 255.18 | 40.91 | 33.68 | 28.63 |
| Tuvalu | 77.85 | 189.83 | 211.32 | 178.77 | 143.58 | 134.29 | 146.28 | 166.00 | 168.38 | 141.26 | 128.97 | 90.26 | 82.67 | 86.01 | 76.04 | 61.01 | 91.53 |

**Supplementary Table 3 Age-standarized incidence rate of global burden of drug use disorders in 204 countries from 1990 to 2021, by locations and sex.**

| nation | sex | Incidence No.(95%UI) | | | ASIR (per 100,000) No.95%UI) | | |
| --- | --- | --- | --- | --- | --- | --- | --- |
|  |  | 1990 | 2021 | 1990-2021 EAPC No.(95%CI) | 1990 | 2021 | 1990-2021 EAPC No.(95%CI) |
| Afghanistan | both | 14,763.95(12,277.90; 17,643.36) | 41,366.69(34,769.75; 47,786.12) | 4.22(-6.69; 16.42) | 156.89(129.97; 185.53) | 145.72(122.13; 169.24) | 0.39(-4.69; 5.74) |
| Albania | both | 5,874.89(4,743.21; 7,249.46) | 4,621.33(3,868.18; 5,424.94) | -0.71(-9.07; 8.43) | 171.33(138.32; 209.08) | 175.38(146.73; 206.30) | 0.26(-4.99; 5.81) |
| Algeria | both | 32,284.58(26,522.24; 38,765.89) | 66,958.77(55,938.15; 79,130.15) | 2.75(-8.48; 15.37) | 146.15(118.99; 175.49) | 147.58(123.66; 172.72) | 0.41(-4.68; 5.78) |
| American Samoa | both | 103.79(84.27; 130.82) | 98.53(79.39; 122.96) | 0.08(-4.60; 4.99) | 212.94(176.34; 264.34) | 193.53(157.71; 235.50) | -0.06(-5.39; 5.58) |
| Andorra | both | 192.51(155.80; 233.42) | 235.03(196.04; 286.14) | 0.75(-4.83; 6.65) | 305.35(249.74; 372.32) | 280.53(233.30; 340.67) | 0.15(-5.57; 6.20) |
| Angola | both | 11,921.06(9,924.06; 14,218.75) | 32,402.65(26,666.61; 39,443.03) | 3.71(-6.91; 15.54) | 131.74(109.61; 156.03) | 115.22(95.43; 135.90) | -0.01(-4.84; 5.05) |
| Antigua and Barbuda | both | 130.50(104.64; 160.91) | 184.60(150.09; 223.26) | 1.44(-3.95; 7.14) | 209.60(170.02; 257.62) | 197.93(158.70; 246.37) | 0.08(-5.31; 5.77) |
| Argentina | both | 64,135.43(52,907.89; 76,174.74) | 84,881.64(71,511.19; 100,693.64) | 1.33(-9.97; 14.05) | 197.96(163.00; 235.85) | 180.41(152.92; 212.56) | 0.10(-5.18; 5.67) |
| Armenia | both | 5,794.49(4,745.11; 6,912.13) | 4,651.26(3,882.77; 5,492.61) | -0.32(-8.72; 8.86) | 165.83(136.39; 198.23) | 157.96(133.58; 184.26) | 0.19(-4.96; 5.62) |
| Australia | both | 97,422.16(82,976.28; 113,122.86) | 104,379.27(89,570.16; 120,390.32) | 0.69(-10.74; 13.58) | 557.18(475.39; 643.21) | 429.39(370.03; 491.99) | -0.33(-6.44; 6.19) |
| Austria | both | 20,763.99(17,072.06; 24,758.97) | 23,289.67(19,746.44; 27,027.40) | 0.71(-9.32; 11.84) | 262.11(216.14; 311.96) | 284.40(241.97; 325.95) | 0.57(-5.19; 6.67) |
| Azerbaijan | both | 11,851.74(9,684.29; 14,224.86) | 17,070.38(14,230.32; 20,410.80) | 1.69(-8.13; 12.55) | 159.37(130.26; 192.44) | 152.09(126.70; 180.41) | 0.17(-4.94; 5.55) |
| Bahamas | both | 689.12(561.75; 830.31) | 953.64(788.14; 1,151.96) | 1.31(-5.68; 8.82) | 244.31(201.43; 291.67) | 226.10(185.15; 273.82) | 0.01(-5.49; 5.83) |
| Bahrain | both | 893.27(726.97; 1,085.61) | 2,564.30(2,093.44; 3,116.59) | 4.75(-3.49; 13.69) | 155.05(127.81; 185.86) | 133.69(110.41; 160.04) | -0.10(-5.07; 5.13) |
| Bangladesh | both | 136,530.75(111,855.70; 162,614.57) | 234,784.69(196,162.02; 281,052.47) | 2.27(-10.09; 16.33) | 151.92(124.94; 180.42) | 136.65(114.37; 164.56) | 0.15(-4.86; 5.41) |
| Barbados | both | 644.98(519.10; 802.05) | 590.16(489.32; 695.86) | 0.10(-6.34; 6.99) | 244.43(197.03; 304.50) | 216.77(179.54; 261.00) | -0.14(-5.58; 5.62) |
| Belarus | both | 28,187.00(23,656.50; 33,237.38) | 20,088.05(16,889.21; 23,740.15) | -0.67(-10.42; 10.14) | 269.52(227.64; 319.54) | 223.93(189.66; 266.36) | -0.16(-5.63; 5.63) |
| Belgium | both | 29,747.84(24,450.25; 35,527.96) | 28,331.19(24,265.07; 32,851.99) | 0.22(-9.93; 11.52) | 305.80(252.47; 363.69) | 276.09(236.35; 317.05) | 0.01(-5.69; 6.04) |
| Belize | both | 424.46(340.66; 525.41) | 931.22(763.90; 1,138.02) | 2.95(-4.12; 10.54) | 220.41(182.01; 262.35) | 193.01(159.35; 233.68) | -0.15(-5.48; 5.47) |
| Benin | both | 4,141.53(3,397.41; 4,995.52) | 11,927.28(9,932.14; 14,232.93) | 3.95(-5.71; 14.61) | 109.38(90.02; 131.24) | 101.50(84.95; 120.11) | 0.25(-4.46; 5.20) |
| Bermuda | both | 153.08(126.30; 183.33) | 122.61(101.17; 148.66) | -0.38(-5.26; 4.75) | 238.07(194.20; 291.90) | 215.49(172.51; 268.04) | -0.02(-5.46; 5.74) |
| Bhutan | both | 711.81(571.75; 877.94) | 1,038.90(859.30; 1,233.40) | 2.08(-5.04; 9.75) | 124.56(101.25; 148.81) | 122.13(101.57; 144.83) | 0.32(-4.58; 5.47) |
| Bolivia (Plurinational State of) | both | 11,789.07(9,985.80; 13,553.43) | 18,682.82(15,676.66; 21,568.57) | 2.11(-7.83; 13.12) | 193.94(165.30; 223.02) | 151.36(127.47; 174.13) | -0.26(-5.33; 5.09) |
| Bosnia and Herzegovina | both | 7,497.13(5,958.99; 9,113.46) | 4,432.60(3,583.76; 5,355.82) | -0.94(-9.25; 8.13) | 152.43(120.80; 185.62) | 140.19(113.43; 168.61) | 0.24(-4.79; 5.54) |
| Botswana | both | 2,050.33(1,702.95; 2,435.52) | 3,322.13(2,774.43; 3,925.96) | 2.20(-6.07; 11.20) | 167.96(140.62; 196.21) | 127.37(106.89; 149.90) | -0.21(-5.12; 4.96) |
| Brazil | both | 324,383.18(273,468.18; 392,422.86) | 411,752.60(350,243.05; 474,141.24) | 1.31(-11.47; 15.93) | 204.66(174.34; 243.51) | 182.11(154.74; 209.69) | 0.16(-5.14; 5.76) |
| Brunei Darussalam | both | 681.16(549.67; 832.47) | 1,101.81(899.49; 1,330.61) | 1.84(-5.33; 9.56) | 241.94(196.20; 292.81) | 211.91(171.78; 258.82) | -0.17(-5.59; 5.57) |
| Bulgaria | both | 16,309.02(13,501.05; 19,336.74) | 11,657.72(9,912.97; 13,715.07) | -0.86(-10.07; 9.30) | 193.24(160.85; 227.63) | 201.10(172.81; 230.79) | 0.37(-5.02; 6.05) |
| Burkina Faso | both | 7,488.59(6,148.90; 9,071.67) | 17,815.81(14,769.55; 20,995.51) | 3.62(-6.42; 14.72) | 102.26(84.42; 122.67) | 94.04(77.89; 110.42) | 0.22(-4.41; 5.08) |
| Burundi | both | 5,522.83(4,469.44; 7,069.80) | 12,091.78(9,607.13; 15,405.72) | 3.06(-6.54; 13.65) | 114.17(93.40; 139.16) | 96.81(79.27; 119.12) | -0.18(-4.82; 4.70) |
| Cabo Verde | both | 368.96(308.43; 433.62) | 733.45(601.30; 878.15) | 3.16(-3.69; 10.50) | 130.75(109.05; 153.79) | 119.78(98.45; 143.88) | 0.48(-4.41; 5.62) |
| Cambodia | both | 13,606.52(11,213.62; 16,580.42) | 24,129.49(19,971.38; 28,368.18) | 2.21(-7.99; 13.53) | 145.20(121.27; 173.98) | 134.05(111.75; 156.45) | 0.00(-4.98; 5.24) |
| Cameroon | both | 10,755.25(8,927.54; 12,632.83) | 29,380.77(24,540.15; 35,021.20) | 4.17(-6.40; 15.94) | 124.98(104.58; 147.39) | 104.41(87.77; 123.99) | 0.17(-4.57; 5.14) |
| Canada | both | 112,477.90(97,822.92; 128,343.81) | 130,071.09(115,623.44; 149,937.65) | 1.11(-10.56; 14.32) | 419.59(369.93; 472.74) | 414.66(370.16; 468.42) | 0.72(-5.42; 7.26) |
| Central African Republic | both | 3,055.12(2,569.92; 3,646.63) | 5,347.60(4,429.74; 6,485.63) | 2.11(-6.62; 11.65) | 125.16(105.23; 147.52) | 103.07(86.20; 122.28) | -0.28(-4.98; 4.66) |
| Chad | both | 5,093.44(4,224.18; 6,073.39) | 12,836.80(10,677.31; 15,387.94) | 3.53(-6.17; 14.23) | 107.90(89.99; 128.46) | 94.35(78.29; 112.53) | 0.08(-4.55; 4.94) |
| Chile | both | 32,343.00(27,200.39; 37,647.84) | 43,113.67(36,879.07; 49,718.61) | 1.18(-9.47; 13.08) | 229.29(192.88; 267.91) | 234.74(201.34; 270.02) | 0.33(-5.21; 6.21) |
| China | both | 3012,309.83(2552,862.41; 3500,349.66) | 2451,314.00(2046,472.04; 2907,370.53) | -0.18(-14.37; 16.37) | 229.82(194.76; 267.06) | 173.24(145.45; 203.89) | -0.63(-5.83; 4.85) |
| Colombia | both | 59,806.36(50,634.99; 69,769.40) | 92,195.19(78,321.81; 106,817.74) | 1.50(-9.90; 14.34) | 178.75(151.28; 209.16) | 181.54(154.32; 209.98) | 0.25(-5.04; 5.83) |
| Comoros | both | 468.88(375.37; 607.24) | 808.28(650.53; 984.61) | 2.07(-4.80; 9.44) | 113.80(91.65; 139.38) | 104.70(85.23; 126.14) | 0.07(-4.67; 5.04) |
| Congo | both | 3,115.44(2,599.99; 3,714.15) | 6,609.58(5,472.56; 7,955.00) | 2.85(-6.14; 12.71) | 145.80(122.01; 172.21) | 121.40(100.90; 144.84) | -0.19(-5.06; 4.93) |
| Cook Islands | both | 41.71(33.95; 52.38) | 33.11(26.67; 40.45) | -0.52(-4.08; 3.19) | 215.35(177.05; 265.15) | 194.23(156.94; 237.98) | -0.05(-5.39; 5.60) |
| Costa Rica | both | 5,145.11(4,201.76; 6,300.12) | 7,657.36(6,393.21; 8,960.39) | 1.66(-7.39; 11.59) | 170.25(139.43; 206.28) | 152.88(128.15; 178.57) | 0.10(-5.01; 5.49) |
| Croatia | both | 11,141.57(9,190.74; 13,210.46) | 8,425.03(7,211.75; 9,838.94) | -0.64(-9.58; 9.18) | 228.49(189.67; 271.55) | 227.22(196.20; 262.87) | 0.27(-5.25; 6.10) |
| Cuba | both | 24,532.09(19,970.73; 30,561.18) | 18,382.77(14,740.68; 22,544.65) | -0.56(-10.24; 10.17) | 204.83(166.31; 254.79) | 179.55(142.08; 228.61) | -0.20(-5.46; 5.34) |
| Cyprus | both | 1,897.38(1,514.86; 2,295.06) | 3,011.12(2,410.98; 3,625.30) | 2.04(-6.13; 10.93) | 235.20(188.65; 284.60) | 210.24(171.55; 249.72) | 0.02(-5.40; 5.76) |
| Czechia | both | 26,606.00(22,041.09; 31,319.92) | 22,707.91(19,260.71; 26,411.59) | -0.23(-10.13; 10.76) | 258.61(215.27; 303.45) | 253.86(216.55; 294.07) | 0.38(-5.25; 6.35) |
| Côte d'Ivoire | both | 11,834.29(9,842.42; 14,112.27) | 25,340.18(20,841.62; 29,988.00) | 2.94(-7.37; 14.40) | 114.51(95.33; 137.42) | 99.94(83.27; 117.52) | 0.12(-4.57; 5.04) |
| Democratic People's Republic of Korea | both | 42,781.96(35,698.75; 50,194.61) | 48,421.12(40,365.48; 56,669.54) | 0.79(-9.93; 12.78) | 200.21(166.33; 234.34) | 169.38(141.36; 197.93) | -0.24(-5.44; 5.24) |
| Democratic Republic of the Congo | both | 40,890.96(34,050.25; 49,178.78) | 89,804.20(73,930.22; 107,987.71) | 3.03(-8.50; 16.01) | 125.46(104.03; 147.56) | 107.19(89.53; 126.61) | -0.12(-4.87; 4.86) |
| Denmark | both | 19,061.71(15,978.06; 22,950.80) | 16,671.62(13,964.32; 19,466.11) | -0.10(-9.72; 10.54) | 360.00(302.46; 429.17) | 309.32(258.96; 361.01) | -0.14(-5.93; 6.01) |
| Djibouti | both | 537.70(427.71; 697.49) | 1,450.82(1,184.31; 1,785.66) | 3.96(-3.63; 12.13) | 117.72(95.69; 144.59) | 108.06(88.85; 132.39) | 0.12(-4.65; 5.13) |
| Dominica | both | 190.05(149.55; 254.60) | 154.11(122.74; 189.07) | -0.49(-5.59; 4.87) | 246.09(197.86; 317.37) | 223.20(176.99; 275.55) | -0.22(-5.69; 5.57) |
| Dominican Republic | both | 11,004.38(8,982.48; 13,365.81) | 19,031.92(15,462.40; 23,429.03) | 2.00(-7.96; 13.02) | 153.82(125.88; 185.24) | 164.23(132.97; 203.79) | 0.41(-4.79; 5.89) |
| Ecuador | both | 16,710.17(13,934.61; 19,640.39) | 27,761.95(23,255.32; 32,275.80) | 2.18(-8.16; 13.67) | 168.75(141.54; 197.22) | 148.09(124.09; 172.49) | 0.09(-5.00; 5.45) |
| Egypt | both | 65,360.12(53,216.60; 79,163.94) | 133,380.12(111,326.80; 161,255.75) | 2.62(-9.25; 16.04) | 127.48(103.88; 155.55) | 126.23(105.51; 152.24) | 0.25(-4.68; 5.44) |
| El Salvador | both | 6,685.32(5,595.97; 7,909.05) | 8,408.79(6,976.11; 10,111.55) | 1.02(-8.02; 10.95) | 139.35(115.63; 165.59) | 127.65(105.50; 153.76) | 0.02(-4.88; 5.16) |
| Equatorial Guinea | both | 469.04(393.55; 558.38) | 1,910.89(1,548.09; 2,292.41) | 5.44(-2.52; 14.06) | 132.42(111.28; 155.45) | 126.57(103.58; 150.29) | 0.48(-4.47; 5.68) |
| Eritrea | both | 3,231.17(2,621.89; 4,167.16) | 7,251.83(5,786.20; 8,942.86) | 3.15(-5.96; 13.15) | 119.04(98.54; 144.72) | 108.97(89.04; 131.92) | 0.01(-4.76; 5.02) |
| Estonia | both | 4,916.63(4,212.40; 5,697.25) | 4,379.76(3,928.16; 4,953.10) | -0.17(-8.52; 8.93) | 321.69(276.13; 371.48) | 406.43(367.40; 454.31) | 0.82(-5.29; 7.32) |
| Eswatini | both | 1,284.20(1,081.86; 1,515.39) | 1,710.71(1,447.37; 1,996.03) | 1.26(-6.29; 9.42) | 176.43(149.44; 205.31) | 144.23(122.18; 166.99) | -0.14(-5.18; 5.16) |
| Ethiopia | both | 47,195.38(38,588.66; 58,356.33) | 103,507.63(84,506.60; 126,272.73) | 2.79(-8.85; 15.91) | 105.96(88.70; 126.26) | 95.89(78.94; 113.56) | -0.02(-4.67; 4.84) |
| Fiji | both | 1,530.91(1,232.08; 1,952.47) | 1,622.05(1,302.65; 2,010.89) | 0.41(-7.03; 8.45) | 191.01(156.20; 238.13) | 171.34(137.98; 212.06) | -0.09(-5.30; 5.42) |
| Finland | both | 16,476.67(13,771.75; 19,560.88) | 15,464.61(13,426.75; 17,942.58) | 0.00(-9.56; 10.57) | 315.44(265.17; 372.34) | 317.56(277.28; 365.39) | 0.24(-5.59; 6.44) |
| France | both | 170,553.46(141,418.32; 203,584.38) | 170,474.15(144,815.96; 199,426.68) | 0.30(-11.53; 13.72) | 299.24(248.58; 354.86) | 286.37(241.95; 336.20) | 0.20(-5.53; 6.29) |
| Gabon | both | 1,409.14(1,177.16; 1,659.73) | 2,277.40(1,890.97; 2,723.90) | 2.03(-5.85; 10.58) | 161.38(135.47; 190.15) | 127.43(105.72; 151.56) | -0.37(-5.28; 4.79) |
| Gambia | both | 970.37(801.10; 1,148.87) | 2,097.56(1,735.93; 2,504.38) | 3.02(-4.86; 11.55) | 117.02(97.76; 139.30) | 96.87(80.20; 114.47) | -0.02(-4.67; 4.86) |
| Georgia | both | 8,974.13(7,335.91; 10,742.67) | 5,223.55(4,325.11; 6,175.84) | -1.38(-9.81; 7.82) | 160.41(131.05; 192.53) | 153.61(128.91; 179.60) | 0.23(-4.89; 5.63) |
| Germany | both | 226,571.34(187,889.22; 273,037.88) | 212,160.46(183,311.16; 253,506.58) | 0.03(-11.98; 13.67) | 286.35(239.96; 339.92) | 287.31(247.52; 339.06) | 0.35(-5.40; 6.44) |
| Ghana | both | 15,785.33(13,270.79; 18,755.84) | 32,794.89(26,989.58; 39,050.85) | 3.40(-7.20; 15.22) | 120.86(101.42; 142.87) | 98.94(81.71; 117.43) | 0.25(-4.43; 5.17) |
| Greece | both | 22,179.64(17,654.91; 27,422.14) | 18,711.03(15,449.55; 22,594.97) | -0.36(-10.08; 10.41) | 213.60(170.25; 263.20) | 206.61(172.65; 245.49) | 0.11(-5.30; 5.84) |
| Greenland | both | 195.93(163.30; 230.40) | 160.41(136.74; 186.53) | 0.00(-5.15; 5.43) | 331.05(275.27; 389.17) | 304.91(258.27; 358.25) | 0.26(-5.54; 6.41) |
| Grenada | both | 181.39(144.94; 232.11) | 217.95(177.98; 263.53) | 0.82(-4.72; 6.67) | 217.77(176.80; 269.07) | 204.43(164.27; 251.35) | 0.10(-5.32; 5.84) |
| Guam | both | 329.56(269.16; 399.24) | 313.55(255.59; 378.06) | 0.21(-5.62; 6.40) | 220.62(181.29; 271.60) | 203.01(162.81; 246.88) | 0.00(-5.39; 5.70) |
| Guatemala | both | 14,415.40(12,122.92; 16,987.50) | 24,639.28(20,507.68; 29,976.76) | 2.69(-7.54; 14.05) | 223.32(186.74; 265.79) | 153.64(127.82; 185.75) | -0.72(-5.77; 4.60) |
| Guinea | both | 5,239.59(4,306.30; 6,270.97) | 10,752.18(8,903.00; 12,843.55) | 2.69(-6.76; 13.10) | 104.35(86.38; 124.90) | 94.98(78.77; 112.57) | 0.16(-4.48; 5.03) |
| Guinea-Bissau | both | 874.90(728.38; 1,045.79) | 1,735.98(1,444.87; 2,080.00) | 2.79(-4.88; 11.09) | 105.96(87.63; 125.94) | 94.62(79.25; 112.59) | 0.14(-4.50; 5.00) |
| Guyana | both | 1,406.51(1,163.09; 1,697.96) | 1,299.38(1,059.52; 1,595.77) | 0.05(-7.15; 7.82) | 173.41(145.35; 207.16) | 162.81(132.57; 200.57) | 0.04(-5.13; 5.49) |
| Haiti | both | 9,767.43(7,930.19; 12,141.39) | 23,100.37(18,347.14; 28,895.99) | 2.77(-7.41; 14.07) | 161.45(132.35; 195.97) | 167.50(134.03; 206.61) | 0.13(-5.05; 5.59) |
| Honduras | both | 6,659.60(5,532.23; 7,984.62) | 16,411.18(13,531.92; 19,666.25) | 3.20(-6.70; 14.15) | 165.42(137.53; 196.93) | 155.95(129.61; 185.71) | 0.04(-5.08; 5.43) |
| Hungary | both | 20,661.73(17,137.72; 24,327.36) | 15,690.60(13,014.26; 18,602.53) | -0.48(-10.01; 10.06) | 202.04(167.23; 236.65) | 182.80(152.70; 214.08) | 0.17(-5.12; 5.75) |
| Iceland | both | 782.11(648.59; 930.91) | 1,074.86(917.16; 1,254.47) | 1.33(-5.78; 8.97) | 296.53(246.48; 352.56) | 318.52(272.44; 368.13) | 0.49(-5.37; 6.71) |
| India | both | 1129,088.60(939,366.33; 1347,653.49) | 2047,672.59(1706,130.84; 2396,268.76) | 2.41(-11.98; 19.14) | 137.74(114.80; 162.54) | 133.13(111.12; 155.54) | 0.29(-4.69; 5.53) |
| Indonesia | both | 247,874.54(207,051.49; 294,269.11) | 381,218.01(314,170.72; 449,546.29) | 1.54(-11.18; 16.08) | 131.15(109.87; 154.88) | 124.92(102.86; 147.64) | 0.15(-4.76; 5.32) |
| Iran (Islamic Republic of) | both | 95,317.03(80,470.09; 111,121.29) | 186,012.98(156,957.36; 216,144.49) | 2.62(-9.57; 16.46) | 185.84(157.44; 217.68) | 200.93(171.25; 230.69) | 0.45(-4.95; 6.16) |
| Iraq | both | 21,691.79(18,108.82; 25,724.46) | 56,089.41(47,093.37; 66,755.48) | 3.26(-7.85; 15.71) | 143.68(119.92; 170.73) | 129.19(107.96; 153.92) | -0.16(-5.09; 5.03) |
| Ireland | both | 12,153.89(10,119.38; 14,338.17) | 15,612.61(13,340.14; 18,040.54) | 1.24(-8.44; 11.96) | 330.81(275.00; 391.20) | 332.36(285.03; 380.24) | 0.28(-5.60; 6.53) |
| Israel | both | 13,339.64(11,073.28; 15,889.20) | 21,008.42(17,369.97; 25,163.14) | 1.88(-8.15; 13.01) | 273.03(226.04; 325.54) | 229.72(189.86; 276.96) | -0.08(-5.59; 5.75) |
| Italy | both | 183,432.53(152,911.81; 221,671.13) | 129,112.92(107,184.21; 151,356.52) | -0.73(-12.19; 12.23) | 324.00(267.71; 400.72) | 252.43(209.57; 299.14) | -0.55(-6.12; 5.36) |
| Jamaica | both | 5,312.65(4,287.17; 6,658.89) | 5,909.56(4,856.35; 6,956.54) | 0.59(-8.12; 10.12) | 214.34(175.15; 261.46) | 200.37(163.85; 237.74) | -0.04(-5.41; 5.64) |
| Japan | both | 288,431.34(234,627.20; 348,365.29) | 207,715.97(171,972.48; 247,140.30) | -0.77(-12.66; 12.75) | 220.73(179.20; 266.52) | 199.69(164.06; 241.59) | 0.01(-5.36; 5.69) |
| Jordan | both | 4,843.53(3,990.42; 5,821.10) | 17,124.72(14,286.42; 20,464.27) | 4.81(-5.30; 16.00) | 141.73(115.55; 170.71) | 126.83(105.25; 152.06) | 0.16(-4.77; 5.35) |
| Kazakhstan | both | 36,479.27(30,153.83; 43,068.55) | 44,527.92(38,094.58; 51,851.02) | 1.00(-9.66; 12.91) | 218.82(181.53; 257.56) | 234.72(201.48; 272.60) | 0.36(-5.19; 6.23) |
| Kenya | both | 18,297.02(15,272.03; 21,812.63) | 43,656.39(35,796.44; 52,344.07) | 3.59(-7.32; 15.77) | 95.95(80.60; 113.50) | 86.25(71.30; 101.66) | 0.28(-4.28; 5.05) |
| Kiribati | both | 134.61(109.75; 170.31) | 218.85(179.75; 262.24) | 1.91(-3.65; 7.79) | 180.82(148.62; 224.07) | 172.29(142.90; 203.84) | 0.04(-5.19; 5.55) |
| Kuwait | both | 3,105.31(2,505.44; 3,757.89) | 8,843.06(7,093.56; 10,806.28) | 4.53(-4.91; 14.90) | 153.73(124.99; 184.14) | 150.77(124.88; 178.21) | 0.28(-4.83; 5.67) |
| Kyrgyzstan | both | 7,336.27(6,124.85; 8,682.56) | 11,335.90(9,513.75; 13,346.87) | 1.68(-7.74; 12.06) | 175.14(145.87; 205.81) | 164.98(139.12; 193.72) | 0.04(-5.14; 5.50) |
| Lao People's Democratic Republic | both | 5,718.37(4,694.29; 7,095.79) | 11,216.82(9,238.52; 13,501.91) | 2.47(-7.01; 12.93) | 150.06(123.89; 180.05) | 141.88(116.42; 171.27) | 0.05(-4.99; 5.35) |
| Latvia | both | 8,341.98(7,120.71; 9,622.21) | 4,021.57(3,453.70; 4,664.26) | -1.96(-10.08; 6.90) | 321.46(274.63; 370.69) | 249.90(215.39; 284.91) | -0.42(-5.98; 5.47) |
| Lebanon | both | 4,902.96(4,077.22; 5,824.13) | 9,945.14(8,344.16; 11,662.09) | 2.93(-6.49; 13.30) | 165.64(136.93; 197.22) | 166.64(140.35; 193.43) | 0.62(-4.61; 6.13) |
| Lesotho | both | 2,662.69(2,226.43; 3,188.75) | 2,541.59(2,129.47; 3,011.89) | 0.64(-7.25; 9.21) | 157.42(132.37; 185.17) | 131.39(110.07; 153.69) | 0.03(-4.93; 5.24) |
| Liberia | both | 1,865.04(1,546.45; 2,240.37) | 5,470.19(4,477.00; 6,590.99) | 3.99(-4.91; 13.73) | 116.87(97.33; 137.87) | 102.54(84.56; 121.50) | 0.12(-4.60; 5.07) |
| Libya | both | 6,178.18(5,138.06; 7,390.63) | 14,071.79(11,590.68; 16,865.88) | 3.72(-6.11; 14.57) | 173.01(144.16; 206.64) | 171.09(141.70; 203.20) | 0.70(-4.55; 6.25) |
| Lithuania | both | 9,119.53(7,624.03; 10,786.16) | 6,629.51(5,720.82; 7,593.97) | -0.95(-9.63; 8.56) | 243.72(202.46; 287.83) | 273.86(238.26; 313.80) | 0.49(-5.21; 6.54) |
| Luxembourg | both | 1,281.10(1,053.62; 1,527.77) | 2,034.64(1,718.29; 2,345.77) | 1.76(-5.99; 10.16) | 327.49(270.81; 392.66) | 322.82(274.13; 369.32) | 0.13(-5.72; 6.34) |
| Madagascar | both | 12,266.06(9,901.84; 15,848.57) | 28,279.99(22,499.52; 35,568.37) | 3.09(-7.34; 14.69) | 115.55(94.61; 141.05) | 101.36(82.12; 123.19) | -0.07(-4.77; 4.86) |
| Malawi | both | 9,729.98(7,810.35; 12,499.91) | 19,293.62(15,329.33; 24,508.60) | 2.62(-7.40; 13.71) | 114.30(93.35; 139.56) | 100.29(82.00; 121.75) | -0.06(-4.74; 4.86) |
| Malaysia | both | 32,430.91(26,945.52; 38,570.64) | 53,637.22(44,262.88; 63,383.62) | 2.02(-8.92; 14.28) | 182.91(152.03; 216.48) | 152.58(127.21; 179.50) | -0.18(-5.28; 5.19) |
| Maldives | both | 325.94(268.23; 398.93) | 1,025.42(830.91; 1,237.51) | 4.13(-3.12; 11.91) | 168.09(139.35; 201.39) | 161.31(132.95; 193.37) | 0.06(-5.10; 5.50) |
| Mali | both | 7,292.70(6,027.88; 8,734.49) | 17,889.80(14,747.40; 21,445.21) | 3.48(-6.54; 14.57) | 105.28(86.97; 125.56) | 92.22(76.07; 109.96) | 0.02(-4.59; 4.84) |
| Malta | both | 936.44(758.17; 1,128.33) | 1,004.13(827.43; 1,199.58) | 0.54(-6.45; 8.05) | 244.22(199.66; 291.93) | 256.21(215.41; 300.47) | 0.44(-5.19; 6.42) |
| Marshall Islands | both | 87.93(69.62; 114.83) | 116.36(94.23; 141.97) | 0.94(-3.94; 6.07) | 207.24(171.50; 252.51) | 190.74(156.08; 230.74) | -0.03(-5.35; 5.60) |
| Mauritania | both | 2,035.52(1,689.39; 2,431.30) | 3,778.97(3,133.23; 4,496.71) | 2.63(-5.79; 11.82) | 117.60(97.89; 139.81) | 98.44(81.72; 116.24) | 0.06(-4.61; 4.97) |
| Mauritius | both | 2,176.66(1,788.87; 2,612.16) | 2,811.01(2,370.04; 3,359.28) | 0.97(-7.06; 9.70) | 180.53(149.73; 216.24) | 216.96(182.50; 261.78) | 0.76(-4.74; 6.58) |
| Mexico | both | 127,439.26(106,995.46; 150,340.44) | 184,185.13(154,534.85; 215,257.92) | 1.68(-10.38; 15.37) | 154.03(129.19; 181.49) | 133.85(112.31; 155.56) | 0.10(-4.88; 5.34) |
| Micronesia (Federated States of) | both | 209.38(167.15; 271.84) | 199.96(161.03; 248.11) | -0.08(-5.45; 5.60) | 209.52(173.67; 254.91) | 180.43(146.99; 218.92) | -0.22(-5.48; 5.33) |
| Monaco | both | 83.88(68.04; 102.37) | 91.83(76.52; 107.69) | 0.67(-3.97; 5.54) | 306.71(248.18; 373.05) | 296.40(245.67; 356.50) | 0.21(-5.57; 6.34) |
| Mongolia | both | 3,397.81(2,794.73; 4,155.34) | 5,562.04(4,650.48; 6,521.82) | 2.16(-6.62; 11.75) | 165.13(136.14; 197.01) | 167.75(140.57; 196.33) | 0.38(-4.84; 5.88) |
| Montenegro | both | 1,421.10(1,169.53; 1,700.60) | 1,045.89(859.68; 1,242.63) | -0.46(-7.42; 7.03) | 219.22(180.61; 262.26) | 171.14(140.49; 203.25) | -0.23(-5.44; 5.27) |
| Morocco | both | 36,416.15(30,342.59; 43,471.43) | 56,213.61(46,846.21; 65,314.87) | 1.77(-9.19; 14.06) | 151.19(125.10; 179.71) | 143.89(120.19; 166.97) | 0.23(-4.83; 5.56) |
| Mozambique | both | 12,874.17(10,356.07; 16,553.31) | 31,127.06(24,928.24; 39,073.04) | 3.19(-7.34; 14.91) | 112.49(92.65; 136.39) | 111.99(91.35; 133.78) | 0.38(-4.43; 5.44) |
| Myanmar | both | 65,969.82(54,503.25; 79,399.36) | 89,376.14(72,473.32; 109,098.78) | 1.11(-10.21; 13.86) | 160.04(133.07; 191.50) | 150.76(122.37; 183.85) | 0.00(-5.10; 5.36) |
| Namibia | both | 2,438.06(2,046.79; 2,895.37) | 3,553.32(2,959.46; 4,192.53) | 1.76(-6.55; 10.80) | 181.81(153.31; 212.99) | 141.00(117.61; 165.36) | -0.21(-5.23; 5.07) |
| Nauru | both | 20.32(16.46; 25.68) | 21.17(17.02; 26.48) | 0.18(-2.95; 3.41) | 203.28(167.77; 252.55) | 181.57(148.66; 222.26) | -0.14(-5.41; 5.43) |
| Nepal | both | 22,264.75(18,000.11; 26,773.98) | 37,835.74(30,935.99; 45,439.79) | 2.14(-8.48; 13.99) | 128.39(105.05; 153.35) | 116.05(95.99; 137.69) | 0.07(-4.76; 5.15) |
| Netherlands | both | 46,586.68(38,678.05; 55,308.81) | 38,768.37(32,772.50; 45,542.85) | -0.22(-10.64; 11.40) | 307.07(256.73; 359.95) | 254.29(216.93; 299.97) | -0.23(-5.84; 5.71) |
| New Zealand | both | 14,072.16(11,559.73; 16,929.99) | 19,343.68(16,242.91; 22,853.93) | 0.85(-8.99; 11.75) | 394.05(324.31; 474.54) | 402.36(337.68; 483.62) | 0.15(-5.90; 6.59) |
| Nicaragua | both | 5,552.88(4,639.26; 6,512.47) | 8,711.67(7,100.01; 10,359.46) | 1.83(-7.35; 11.92) | 162.84(135.97; 192.27) | 123.39(101.19; 147.01) | -0.47(-5.34; 4.65) |
| Niger | both | 6,716.20(5,547.31; 8,012.32) | 17,467.68(14,495.48; 21,100.00) | 3.59(-6.41; 14.67) | 107.01(88.45; 127.86) | 90.93(75.51; 108.49) | 0.04(-4.56; 4.85) |
| Nigeria | both | 77,262.54(65,249.04; 90,911.82) | 179,657.43(150,415.86; 212,240.72) | 3.35(-8.89; 17.23) | 100.25(84.82; 118.33) | 91.75(76.79; 107.69) | 0.31(-4.31; 5.15) |
| Niue | both | 4.49(3.65; 5.71) | 3.12(2.55; 3.82) | -1.21(-2.40; -0.01) | 206.73(170.32; 258.88) | 190.42(154.29; 231.56) | 0.00(-5.32; 5.63) |
| North Macedonia | both | 3,451.94(2,804.93; 4,173.82) | 3,505.05(2,868.47; 4,164.80) | 0.47(-7.73; 9.39) | 163.47(133.04; 197.55) | 156.12(128.87; 184.03) | 0.22(-4.92; 5.64) |
| Northern Mariana Islands | both | 113.80(92.69; 137.75) | 96.23(78.99; 116.90) | -1.00(-5.65; 3.89) | 210.32(172.80; 257.41) | 196.20(159.78; 238.28) | -0.06(-5.40; 5.60) |
| Norway | both | 13,543.73(11,047.08; 16,388.97) | 17,482.09(14,912.84; 20,423.94) | 1.04(-8.73; 11.86) | 310.59(254.16; 373.08) | 330.72(281.11; 384.56) | 0.33(-5.55; 6.58) |
| Oman | both | 2,457.90(1,982.11; 3,012.69) | 7,220.02(5,809.36; 8,788.89) | 4.50(-4.74; 14.62) | 133.07(108.56; 162.85) | 126.45(104.45; 149.99) | 0.20(-4.73; 5.38) |
| Pakistan | both | 149,061.33(123,070.70; 178,141.14) | 276,248.51(226,900.73; 327,962.98) | 2.59(-9.95; 16.89) | 151.44(126.57; 177.08) | 117.47(97.87; 138.61) | -0.22(-5.05; 4.86) |
| Palau | both | 35.11(28.52; 43.49) | 34.76(28.49; 41.58) | -0.17(-3.82; 3.62) | 207.47(170.42; 255.77) | 190.07(153.67; 231.77) | -0.05(-5.37; 5.56) |
| Palestine | both | 2,412.44(1,964.56; 2,913.11) | 7,038.60(5,814.70; 8,356.75) | 3.83(-5.32; 13.86) | 141.13(114.53; 171.88) | 132.97(109.64; 158.76) | 0.11(-4.87; 5.34) |
| Panama | both | 4,572.89(3,769.04; 5,500.10) | 6,717.29(5,454.85; 8,027.29) | 1.65(-7.26; 11.42) | 186.07(155.15; 221.22) | 153.60(124.29; 184.17) | -0.19(-5.29; 5.18) |
| Papua New Guinea | both | 7,468.13(5,970.61; 9,770.40) | 18,559.32(14,801.90; 23,158.28) | 3.27(-6.77; 14.39) | 180.08(147.33; 225.06) | 172.16(140.02; 212.43) | 0.03(-5.20; 5.54) |
| Paraguay | both | 6,827.89(5,661.14; 8,038.69) | 10,473.08(8,697.21; 12,289.49) | 2.07(-7.31; 12.41) | 177.13(148.78; 208.96) | 136.14(113.05; 159.40) | -0.21(-5.19; 5.05) |
| Peru | both | 45,709.34(38,030.28; 53,440.64) | 55,811.25(46,745.52; 65,445.39) | 1.41(-9.50; 13.64) | 208.38(175.84; 241.98) | 145.10(121.44; 169.98) | -0.29(-5.33; 5.03) |
| Philippines | both | 105,050.39(88,577.96; 124,691.16) | 160,896.09(133,310.67; 190,797.27) | 1.67(-10.26; 15.19) | 166.05(140.80; 193.78) | 133.34(110.60; 156.90) | -0.44(-5.39; 4.77) |
| Poland | both | 80,316.17(66,751.61; 96,568.31) | 65,220.82(53,745.25; 77,179.59) | -0.36(-11.23; 11.85) | 212.83(176.29; 257.44) | 189.07(155.82; 225.65) | 0.03(-5.28; 5.64) |
| Portugal | both | 26,157.93(21,388.01; 31,113.92) | 20,004.91(16,401.18; 23,731.08) | -0.42(-10.19; 10.42) | 256.82(209.70; 307.22) | 210.47(174.54; 246.85) | -0.16(-5.57; 5.57) |
| Puerto Rico | both | 11,747.77(9,842.75; 14,001.77) | 8,127.86(6,889.31; 9,642.35) | -0.89(-9.78; 8.87) | 316.48(266.09; 375.46) | 263.75(219.01; 318.10) | -0.36(-5.99; 5.60) |
| Qatar | both | 832.97(670.77; 1,024.01) | 5,396.16(4,283.82; 6,719.93) | 8.27(-1.03; 18.45) | 152.98(126.47; 183.08) | 129.62(106.70; 154.89) | -0.15(-5.09; 5.05) |
| Republic of Korea | both | 116,621.69(93,799.66; 143,168.64) | 103,830.92(84,624.17; 124,886.00) | 0.09(-11.27; 12.91) | 227.48(182.92; 279.20) | 213.28(173.32; 261.87) | 0.08(-5.36; 5.84) |
| Republic of Moldova | both | 10,304.76(8,593.80; 12,052.43) | 6,444.78(5,331.81; 7,673.83) | -0.99(-9.64; 8.49) | 230.45(191.98; 269.33) | 178.35(149.85; 207.82) | -0.35(-5.59; 5.18) |
| Romania | both | 38,084.62(30,698.87; 45,833.29) | 27,101.45(21,944.06; 32,782.18) | -0.79(-10.80; 10.35) | 161.11(130.41; 194.30) | 143.39(117.66; 171.11) | 0.03(-5.02; 5.34) |
| Russian Federation | both | 501,381.27(425,647.80; 582,749.17) | 397,455.19(342,434.66; 458,328.60) | -0.60(-13.10; 13.69) | 329.71(280.16; 383.24) | 296.27(256.86; 339.41) | -0.20(-5.95; 5.89) |
| Rwanda | both | 7,550.88(5,953.39; 9,854.94) | 15,807.98(12,418.16; 19,963.23) | 3.34(-6.56; 14.28) | 118.08(96.14; 146.16) | 113.47(91.46; 139.62) | 0.26(-4.57; 5.32) |
| Saint Kitts and Nevis | both | 84.47(67.35; 109.93) | 121.96(100.86; 146.34) | 1.55(-3.41; 6.77) | 206.51(168.78; 259.55) | 196.53(158.69; 244.63) | 0.02(-5.34; 5.68) |
| Saint Lucia | both | 378.24(295.76; 491.57) | 370.92(300.50; 456.46) | 0.55(-5.53; 7.03) | 255.20(204.46; 318.54) | 207.61(164.81; 266.34) | -0.05(-5.54; 5.76) |
| Saint Vincent and the Grenadines | both | 229.71(182.10; 300.82) | 228.70(186.42; 278.75) | 0.08(-5.43; 5.92) | 205.29(167.58; 255.78) | 201.05(161.57; 247.97) | 0.09(-5.30; 5.77) |
| Samoa | both | 364.54(282.60; 492.21) | 470.54(383.11; 565.14) | 0.97(-5.30; 7.66) | 211.12(171.33; 267.64) | 214.83(177.66; 253.30) | 0.13(-5.32; 5.89) |
| San Marino | both | 72.40(59.29; 86.73) | 82.99(70.37; 97.62) | 0.97(-3.58; 5.73) | 301.06(246.24; 365.82) | 285.13(236.89; 345.93) | 0.16(-5.57; 6.23) |
| Sao Tome and Principe | both | 115.42(95.53; 138.20) | 211.67(173.79; 254.09) | 2.55(-3.00; 8.42) | 119.65(99.12; 143.20) | 98.98(82.43; 118.30) | -0.10(-4.77; 4.80) |
| Saudi Arabia | both | 21,591.21(17,460.03; 26,021.18) | 67,598.00(54,756.43; 81,694.98) | 4.46(-6.96; 17.29) | 143.35(116.75; 172.23) | 139.46(114.68; 165.34) | 0.41(-4.63; 5.72) |
| Senegal | both | 7,230.49(5,997.19; 8,516.16) | 14,012.19(11,626.23; 16,581.96) | 2.60(-7.10; 13.32) | 117.27(97.50; 138.53) | 97.25(80.89; 115.52) | -0.10(-4.75; 4.78) |
| Serbia | both | 16,450.73(13,278.30; 19,866.14) | 14,159.22(11,523.27; 16,836.21) | -0.50(-9.94; 9.92) | 174.19(141.02; 210.79) | 162.80(133.71; 193.21) | 0.11(-5.07; 5.58) |
| Seychelles | both | 172.28(140.04; 215.77) | 193.44(158.40; 228.99) | 0.83(-4.56; 6.51) | 223.70(184.40; 273.79) | 181.25(148.63; 216.36) | -0.22(-5.48; 5.34) |
| Sierra Leone | both | 3,725.29(3,114.41; 4,423.48) | 7,831.71(6,529.29; 9,324.72) | 3.55(-5.67; 13.67) | 118.18(98.69; 139.77) | 95.67(79.76; 113.48) | -0.06(-4.70; 4.80) |
| Singapore | both | 8,519.40(6,780.12; 10,434.56) | 12,248.39(9,769.35; 14,825.59) | 1.72(-7.80; 12.22) | 232.16(185.57; 287.14) | 215.00(172.89; 263.74) | 0.01(-5.43; 5.77) |
| Slovakia | both | 11,987.03(9,950.93; 14,086.52) | 9,467.51(7,930.54; 11,175.56) | -0.54(-9.59; 9.42) | 223.52(186.71; 262.48) | 187.66(159.16; 218.25) | -0.24(-5.54; 5.35) |
| Slovenia | both | 4,588.52(3,733.12; 5,443.88) | 4,066.02(3,442.70; 4,746.81) | -0.12(-8.41; 8.92) | 232.22(188.18; 277.21) | 236.58(204.43; 271.33) | 0.40(-5.16; 6.29) |
| Solomon Islands | both | 617.83(484.47; 828.92) | 1,170.71(965.87; 1,400.72) | 2.24(-5.01; 10.04) | 186.56(152.52; 233.15) | 164.01(136.37; 192.72) | -0.14(-5.31; 5.31) |
| Somalia | both | 7,140.04(5,646.65; 9,387.14) | 18,778.01(14,651.07; 24,133.70) | 3.51(-6.56; 14.67) | 111.05(90.45; 136.27) | 93.68(75.99; 115.21) | -0.15(-4.76; 4.69) |
| South Africa | both | 115,227.85(97,415.66; 135,784.27) | 103,854.46(88,293.22; 119,979.36) | -0.23(-11.51; 12.49) | 288.41(246.30; 337.01) | 170.56(144.68; 197.27) | -1.52(-6.63; 3.87) |
| South Sudan | both | 6,488.83(5,207.29; 8,382.53) | 9,208.37(7,212.30; 11,954.05) | 1.55(-7.65; 11.66) | 120.38(97.42; 147.46) | 100.92(82.20; 123.14) | -0.12(-4.82; 4.80) |
| Spain | both | 142,553.89(125,001.53; 162,622.29) | 131,871.79(114,582.28; 152,937.65) | -0.03(-11.59; 13.05) | 364.46(319.24; 416.53) | 341.22(297.81; 384.91) | -0.13(-6.02; 6.13) |
| Sri Lanka | both | 34,621.66(28,954.08; 41,480.41) | 36,139.44(29,886.15; 42,838.06) | 0.31(-10.09; 11.90) | 190.79(160.68; 226.78) | 159.97(132.29; 190.28) | -0.30(-5.44; 5.12) |
| Sudan | both | 21,332.20(17,511.14; 25,676.12) | 57,041.60(47,455.69; 66,916.87) | 3.31(-7.82; 15.78) | 124.42(102.23; 149.44) | 132.59(109.68; 155.88) | 0.45(-4.54; 5.70) |
| Suriname | both | 753.59(612.53; 957.52) | 1,093.20(904.53; 1,296.18) | 1.69(-5.46; 9.39) | 190.95(155.07; 236.92) | 185.34(153.26; 220.02) | 0.17(-5.14; 5.78) |
| Sweden | both | 22,160.37(17,814.18; 27,044.95) | 28,009.47(23,688.22; 33,267.67) | 1.21(-9.04; 12.60) | 259.30(208.29; 316.05) | 296.81(251.02; 351.21) | 0.87(-4.94; 7.03) |
| Switzerland | both | 29,883.12(24,545.47; 36,048.68) | 27,908.54(23,542.32; 32,803.38) | 0.18(-9.96; 11.46) | 445.81(368.31; 537.57) | 353.22(300.02; 411.79) | -0.43(-6.34; 5.85) |
| Syrian Arab Republic | both | 12,985.20(10,614.00; 15,559.49) | 18,563.04(15,509.96; 21,937.32) | 1.57(-8.33; 12.53) | 124.48(101.64; 150.66) | 127.07(105.14; 151.63) | 0.55(-4.41; 5.76) |
| Taiwan (Province of China) | both | 49,738.53(40,343.42; 59,854.71) | 53,764.15(44,655.84; 64,608.59) | 0.55(-10.25; 12.64) | 222.37(181.93; 267.71) | 219.01(182.80; 259.40) | 0.24(-5.24; 6.03) |
| Tajikistan | both | 7,465.87(6,093.44; 9,001.35) | 15,061.86(12,569.79; 17,927.20) | 2.68(-7.10; 13.50) | 154.41(126.48; 184.48) | 146.21(122.00; 172.62) | 0.06(-5.00; 5.40) |
| Thailand | both | 116,100.66(96,154.82; 140,128.46) | 106,412.99(88,074.95; 124,574.99) | 0.22(-11.18; 13.08) | 180.24(149.25; 214.81) | 165.55(138.14; 193.87) | 0.06(-5.13; 5.54) |
| Timor-Leste | both | 1,188.10(980.41; 1,452.00) | 2,069.71(1,667.50; 2,539.48) | 2.08(-5.72; 10.52) | 155.46(128.74; 187.49) | 145.75(119.49; 175.17) | 0.10(-4.97; 5.43) |
| Togo | both | 3,015.48(2,493.97; 3,617.19) | 7,058.38(5,951.34; 8,323.63) | 3.47(-5.64; 13.46) | 102.82(84.85; 123.25) | 91.32(77.08; 107.44) | 0.29(-4.32; 5.12) |
| Tokelau | both | 2.89(2.33; 3.72) | 2.50(2.01; 3.10) | -0.46(-1.43; 0.53) | 194.81(160.04; 240.65) | 182.31(147.82; 222.16) | 0.07(-5.22; 5.65) |
| Tonga | both | 188.63(149.15; 248.92) | 194.88(158.66; 235.60) | 0.28(-5.08; 5.94) | 196.12(160.36; 242.20) | 185.95(153.23; 220.06) | 0.05(-5.26; 5.65) |
| Trinidad and Tobago | both | 2,430.51(1,944.16; 3,141.04) | 2,523.29(2,108.40; 2,972.91) | 0.31(-7.56; 8.84) | 195.89(158.99; 247.82) | 182.30(152.54; 215.33) | 0.12(-5.17; 5.71) |
| Tunisia | both | 10,759.20(8,753.30; 12,898.13) | 17,637.40(14,530.43; 21,130.80) | 2.03(-7.86; 12.97) | 137.04(110.93; 164.96) | 141.78(117.24; 169.16) | 0.56(-4.50; 5.89) |
| Turkey | both | 78,345.61(63,723.24; 94,468.23) | 111,010.21(90,637.03; 134,248.53) | 1.67(-9.92; 14.75) | 135.43(109.84; 164.78) | 123.64(101.21; 149.46) | 0.18(-4.72; 5.34) |
| Turkmenistan | both | 5,656.34(4,650.16; 6,759.84) | 9,185.60(7,672.30; 10,786.16) | 1.76(-7.47; 11.91) | 160.87(132.78; 191.69) | 170.94(142.79; 200.98) | 0.40(-4.84; 5.92) |
| Tuvalu | both | 17.05(13.91; 21.38) | 22.66(18.26; 27.90) | 1.22(-2.02; 4.56) | 187.58(153.73; 233.42) | 177.00(143.43; 215.99) | 0.01(-5.24; 5.55) |
| Uganda | both | 20,388.05(16,202.54; 26,679.52) | 44,925.55(36,389.62; 54,316.52) | 3.11(-7.77; 15.27) | 129.09(106.03; 158.21) | 108.32(89.77; 127.62) | -0.08(-4.84; 4.91) |
| Ukraine | both | 123,938.25(104,566.87; 145,080.46) | 90,757.16(77,443.68; 104,007.54) | -0.60(-11.75; 11.96) | 236.36(199.53; 276.44) | 223.21(191.24; 253.42) | 0.17(-5.31; 5.98) |
| United Arab Emirates | both | 4,302.29(3,435.93; 5,314.38) | 24,768.29(19,431.62; 30,978.85) | 7.29(-3.47; 19.26) | 192.63(158.19; 231.42) | 183.05(154.37; 214.72) | 0.31(-5.00; 5.91) |
| United Kingdom | both | 212,442.75(177,877.53; 255,149.44) | 235,230.00(201,314.42; 274,067.00) | 0.82(-11.38; 14.69) | 392.34(326.67; 477.79) | 390.75(329.91; 464.46) | 0.40(-5.66; 6.84) |
| United Republic of Tanzania | both | 30,464.83(24,726.54; 37,979.69) | 62,731.40(50,443.47; 78,904.02) | 2.71(-8.44; 15.22) | 128.72(106.80; 153.65) | 112.55(92.45; 136.34) | -0.04(-4.84; 5.00) |
| United States of America | both | 1104,273.86(938,952.70; 1297,546.90) | 1583,449.64(1384,480.18; 1793,912.27) | 1.63(-12.41; 17.91) | 438.19(373.14; 517.65) | 531.19(462.11; 605.02) | 1.05(-5.33; 7.87) |
| United States Virgin Islands | both | 278.96(228.86; 343.90) | 180.63(152.26; 216.53) | -0.75(-6.00; 4.79) | 249.78(204.89; 305.65) | 240.24(200.93; 295.40) | 0.13(-5.43; 6.02) |
| Uruguay | both | 8,398.73(7,055.75; 9,741.91) | 7,145.21(6,053.99; 8,316.38) | 0.11(-8.74; 9.81) | 275.53(231.10; 319.73) | 219.23(186.17; 255.97) | -0.10(-5.56; 5.67) |
| Uzbekistan | both | 29,692.24(24,060.74; 35,821.36) | 53,261.56(44,312.57; 62,803.90) | 2.36(-8.61; 14.65) | 151.74(124.00; 182.98) | 150.41(125.37; 176.30) | 0.29(-4.81; 5.67) |
| Vanuatu | both | 251.54(203.73; 322.68) | 519.23(432.17; 612.92) | 2.52(-3.94; 9.41) | 174.12(143.48; 212.68) | 163.05(136.59; 189.71) | -0.03(-5.20; 5.42) |
| Venezuela (Bolivarian Republic of) | both | 29,222.49(23,799.94; 34,915.62) | 35,517.70(29,368.85; 42,142.90) | 1.42(-9.08; 13.14) | 157.80(129.12; 187.64) | 130.70(107.93; 155.33) | -0.17(-5.12; 5.03) |
| Viet Nam | both | 116,182.93(96,755.76; 141,328.61) | 179,127.53(147,921.72; 210,830.50) | 1.71(-10.34; 15.37) | 173.03(144.42; 205.67) | 173.11(143.44; 203.38) | 0.23(-5.02; 5.76) |
| Yemen | both | 12,703.31(10,302.85; 15,502.20) | 40,444.64(33,244.46; 47,878.84) | 4.09(-6.79; 16.25) | 122.02(99.33; 146.74) | 126.03(104.33; 147.84) | 0.38(-4.56; 5.58) |
| Zambia | both | 9,511.21(7,864.93; 11,498.30) | 18,678.96(15,529.75; 22,577.09) | 2.92(-7.09; 14.01) | 131.94(111.67; 155.26) | 101.88(85.13; 120.37) | -0.29(-4.98; 4.64) |
| Zimbabwe | both | 18,195.41(14,992.22; 22,095.28) | 21,372.25(17,528.91; 25,817.73) | 0.74(-9.19; 11.76) | 196.46(164.30; 231.48) | 143.83(121.11; 169.25) | -0.57(-5.58; 4.71) |
| Afghanistan | female | 8,352.86(6,871.59; 10,085.78) | 21,427.02(18,096.36; 24,886.01) | 4.07(-6.19; 15.45) | 168.90(138.81; 201.66) | 154.67(131.02; 181.29) | 0.43(-4.71; 5.86) |
| Albania | female | 2,534.55(2,010.08; 3,130.28) | 1,902.29(1,549.24; 2,328.91) | -0.76(-8.27; 7.37) | 154.95(122.90; 189.55) | 144.14(117.95; 175.81) | 0.09(-4.96; 5.41) |
| Algeria | female | 14,004.34(11,324.39; 17,006.29) | 31,542.75(25,898.29; 38,219.92) | 3.18(-7.38; 14.94) | 132.39(105.63; 161.65) | 139.64(115.34; 167.06) | 0.60(-4.45; 5.92) |
| American Samoa | female | 44.45(36.31; 54.28) | 43.18(35.04; 53.00) | 0.28(-3.59; 4.30) | 188.66(155.67; 227.86) | 176.49(144.05; 213.69) | 0.11(-5.15; 5.65) |
| Andorra | female | 74.22(57.95; 92.28) | 104.30(83.77; 129.17) | 1.14(-3.65; 6.17) | 252.77(197.80; 313.66) | 245.58(197.69; 298.69) | 0.23(-5.36; 6.14) |
| Angola | female | 4,496.37(3,628.50; 5,439.30) | 13,654.68(11,093.90; 16,636.25) | 4.09(-5.72; 14.93) | 103.86(83.44; 124.97) | 94.15(76.18; 114.32) | 0.10(-4.53; 4.96) |
| Antigua and Barbuda | female | 53.52(43.14; 65.07) | 77.79(62.66; 93.73) | 1.43(-3.09; 6.17) | 167.70(135.18; 202.65) | 161.33(128.61; 197.84) | 0.10(-5.09; 5.56) |
| Argentina | female | 30,267.13(24,173.88; 37,297.36) | 41,525.48(33,719.33; 50,613.39) | 1.42(-9.22; 13.30) | 185.63(147.69; 229.86) | 171.65(140.02; 207.94) | 0.11(-5.12; 5.62) |
| Armenia | female | 2,561.10(2,054.51; 3,110.82) | 2,324.56(1,892.28; 2,777.43) | -0.16(-7.91; 8.25) | 144.71(116.96; 176.90) | 151.24(126.49; 179.71) | 0.26(-4.85; 5.64) |
| Australia | female | 41,796.32(34,453.91; 50,003.57) | 50,562.61(40,832.60; 61,625.33) | 0.93(-9.84; 12.98) | 478.39(397.38; 569.02) | 400.37(325.94; 486.67) | -0.17(-6.22; 6.28) |
| Austria | female | 9,114.31(7,263.74; 11,084.71) | 10,487.17(8,689.44; 12,773.34) | 0.78(-8.49; 10.98) | 229.42(184.47; 276.82) | 252.08(209.59; 301.64) | 0.59(-5.04; 6.56) |
| Azerbaijan | female | 5,251.03(4,212.19; 6,438.10) | 8,123.75(6,620.80; 9,704.85) | 1.71(-7.39; 11.71) | 140.21(111.93; 172.21) | 143.33(117.81; 171.19) | 0.21(-4.84; 5.53) |
| Bahamas | female | 271.83(222.09; 327.55) | 397.03(323.53; 479.43) | 1.46(-4.68; 8.00) | 191.24(156.91; 228.73) | 182.16(148.50; 221.75) | 0.08(-5.21; 5.66) |
| Bahrain | female | 298.46(234.68; 374.23) | 852.69(689.18; 1,043.68) | 4.68(-2.43; 12.32) | 136.04(108.08; 167.93) | 131.67(106.89; 160.88) | 0.39(-4.59; 5.63) |
| Bangladesh | female | 65,504.17(53,128.99; 80,062.03) | 121,139.71(99,320.96; 144,637.81) | 2.57(-9.20; 15.87) | 150.32(122.76; 181.53) | 135.75(111.67; 161.84) | 0.17(-4.83; 5.44) |
| Barbados | female | 280.69(229.48; 340.38) | 259.79(213.40; 310.43) | 0.16(-5.48; 6.14) | 209.07(170.19; 253.48) | 182.94(147.91; 219.74) | -0.16(-5.44; 5.41) |
| Belarus | female | 11,732.01(9,537.10; 14,068.25) | 9,874.15(8,035.45; 11,938.11) | -0.32(-9.44; 9.71) | 217.11(177.47; 260.15) | 203.46(166.21; 243.77) | 0.04(-5.35; 5.74) |
| Belgium | female | 12,567.13(10,164.33; 15,104.48) | 12,827.26(10,618.85; 15,448.11) | 0.38(-9.04; 10.78) | 257.29(209.17; 308.15) | 241.21(200.64; 287.46) | 0.09(-5.47; 5.98) |
| Belize | female | 160.63(129.77; 196.34) | 375.75(308.29; 454.04) | 3.15(-3.02; 9.72) | 177.13(146.15; 209.35) | 156.04(128.45; 187.00) | -0.14(-5.27; 5.26) |
| Benin | female | 1,916.78(1,522.21; 2,347.12) | 5,243.49(4,378.65; 6,266.73) | 3.68(-5.16; 13.33) | 94.84(75.31; 116.05) | 87.00(71.53; 105.21) | 0.14(-4.42; 4.91) |
| Bermuda | female | 63.29(51.00; 76.46) | 51.45(41.82; 62.08) | -0.34(-4.36; 3.85) | 190.64(154.69; 232.15) | 175.63(142.56; 215.38) | 0.05(-5.20; 5.58) |
| Bhutan | female | 325.24(262.65; 396.93) | 463.44(377.81; 555.04) | 2.13(-4.20; 8.87) | 123.06(99.49; 149.26) | 115.00(93.67; 137.63) | 0.32(-4.52; 5.41) |
| Bolivia (Plurinational State of) | female | 5,499.79(4,630.40; 6,360.93) | 8,934.98(7,438.06; 10,444.44) | 2.12(-7.10; 12.27) | 181.05(152.27; 210.62) | 145.42(120.74; 169.81) | -0.22(-5.26; 5.09) |
| Bosnia and Herzegovina | female | 3,468.62(2,710.43; 4,323.45) | 2,148.86(1,682.61; 2,654.78) | -0.93(-8.55; 7.33) | 144.19(112.88; 179.38) | 132.38(104.00; 161.67) | 0.16(-4.81; 5.39) |
| Botswana | female | 879.41(710.36; 1,057.81) | 1,488.48(1,208.80; 1,807.98) | 2.47(-5.04; 10.56) | 141.25(115.59; 170.46) | 113.50(93.00; 137.14) | 0.06(-4.76; 5.11) |
| Brazil | female | 144,240.60(120,585.22; 173,248.70) | 182,719.77(153,762.12; 214,455.37) | 1.21(-10.80; 14.84) | 182.45(153.26; 216.61) | 158.17(133.49; 184.96) | -0.02(-5.16; 5.41) |
| Brunei Darussalam | female | 294.99(233.14; 368.35) | 513.91(414.74; 628.21) | 1.98(-4.44; 8.84) | 227.77(181.49; 282.38) | 211.14(169.48; 259.40) | -0.05(-5.47; 5.69) |
| Bulgaria | female | 7,165.95(5,782.35; 8,744.97) | 4,972.82(4,102.51; 6,053.93) | -0.89(-9.30; 8.30) | 166.70(134.80; 201.80) | 162.67(136.16; 194.78) | 0.27(-4.90; 5.72) |
| Burkina Faso | female | 3,544.29(2,839.66; 4,399.39) | 8,030.25(6,561.17; 9,663.01) | 3.37(-5.86; 13.51) | 89.79(71.63; 112.19) | 79.34(64.17; 96.39) | 0.05(-4.41; 4.71) |
| Burundi | female | 2,375.04(1,919.52; 2,918.51) | 4,864.85(3,900.10; 6,108.43) | 2.93(-5.77; 12.43) | 97.80(79.70; 119.49) | 80.46(65.04; 98.62) | -0.17(-4.63; 4.50) |
| Cabo Verde | female | 157.82(128.21; 190.65) | 273.28(219.40; 332.88) | 2.40(-3.41; 8.56) | 105.16(84.77; 127.92) | 92.06(73.74; 111.59) | 0.10(-4.51; 4.93) |
| Cambodia | female | 6,643.28(5,430.37; 8,085.79) | 11,166.14(9,191.81; 13,314.88) | 2.11(-7.33; 12.52) | 134.62(110.78; 163.13) | 123.37(101.72; 147.30) | 0.06(-4.84; 5.21) |
| Cameroon | female | 4,592.30(3,721.68; 5,540.31) | 12,671.92(10,325.90; 15,269.77) | 3.95(-5.78; 14.68) | 103.99(84.13; 124.96) | 88.36(72.43; 106.73) | 0.02(-4.54; 4.79) |
| Canada | female | 52,113.11(44,425.53; 61,309.08) | 60,389.84(52,342.99; 71,573.57) | 1.09(-9.86; 13.38) | 385.35(332.23; 445.44) | 378.36(329.87; 441.08) | 0.69(-5.36; 7.12) |
| Central African Republic | female | 1,227.26(1,008.31; 1,464.90) | 2,267.87(1,874.53; 2,737.79) | 2.31(-5.60; 10.87) | 100.66(82.87; 120.68) | 86.23(71.06; 102.49) | -0.14(-4.67; 4.60) |
| Chad | female | 2,357.67(1,916.61; 2,882.61) | 5,802.04(4,766.89; 7,063.62) | 3.34(-5.57; 13.08) | 95.68(77.00; 117.03) | 82.92(67.54; 101.66) | -0.02(-4.52; 4.69) |
| Chile | female | 14,462.69(11,801.63; 17,444.76) | 19,982.00(16,918.49; 23,480.07) | 1.27(-8.66; 12.27) | 204.30(166.32; 247.99) | 212.68(180.51; 247.56) | 0.35(-5.10; 6.11) |
| China | female | 1385,292.46(1165,704.38; 1619,959.71) | 1161,457.79(957,930.89; 1396,303.90) | -0.03(-13.57; 15.64) | 220.09(185.73; 257.47) | 165.00(136.37; 195.85) | -0.62(-5.78; 4.81) |
| Colombia | female | 26,808.16(22,343.12; 31,641.00) | 38,782.09(32,417.34; 45,812.93) | 1.37(-9.20; 13.17) | 158.86(131.78; 189.05) | 149.11(125.19; 176.05) | 0.07(-5.01; 5.43) |
| Comoros | female | 193.77(155.05; 242.62) | 322.82(260.98; 394.96) | 2.02(-3.93; 8.34) | 96.14(76.86; 119.38) | 84.26(67.97; 102.89) | 0.01(-4.51; 4.73) |
| Congo | female | 1,158.66(939.85; 1,386.90) | 2,770.36(2,274.95; 3,355.28) | 3.31(-4.87; 12.19) | 109.95(88.55; 132.45) | 101.35(83.23; 122.45) | 0.17(-4.54; 5.10) |
| Cook Islands | female | 17.89(14.62; 21.89) | 15.53(12.56; 18.86) | -0.15(-2.97; 2.75) | 194.86(159.72; 234.49) | 171.53(139.03; 209.60) | -0.06(-5.28; 5.44) |
| Costa Rica | female | 2,185.09(1,761.53; 2,657.26) | 3,493.75(2,849.61; 4,207.10) | 1.92(-6.39; 10.97) | 146.12(118.48; 177.97) | 133.23(109.50; 159.47) | 0.15(-4.83; 5.39) |
| Croatia | female | 4,739.31(3,827.68; 5,704.76) | 3,513.65(2,927.91; 4,223.36) | -0.66(-8.77; 8.17) | 193.49(156.74; 231.46) | 180.63(151.16; 215.94) | 0.17(-5.11; 5.75) |
| Cuba | female | 10,323.30(8,405.55; 12,626.09) | 8,020.21(6,512.71; 9,761.74) | -0.53(-9.44; 9.25) | 174.05(140.88; 213.18) | 153.97(124.56; 193.31) | -0.22(-5.33; 5.15) |
| Cyprus | female | 806.86(630.97; 994.31) | 1,372.72(1,074.69; 1,727.88) | 2.24(-5.16; 10.23) | 202.62(159.19; 249.07) | 180.49(144.90; 221.30) | -0.03(-5.29; 5.53) |
| Czechia | female | 11,455.57(9,302.94; 13,697.95) | 10,284.98(8,436.86; 12,268.05) | -0.10(-9.27; 10.00) | 221.31(180.95; 263.48) | 219.42(184.73; 258.73) | 0.41(-5.08; 6.21) |
| Côte d'Ivoire | female | 4,789.29(3,811.07; 5,934.90) | 10,460.76(8,464.86; 12,610.81) | 2.86(-6.58; 13.26) | 96.98(76.87; 121.40) | 85.01(68.78; 102.48) | 0.03(-4.50; 4.76) |
| Democratic People's Republic of Korea | female | 21,889.75(18,031.56; 25,885.82) | 22,882.18(18,699.16; 27,249.18) | 0.52(-9.47; 11.60) | 194.65(159.89; 229.67) | 162.98(133.04; 193.83) | -0.19(-5.35; 5.25) |
| Democratic Republic of the Congo | female | 16,032.85(12,939.92; 19,477.04) | 35,848.15(29,447.71; 43,595.93) | 3.11(-7.55; 15.00) | 99.45(80.28; 120.59) | 88.58(72.58; 107.88) | 0.03(-4.53; 4.82) |
| Denmark | female | 7,946.42(6,295.76; 9,847.14) | 7,457.40(6,069.83; 9,167.58) | -0.04(-8.91; 9.69) | 300.89(240.45; 368.78) | 272.70(222.93; 333.95) | -0.13(-5.80; 5.87) |
| Djibouti | female | 192.06(152.42; 239.49) | 530.09(419.60; 654.54) | 4.23(-2.35; 11.25) | 97.40(77.21; 121.15) | 84.86(67.54; 103.85) | 0.06(-4.46; 4.80) |
| Dominica | female | 71.07(56.49; 91.23) | 64.48(51.96; 78.80) | -0.12(-4.37; 4.32) | 196.18(158.43; 243.46) | 190.34(152.60; 233.82) | 0.02(-5.31; 5.64) |
| Dominican Republic | female | 4,770.76(3,843.38; 5,852.34) | 8,453.17(6,946.53; 10,249.67) | 1.95(-7.21; 12.03) | 132.44(106.52; 162.37) | 147.04(120.48; 178.87) | 0.48(-4.61; 5.85) |
| Ecuador | female | 7,710.60(6,356.79; 9,089.39) | 13,564.00(11,156.82; 16,100.26) | 2.28(-7.37; 12.94) | 157.93(130.20; 188.25) | 144.02(118.46; 170.72) | 0.14(-4.92; 5.47) |
| Egypt | female | 32,419.67(26,193.89; 39,495.31) | 64,861.95(53,102.95; 77,986.02) | 2.61(-8.57; 15.16) | 130.08(104.82; 159.88) | 127.50(104.97; 152.45) | 0.26(-4.68; 5.46) |
| El Salvador | female | 3,153.78(2,603.27; 3,797.58) | 4,242.68(3,456.42; 5,166.35) | 1.25(-7.17; 10.43) | 128.68(104.39; 155.04) | 119.06(96.68; 145.41) | 0.06(-4.78; 5.15) |
| Equatorial Guinea | female | 192.58(157.41; 230.78) | 683.48(554.35; 827.70) | 4.85(-2.03; 12.22) | 102.33(83.27; 122.92) | 100.94(81.75; 122.12) | 0.58(-4.15; 5.53) |
| Eritrea | female | 1,298.06(1,040.32; 1,608.87) | 2,660.05(2,148.76; 3,279.32) | 2.96(-5.15; 11.77) | 98.82(80.21; 121.70) | 82.99(67.11; 101.07) | -0.15(-4.64; 4.56) |
| Estonia | female | 1,960.43(1,615.68; 2,372.24) | 1,660.93(1,416.78; 1,953.21) | -0.40(-7.81; 7.60) | 246.69(204.25; 296.07) | 295.44(253.35; 341.81) | 0.60(-5.19; 6.74) |
| Eswatini | female | 556.57(459.59; 668.21) | 709.99(593.84; 844.34) | 1.17(-5.52; 8.32) | 147.13(121.49; 175.92) | 119.90(100.41; 143.49) | -0.15(-5.00; 4.95) |
| Ethiopia | female | 19,424.75(16,014.97; 23,402.47) | 40,035.49(32,866.04; 47,889.78) | 2.65(-8.07; 14.61) | 88.32(72.74; 105.57) | 75.83(62.23; 90.14) | -0.12(-4.52; 4.48) |
| Fiji | female | 690.10(567.15; 846.15) | 711.76(581.87; 858.53) | 0.43(-6.21; 7.55) | 178.03(147.10; 215.91) | 153.77(125.78; 185.36) | -0.13(-5.24; 5.25) |
| Finland | female | 7,181.58(5,695.51; 9,042.07) | 6,845.79(5,680.55; 8,235.54) | -0.02(-8.81; 9.61) | 274.46(219.67; 343.65) | 275.43(229.95; 331.41) | 0.20(-5.50; 6.24) |
| France | female | 74,678.39(59,523.64; 90,715.85) | 79,508.25(66,733.54; 94,588.33) | 0.48(-10.67; 13.02) | 260.54(208.11; 314.17) | 255.70(213.66; 303.29) | 0.30(-5.33; 6.27) |
| Gabon | female | 496.60(408.64; 592.49) | 941.76(766.19; 1,133.95) | 2.58(-4.48; 10.15) | 118.37(96.53; 141.91) | 101.05(81.42; 121.42) | -0.12(-4.81; 4.80) |
| Gambia | female | 421.90(341.97; 515.56) | 948.41(773.74; 1,156.59) | 2.98(-4.10; 10.59) | 102.34(82.40; 125.66) | 84.78(68.95; 101.86) | -0.16(-4.67; 4.57) |
| Georgia | female | 4,142.77(3,326.61; 5,073.63) | 2,544.52(2,084.35; 3,101.13) | -1.40(-9.14; 7.00) | 142.33(114.26; 175.11) | 146.13(120.06; 175.47) | 0.26(-4.81; 5.60) |
| Germany | female | 93,699.83(74,172.41; 115,775.20) | 90,224.37(72,777.83; 112,331.13) | 0.03(-11.18; 12.66) | 236.19(190.33; 290.42) | 243.68(198.96; 298.92) | 0.32(-5.26; 6.23) |
| Ghana | female | 6,998.40(5,661.02; 8,488.66) | 15,337.89(12,595.91; 18,615.10) | 3.29(-6.57; 14.18) | 105.60(85.31; 129.00) | 87.71(71.43; 105.72) | 0.02(-4.54; 4.80) |
| Greece | female | 9,954.86(7,628.42; 12,543.79) | 8,756.43(6,874.08; 11,085.66) | -0.21(-9.23; 9.70) | 189.29(146.00; 239.13) | 182.32(145.65; 226.17) | 0.09(-5.19; 5.67) |
| Greenland | female | 84.65(69.04; 100.31) | 77.20(65.10; 90.52) | 0.27(-4.17; 4.92) | 316.64(259.61; 374.88) | 300.46(251.77; 354.72) | 0.31(-5.48; 6.45) |
| Grenada | female | 73.79(58.75; 92.18) | 90.59(74.22; 110.68) | 0.85(-3.82; 5.74) | 181.89(147.68; 220.80) | 174.85(141.89; 217.06) | 0.14(-5.13; 5.71) |
| Guam | female | 136.95(111.80; 165.22) | 130.65(106.23; 158.71) | 0.16(-4.81; 5.39) | 201.88(165.40; 241.81) | 171.74(139.03; 209.27) | -0.19(-5.40; 5.31) |
| Guatemala | female | 5,386.14(4,357.61; 6,533.91) | 11,224.77(9,252.46; 13,327.20) | 3.19(-6.33; 13.68) | 163.55(130.31; 200.79) | 133.42(109.55; 159.80) | -0.35(-5.28; 4.84) |
| Guinea | female | 2,445.06(1,967.26; 3,000.39) | 5,011.77(4,028.72; 6,132.27) | 2.60(-6.10; 12.10) | 92.18(74.40; 114.46) | 82.34(66.44; 100.76) | -0.01(-4.50; 4.70) |
| Guinea-Bissau | female | 397.16(319.48; 487.37) | 792.39(647.63; 968.52) | 2.65(-4.24; 10.03) | 91.39(72.84; 112.04) | 82.02(66.37; 100.15) | 0.01(-4.48; 4.71) |
| Guyana | female | 572.73(468.25; 694.01) | 567.06(462.88; 685.48) | 0.16(-6.25; 7.00) | 143.87(117.79; 174.27) | 140.34(113.84; 170.72) | 0.10(-4.93; 5.39) |
| Haiti | female | 4,215.84(3,393.41; 5,186.64) | 9,807.84(7,976.40; 12,066.43) | 2.78(-6.57; 13.08) | 137.10(110.95; 166.53) | 139.76(114.22; 171.04) | 0.08(-4.92; 5.35) |
| Honduras | female | 2,658.00(2,165.43; 3,190.43) | 6,812.30(5,498.29; 8,260.94) | 3.34(-5.72; 13.26) | 132.20(107.36; 160.26) | 124.49(100.51; 150.65) | 0.01(-4.88; 5.16) |
| Hungary | female | 9,276.97(7,540.04; 11,131.32) | 7,050.42(5,701.93; 8,562.66) | -0.42(-9.21; 9.21) | 177.32(144.10; 212.19) | 155.84(127.34; 189.04) | 0.15(-4.98; 5.56) |
| Iceland | female | 347.62(277.90; 426.81) | 499.34(421.01; 598.22) | 1.28(-5.06; 8.06) | 268.77(214.43; 328.50) | 298.38(251.98; 355.18) | 0.42(-5.37; 6.56) |
| India | female | 524,651.50(433,092.38; 622,577.16) | 867,787.10(722,115.91; 1021,265.21) | 2.20(-11.36; 17.84) | 133.77(111.01; 158.25) | 116.23(96.55; 136.24) | 0.04(-4.79; 5.12) |
| Indonesia | female | 125,509.34(104,300.65; 149,855.99) | 177,619.52(145,403.55; 210,075.20) | 1.34(-10.65; 14.94) | 131.81(109.82; 156.52) | 117.92(96.89; 139.61) | 0.02(-4.83; 5.12) |
| Iran (Islamic Republic of) | female | 36,894.42(30,463.27; 44,172.73) | 71,349.92(58,297.61; 86,802.52) | 2.73(-8.57; 15.42) | 151.47(124.36; 182.33) | 151.56(125.88; 179.75) | 0.29(-4.82; 5.68) |
| Iraq | female | 9,391.24(7,680.87; 11,312.96) | 25,706.76(21,017.03; 30,798.01) | 3.44(-6.93; 14.97) | 132.54(107.13; 162.31) | 125.51(102.35; 150.21) | -0.07(-4.98; 5.09) |
| Ireland | female | 5,231.03(4,268.81; 6,317.83) | 7,127.13(5,769.35; 8,671.82) | 1.44(-7.51; 11.26) | 290.01(235.74; 352.45) | 289.28(237.59; 347.22) | 0.26(-5.49; 6.35) |
| Israel | female | 5,520.15(4,417.28; 6,705.48) | 8,863.01(7,095.44; 10,721.20) | 1.85(-7.35; 11.96) | 225.44(180.65; 273.32) | 194.55(156.06; 237.94) | -0.09(-5.42; 5.55) |
| Italy | female | 76,276.97(62,459.06; 92,280.26) | 56,523.30(46,241.70; 67,840.32) | -0.58(-11.30; 11.44) | 267.93(218.93; 327.66) | 214.27(174.76; 256.29) | -0.47(-5.88; 5.26) |
| Jamaica | female | 2,139.53(1,740.19; 2,647.50) | 2,560.60(2,087.91; 3,038.86) | 0.71(-7.20; 9.30) | 175.05(143.14; 212.45) | 171.63(139.08; 204.47) | 0.05(-5.18; 5.56) |
| Japan | female | 140,908.51(113,310.65; 172,228.97) | 106,725.16(87,808.20; 128,059.68) | -0.64(-11.94; 12.10) | 214.85(173.33; 261.61) | 202.27(164.46; 244.38) | 0.08(-5.31; 5.77) |
| Jordan | female | 2,067.02(1,650.42; 2,511.28) | 7,860.47(6,426.41; 9,423.95) | 4.95(-4.40; 15.22) | 135.18(107.96; 165.72) | 129.21(105.46; 155.90) | 0.31(-4.65; 5.52) |
| Kazakhstan | female | 14,969.91(12,231.08; 17,688.52) | 18,825.58(15,459.88; 22,629.59) | 1.08(-8.77; 11.99) | 178.74(146.27; 211.66) | 193.82(160.44; 230.75) | 0.38(-4.97; 6.04) |
| Kenya | female | 8,270.20(6,796.98; 9,951.98) | 17,343.08(14,122.12; 20,897.97) | 3.27(-6.70; 14.32) | 88.21(72.75; 107.26) | 68.80(55.62; 82.99) | -0.04(-4.35; 4.47) |
| Kiribati | female | 60.23(49.13; 73.56) | 92.25(76.03; 109.12) | 1.82(-2.86; 6.73) | 162.54(133.63; 196.17) | 144.42(119.29; 169.57) | -0.10(-5.14; 5.22) |
| Kuwait | female | 1,122.72(890.37; 1,376.09) | 3,794.49(3,012.27; 4,737.09) | 5.37(-3.31; 14.82) | 140.48(112.30; 171.13) | 131.71(108.04; 159.94) | 0.27(-4.71; 5.51) |
| Kyrgyzstan | female | 3,107.28(2,509.49; 3,727.63) | 5,216.94(4,331.07; 6,279.52) | 1.83(-6.85; 11.32) | 149.81(120.56; 180.42) | 151.12(125.87; 181.52) | 0.14(-4.96; 5.51) |
| Lao People's Democratic Republic | female | 2,694.78(2,214.40; 3,258.24) | 5,260.17(4,268.01; 6,363.83) | 2.49(-6.26; 12.06) | 139.10(114.76; 166.60) | 134.29(108.67; 161.15) | 0.14(-4.85; 5.38) |
| Latvia | female | 3,474.86(2,894.01; 4,122.12) | 1,791.70(1,484.05; 2,151.79) | -1.75(-9.12; 6.23) | 257.12(214.86; 304.51) | 206.09(171.32; 245.60) | -0.28(-5.65; 5.40) |
| Lebanon | female | 2,367.04(1,953.53; 2,859.47) | 4,525.99(3,670.91; 5,438.64) | 2.84(-5.80; 12.27) | 158.50(130.37; 192.57) | 150.29(123.06; 178.31) | 0.47(-4.64; 5.87) |
| Lesotho | female | 1,113.80(910.21; 1,350.20) | 1,098.18(916.74; 1,319.94) | 0.65(-6.43; 8.27) | 133.83(109.72; 161.07) | 114.46(95.81; 136.72) | 0.05(-4.78; 5.11) |
| Liberia | female | 838.29(672.58; 1,018.01) | 2,308.96(1,885.44; 2,802.22) | 3.76(-4.26; 12.47) | 101.53(81.55; 122.51) | 88.01(71.96; 107.02) | 0.01(-4.55; 4.79) |
| Libya | female | 2,433.98(2,000.26; 2,896.75) | 6,364.96(5,260.34; 7,735.27) | 4.08(-5.00; 14.03) | 155.31(126.38; 187.61) | 159.91(132.82; 192.33) | 0.83(-4.37; 6.31) |
| Lithuania | female | 3,857.83(3,118.05; 4,652.78) | 2,530.88(2,088.50; 3,060.34) | -1.15(-8.90; 7.27) | 199.53(161.63; 241.26) | 191.85(157.57; 230.29) | 0.17(-5.16; 5.80) |
| Luxembourg | female | 515.35(407.97; 627.57) | 897.45(736.83; 1,069.76) | 1.97(-4.99; 9.45) | 263.06(209.88; 317.48) | 279.31(233.66; 331.56) | 0.31(-5.41; 6.37) |
| Madagascar | female | 5,072.48(4,051.22; 6,267.09) | 11,601.87(9,313.30; 14,371.57) | 3.12(-6.45; 13.66) | 98.34(78.43; 121.15) | 83.15(67.33; 100.52) | -0.09(-4.59; 4.62) |
| Malawi | female | 4,202.94(3,385.88; 5,178.50) | 7,855.63(6,278.85; 9,799.39) | 2.52(-6.62; 12.54) | 99.91(80.32; 122.70) | 81.72(66.09; 98.85) | -0.18(-4.66; 4.50) |
| Malaysia | female | 13,188.89(10,847.67; 15,729.62) | 22,183.02(17,900.63; 26,840.50) | 2.13(-7.98; 13.35) | 150.69(124.05; 180.74) | 131.87(106.76; 159.35) | 0.03(-4.93; 5.25) |
| Maldives | female | 145.12(119.22; 175.51) | 313.99(251.31; 380.14) | 2.96(-3.02; 9.31) | 156.90(129.10; 186.65) | 139.04(111.86; 167.39) | -0.14(-5.14; 5.13) |
| Mali | female | 3,363.06(2,720.39; 4,130.56) | 8,205.51(6,691.49; 9,961.64) | 3.40(-5.85; 13.55) | 93.73(75.06; 115.89) | 82.71(67.19; 100.12) | -0.01(-4.50; 4.70) |
| Malta | female | 415.29(326.46; 520.50) | 415.69(334.26; 509.37) | 0.36(-5.76; 6.87) | 214.58(170.77; 267.36) | 210.22(170.87; 255.85) | 0.31(-5.13; 6.06) |
| Marshall Islands | female | 38.11(30.91; 47.17) | 52.06(42.96; 62.72) | 1.14(-2.94; 5.40) | 195.15(159.75; 233.16) | 177.46(147.61; 211.89) | 0.01(-5.25; 5.56) |
| Mauritania | female | 884.17(714.97; 1,076.29) | 1,733.09(1,418.42; 2,115.35) | 2.66(-5.00; 10.94) | 101.26(82.03; 123.58) | 86.74(70.66; 106.52) | 0.02(-4.53; 4.78) |
| Mauritius | female | 926.01(759.69; 1,117.34) | 995.14(816.50; 1,170.18) | 0.48(-6.50; 7.97) | 155.73(128.14; 186.78) | 151.62(124.11; 179.21) | 0.20(-4.90; 5.59) |
| Mexico | female | 59,161.58(49,195.02; 69,955.13) | 86,696.61(72,068.06; 102,747.15) | 1.71(-9.65; 14.51) | 141.51(117.45; 169.28) | 122.72(102.13; 145.70) | 0.09(-4.81; 5.24) |
| Micronesia (Federated States of) | female | 90.60(73.50; 112.16) | 85.44(70.27; 103.22) | -0.05(-4.58; 4.70) | 192.86(159.56; 231.30) | 161.49(133.36; 193.77) | -0.23(-5.39; 5.20) |
| Monaco | female | 36.90(29.58; 45.38) | 42.51(34.42; 51.22) | 0.77(-3.09; 4.79) | 256.93(205.95; 313.83) | 258.78(209.85; 317.20) | 0.28(-5.36; 6.26) |
| Mongolia | female | 1,395.49(1,129.00; 1,706.49) | 2,553.54(2,107.42; 3,010.03) | 2.40(-5.63; 11.11) | 142.26(115.33; 173.08) | 151.07(125.34; 177.45) | 0.35(-4.75; 5.73) |
| Montenegro | female | 589.00(476.53; 714.59) | 475.49(378.49; 584.21) | -0.19(-6.41; 6.44) | 184.40(149.14; 223.77) | 152.11(121.72; 186.16) | -0.07(-5.18; 5.31) |
| Morocco | female | 17,848.55(14,535.63; 21,633.38) | 26,799.19(21,947.10; 31,940.42) | 1.72(-8.53; 13.12) | 148.26(121.47; 179.16) | 137.03(112.43; 162.87) | 0.15(-4.86; 5.42) |
| Mozambique | female | 5,696.64(4,534.63; 7,064.65) | 11,968.14(9,640.57; 14,886.66) | 2.77(-6.80; 13.31) | 95.06(75.92; 117.13) | 82.05(66.97; 99.27) | 0.00(-4.49; 4.70) |
| Myanmar | female | 27,990.51(23,157.39; 33,905.13) | 40,018.44(32,287.38; 48,113.36) | 1.35(-9.24; 13.17) | 138.99(114.93; 165.75) | 131.10(105.20; 157.94) | 0.01(-4.94; 5.22) |
| Namibia | female | 1,033.35(845.77; 1,254.67) | 1,561.49(1,282.88; 1,863.68) | 1.92(-5.60; 10.03) | 155.41(127.75; 187.00) | 122.09(101.00; 145.41) | -0.13(-5.01; 5.00) |
| Nauru | female | 8.84(7.22; 10.81) | 8.98(7.31; 10.98) | 0.19(-2.08; 2.50) | 185.36(153.50; 224.39) | 158.87(130.94; 191.40) | -0.17(-5.31; 5.25) |
| Nepal | female | 11,646.94(9,394.16; 14,130.92) | 19,605.21(15,930.69; 23,424.65) | 2.31(-7.69; 13.40) | 131.31(105.64; 159.26) | 111.23(90.44; 133.40) | -0.01(-4.80; 5.02) |
| Netherlands | female | 20,087.28(16,383.77; 24,188.17) | 17,661.30(14,492.19; 21,099.40) | -0.12(-9.80; 10.60) | 266.98(220.69; 316.40) | 227.93(189.40; 268.82) | -0.19(-5.69; 5.63) |
| New Zealand | female | 5,941.88(4,825.41; 7,197.46) | 8,500.26(7,080.13; 10,186.42) | 1.05(-8.03; 11.02) | 332.72(270.54; 403.96) | 349.93(289.68; 422.23) | 0.27(-5.65; 6.57) |
| Nicaragua | female | 2,475.45(2,040.23; 2,937.95) | 4,143.06(3,353.58; 5,056.27) | 2.01(-6.47; 11.25) | 144.62(118.43; 174.33) | 115.70(94.36; 140.50) | -0.33(-5.14; 4.73) |
| Niger | female | 3,007.03(2,433.25; 3,685.09) | 7,994.79(6,533.87; 9,705.50) | 3.60(-5.63; 13.75) | 95.13(76.30; 117.36) | 82.16(66.95; 99.93) | 0.04(-4.45; 4.74) |
| Nigeria | female | 30,415.12(25,142.00; 36,833.33) | 80,889.84(66,658.68; 96,416.55) | 3.63(-7.88; 16.57) | 83.80(68.30; 102.70) | 77.31(63.30; 92.67) | 0.20(-4.25; 4.85) |
| Niue | female | 1.92(1.58; 2.34) | 1.32(1.08; 1.60) | -1.18(-1.58; -0.78) | 186.25(152.17; 224.63) | 164.51(133.94; 199.46) | -0.04(-5.22; 5.42) |
| North Macedonia | female | 1,522.82(1,200.03; 1,888.95) | 1,529.45(1,211.56; 1,886.29) | 0.45(-6.95; 8.43) | 146.26(115.49; 181.38) | 134.66(107.85; 163.30) | 0.17(-4.82; 5.43) |
| Northern Mariana Islands | female | 51.52(41.71; 62.18) | 39.74(32.13; 48.10) | -1.30(-5.13; 2.68) | 198.28(163.62; 238.19) | 168.84(136.27; 204.45) | -0.13(-5.32; 5.36) |
| Norway | female | 5,763.76(4,594.98; 7,072.30) | 7,964.93(6,773.90; 9,476.87) | 1.10(-7.92; 11.01) | 267.88(214.43; 327.94) | 300.99(253.66; 356.93) | 0.38(-5.41; 6.52) |
| Oman | female | 825.76(656.59; 1,015.96) | 2,520.35(2,016.22; 3,085.02) | 4.16(-3.99; 13.00) | 130.25(104.13; 161.10) | 128.80(105.82; 155.91) | 0.33(-4.63; 5.55) |
| Pakistan | female | 72,159.13(58,957.41; 87,181.96) | 129,559.39(106,703.48; 153,336.80) | 2.54(-9.29; 15.90) | 153.71(127.16; 182.05) | 111.78(92.72; 132.07) | -0.39(-5.17; 4.63) |
| Palau | female | 15.21(12.42; 18.43) | 13.19(10.79; 15.96) | -0.52(-3.18; 2.22) | 188.66(155.30; 226.98) | 164.37(134.62; 198.26) | -0.12(-5.29; 5.34) |
| Palestine | female | 1,127.28(916.28; 1,364.68) | 3,308.76(2,706.53; 3,975.78) | 3.96(-4.45; 13.11) | 136.81(109.61; 168.37) | 130.08(105.81; 155.93) | 0.15(-4.80; 5.37) |
| Panama | female | 1,825.29(1,484.41; 2,193.62) | 2,948.44(2,410.80; 3,572.88) | 1.96(-6.18; 10.80) | 152.65(123.95; 183.87) | 136.12(110.89; 165.05) | 0.05(-4.94; 5.31) |
| Papua New Guinea | female | 3,152.87(2,566.95; 3,934.34) | 7,737.80(6,298.17; 9,435.07) | 3.33(-5.86; 13.43) | 162.57(134.02; 198.81) | 149.88(123.15; 182.15) | 0.03(-5.06; 5.39) |
| Paraguay | female | 3,643.12(2,914.66; 4,384.94) | 4,862.17(4,005.56; 5,772.05) | 1.92(-6.71; 11.34) | 188.84(153.92; 226.04) | 128.75(106.49; 151.88) | -0.32(-5.25; 4.87) |
| Peru | female | 19,860.40(16,360.44; 23,315.57) | 27,274.94(22,407.90; 32,077.81) | 1.64(-8.62; 13.05) | 183.88(152.95; 215.75) | 141.52(116.46; 166.49) | -0.12(-5.15; 5.18) |
| Philippines | female | 41,937.57(34,953.02; 50,217.35) | 71,570.00(59,562.47; 84,377.15) | 2.11(-9.11; 14.72) | 136.12(113.90; 161.36) | 122.13(101.31; 144.58) | 0.02(-4.87; 5.16) |
| Poland | female | 33,825.91(27,726.17; 40,662.36) | 28,164.34(22,699.64; 34,072.85) | -0.20(-10.31; 11.04) | 176.42(145.45; 211.73) | 153.30(124.89; 184.65) | 0.04(-5.07; 5.42) |
| Portugal | female | 11,771.91(9,398.28; 14,515.67) | 9,821.31(7,917.24; 11,959.84) | -0.17(-9.29; 9.88) | 227.04(181.17; 279.84) | 192.60(154.54; 233.86) | -0.08(-5.41; 5.55) |
| Puerto Rico | female | 4,366.10(3,466.96; 5,444.70) | 3,521.63(2,903.55; 4,300.11) | -0.44(-8.59; 8.42) | 227.29(180.73; 283.35) | 217.44(176.97; 267.81) | 0.05(-5.42; 5.83) |
| Qatar | female | 203.60(159.53; 256.87) | 1,430.66(1,136.66; 1,793.72) | 7.63(-0.20; 16.08) | 135.94(109.10; 167.94) | 134.32(109.92; 162.63) | 0.51(-4.49; 5.78) |
| Republic of Korea | female | 54,986.53(43,469.64; 68,754.16) | 51,868.98(41,786.76; 63,102.40) | 0.17(-10.55; 12.18) | 220.90(174.76; 276.59) | 214.44(173.73; 264.22) | 0.11(-5.33; 5.87) |
| Republic of Moldova | female | 4,533.79(3,645.68; 5,455.87) | 3,109.01(2,497.31; 3,821.68) | -0.88(-8.85; 7.78) | 194.98(157.55; 233.46) | 163.32(132.52; 196.74) | -0.25(-5.40; 5.19) |
| Romania | female | 18,027.73(14,235.39; 22,176.35) | 13,160.60(10,275.48; 16,453.28) | -0.75(-10.09; 9.57) | 152.06(120.30; 187.60) | 136.97(107.13; 167.54) | 0.04(-4.96; 5.30) |
| Russian Federation | female | 204,090.43(170,499.75; 240,674.09) | 176,256.75(148,484.58; 206,321.05) | -0.38(-12.17; 12.98) | 259.48(218.05; 305.32) | 244.45(207.60; 283.49) | -0.11(-5.68; 5.78) |
| Rwanda | female | 3,094.77(2,440.50; 3,909.55) | 6,173.76(4,899.34; 7,671.66) | 3.26(-5.71; 13.08) | 98.33(78.58; 123.62) | 88.96(71.38; 109.28) | 0.15(-4.43; 4.94) |
| Saint Kitts and Nevis | female | 33.74(26.81; 43.10) | 51.34(41.83; 61.64) | 1.71(-2.38; 5.98) | 166.64(134.68; 207.12) | 164.54(133.92; 202.46) | 0.12(-5.06; 5.59) |
| Saint Lucia | female | 151.54(117.73; 193.53) | 154.28(125.42; 185.00) | 0.61(-4.60; 6.11) | 206.36(164.72; 253.39) | 170.91(136.85; 211.18) | -0.04(-5.33; 5.55) |
| Saint Vincent and the Grenadines | female | 87.41(69.22; 111.80) | 87.09(70.63; 105.87) | 0.12(-4.43; 4.90) | 161.64(130.41; 200.51) | 155.12(125.52; 189.50) | 0.03(-5.09; 5.43) |
| Samoa | female | 143.05(114.02; 181.98) | 182.59(151.15; 219.67) | 1.15(-4.19; 6.78) | 187.17(152.08; 226.87) | 179.35(148.90; 212.66) | 0.15(-5.13; 5.71) |
| San Marino | female | 30.29(24.23; 37.19) | 38.40(31.43; 46.44) | 1.31(-2.48; 5.23) | 254.66(201.98; 313.76) | 245.12(197.05; 299.99) | 0.21(-5.37; 6.11) |
| Sao Tome and Principe | female | 50.79(40.99; 61.90) | 89.89(71.99; 109.24) | 2.28(-2.39; 7.18) | 105.81(84.81; 129.39) | 84.41(67.54; 102.48) | -0.31(-4.81; 4.41) |
| Saudi Arabia | female | 8,426.99(6,733.53; 10,256.60) | 28,485.31(22,794.29; 34,975.97) | 4.75(-5.86; 16.56) | 142.84(114.30; 174.85) | 144.86(117.57; 175.07) | 0.53(-4.55; 5.89) |
| Senegal | female | 3,238.07(2,629.30; 3,926.06) | 6,229.17(5,131.08; 7,583.71) | 2.47(-6.43; 12.22) | 102.32(82.95; 123.72) | 85.24(69.68; 103.50) | -0.17(-4.69; 4.56) |
| Serbia | female | 7,210.79(5,698.63; 8,761.74) | 5,834.16(4,667.20; 7,247.37) | -0.53(-9.13; 8.87) | 151.88(120.16; 185.51) | 131.83(105.24; 163.27) | 0.02(-4.95; 5.24) |
| Seychelles | female | 70.85(57.64; 87.61) | 75.93(61.87; 90.60) | 0.72(-3.73; 5.36) | 189.68(155.09; 231.67) | 152.06(124.12; 181.83) | -0.18(-5.27; 5.18) |
| Sierra Leone | female | 1,691.81(1,382.64; 2,041.33) | 3,518.43(2,871.80; 4,300.21) | 3.21(-5.19; 12.36) | 103.67(84.12; 125.84) | 84.55(68.92; 103.48) | -0.17(-4.68; 4.55) |
| Singapore | female | 4,067.41(3,183.91; 5,095.82) | 6,242.07(4,932.08; 7,637.03) | 1.78(-7.08; 11.49) | 225.83(177.25; 284.56) | 216.77(172.07; 267.04) | 0.06(-5.39; 5.83) |
| Slovakia | female | 5,215.74(4,217.30; 6,317.55) | 4,287.27(3,512.97; 5,149.69) | -0.36(-8.68; 8.71) | 193.06(156.72; 232.40) | 162.25(134.06; 190.94) | -0.18(-5.33; 5.25) |
| Slovenia | female | 1,897.78(1,512.99; 2,294.59) | 1,652.87(1,339.39; 1,983.91) | -0.15(-7.57; 7.87) | 192.80(153.46; 234.19) | 185.98(153.50; 220.82) | 0.30(-5.02; 5.92) |
| Solomon Islands | female | 265.27(213.75; 334.26) | 497.34(408.62; 588.54) | 2.31(-4.09; 9.14) | 171.90(141.08; 209.51) | 144.76(119.02; 168.88) | -0.20(-5.24; 5.11) |
| Somalia | female | 2,914.33(2,333.88; 3,617.27) | 7,303.74(5,901.31; 9,182.62) | 3.47(-5.67; 13.50) | 96.37(77.56; 118.47) | 78.38(63.83; 96.13) | -0.17(-4.61; 4.47) |
| South Africa | female | 50,944.45(42,702.67; 61,019.80) | 44,871.94(37,835.75; 52,709.29) | -0.18(-10.71; 11.59) | 250.36(210.87; 296.07) | 145.50(123.44; 170.45) | -1.42(-6.40; 3.81) |
| South Sudan | female | 2,486.84(1,988.15; 3,081.93) | 3,788.10(3,013.39; 4,723.69) | 1.87(-6.50; 10.99) | 103.06(82.37; 126.23) | 83.73(67.07; 102.22) | -0.16(-4.67; 4.55) |
| Spain | female | 57,105.80(48,484.77; 66,099.05) | 58,331.17(49,817.65; 68,393.39) | 0.17(-10.66; 12.31) | 293.79(249.56; 340.47) | 292.41(254.91; 335.76) | -0.05(-5.80; 6.04) |
| Sri Lanka | female | 14,407.36(11,863.79; 17,321.39) | 16,394.01(13,370.63; 19,709.66) | 0.71(-8.98; 11.42) | 159.55(131.32; 191.54) | 140.39(113.92; 168.91) | -0.03(-5.05; 5.26) |
| Sudan | female | 9,973.11(8,052.70; 12,097.47) | 27,299.75(22,346.55; 32,652.74) | 3.38(-7.05; 14.98) | 117.23(95.16; 143.22) | 128.25(105.18; 153.42) | 0.49(-4.47; 5.70) |
| Suriname | female | 294.03(236.23; 366.66) | 450.65(371.33; 528.77) | 1.85(-4.44; 8.55) | 153.28(122.56; 188.36) | 151.26(124.74; 178.63) | 0.20(-4.91; 5.58) |
| Sweden | female | 9,393.90(7,400.47; 11,657.25) | 11,463.44(9,365.40; 13,849.84) | 0.98(-8.39; 11.31) | 221.48(176.59; 272.24) | 239.91(196.95; 290.07) | 0.60(-4.98; 6.52) |
| Switzerland | female | 12,355.55(10,091.24; 14,997.99) | 12,513.71(10,203.79; 14,937.00) | 0.31(-9.08; 10.68) | 371.26(303.88; 447.87) | 313.10(258.82; 369.56) | -0.33(-6.13; 5.82) |
| Syrian Arab Republic | female | 6,348.53(5,065.47; 7,708.20) | 10,478.30(8,609.66; 12,521.70) | 1.96(-7.42; 12.29) | 126.59(100.98; 154.96) | 133.11(109.47; 160.13) | 0.57(-4.43; 5.82) |
| Taiwan (Province of China) | female | 23,466.44(19,011.15; 28,339.98) | 23,464.91(19,017.39; 28,935.24) | 0.46(-9.55; 11.58) | 214.88(175.71; 257.86) | 188.54(151.95; 228.37) | 0.06(-5.26; 5.68) |
| Tajikistan | female | 3,159.13(2,548.48; 3,836.18) | 7,011.60(5,797.56; 8,363.28) | 2.81(-6.24; 12.74) | 135.61(109.34; 165.35) | 138.74(114.85; 165.33) | 0.15(-4.86; 5.43) |
| Thailand | female | 46,656.58(37,933.86; 56,576.96) | 47,314.36(38,569.07; 56,144.04) | 0.53(-10.15; 12.47) | 147.66(119.77; 179.06) | 136.01(110.85; 161.60) | 0.11(-4.89; 5.37) |
| Timor-Leste | female | 516.64(426.23; 620.49) | 892.81(726.56; 1,091.84) | 2.11(-4.86; 9.59) | 141.94(117.50; 170.38) | 130.12(105.66; 157.89) | 0.10(-4.85; 5.31) |
| Togo | female | 1,370.48(1,091.43; 1,704.67) | 3,198.52(2,594.23; 3,893.23) | 3.30(-5.02; 12.35) | 89.33(70.90; 111.61) | 78.71(64.12; 95.29) | 0.11(-4.35; 4.77) |
| Tokelau | female | 1.28(1.05; 1.57) | 1.04(0.85; 1.26) | -0.65(-0.97; -0.33) | 176.51(145.55; 214.42) | 156.71(127.18; 189.97) | 0.01(-5.12; 5.42) |
| Tonga | female | 87.65(70.29; 111.28) | 90.84(73.74; 109.57) | 0.36(-4.25; 5.19) | 187.56(152.99; 227.53) | 173.33(141.95; 206.96) | 0.03(-5.20; 5.56) |
| Trinidad and Tobago | female | 996.13(793.98; 1,251.73) | 1,036.92(855.24; 1,243.04) | 0.32(-6.68; 7.86) | 163.14(132.27; 201.34) | 149.09(123.20; 176.94) | 0.01(-5.07; 5.37) |
| Tunisia | female | 5,019.56(3,986.94; 6,078.75) | 8,599.81(6,929.43; 10,527.68) | 2.26(-6.96; 12.38) | 130.13(103.70; 160.06) | 135.02(108.97; 163.51) | 0.60(-4.41; 5.88) |
| Turkey | female | 38,997.15(31,271.40; 47,901.00) | 57,393.25(46,182.75; 69,197.78) | 1.77(-9.21; 14.08) | 138.22(109.88; 171.79) | 128.94(104.23; 156.69) | 0.25(-4.70; 5.46) |
| Turkmenistan | female | 2,420.45(1,972.82; 2,939.98) | 4,124.84(3,470.17; 4,889.88) | 1.72(-6.73; 10.93) | 140.96(114.07; 173.27) | 161.97(136.10; 192.32) | 0.47(-4.71; 5.94) |
| Tuvalu | female | 8.23(6.76; 10.01) | 9.09(7.45; 11.08) | 0.68(-1.60; 3.02) | 170.50(140.22; 207.44) | 153.53(126.22; 186.39) | -0.04(-5.15; 5.34) |
| Uganda | female | 8,260.52(6,645.83; 10,229.87) | 16,975.35(13,869.41; 20,476.03) | 3.02(-6.91; 14.00) | 108.79(88.61; 132.01) | 84.66(68.85; 101.09) | -0.19(-4.70; 4.53) |
| Ukraine | female | 51,692.62(42,473.94; 61,822.55) | 37,793.68(31,120.07; 45,649.35) | -0.67(-11.00; 10.86) | 187.96(154.70; 224.91) | 170.07(142.04; 201.27) | -0.02(-5.23; 5.47) |
| United Arab Emirates | female | 1,074.21(859.93; 1,314.86) | 5,486.96(4,347.00; 6,913.96) | 6.66(-2.50; 16.69) | 170.40(139.40; 206.23) | 159.38(129.96; 190.32) | 0.29(-4.88; 5.73) |
| United Kingdom | female | 90,768.00(75,075.32; 109,439.86) | 105,234.43(89,545.45; 123,542.18) | 0.78(-10.66; 13.68) | 330.63(272.85; 400.76) | 336.23(284.82; 396.38) | 0.34(-5.56; 6.61) |
| United Republic of Tanzania | female | 13,759.96(11,102.16; 17,213.31) | 26,026.87(20,811.28; 32,695.24) | 2.56(-7.73; 13.99) | 113.71(93.32; 137.39) | 89.73(72.20; 109.85) | -0.24(-4.80; 4.53) |
| United States of America | female | 502,283.85(423,032.31; 590,566.66) | 747,345.68(653,622.39; 845,629.95) | 1.84(-11.54; 17.24) | 395.04(334.08; 467.35) | 496.42(433.01; 563.83) | 1.28(-5.05; 8.04) |
| United States Virgin Islands | female | 109.69(87.89; 136.62) | 74.35(60.77; 89.31) | -0.65(-5.04; 3.94) | 189.77(152.41; 234.89) | 191.23(155.62; 231.97) | 0.20(-5.14; 5.85) |
| Uruguay | female | 3,896.38(3,216.29; 4,601.69) | 3,427.94(2,862.38; 4,053.48) | 0.17(-7.97; 9.04) | 253.05(208.55; 298.90) | 202.62(170.53; 239.75) | -0.12(-5.50; 5.56) |
| Uzbekistan | female | 12,726.16(10,223.71; 15,629.36) | 25,265.19(20,571.38; 30,277.00) | 2.52(-7.75; 13.93) | 134.11(107.53; 164.84) | 141.66(115.79; 168.52) | 0.34(-4.70; 5.65) |
| Vanuatu | female | 110.89(90.67; 137.46) | 224.16(185.92; 263.98) | 2.55(-3.06; 8.48) | 159.77(131.54; 193.29) | 143.90(119.50; 168.10) | -0.09(-5.13; 5.22) |
| Venezuela (Bolivarian Republic of) | female | 13,051.70(10,532.79; 15,750.07) | 17,350.81(14,226.70; 20,861.05) | 1.54(-8.30; 12.43) | 142.18(114.40; 172.90) | 121.79(99.98; 146.48) | -0.16(-5.03; 4.97) |
| Viet Nam | female | 51,972.68(42,976.83; 62,243.80) | 71,548.40(57,455.37; 86,867.53) | 1.48(-9.68; 14.02) | 151.97(125.36; 180.75) | 137.38(111.29; 165.85) | 0.01(-4.99; 5.28) |
| Yemen | female | 5,636.57(4,492.88; 6,968.27) | 18,749.66(15,313.21; 22,414.97) | 4.24(-5.91; 15.48) | 112.93(89.30; 141.10) | 119.25(97.57; 141.41) | 0.39(-4.50; 5.53) |
| Zambia | female | 4,124.97(3,415.07; 4,937.33) | 7,282.14(5,916.31; 8,956.09) | 2.69(-6.39; 12.64) | 115.08(95.41; 138.36) | 79.66(64.56; 97.16) | -0.50(-4.93; 4.15) |
| Zimbabwe | female | 7,746.53(6,206.92; 9,632.93) | 8,186.94(6,740.08; 9,974.59) | 0.52(-8.48; 10.41) | 161.88(131.33; 197.31) | 106.29(87.84; 129.06) | -0.79(-5.50; 4.15) |
| Afghanistan | male | 6,411.10(5,279.17; 7,934.36) | 19,939.68(16,592.62; 23,683.65) | 4.40(-5.82; 15.74) | 143.11(118.60; 170.57) | 136.71(114.06; 161.24) | 0.35(-4.66; 5.63) |
| Albania | male | 3,340.34(2,702.43; 4,137.55) | 2,719.04(2,318.99; 3,165.09) | -0.67(-8.53; 7.87) | 187.28(152.18; 229.00) | 203.48(172.64; 237.32) | 0.35(-5.06; 6.07) |
| Algeria | male | 18,280.25(14,908.43; 22,467.04) | 35,416.02(29,362.31; 41,547.97) | 2.42(-8.18; 14.23) | 159.42(130.06; 192.21) | 155.17(129.48; 181.16) | 0.26(-4.88; 5.68) |
| American Samoa | male | 59.34(47.18; 77.65) | 55.35(43.61; 71.10) | -0.08(-4.18; 4.20) | 235.34(191.52; 300.76) | 209.39(167.53; 260.71) | -0.18(-5.59; 5.54) |
| Andorra | male | 118.29(95.07; 145.24) | 130.73(107.85; 165.53) | 0.47(-4.50; 5.71) | 351.53(284.74; 436.94) | 312.99(256.41; 387.60) | 0.11(-5.71; 6.28) |
| Angola | male | 7,424.69(6,214.41; 8,998.65) | 18,747.97(15,399.03; 22,859.54) | 3.46(-6.60; 14.61) | 158.96(132.49; 189.31) | 138.57(115.08; 165.06) | -0.02(-5.03; 5.25) |
| Antigua and Barbuda | male | 76.99(61.47; 97.79) | 106.82(86.84; 131.29) | 1.45(-3.40; 6.54) | 253.15(203.81; 317.18) | 234.66(186.68; 296.97) | 0.02(-5.53; 5.90) |
| Argentina | male | 33,868.30(28,131.35; 39,614.57) | 43,356.17(37,050.45; 50,388.68) | 1.25(-9.42; 13.16) | 210.27(174.32; 247.30) | 188.43(161.33; 218.38) | 0.08(-5.24; 5.70) |
| Armenia | male | 3,233.39(2,673.05; 3,840.31) | 2,326.70(1,960.31; 2,731.37) | -0.46(-8.19; 7.92) | 186.95(154.63; 222.50) | 163.12(136.95; 191.93) | 0.11(-5.06; 5.57) |
| Australia | male | 55,625.84(47,139.00; 64,823.47) | 53,816.66(46,634.49; 62,782.85) | 0.49(-10.30; 12.58) | 634.08(539.86; 737.73) | 455.71(394.97; 528.03) | -0.46(-6.63; 6.12) |
| Austria | male | 11,649.68(9,698.35; 13,743.44) | 12,802.50(10,771.60; 14,817.83) | 0.66(-8.81; 11.10) | 293.28(242.41; 345.07) | 314.33(266.45; 361.47) | 0.54(-5.32; 6.77) |
| Azerbaijan | male | 6,600.70(5,414.26; 7,877.22) | 8,946.63(7,343.29; 10,740.74) | 1.66(-7.53; 11.77) | 178.62(147.85; 214.17) | 159.54(131.11; 191.11) | 0.09(-5.06; 5.52) |
| Bahamas | male | 417.29(338.43; 511.87) | 556.62(448.61; 687.03) | 1.21(-5.25; 8.10) | 298.59(242.79; 360.22) | 271.57(218.09; 336.76) | -0.03(-5.70; 5.99) |
| Bahrain | male | 594.81(479.70; 719.07) | 1,711.61(1,384.69; 2,093.68) | 4.78(-3.06; 13.25) | 169.68(140.25; 201.73) | 135.72(110.69; 163.21) | -0.42(-5.39; 4.82) |
| Bangladesh | male | 71,026.58(57,396.37; 84,019.06) | 113,644.98(94,423.21; 135,685.82) | 1.96(-9.67; 15.10) | 153.38(124.79; 182.64) | 137.73(114.59; 164.40) | 0.11(-4.89; 5.38) |
| Barbados | male | 364.29(290.76; 464.71) | 330.37(272.46; 397.20) | 0.06(-5.81; 6.30) | 279.65(224.36; 354.69) | 249.58(202.83; 305.18) | -0.12(-5.70; 5.79) |
| Belarus | male | 16,454.99(13,890.42; 19,428.39) | 10,213.90(8,704.38; 12,114.21) | -0.94(-10.04; 9.07) | 321.81(271.22; 380.65) | 241.59(204.58; 288.07) | -0.34(-5.88; 5.53) |
| Belgium | male | 17,180.71(13,964.70; 20,748.21) | 15,503.93(13,049.67; 18,144.81) | 0.10(-9.48; 10.69) | 352.22(288.20; 423.27) | 309.68(262.35; 362.84) | -0.05(-5.85; 6.11) |
| Belize | male | 263.83(208.74; 331.12) | 555.47(439.32; 700.52) | 2.82(-3.72; 9.80) | 263.09(215.40; 318.58) | 230.40(185.48; 287.08) | -0.15(-5.64; 5.67) |
| Benin | male | 2,224.74(1,849.46; 2,691.73) | 6,683.79(5,540.39; 7,977.23) | 4.17(-4.95; 14.16) | 126.32(105.62; 150.70) | 117.07(98.14; 138.41) | 0.31(-4.55; 5.41) |
| Bermuda | male | 89.79(73.90; 108.75) | 71.15(58.09; 88.23) | -0.41(-4.75; 4.12) | 285.95(232.67; 355.31) | 255.01(202.99; 323.71) | -0.07(-5.68; 5.86) |
| Bhutan | male | 386.56(306.88; 488.64) | 575.46(475.44; 685.84) | 2.05(-4.49; 9.03) | 125.98(101.57; 151.71) | 128.71(106.38; 152.87) | 0.32(-4.62; 5.53) |
| Bolivia (Plurinational State of) | male | 6,289.27(5,361.08; 7,348.22) | 9,747.84(8,156.48; 11,304.62) | 2.10(-7.21; 12.34) | 207.00(178.00; 238.34) | 157.04(131.47; 181.43) | -0.30(-5.41; 5.09) |
| Bosnia and Herzegovina | male | 4,028.51(3,218.83; 4,915.58) | 2,283.74(1,873.80; 2,755.55) | -0.96(-8.64; 7.37) | 159.86(127.76; 195.62) | 147.40(120.36; 179.67) | 0.31(-4.78; 5.66) |
| Botswana | male | 1,170.92(965.24; 1,427.29) | 1,833.65(1,522.76; 2,138.37) | 1.99(-5.68; 10.29) | 198.17(166.02; 232.69) | 141.29(117.89; 164.86) | -0.47(-5.48; 4.80) |
| Brazil | male | 180,142.59(150,412.95; 222,557.80) | 229,032.84(195,374.57; 263,779.02) | 1.38(-10.87; 15.31) | 227.30(192.71; 275.34) | 205.95(176.22; 237.68) | 0.29(-5.14; 6.03) |
| Brunei Darussalam | male | 386.17(311.36; 469.81) | 587.90(476.07; 707.35) | 1.73(-4.82; 8.73) | 255.63(208.60; 307.77) | 212.70(173.15; 258.41) | -0.29(-5.72; 5.44) |
| Bulgaria | male | 9,143.07(7,635.89; 10,606.83) | 6,684.90(5,731.86; 7,682.04) | -0.84(-9.53; 8.68) | 218.83(182.74; 254.14) | 237.13(205.30; 271.82) | 0.42(-5.13; 6.29) |
| Burkina Faso | male | 3,944.30(3,261.62; 4,797.71) | 9,785.56(8,115.79; 11,625.18) | 3.82(-5.65; 14.23) | 116.85(97.82; 137.76) | 110.42(91.49; 130.00) | 0.35(-4.45; 5.39) |
| Burundi | male | 3,147.79(2,502.41; 4,123.48) | 7,226.93(5,596.93; 9,469.03) | 3.15(-5.95; 13.14) | 131.67(107.61; 163.18) | 113.42(91.58; 140.43) | -0.20(-5.00; 4.85) |
| Cabo Verde | male | 211.15(178.44; 250.21) | 460.18(367.55; 561.07) | 3.62(-2.79; 10.45) | 162.51(136.35; 190.80) | 145.76(117.88; 177.38) | 0.51(-4.58; 5.87) |
| Cambodia | male | 6,963.24(5,663.31; 8,776.83) | 12,963.35(10,732.70; 15,346.05) | 2.29(-7.32; 12.90) | 156.34(130.21; 189.83) | 144.23(119.13; 170.28) | -0.08(-5.12; 5.24) |
| Cameroon | male | 6,162.95(5,178.74; 7,285.27) | 16,708.85(13,832.08; 20,054.09) | 4.32(-5.71; 15.42) | 148.02(125.31; 173.35) | 120.91(100.87; 144.21) | 0.24(-4.65; 5.37) |
| Canada | male | 60,364.79(52,796.74; 68,528.46) | 69,681.25(61,633.05; 78,355.99) | 1.13(-9.96; 13.60) | 452.05(400.19; 506.91) | 448.41(397.52; 500.87) | 0.75(-5.47; 7.38) |
| Central African Republic | male | 1,827.86(1,518.20; 2,201.11) | 3,079.74(2,515.15; 3,842.20) | 1.97(-6.21; 10.87) | 151.19(127.79; 177.56) | 121.33(100.40; 146.37) | -0.36(-5.22; 4.75) |
| Chad | male | 2,735.77(2,285.03; 3,277.42) | 7,034.75(5,704.93; 8,496.06) | 3.69(-5.43; 13.69) | 121.52(102.47; 142.42) | 106.28(87.65; 125.68) | 0.14(-4.61; 5.13) |
| Chile | male | 17,880.30(15,314.37; 20,664.54) | 23,131.67(19,850.38; 26,726.05) | 1.10(-8.95; 12.27) | 254.08(217.00; 293.42) | 255.11(220.20; 294.90) | 0.31(-5.32; 6.27) |
| China | male | 1627,017.37(1383,146.24; 1884,682.01) | 1289,856.21(1087,512.56; 1513,406.85) | -0.32(-13.92; 15.43) | 238.92(203.28; 277.14) | 180.21(151.70; 208.57) | -0.65(-5.89; 4.88) |
| Colombia | male | 32,998.20(27,945.76; 38,731.83) | 53,413.10(45,661.07; 62,435.96) | 1.60(-9.30; 13.80) | 199.24(168.60; 234.92) | 212.72(181.04; 248.80) | 0.35(-5.10; 6.11) |
| Comoros | male | 275.11(217.06; 368.61) | 485.45(390.32; 600.97) | 2.11(-4.26; 8.90) | 132.04(107.41; 163.99) | 125.25(101.56; 152.78) | 0.09(-4.82; 5.26) |
| Congo | male | 1,956.78(1,622.38; 2,347.14) | 3,839.23(3,113.10; 4,673.58) | 2.56(-5.87; 11.75) | 183.46(153.12; 218.04) | 142.23(115.83; 169.76) | -0.43(-5.44; 4.84) |
| Cook Islands | male | 23.83(19.05; 31.20) | 17.58(14.12; 21.94) | -0.82(-3.75; 2.20) | 234.39(190.47; 296.94) | 218.52(174.25; 273.14) | 0.00(-5.46; 5.78) |
| Costa Rica | male | 2,960.02(2,401.07; 3,717.22) | 4,163.61(3,496.77; 4,853.06) | 1.46(-6.99; 10.67) | 194.37(159.72; 239.59) | 173.18(145.26; 202.04) | 0.09(-5.15; 5.61) |
| Croatia | male | 6,402.26(5,296.38; 7,668.49) | 4,911.39(4,201.66; 5,779.53) | -0.62(-9.05; 8.59) | 262.08(216.33; 313.08) | 271.23(234.14; 312.05) | 0.32(-5.37; 6.36) |
| Cuba | male | 14,208.79(11,580.45; 18,084.93) | 10,362.56(8,302.78; 12,942.83) | -0.57(-9.71; 9.49) | 234.61(189.55; 299.65) | 203.64(158.56; 263.79) | -0.20(-5.57; 5.49) |
| Cyprus | male | 1,090.52(875.09; 1,329.51) | 1,638.40(1,333.49; 1,978.81) | 1.89(-5.68; 10.06) | 265.56(213.80; 322.60) | 237.94(197.25; 283.17) | 0.06(-5.49; 5.94) |
| Czechia | male | 15,150.43(12,635.83; 17,869.91) | 12,422.93(10,543.29; 14,305.27) | -0.33(-9.66; 9.96) | 294.15(246.04; 347.19) | 286.35(243.26; 330.32) | 0.36(-5.39; 6.46) |
| Côte d'Ivoire | male | 7,045.00(5,907.54; 8,350.79) | 14,879.43(12,216.92; 17,683.45) | 2.99(-6.81; 13.82) | 131.42(110.54; 155.22) | 113.61(94.08; 134.45) | 0.17(-4.65; 5.23) |
| Democratic People's Republic of Korea | male | 20,892.21(17,431.21; 24,739.62) | 25,538.94(21,612.78; 29,818.03) | 1.06(-9.08; 12.33) | 206.34(172.28; 243.68) | 175.47(146.92; 204.06) | -0.31(-5.53; 5.21) |
| Democratic Republic of the Congo | male | 24,858.11(20,595.43; 30,192.73) | 53,956.06(43,905.72; 65,906.91) | 2.98(-8.06; 15.34) | 152.49(127.83; 179.64) | 125.51(103.39; 148.94) | -0.26(-5.15; 4.89) |
| Denmark | male | 11,115.29(9,260.72; 13,810.17) | 9,214.22(7,643.08; 10,921.91) | -0.15(-9.21; 9.82) | 416.18(346.85; 513.32) | 344.03(285.87; 407.73) | -0.14(-6.04; 6.13) |
| Djibouti | male | 345.64(275.11; 457.28) | 920.73(744.03; 1,141.50) | 3.80(-3.32; 11.43) | 134.55(109.66; 167.24) | 127.89(104.21; 157.91) | 0.17(-4.77; 5.36) |
| Dominica | male | 118.98(92.30; 163.82) | 89.64(70.85; 111.35) | -0.74(-5.28; 4.03) | 292.66(232.21; 385.99) | 254.15(198.81; 316.95) | -0.37(-5.96; 5.55) |
| Dominican Republic | male | 6,233.62(5,052.35; 7,685.73) | 10,578.75(8,508.30; 13,311.49) | 2.03(-7.36; 12.37) | 176.75(144.72; 211.96) | 181.07(144.73; 230.07) | 0.31(-4.98; 5.90) |
| Ecuador | male | 8,999.57(7,475.94; 10,724.19) | 14,197.95(11,837.77; 16,722.11) | 2.08(-7.60; 12.77) | 179.72(151.58; 211.25) | 151.69(126.74; 178.11) | 0.03(-5.08; 5.41) |
| Egypt | male | 32,940.45(27,047.34; 39,964.99) | 68,518.17(57,025.63; 83,042.85) | 2.62(-8.61; 15.24) | 124.87(102.36; 152.64) | 125.06(104.57; 151.02) | 0.25(-4.67; 5.42) |
| El Salvador | male | 3,531.54(2,967.66; 4,178.07) | 4,166.11(3,478.12; 5,027.91) | 0.80(-7.54; 9.89) | 150.64(126.20; 177.89) | 136.46(113.43; 164.84) | -0.03(-4.98; 5.18) |
| Equatorial Guinea | male | 276.46(231.72; 334.80) | 1,227.41(985.54; 1,493.36) | 5.82(-1.72; 13.93) | 168.67(141.82; 198.13) | 149.00(122.36; 177.47) | 0.19(-4.91; 5.55) |
| Eritrea | male | 1,933.11(1,546.26; 2,567.52) | 4,591.79(3,627.02; 5,735.42) | 3.27(-5.41; 12.74) | 139.50(115.61; 170.94) | 134.25(109.00; 163.63) | 0.09(-4.89; 5.34) |
| Estonia | male | 2,956.20(2,515.00; 3,434.05) | 2,718.82(2,395.70; 3,065.87) | -0.03(-7.93; 8.55) | 393.57(336.29; 454.91) | 510.37(458.09; 571.24) | 0.91(-5.43; 7.67) |
| Eswatini | male | 727.63(616.92; 867.62) | 1,000.72(828.22; 1,175.02) | 1.33(-5.70; 8.88) | 210.83(179.72; 245.88) | 168.88(142.09; 194.63) | -0.21(-5.40; 5.26) |
| Ethiopia | male | 27,770.62(22,471.92; 34,896.98) | 63,472.14(51,630.62; 79,065.46) | 2.88(-8.31; 15.43) | 124.19(104.11; 149.37) | 115.97(95.86; 138.79) | 0.03(-4.81; 5.11) |
| Fiji | male | 840.80(655.34; 1,105.16) | 910.30(722.02; 1,165.53) | 0.39(-6.49; 7.78) | 203.26(162.20; 261.04) | 188.12(149.48; 240.11) | -0.05(-5.36; 5.56) |
| Finland | male | 9,295.09(7,629.18; 11,168.01) | 8,618.82(7,443.92; 10,443.74) | 0.03(-8.98; 9.92) | 354.46(294.50; 421.43) | 357.30(308.54; 428.00) | 0.27(-5.69; 6.59) |
| France | male | 95,875.07(78,704.33; 114,545.52) | 90,965.90(76,109.42; 107,238.52) | 0.16(-11.08; 12.82) | 336.70(278.90; 399.05) | 315.78(263.87; 373.57) | 0.13(-5.70; 6.32) |
| Gabon | male | 912.54(762.05; 1,089.57) | 1,335.65(1,095.63; 1,623.03) | 1.71(-5.63; 9.62) | 203.49(171.37; 241.17) | 156.74(130.66; 188.83) | -0.45(-5.55; 4.93) |
| Gambia | male | 548.47(459.95; 650.38) | 1,149.15(941.22; 1,362.71) | 3.04(-4.24; 10.87) | 131.97(111.60; 155.17) | 109.64(91.12; 129.85) | 0.11(-4.67; 5.14) |
| Georgia | male | 4,831.37(4,060.63; 5,716.00) | 2,679.03(2,229.85; 3,158.51) | -1.38(-9.17; 7.09) | 178.51(149.72; 211.55) | 159.96(133.90; 187.79) | 0.16(-5.00; 5.60) |
| Germany | male | 132,871.51(110,798.84; 161,208.50) | 121,936.09(103,945.91; 146,898.21) | 0.02(-11.47; 13.01) | 333.83(280.36; 397.99) | 326.54(280.38; 387.87) | 0.35(-5.52; 6.60) |
| Ghana | male | 8,786.94(7,414.93; 10,449.15) | 17,457.00(14,528.53; 20,702.82) | 3.48(-6.52; 14.55) | 137.09(116.93; 159.82) | 111.54(92.75; 130.81) | 0.46(-4.36; 5.51) |
| Greece | male | 12,224.78(9,896.11; 15,029.55) | 9,954.60(8,323.86; 11,798.10) | -0.49(-9.60; 9.55) | 237.20(192.38; 290.77) | 229.95(194.93; 271.36) | 0.14(-5.39; 5.98) |
| Greenland | male | 111.28(93.16; 131.51) | 83.21(70.42; 96.52) | -0.23(-4.72; 4.47) | 346.64(287.93; 411.49) | 309.89(259.50; 363.95) | 0.20(-5.61; 6.36) |
| Grenada | male | 107.59(84.91; 141.35) | 127.36(102.91; 155.97) | 0.79(-4.21; 6.06) | 253.23(203.52; 319.17) | 231.14(184.14; 287.63) | 0.04(-5.51; 5.91) |
| Guam | male | 192.60(155.02; 240.68) | 182.90(145.67; 222.37) | 0.25(-5.06; 5.84) | 237.18(191.63; 300.25) | 231.90(182.22; 285.76) | 0.14(-5.38; 5.99) |
| Guatemala | male | 9,029.26(7,408.56; 10,963.11) | 13,414.51(10,699.59; 16,995.32) | 2.34(-7.26; 12.94) | 286.80(233.11; 353.42) | 176.07(139.90; 222.80) | -0.93(-6.10; 4.53) |
| Guinea | male | 2,794.53(2,335.95; 3,346.92) | 5,740.41(4,770.36; 6,862.09) | 2.77(-6.08; 12.45) | 117.77(99.18; 138.77) | 109.35(91.86; 128.68) | 0.33(-4.46; 5.36) |
| Guinea-Bissau | male | 477.75(399.65; 572.55) | 943.59(781.94; 1,129.41) | 2.90(-4.17; 10.50) | 122.40(103.48; 143.28) | 108.81(90.35; 130.51) | 0.24(-4.54; 5.26) |
| Guyana | male | 833.77(691.30; 1,019.51) | 732.32(582.87; 914.11) | -0.02(-6.67; 7.10) | 203.75(171.33; 242.61) | 185.17(146.81; 230.76) | -0.02(-5.31; 5.57) |
| Haiti | male | 5,551.59(4,478.86; 7,011.10) | 13,292.52(10,265.46; 17,030.77) | 2.76(-6.88; 13.39) | 187.63(154.20; 228.70) | 196.07(153.08; 246.78) | 0.15(-5.18; 5.77) |
| Honduras | male | 4,001.61(3,284.00; 4,873.20) | 9,598.88(7,693.45; 11,817.57) | 3.11(-6.26; 13.41) | 200.13(165.92; 238.14) | 189.94(155.48; 230.02) | 0.08(-5.23; 5.69) |
| Hungary | male | 11,384.76(9,490.75; 13,356.53) | 8,640.19(7,263.62; 10,191.06) | -0.52(-9.49; 9.33) | 225.50(188.43; 265.04) | 208.20(175.50; 242.53) | 0.18(-5.24; 5.91) |
| Iceland | male | 434.50(358.31; 525.67) | 575.53(483.33; 697.05) | 1.37(-5.13; 8.31) | 322.96(266.33; 387.52) | 337.32(285.41; 402.73) | 0.53(-5.38; 6.82) |
| India | male | 604,437.09(496,683.73; 747,639.16) | 1179,885.49(978,268.22; 1388,694.75) | 2.57(-11.32; 18.65) | 141.27(116.90; 169.11) | 148.99(123.73; 174.42) | 0.50(-4.60; 5.88) |
| Indonesia | male | 122,365.19(102,878.86; 145,288.05) | 203,598.49(167,640.15; 239,945.87) | 1.73(-10.44; 15.54) | 130.48(109.67; 153.43) | 131.46(108.41; 155.36) | 0.27(-4.70; 5.51) |
| Iran (Islamic Republic of) | male | 58,422.61(49,814.65; 68,135.10) | 114,663.07(97,732.75; 131,283.30) | 2.56(-9.17; 15.80) | 219.18(186.67; 254.72) | 248.21(212.61; 282.32) | 0.55(-5.07; 6.49) |
| Iraq | male | 12,300.55(10,326.13; 14,790.60) | 30,382.65(25,512.69; 36,257.56) | 3.11(-7.39; 14.81) | 154.37(129.69; 181.91) | 132.71(111.25; 157.98) | -0.24(-5.19; 4.98) |
| Ireland | male | 6,922.86(5,779.36; 8,213.73) | 8,485.49(7,170.94; 9,902.88) | 1.09(-8.01; 11.08) | 370.22(308.76; 439.40) | 373.81(318.58; 434.94) | 0.31(-5.70; 6.69) |
| Israel | male | 7,819.48(6,391.03; 9,486.51) | 12,145.41(9,888.11; 14,973.07) | 1.91(-7.61; 12.40) | 320.74(259.86; 391.87) | 263.88(214.39; 325.38) | -0.09(-5.73; 5.90) |
| Italy | male | 107,155.56(89,206.16; 130,139.40) | 72,589.62(60,990.90; 84,983.14) | -0.84(-11.77; 11.44) | 378.47(312.25; 465.96) | 288.52(238.85; 341.95) | -0.61(-6.32; 5.44) |
| Jamaica | male | 3,173.12(2,546.91; 4,057.10) | 3,348.95(2,750.59; 3,950.54) | 0.50(-7.65; 9.38) | 254.34(207.75; 314.12) | 228.53(186.79; 273.15) | -0.13(-5.62; 5.69) |
| Japan | male | 147,522.83(120,361.86; 179,327.19) | 100,990.81(84,197.65; 119,261.28) | -0.89(-12.11; 11.77) | 226.12(183.36; 276.28) | 197.02(163.10; 237.69) | -0.05(-5.41; 5.61) |
| Jordan | male | 2,776.51(2,297.81; 3,367.12) | 9,264.25(7,632.82; 11,190.16) | 4.70(-4.80; 15.14) | 147.61(123.18; 177.34) | 125.33(102.93; 151.05) | 0.04(-4.87; 5.21) |
| Kazakhstan | male | 21,509.36(17,659.67; 25,598.77) | 25,702.34(21,399.02; 30,599.82) | 0.94(-9.19; 12.21) | 258.87(212.77; 307.53) | 275.74(231.86; 325.43) | 0.35(-5.36; 6.40) |
| Kenya | male | 10,026.82(8,338.73; 12,030.70) | 26,313.31(21,631.95; 32,113.16) | 3.82(-6.62; 15.42) | 104.09(87.72; 122.60) | 104.42(87.62; 123.47) | 0.52(-4.23; 5.51) |
| Kiribati | male | 74.37(58.30; 99.90) | 126.60(100.85; 154.41) | 1.98(-3.04; 7.25) | 198.20(158.92; 256.75) | 200.00(161.78; 240.76) | 0.15(-5.23; 5.83) |
| Kuwait | male | 1,982.59(1,589.27; 2,406.41) | 5,048.57(4,066.79; 6,171.43) | 4.01(-4.82; 13.67) | 164.63(134.01; 198.27) | 168.41(139.03; 202.31) | 0.34(-4.88; 5.85) |
| Kyrgyzstan | male | 4,228.99(3,529.68; 5,035.83) | 6,118.96(5,130.28; 7,217.85) | 1.56(-7.25; 11.22) | 200.41(167.48; 236.08) | 178.34(150.17; 208.25) | -0.04(-5.30; 5.50) |
| Lao People's Democratic Republic | male | 3,023.59(2,415.19; 3,929.78) | 5,956.65(4,807.09; 7,270.02) | 2.46(-6.41; 12.17) | 161.41(132.69; 198.53) | 149.16(121.00; 182.20) | -0.04(-5.12; 5.31) |
| Latvia | male | 4,867.12(4,186.82; 5,627.15) | 2,229.87(1,927.09; 2,575.47) | -2.11(-9.68; 6.09) | 384.20(329.89; 443.71) | 291.05(252.93; 330.65) | -0.53(-6.25; 5.52) |
| Lebanon | male | 2,535.92(2,123.33; 3,002.29) | 5,419.15(4,537.87; 6,329.14) | 3.01(-5.82; 12.67) | 172.22(143.72; 204.11) | 181.34(151.99; 210.00) | 0.72(-4.59; 6.34) |
| Lesotho | male | 1,548.89(1,277.79; 1,905.10) | 1,443.42(1,173.83; 1,763.70) | 0.64(-6.71; 8.56) | 182.22(153.38; 217.15) | 148.31(122.82; 176.78) | -0.03(-5.10; 5.32) |
| Liberia | male | 1,026.75(857.96; 1,231.84) | 3,161.23(2,564.18; 3,864.37) | 4.16(-4.21; 13.27) | 134.33(113.57; 158.50) | 116.56(95.86; 139.46) | 0.14(-4.70; 5.23) |
| Libya | male | 3,744.20(3,083.50; 4,507.22) | 7,706.83(6,316.27; 9,216.23) | 3.44(-5.77; 13.56) | 188.00(156.33; 226.30) | 181.60(149.86; 214.96) | 0.61(-4.70; 6.22) |
| Lithuania | male | 5,261.70(4,394.33; 6,214.18) | 4,098.63(3,550.68; 4,747.43) | -0.82(-9.05; 8.16) | 287.08(239.78; 338.86) | 351.76(306.24; 407.13) | 0.67(-5.29; 7.00) |
| Luxembourg | male | 765.75(626.74; 934.63) | 1,137.19(939.35; 1,399.79) | 1.61(-5.56; 9.33) | 389.44(321.50; 473.74) | 364.05(301.99; 437.55) | 0.00(-5.96; 6.34) |
| Madagascar | male | 7,193.58(5,732.84; 9,538.63) | 16,678.12(13,216.12; 21,542.55) | 3.07(-6.85; 14.04) | 133.09(109.08; 165.82) | 120.44(97.18; 148.09) | -0.06(-4.93; 5.06) |
| Malawi | male | 5,527.04(4,412.52; 7,351.29) | 11,438.00(8,933.90; 15,049.32) | 2.69(-6.82; 13.17) | 129.62(106.37; 161.07) | 120.52(98.75; 146.51) | 0.05(-4.82; 5.17) |
| Malaysia | male | 19,242.02(15,740.20; 23,442.99) | 31,454.20(26,019.46; 36,630.70) | 1.94(-8.49; 13.56) | 214.58(174.84; 261.42) | 171.61(143.58; 198.67) | -0.36(-5.56; 5.13) |
| Maldives | male | 180.81(146.08; 229.26) | 711.44(572.78; 863.43) | 4.88(-2.04; 12.29) | 179.62(146.55; 217.76) | 176.91(144.84; 211.44) | 0.10(-5.15; 5.65) |
| Mali | male | 3,929.64(3,283.82; 4,674.19) | 9,684.29(7,884.66; 11,691.04) | 3.55(-5.88; 13.91) | 117.97(99.04; 138.20) | 102.02(84.32; 122.13) | 0.02(-4.68; 4.96) |
| Malta | male | 521.15(421.33; 629.28) | 588.44(482.75; 711.03) | 0.68(-5.79; 7.60) | 272.47(223.64; 326.94) | 299.60(252.69; 354.11) | 0.54(-5.26; 6.69) |
| Marshall Islands | male | 49.83(38.51; 68.13) | 64.30(50.67; 80.44) | 0.77(-3.51; 5.25) | 218.97(176.53; 277.08) | 203.79(162.87; 253.73) | -0.05(-5.44; 5.64) |
| Mauritania | male | 1,151.35(959.12; 1,365.90) | 2,045.88(1,698.40; 2,442.38) | 2.61(-5.21; 11.08) | 134.91(113.22; 159.08) | 110.98(92.65; 131.39) | 0.10(-4.69; 5.14) |
| Mauritius | male | 1,250.65(1,021.52; 1,529.91) | 1,815.87(1,485.37; 2,292.28) | 1.28(-6.36; 9.54) | 204.71(167.36; 248.37) | 280.90(228.24; 354.34) | 1.12(-4.67; 7.25) |
| Mexico | male | 68,277.68(57,378.27; 80,862.95) | 97,488.52(82,694.27; 112,182.57) | 1.66(-9.81; 14.58) | 167.17(140.80; 196.30) | 145.37(123.21; 167.56) | 0.11(-4.96; 5.44) |
| Micronesia (Federated States of) | male | 118.79(92.36; 161.17) | 114.52(90.73; 146.13) | -0.10(-4.92; 4.97) | 225.13(183.23; 283.70) | 198.15(159.73; 249.23) | -0.21(-5.56; 5.44) |
| Monaco | male | 46.97(37.79; 57.23) | 49.32(40.23; 59.02) | 0.59(-3.43; 4.78) | 355.66(288.23; 438.43) | 333.24(271.10; 405.82) | 0.18(-5.72; 6.44) |
| Mongolia | male | 2,002.33(1,637.83; 2,447.61) | 3,008.50(2,526.06; 3,558.51) | 1.98(-6.18; 10.85) | 187.75(156.46; 225.54) | 183.64(154.23; 216.34) | 0.38(-4.92; 5.99) |
| Montenegro | male | 832.10(688.35; 990.64) | 570.39(474.41; 675.75) | -0.66(-7.03; 6.15) | 251.81(209.22; 300.67) | 188.63(158.13; 222.27) | -0.35(-5.65; 5.26) |
| Morocco | male | 18,567.60(15,453.68; 22,584.69) | 29,414.42(24,640.53; 34,649.74) | 1.82(-8.53; 13.34) | 154.05(127.99; 185.25) | 150.37(125.96; 176.77) | 0.29(-4.81; 5.67) |
| Mozambique | male | 7,177.53(5,739.36; 9,535.34) | 19,158.93(15,020.30; 24,390.62) | 3.48(-6.61; 14.66) | 132.76(108.98; 165.77) | 146.17(117.88; 177.21) | 0.65(-4.44; 6.02) |
| Myanmar | male | 37,979.31(30,561.29; 47,955.34) | 49,357.70(39,680.51; 61,325.14) | 0.93(-9.82; 12.96) | 180.95(148.01; 221.67) | 170.51(136.74; 210.36) | -0.01(-5.23; 5.49) |
| Namibia | male | 1,404.72(1,182.18; 1,701.22) | 1,991.82(1,629.27; 2,375.00) | 1.64(-6.09; 10.01) | 209.64(177.52; 246.37) | 160.83(132.71; 189.96) | -0.28(-5.43; 5.14) |
| Nauru | male | 11.48(9.09; 15.03) | 12.19(9.53; 15.69) | 0.17(-2.40; 2.82) | 220.75(177.58; 283.21) | 203.07(163.63; 254.84) | -0.12(-5.50; 5.57) |
| Nepal | male | 10,617.81(8,513.34; 12,979.68) | 18,230.53(14,958.42; 22,054.43) | 1.94(-7.96; 12.90) | 125.00(101.51; 150.09) | 121.41(100.15; 145.80) | 0.15(-4.73; 5.28) |
| Netherlands | male | 26,499.40(22,223.21; 31,319.13) | 21,107.06(17,924.48; 24,757.64) | -0.30(-10.14; 10.62) | 345.43(290.47; 404.98) | 279.57(235.24; 328.94) | -0.26(-5.97; 5.79) |
| New Zealand | male | 8,130.28(6,638.50; 9,922.67) | 10,843.42(8,926.40; 13,038.13) | 0.70(-8.58; 10.91) | 454.48(371.50; 556.07) | 449.81(368.19; 550.31) | 0.04(-6.12; 6.60) |
| Nicaragua | male | 3,077.43(2,570.36; 3,620.84) | 4,568.60(3,722.89; 5,448.33) | 1.68(-6.87; 11.00) | 182.18(152.97; 213.09) | 130.98(106.95; 155.54) | -0.62(-5.54; 4.56) |
| Niger | male | 3,709.17(3,073.45; 4,425.11) | 9,472.89(7,776.92; 11,427.11) | 3.58(-5.82; 13.93) | 119.74(100.08; 141.69) | 100.27(83.47; 118.63) | 0.03(-4.66; 4.95) |
| Nigeria | male | 46,847.43(39,871.57; 54,180.13) | 98,767.59(83,110.98; 116,487.61) | 3.16(-8.49; 16.29) | 116.36(99.38; 134.68) | 108.27(91.39; 126.24) | 0.48(-4.32; 5.51) |
| Niue | male | 2.57(2.03; 3.42) | 1.80(1.44; 2.27) | -1.23(-1.88; -0.59) | 224.05(181.21; 287.08) | 212.87(170.58; 266.56) | 0.02(-5.41; 5.77) |
| North Macedonia | male | 1,929.12(1,580.56; 2,319.13) | 1,975.60(1,646.13; 2,326.31) | 0.49(-7.16; 8.76) | 179.72(147.12; 215.51) | 176.03(147.49; 205.13) | 0.25(-5.01; 5.80) |
| Northern Mariana Islands | male | 62.28(50.45; 76.29) | 56.49(45.95; 69.47) | -0.72(-4.84; 3.58) | 225.18(180.82; 284.48) | 221.67(179.98; 273.66) | -0.05(-5.52; 5.74) |
| Norway | male | 7,779.97(6,338.85; 9,314.29) | 9,517.16(8,087.03; 10,983.94) | 1.00(-8.20; 11.12) | 351.16(286.61; 420.15) | 358.57(305.09; 413.77) | 0.29(-5.67; 6.63) |
| Oman | male | 1,632.14(1,306.84; 2,008.84) | 4,699.67(3,782.71; 5,744.43) | 4.66(-4.17; 14.31) | 136.71(110.74; 166.71) | 126.75(104.51; 150.18) | 0.12(-4.80; 5.30) |
| Pakistan | male | 76,902.20(63,395.76; 92,526.75) | 146,689.12(120,647.52; 176,260.52) | 2.64(-9.31; 16.17) | 149.58(124.92; 176.21) | 123.05(101.49; 145.54) | -0.07(-4.96; 5.07) |
| Palau | male | 19.90(15.93; 25.64) | 21.58(17.56; 25.84) | 0.08(-3.10; 3.37) | 224.95(181.00; 286.39) | 212.10(168.37; 264.61) | -0.04(-5.47; 5.70) |
| Palestine | male | 1,285.16(1,046.25; 1,612.98) | 3,729.84(3,049.40; 4,532.15) | 3.72(-4.79; 13.00) | 144.94(119.69; 174.57) | 135.61(111.35; 163.20) | 0.08(-4.92; 5.33) |
| Panama | male | 2,747.61(2,251.46; 3,390.20) | 3,768.84(3,022.07; 4,566.71) | 1.43(-6.90; 10.51) | 218.75(181.24; 261.89) | 170.42(136.30; 206.59) | -0.37(-5.56; 5.11) |
| Papua New Guinea | male | 4,315.26(3,340.65; 5,862.07) | 10,821.52(8,439.01; 13,840.00) | 3.22(-6.29; 13.70) | 196.07(155.14; 254.74) | 192.31(153.23; 241.38) | 0.02(-5.31; 5.66) |
| Paraguay | male | 3,184.78(2,671.64; 3,763.76) | 5,610.91(4,629.39; 6,644.01) | 2.23(-6.57; 11.86) | 165.23(139.47; 193.54) | 143.21(117.66; 169.79) | -0.09(-5.13; 5.23) |
| Peru | male | 25,848.93(21,528.98; 30,650.47) | 28,536.30(24,113.10; 33,617.58) | 1.21(-9.04; 12.63) | 233.48(197.49; 273.68) | 147.92(124.68; 174.03) | -0.46(-5.51; 4.87) |
| Philippines | male | 63,112.82(53,127.29; 75,679.55) | 89,326.08(74,202.01; 106,935.90) | 1.34(-10.00; 14.12) | 195.90(166.75; 230.16) | 143.90(119.56; 170.64) | -0.80(-5.81; 4.47) |
| Poland | male | 46,490.26(38,416.30; 56,892.13) | 37,056.48(30,609.23; 43,408.62) | -0.47(-10.81; 11.06) | 247.63(204.54; 304.57) | 223.11(183.20; 268.65) | 0.02(-5.45; 5.82) |
| Portugal | male | 14,386.02(11,884.10; 17,014.74) | 10,183.60(8,515.00; 11,992.38) | -0.64(-9.76; 9.41) | 285.84(235.75; 339.57) | 227.13(190.61; 265.44) | -0.22(-5.71; 5.59) |
| Puerto Rico | male | 7,381.67(6,157.62; 8,972.64) | 4,606.23(3,841.56; 5,696.92) | -1.18(-9.51; 7.91) | 411.50(344.78; 496.11) | 311.00(256.19; 389.78) | -0.63(-6.40; 5.49) |
| Qatar | male | 629.37(506.98; 769.33) | 3,965.50(3,128.85; 4,934.07) | 8.47(-0.55; 18.30) | 163.98(137.42; 194.42) | 130.70(106.91; 157.33) | -0.41(-5.35; 4.78) |
| Republic of Korea | male | 61,635.15(49,522.45; 75,707.50) | 51,961.93(42,415.27; 61,839.06) | 0.01(-10.70; 12.02) | 233.63(189.28; 285.14) | 212.10(172.04; 260.25) | 0.05(-5.39; 5.80) |
| Republic of Moldova | male | 5,770.97(4,851.53; 6,744.09) | 3,335.78(2,788.36; 3,873.21) | -1.08(-9.11; 7.65) | 266.49(223.72; 311.54) | 192.17(162.15; 221.13) | -0.45(-5.76; 5.16) |
| Romania | male | 20,056.89(16,303.45; 24,095.77) | 13,940.85(11,475.81; 16,699.12) | -0.82(-10.21; 9.55) | 169.77(137.64; 204.00) | 149.37(124.57; 176.46) | 0.01(-5.07; 5.37) |
| Russian Federation | male | 297,290.84(253,289.61; 343,555.05) | 221,198.45(192,431.42; 252,352.75) | -0.76(-12.71; 12.83) | 398.67(340.25; 460.67) | 345.62(300.49; 393.84) | -0.27(-6.17; 5.99) |
| Rwanda | male | 4,456.10(3,461.61; 5,951.33) | 9,634.22(7,382.65; 12,418.39) | 3.39(-6.03; 13.76) | 139.04(112.65; 173.98) | 139.44(111.07; 173.15) | 0.32(-4.71; 5.61) |
| Saint Kitts and Nevis | male | 50.73(40.09; 67.19) | 70.61(57.11; 86.90) | 1.44(-2.96; 6.05) | 246.59(199.46; 313.08) | 227.94(180.43; 289.40) | -0.06(-5.55; 5.76) |
| Saint Lucia | male | 226.70(176.15; 297.65) | 216.64(172.57; 272.08) | 0.51(-5.05; 6.39) | 305.31(244.98; 387.18) | 242.69(186.61; 323.10) | -0.09(-5.73; 5.90) |
| Saint Vincent and the Grenadines | male | 142.30(112.11; 189.72) | 141.60(112.92; 175.86) | 0.06(-4.98; 5.37) | 248.16(201.21; 313.22) | 245.22(192.49; 306.15) | 0.10(-5.48; 6.01) |
| Samoa | male | 221.49(166.52; 312.43) | 287.95(227.46; 348.62) | 0.87(-4.91; 6.99) | 231.42(183.63; 303.63) | 248.16(200.98; 296.12) | 0.15(-5.45; 6.07) |
| San Marino | male | 42.11(34.36; 50.49) | 44.59(36.68; 54.79) | 0.70(-3.21; 4.77) | 347.29(282.63; 423.37) | 324.77(265.46; 408.71) | 0.13(-5.72; 6.35) |
| Sao Tome and Principe | male | 64.63(53.66; 77.02) | 121.78(100.57; 144.83) | 2.75(-2.25; 8.01) | 133.86(112.77; 157.27) | 113.67(94.48; 135.49) | 0.05(-4.77; 5.10) |
| Saudi Arabia | male | 13,164.22(10,657.40; 15,995.54) | 39,112.70(31,951.21; 47,643.64) | 4.27(-6.61; 16.40) | 145.15(118.66; 175.32) | 136.47(112.92; 162.38) | 0.30(-4.71; 5.57) |
| Senegal | male | 3,992.42(3,325.48; 4,726.74) | 7,783.02(6,442.81; 9,246.46) | 2.70(-6.44; 12.74) | 133.54(112.31; 156.20) | 109.67(91.40; 129.90) | -0.07(-4.84; 4.94) |
| Serbia | male | 9,239.94(7,463.27; 11,190.28) | 8,325.07(6,907.54; 9,757.46) | -0.48(-9.43; 9.34) | 195.63(158.21; 237.84) | 190.39(159.46; 222.40) | 0.16(-5.18; 5.80) |
| Seychelles | male | 101.44(82.36; 127.55) | 117.51(95.70; 140.85) | 0.90(-3.99; 6.04) | 256.91(210.12; 316.21) | 208.02(167.89; 253.25) | -0.27(-5.66; 5.44) |
| Sierra Leone | male | 2,033.48(1,687.58; 2,413.93) | 4,313.28(3,588.10; 5,118.55) | 3.81(-4.84; 13.25) | 134.08(112.86; 157.78) | 106.99(89.77; 125.67) | -0.02(-4.77; 4.97) |
| Singapore | male | 4,451.99(3,569.98; 5,435.03) | 6,006.33(4,878.16; 7,182.08) | 1.65(-7.18; 11.33) | 237.36(192.92; 290.70) | 213.59(174.29; 259.74) | 0.01(-5.44; 5.76) |
| Slovakia | male | 6,771.30(5,672.56; 7,920.33) | 5,180.24(4,367.02; 6,050.74) | -0.67(-9.15; 8.59) | 252.84(211.98; 295.63) | 211.66(180.81; 245.65) | -0.30(-5.71; 5.42) |
| Slovenia | male | 2,690.74(2,191.23; 3,196.48) | 2,413.15(2,084.13; 2,776.65) | -0.10(-7.89; 8.35) | 270.29(219.89; 322.76) | 283.87(246.78; 323.48) | 0.44(-5.30; 6.53) |
| Solomon Islands | male | 352.55(267.66; 490.32) | 673.37(549.72; 823.07) | 2.18(-4.51; 9.35) | 200.49(160.66; 260.83) | 181.82(151.16; 217.72) | -0.10(-5.37; 5.47) |
| Somalia | male | 4,225.70(3,310.25; 5,789.74) | 11,474.27(8,702.73; 15,230.74) | 3.54(-6.05; 14.11) | 124.39(101.77; 155.82) | 108.41(87.03; 135.20) | -0.10(-4.87; 4.90) |
| South Africa | male | 64,283.39(54,151.29; 75,821.94) | 58,982.52(49,567.10; 69,396.75) | -0.27(-11.01; 11.77) | 329.08(281.45; 384.15) | 195.03(163.69; 229.32) | -1.64(-6.85; 3.87) |
| South Sudan | male | 4,002.00(3,169.17; 5,294.28) | 5,420.27(4,119.50; 7,349.53) | 1.33(-7.34; 10.81) | 135.77(109.81; 168.86) | 118.52(96.58; 145.60) | -0.04(-4.90; 5.06) |
| Spain | male | 85,448.09(74,876.29; 96,912.33) | 73,540.62(62,568.59; 86,680.53) | -0.17(-11.18; 12.20) | 432.66(378.38; 490.73) | 388.02(334.62; 443.11) | -0.19(-6.20; 6.21) |
| Sri Lanka | male | 20,214.29(16,646.61; 24,621.60) | 19,745.43(16,484.94; 23,526.39) | -0.01(-9.80; 10.85) | 221.83(183.77; 267.47) | 179.94(149.80; 214.12) | -0.49(-5.74; 5.05) |
| Sudan | male | 11,359.09(9,313.72; 14,051.68) | 29,741.85(24,885.84; 35,547.94) | 3.25(-7.25; 14.93) | 131.55(107.25; 158.13) | 136.82(114.52; 163.49) | 0.40(-4.62; 5.69) |
| Suriname | male | 459.56(371.56; 593.94) | 642.56(524.16; 776.41) | 1.59(-5.03; 8.68) | 227.72(186.01; 283.54) | 219.17(178.27; 264.16) | 0.16(-5.31; 5.95) |
| Sweden | male | 12,766.47(10,218.35; 15,595.70) | 16,546.03(14,026.75; 19,592.31) | 1.37(-8.39; 12.16) | 295.38(236.75; 362.07) | 349.59(296.52; 410.24) | 1.04(-4.95; 7.39) |
| Switzerland | male | 17,527.58(14,201.31; 21,558.17) | 15,394.82(12,634.82; 18,401.09) | 0.08(-9.49; 10.66) | 515.83(417.71; 634.43) | 390.86(325.89; 461.34) | -0.50(-6.51; 5.89) |
| Syrian Arab Republic | male | 6,636.67(5,433.81; 8,000.00) | 8,084.74(6,692.58; 9,671.82) | 1.16(-7.91; 11.12) | 122.40(100.68; 146.74) | 118.83(98.92; 142.23) | 0.48(-4.40; 5.62) |
| Taiwan (Province of China) | male | 26,272.09(21,141.78; 32,106.81) | 30,299.23(24,433.15; 36,536.14) | 0.61(-9.65; 12.05) | 229.63(184.85; 281.23) | 248.17(204.87; 295.96) | 0.39(-5.22; 6.33) |
| Tajikistan | male | 4,306.74(3,531.28; 5,273.09) | 8,050.27(6,614.07; 9,731.48) | 2.58(-6.59; 12.64) | 173.37(142.73; 208.70) | 153.09(126.40; 184.09) | -0.03(-5.14; 5.34) |
| Thailand | male | 69,444.08(56,783.89; 84,884.28) | 59,098.63(49,385.83; 69,406.88) | 0.00(-10.83; 12.14) | 212.24(175.36; 258.21) | 194.93(161.72; 230.13) | 0.03(-5.32; 5.69) |
| Timor-Leste | male | 671.46(545.16; 841.31) | 1,176.90(939.59; 1,477.23) | 2.05(-5.19; 9.84) | 167.56(136.64; 206.02) | 161.00(131.75; 193.99) | 0.12(-5.05; 5.57) |
| Togo | male | 1,645.00(1,378.96; 1,961.60) | 3,859.86(3,233.33; 4,561.22) | 3.60(-4.93; 12.89) | 117.77(99.05; 139.47) | 105.48(89.07; 125.05) | 0.42(-4.34; 5.41) |
| Tokelau | male | 1.61(1.25; 2.16) | 1.46(1.16; 1.87) | -0.30(-0.75; 0.14) | 211.67(169.84; 271.44) | 205.41(164.95; 257.33) | 0.09(-5.32; 5.80) |
| Tonga | male | 100.99(77.84; 139.20) | 104.04(84.60; 127.16) | 0.20(-4.53; 5.17) | 203.56(163.69; 259.12) | 197.82(163.19; 236.53) | 0.06(-5.30; 5.73) |
| Trinidad and Tobago | male | 1,434.39(1,138.37; 1,889.88) | 1,486.37(1,241.50; 1,755.49) | 0.30(-7.06; 8.23) | 227.98(184.81; 294.10) | 214.43(178.58; 255.01) | 0.19(-5.26; 5.95) |
| Tunisia | male | 5,739.64(4,693.14; 7,013.77) | 9,037.60(7,447.39; 10,829.56) | 1.82(-7.40; 11.96) | 143.75(118.29; 172.73) | 148.20(122.74; 176.59) | 0.52(-4.59; 5.89) |
| Turkey | male | 39,348.46(32,441.35; 47,034.29) | 53,616.97(43,200.66; 65,281.20) | 1.57(-9.33; 13.77) | 132.62(109.42; 159.38) | 118.42(96.15; 143.93) | 0.12(-4.74; 5.23) |
| Turkmenistan | male | 3,235.89(2,664.08; 3,901.55) | 5,060.76(4,184.91; 6,004.75) | 1.79(-6.87; 11.24) | 180.69(150.34; 213.83) | 178.58(148.14; 212.17) | 0.29(-4.99; 5.86) |
| Tuvalu | male | 8.82(6.99; 11.61) | 13.57(10.78; 17.15) | 1.65(-1.07; 4.44) | 204.09(164.40; 261.35) | 197.70(157.64; 249.06) | 0.02(-5.34; 5.69) |
| Uganda | male | 12,127.54(9,321.40; 16,437.45) | 27,950.20(22,175.91; 34,633.88) | 3.17(-7.26; 14.76) | 150.51(121.55; 190.65) | 133.08(110.05; 157.04) | -0.02(-4.98; 5.21) |
| Ukraine | male | 72,245.63(60,833.04; 84,063.51) | 52,963.48(45,783.99; 60,440.71) | -0.55(-11.22; 11.39) | 285.33(241.61; 331.62) | 273.94(239.37; 313.87) | 0.27(-5.43; 6.31) |
| United Arab Emirates | male | 3,228.09(2,568.39; 4,046.68) | 19,281.32(15,047.18; 24,278.68) | 7.47(-3.07; 19.16) | 203.59(167.77; 246.95) | 200.74(170.46; 234.28) | 0.38(-5.02; 6.09) |
| United Kingdom | male | 121,674.75(101,699.28; 147,238.46) | 129,995.57(111,205.31; 152,140.47) | 0.84(-10.81; 14.02) | 452.85(377.21; 554.08) | 444.10(371.67; 527.35) | 0.43(-5.76; 7.02) |
| United Republic of Tanzania | male | 16,704.87(13,378.89; 21,374.97) | 36,704.53(29,219.23; 46,754.86) | 2.82(-7.83; 14.70) | 145.19(119.95; 175.68) | 137.51(112.86; 166.68) | 0.12(-4.88; 5.39) |
| United States of America | male | 601,990.01(512,568.61; 707,283.67) | 836,103.95(734,103.03; 951,329.82) | 1.44(-11.98; 16.90) | 479.00(408.87; 566.49) | 563.74(491.35; 642.65) | 0.86(-5.57; 7.72) |
| United States Virgin Islands | male | 169.27(135.51; 211.85) | 106.29(86.12; 132.36) | -0.82(-5.54; 4.14) | 314.41(254.04; 389.92) | 290.03(233.84; 363.84) | 0.06(-5.68; 6.15) |
| Uruguay | male | 4,502.35(3,801.60; 5,246.10) | 3,717.27(3,137.46; 4,324.32) | 0.05(-8.17; 9.01) | 297.07(250.30; 345.99) | 234.45(198.14; 272.56) | -0.09(-5.62; 5.75) |
| Uzbekistan | male | 16,966.07(13,893.94; 20,472.72) | 27,996.37(23,380.93; 33,110.67) | 2.24(-8.11; 13.74) | 169.37(139.37; 203.32) | 158.46(132.36; 187.58) | 0.24(-4.92; 5.67) |
| Vanuatu | male | 140.65(110.01; 187.09) | 295.07(241.45; 353.30) | 2.49(-3.39; 8.74) | 187.89(151.46; 241.18) | 181.18(150.69; 213.25) | 0.01(-5.26; 5.58) |
| Venezuela (Bolivarian Republic of) | male | 16,170.79(13,334.69; 19,390.03) | 18,166.89(14,764.03; 21,743.43) | 1.32(-8.54; 12.24) | 173.43(143.34; 207.20) | 139.92(114.84; 167.21) | -0.18(-5.19; 5.09) |
| Viet Nam | male | 64,210.25(52,129.09; 79,813.53) | 107,579.14(87,724.58; 127,320.27) | 1.88(-9.71; 14.95) | 194.88(160.41; 234.12) | 206.51(168.32; 245.39) | 0.34(-5.08; 6.07) |
| Yemen | male | 7,066.73(5,735.64; 8,837.41) | 21,694.99(17,740.26; 26,089.58) | 3.98(-6.28; 15.37) | 130.60(107.02; 156.69) | 132.57(108.81; 156.49) | 0.39(-4.60; 5.63) |
| Zambia | male | 5,386.24(4,463.94; 6,637.39) | 11,396.82(9,387.85; 14,037.15) | 3.09(-6.46; 13.62) | 149.69(126.48; 177.46) | 124.98(104.50; 147.82) | -0.14(-5.04; 5.01) |
| Zimbabwe | male | 10,448.88(8,583.52; 12,871.97) | 13,185.31(10,505.49; 16,376.36) | 0.89(-8.59; 11.36) | 233.37(195.07; 278.82) | 186.01(149.67; 226.37) | -0.39(-5.67; 5.17) |

**Supplementary Table 4 Incidence of drug use disorders in 204 countries in 2021 by countries and ages.**

|  | 15-19 years | 20-24 years | 25-29 years | 30-34 years | 35-39 years | 40-44 years | 45-49 years | 50-54 years | 55-59 years | 60-64 years | 65-69 years | 70-74 years | 75-79 years | 80-84 | 85-89 | 90-94 | 95+ years |
| --- | --- | --- | --- | --- | --- | --- | --- | --- | --- | --- | --- | --- | --- | --- | --- | --- | --- |
| Paraguay | 580.51 | 754.68 | 547.08 | 525.94 | 584.83 | 588.65 | 544.31 | 405.66 | 170.57 | 48.91 | 40.65 | 35.65 | 33.97 | 36.97 | 44.86 | 58.15 | 78.69 |
| Australia | 928.91 | 863.54 | 735.41 | 751.69 | 778.79 | 701.27 | 521.98 | 337.57 | 150.20 | 52.82 | 45.75 | 40.97 | 38.59 | 39.89 | 44.51 | 50.40 | 56.58 |
| Dominican Republic | 579.51 | 543.61 | 531.75 | 577.34 | 599.82 | 551.66 | 435.66 | 295.52 | 128.29 | 41.52 | 35.50 | 31.30 | 28.94 | 30.15 | 34.78 | 40.69 | 48.03 |
| Zimbabwe | 1,736.67 | 1,516.63 | 856.47 | 626.89 | 600.76 | 525.73 | 421.94 | 294.89 | 135.91 | 51.45 | 42.28 | 38.88 | 41.29 | 42.98 | 44.04 | 45.00 | 45.76 |
| Japan | 768.81 | 561.97 | 531.30 | 544.69 | 583.20 | 539.18 | 417.24 | 281.56 | 128.03 | 46.40 | 37.14 | 30.03 | 25.11 | 25.49 | 30.59 | 35.46 | 39.77 |
| Yemen | 1,080.95 | 751.08 | 568.51 | 584.53 | 640.03 | 576.21 | 410.22 | 260.29 | 121.26 | 45.81 | 33.88 | 25.58 | 20.94 | 20.84 | 25.01 | 30.07 | 36.25 |
| Iraq | 656.78 | 680.38 | 561.68 | 541.62 | 558.56 | 511.11 | 403.17 | 274.20 | 118.88 | 41.93 | 44.16 | 48.63 | 56.00 | 71.78 | 97.16 | 115.83 | 125.25 |
| Togo | 905.53 | 510.53 | 512.00 | 565.52 | 627.52 | 570.69 | 400.04 | 249.51 | 114.90 | 43.02 | 34.01 | 27.28 | 22.80 | 25.16 | 34.16 | 49.23 | 71.57 |
| Mozambique | 601.62 | 469.09 | 484.67 | 543.27 | 565.42 | 516.08 | 399.46 | 267.74 | 118.24 | 41.79 | 38.20 | 36.91 | 37.55 | 40.65 | 45.99 | 51.40 | 56.71 |
| Northern Mariana Islands | 1,170.54 | 1,107.22 | 595.50 | 558.22 | 570.39 | 514.94 | 397.65 | 265.60 | 125.01 | 48.84 | 37.17 | 28.35 | 22.56 | 22.97 | 29.12 | 36.20 | 43.34 |
| Georgia | 609.40 | 721.40 | 613.98 | 579.33 | 535.78 | 473.84 | 395.65 | 282.85 | 129.99 | 49.00 | 40.49 | 33.87 | 29.16 | 29.81 | 35.60 | 42.24 | 49.78 |
| Samoa | 554.65 | 324.28 | 363.12 | 455.81 | 537.14 | 515.53 | 395.33 | 260.70 | 110.49 | 34.41 | 32.08 | 32.50 | 35.66 | 38.10 | 39.84 | 42.38 | 45.80 |
| Malaysia | 735.82 | 567.03 | 542.57 | 556.35 | 543.08 | 484.60 | 385.29 | 264.14 | 117.10 | 43.17 | 42.39 | 44.54 | 49.83 | 60.08 | 75.15 | 88.82 | 102.02 |
| Timor-Leste | 508.37 | 691.71 | 592.13 | 571.58 | 533.47 | 471.09 | 383.01 | 266.85 | 123.62 | 47.07 | 37.17 | 29.87 | 25.23 | 27.20 | 35.84 | 46.18 | 57.85 |
| Monaco | 535.85 | 299.48 | 302.72 | 381.76 | 472.54 | 466.22 | 367.81 | 248.17 | 105.91 | 34.30 | 32.39 | 33.27 | 36.86 | 39.14 | 40.18 | 42.05 | 44.81 |
| Somalia | 600.85 | 462.97 | 467.15 | 516.52 | 536.74 | 485.54 | 367.61 | 243.76 | 109.59 | 40.33 | 35.99 | 33.69 | 33.55 | 36.04 | 40.99 | 46.34 | 52.50 |
| Andorra | 603.00 | 453.71 | 465.00 | 514.13 | 525.96 | 472.41 | 353.84 | 233.21 | 106.53 | 39.21 | 31.33 | 25.60 | 21.98 | 22.50 | 26.61 | 31.10 | 35.88 |
| Grenada | 633.68 | 510.13 | 490.08 | 514.80 | 516.85 | 461.86 | 347.12 | 226.81 | 102.15 | 37.17 | 32.02 | 28.40 | 26.32 | 27.75 | 32.55 | 37.60 | 42.71 |
| Austria | 646.95 | 535.48 | 471.60 | 474.01 | 505.93 | 461.78 | 345.03 | 226.96 | 103.78 | 38.16 | 30.31 | 24.28 | 20.04 | 20.34 | 24.97 | 29.68 | 34.25 |
| Germany | 743.16 | 387.02 | 396.15 | 440.58 | 465.93 | 432.19 | 343.54 | 236.89 | 107.44 | 43.08 | 44.33 | 51.48 | 64.69 | 86.00 | 115.84 | 125.49 | 112.47 |
| Central African Republic | 1,435.85 | 741.80 | 569.87 | 505.96 | 493.36 | 434.70 | 341.92 | 234.15 | 107.78 | 41.36 | 35.00 | 31.36 | 30.45 | 33.47 | 40.45 | 48.43 | 57.50 |
| United Republic of Tanzania | 1,092.41 | 462.44 | 443.17 | 464.38 | 491.48 | 449.51 | 338.23 | 223.46 | 101.80 | 39.69 | 37.39 | 38.53 | 43.11 | 52.12 | 65.77 | 73.79 | 75.70 |
| Fiji | 1,045.16 | 1,183.65 | 835.33 | 625.79 | 509.51 | 413.01 | 335.46 | 236.15 | 113.56 | 47.33 | 38.38 | 31.58 | 27.19 | 26.87 | 29.76 | 34.74 | 42.16 |
| Seychelles | 618.09 | 666.69 | 561.17 | 522.22 | 473.33 | 408.44 | 333.92 | 238.56 | 120.53 | 57.61 | 50.71 | 45.82 | 43.28 | 44.32 | 46.95 | 55.12 | 68.35 |
| Belize | 663.58 | 395.95 | 426.56 | 465.16 | 483.21 | 437.19 | 330.33 | 219.70 | 101.84 | 41.03 | 37.64 | 37.29 | 40.05 | 44.95 | 51.31 | 59.08 | 68.22 |
| Belgium | 415.60 | 414.31 | 378.37 | 383.48 | 384.57 | 364.95 | 327.13 | 244.90 | 115.40 | 47.29 | 41.43 | 45.67 | 62.52 | 70.44 | 64.95 | 66.68 | 77.81 |
| Trinidad and Tobago | 314.41 | 447.02 | 386.33 | 409.18 | 462.05 | 429.35 | 319.93 | 211.47 | 101.39 | 45.13 | 42.73 | 39.99 | 36.93 | 36.78 | 39.42 | 44.00 | 50.38 |
| Malawi | 503.39 | 563.87 | 558.92 | 537.11 | 469.52 | 396.15 | 318.50 | 223.66 | 108.79 | 47.58 | 40.32 | 34.77 | 31.96 | 32.34 | 34.66 | 39.69 | 46.96 |
| Kyrgyzstan | 350.11 | 474.23 | 475.82 | 472.35 | 423.81 | 370.21 | 317.04 | 231.38 | 111.34 | 49.85 | 47.72 | 53.70 | 67.01 | 73.97 | 75.44 | 78.50 | 82.76 |
| Jordan | 443.79 | 385.63 | 389.37 | 424.66 | 444.65 | 404.85 | 307.40 | 205.14 | 94.67 | 36.63 | 31.00 | 27.27 | 25.40 | 30.34 | 42.31 | 49.77 | 52.81 |
| Libya | 569.96 | 525.34 | 427.18 | 387.12 | 377.54 | 349.44 | 304.40 | 225.40 | 110.39 | 49.13 | 42.20 | 36.13 | 31.41 | 30.58 | 32.78 | 36.71 | 42.69 |
| Kazakhstan | 582.23 | 464.19 | 402.96 | 416.23 | 436.79 | 396.60 | 302.13 | 204.46 | 98.63 | 41.87 | 34.19 | 29.77 | 28.59 | 31.54 | 38.40 | 45.92 | 54.26 |
| Bhutan | 530.30 | 298.70 | 284.87 | 332.27 | 376.32 | 365.30 | 301.43 | 210.90 | 93.27 | 33.17 | 29.77 | 29.81 | 33.41 | 36.95 | 40.35 | 46.27 | 54.49 |
| Mauritius | 533.04 | 518.83 | 465.06 | 457.09 | 451.41 | 397.70 | 299.69 | 198.88 | 91.47 | 35.93 | 32.36 | 30.63 | 30.65 | 34.06 | 40.72 | 46.38 | 52.04 |
| Democratic People's Republic of Korea | 629.53 | 415.84 | 408.18 | 426.62 | 426.87 | 382.67 | 297.86 | 203.27 | 94.82 | 37.26 | 30.66 | 26.61 | 25.16 | 28.22 | 35.59 | 43.70 | 52.60 |
| United States Virgin Islands | 281.36 | 371.71 | 302.94 | 308.91 | 356.82 | 347.69 | 284.60 | 201.99 | 102.00 | 48.31 | 41.12 | 38.04 | 39.00 | 39.88 | 40.69 | 44.52 | 51.45 |
| Bahrain | 627.99 | 271.11 | 280.52 | 332.52 | 378.44 | 359.58 | 281.48 | 190.30 | 86.61 | 31.77 | 25.46 | 21.84 | 20.93 | 22.13 | 25.53 | 29.60 | 34.42 |
| Czechia | 378.31 | 358.35 | 392.72 | 416.88 | 401.65 | 356.62 | 281.22 | 192.38 | 88.65 | 34.13 | 28.42 | 25.66 | 25.71 | 28.46 | 34.10 | 41.47 | 39.60 |
| Haiti | 218.65 | 215.94 | 224.32 | 273.52 | 330.80 | 333.82 | 280.71 | 201.09 | 97.46 | 41.42 | 33.21 | 27.00 | 22.81 | 22.02 | 24.65 | 27.78 | 31.97 |
| Guinea | 1,008.04 | 595.82 | 426.79 | 408.16 | 383.02 | 334.12 | 276.59 | 199.53 | 95.33 | 38.72 | 30.05 | 23.81 | 20.41 | 20.15 | 23.09 | 26.65 | 30.95 |
| Madagascar | 233.07 | 300.69 | 280.63 | 308.45 | 364.80 | 353.68 | 276.39 | 189.46 | 93.73 | 43.60 | 39.16 | 38.00 | 40.14 | 41.88 | 43.24 | 46.54 | 51.08 |
| Mongolia | 437.43 | 381.62 | 371.40 | 387.06 | 391.12 | 349.44 | 274.34 | 187.89 | 87.95 | 35.53 | 30.07 | 26.24 | 23.94 | 24.31 | 27.89 | 32.85 | 40.01 |
| Barbados | 153.97 | 208.08 | 197.53 | 240.29 | 289.04 | 301.27 | 274.25 | 206.32 | 100.95 | 46.50 | 43.40 | 42.04 | 42.41 | 43.35 | 44.87 | 46.66 | 48.92 |
| Guatemala | 407.89 | 396.12 | 360.56 | 372.52 | 380.54 | 346.27 | 271.21 | 185.72 | 86.00 | 32.69 | 26.00 | 21.03 | 17.73 | 17.55 | 20.42 | 23.71 | 27.99 |
| Italy | 381.37 | 486.86 | 338.21 | 309.74 | 356.92 | 341.29 | 268.62 | 186.52 | 94.39 | 44.59 | 37.18 | 34.61 | 36.94 | 38.37 | 39.35 | 40.99 | 40.92 |
| Viet nam | 430.92 | 547.14 | 433.88 | 390.14 | 362.65 | 321.33 | 268.59 | 195.11 | 98.04 | 44.43 | 34.46 | 27.51 | 23.89 | 24.18 | 27.98 | 33.71 | 41.65 |
| Democratic Republic of the Congo | 755.09 | 433.36 | 359.44 | 340.06 | 327.11 | 303.22 | 268.42 | 199.90 | 95.15 | 39.74 | 33.98 | 31.08 | 31.24 | 35.24 | 43.18 | 50.54 | 57.16 |
| Saint Vincent and the Grenadines | 442.08 | 382.55 | 357.68 | 371.28 | 371.89 | 336.16 | 265.95 | 184.26 | 87.14 | 35.97 | 31.00 | 28.13 | 27.47 | 30.46 | 37.09 | 44.16 | 51.41 |
| Serbia | 198.65 | 206.44 | 236.54 | 270.13 | 280.13 | 277.59 | 261.21 | 200.35 | 92.41 | 35.72 | 30.71 | 26.71 | 23.76 | 23.93 | 27.00 | 30.41 | 34.12 |
| Guinea-Bissau | 550.58 | 279.24 | 265.99 | 311.72 | 354.00 | 335.55 | 261.20 | 176.70 | 81.16 | 30.15 | 23.90 | 20.34 | 19.76 | 22.94 | 28.34 | 31.98 | 36.21 |
| Bulgaria | 514.61 | 417.97 | 334.15 | 353.37 | 346.13 | 309.61 | 257.12 | 184.75 | 86.13 | 34.80 | 30.74 | 29.13 | 30.24 | 31.69 | 32.48 | 33.11 | 38.42 |
| Gambia | 444.48 | 271.60 | 255.03 | 295.63 | 336.09 | 322.31 | 256.89 | 176.32 | 80.00 | 28.87 | 23.00 | 19.62 | 18.72 | 19.67 | 22.51 | 25.91 | 29.86 |
| Guyana | 455.94 | 348.64 | 300.59 | 322.25 | 355.72 | 332.44 | 256.72 | 177.09 | 90.54 | 45.02 | 40.72 | 36.80 | 33.78 | 33.68 | 34.41 | 35.73 | 39.15 |
| Singapore | 513.62 | 276.69 | 258.96 | 299.69 | 334.28 | 318.64 | 256.62 | 178.20 | 81.35 | 30.04 | 23.90 | 20.44 | 19.74 | 21.41 | 25.21 | 29.61 | 34.81 |
| Suriname | 513.54 | 422.90 | 346.52 | 370.40 | 363.97 | 321.52 | 256.04 | 179.24 | 84.32 | 32.70 | 25.66 | 20.61 | 17.73 | 18.37 | 22.77 | 30.23 | 41.26 |
| Cuba | 540.27 | 384.62 | 384.87 | 382.38 | 355.05 | 312.49 | 255.76 | 181.32 | 86.28 | 36.22 | 31.33 | 29.81 | 31.76 | 48.63 | 82.03 | 97.10 | 89.05 |
| Sweden | 624.72 | 421.98 | 389.00 | 366.14 | 336.03 | 298.31 | 253.60 | 184.81 | 89.26 | 37.52 | 29.67 | 23.82 | 19.98 | 21.03 | 27.10 | 33.45 | 39.80 |
| Saudi Arabia | 511.59 | 425.78 | 344.80 | 363.57 | 354.44 | 313.57 | 253.40 | 178.76 | 82.91 | 32.18 | 26.51 | 22.86 | 21.30 | 23.53 | 29.18 | 35.20 | 41.78 |
| Namibia | 289.05 | 306.63 | 311.59 | 323.95 | 306.35 | 283.02 | 252.28 | 187.49 | 87.31 | 35.04 | 30.86 | 28.40 | 27.53 | 29.06 | 33.11 | 37.62 | 41.47 |
| Antigua and Barbuda | 529.36 | 283.26 | 263.52 | 300.21 | 329.83 | 312.91 | 251.52 | 174.74 | 79.68 | 29.28 | 23.50 | 20.30 | 19.69 | 20.74 | 23.60 | 27.40 | 33.23 |
| China | 728.96 | 336.05 | 317.61 | 334.87 | 331.54 | 300.62 | 249.65 | 180.68 | 88.50 | 38.56 | 31.13 | 25.78 | 22.54 | 22.67 | 26.09 | 30.09 | 33.42 |
| Taiwan (Province of China) | 342.34 | 315.41 | 249.06 | 261.50 | 290.55 | 281.43 | 247.06 | 191.01 | 107.40 | 58.17 | 44.30 | 38.11 | 39.73 | 44.24 | 50.78 | 61.75 | 76.57 |
| Egypt | 698.09 | 275.42 | 274.31 | 315.51 | 339.44 | 314.78 | 246.80 | 167.19 | 76.41 | 28.19 | 22.85 | 20.51 | 21.27 | 24.50 | 28.72 | 32.21 | 36.93 |
| Argentina | 424.36 | 252.66 | 283.55 | 306.74 | 301.79 | 281.18 | 245.70 | 182.10 | 87.57 | 39.24 | 37.42 | 37.31 | 38.86 | 40.72 | 42.89 | 45.76 | 49.38 |
| Indonesia | 302.03 | 225.73 | 229.23 | 262.71 | 282.57 | 277.37 | 245.10 | 182.81 | 95.57 | 48.11 | 40.31 | 34.50 | 30.72 | 29.31 | 30.69 | 35.83 | 43.98 |
| Lithuania | 287.28 | 317.66 | 274.41 | 277.01 | 310.94 | 299.17 | 243.49 | 172.44 | 87.27 | 42.02 | 36.95 | 35.57 | 38.18 | 40.41 | 42.18 | 45.90 | 54.13 |
| Colombia | 290.11 | 398.66 | 304.10 | 294.79 | 314.25 | 293.31 | 243.19 | 177.06 | 91.58 | 46.76 | 42.77 | 40.15 | 38.83 | 39.26 | 40.66 | 42.05 | 44.12 |
| United Kingdom | 476.37 | 254.03 | 240.76 | 273.31 | 302.57 | 291.60 | 243.10 | 172.48 | 79.74 | 30.91 | 25.82 | 23.33 | 23.49 | 25.22 | 28.34 | 32.07 | 36.42 |
| San Marino | 660.69 | 300.03 | 303.12 | 325.19 | 322.41 | 292.20 | 242.86 | 175.33 | 84.90 | 37.06 | 32.11 | 29.34 | 28.73 | 30.41 | 34.26 | 38.71 | 43.56 |
| Cameroon | 129.28 | 161.98 | 173.66 | 216.61 | 261.40 | 268.41 | 242.83 | 182.66 | 88.74 | 40.02 | 34.76 | 31.98 | 31.27 | 31.78 | 34.19 | 37.04 | 39.47 |
| American Samoa | 450.36 | 330.27 | 278.40 | 297.92 | 332.30 | 312.03 | 241.79 | 167.39 | 85.89 | 43.83 | 41.69 | 39.36 | 36.80 | 35.73 | 36.41 | 38.94 | 43.01 |
| Slovenia | 586.37 | 256.92 | 249.08 | 289.05 | 324.85 | 306.59 | 239.60 | 162.64 | 75.83 | 28.59 | 21.15 | 16.26 | 13.91 | 13.68 | 15.57 | 17.83 | 19.88 |
| Slovakia | 619.58 | 284.37 | 258.25 | 294.83 | 320.75 | 300.60 | 239.32 | 165.03 | 76.94 | 29.05 | 21.45 | 16.51 | 14.34 | 15.09 | 18.82 | 23.52 | 28.49 |
| Côte d'Ivoire | 437.75 | 328.70 | 292.89 | 314.75 | 339.02 | 312.14 | 238.59 | 163.49 | 84.89 | 43.33 | 38.56 | 34.60 | 31.15 | 31.62 | 32.96 | 35.27 | 40.24 |
| Senegal | 337.97 | 400.24 | 322.81 | 308.45 | 302.91 | 279.06 | 238.26 | 174.91 | 86.87 | 38.66 | 31.03 | 25.07 | 20.98 | 20.05 | 22.06 | 26.14 | 32.81 |
| Lesotho | 292.90 | 333.53 | 283.24 | 284.15 | 293.62 | 276.78 | 237.41 | 175.11 | 88.09 | 40.49 | 32.82 | 29.27 | 29.53 | 30.17 | 31.33 | 35.14 | 41.98 |
| Mexico | 449.23 | 331.68 | 272.17 | 288.53 | 325.14 | 306.28 | 235.97 | 163.26 | 84.78 | 44.06 | 41.60 | 38.96 | 36.21 | 34.04 | 31.53 | 30.91 | 33.69 |
| Algeria | 239.77 | 256.76 | 232.55 | 248.68 | 289.30 | 283.86 | 232.74 | 164.71 | 80.86 | 36.65 | 31.96 | 31.07 | 34.00 | 37.85 | 41.42 | 37.56 | 37.47 |
| Finland | 274.90 | 209.47 | 200.97 | 224.49 | 257.87 | 259.30 | 232.65 | 177.33 | 90.42 | 42.73 | 35.51 | 32.72 | 35.30 | 38.72 | 41.97 | 48.05 | 56.64 |
| Kiribati | 482.55 | 464.58 | 313.88 | 325.24 | 328.70 | 292.20 | 232.63 | 165.51 | 81.92 | 35.67 | 26.93 | 20.93 | 17.62 | 18.54 | 23.55 | 28.90 | 34.86 |
| Tuvalu | 231.32 | 218.34 | 217.13 | 249.39 | 280.26 | 273.53 | 232.04 | 174.15 | 98.94 | 52.47 | 36.77 | 29.62 | 31.36 | 32.67 | 33.68 | 33.57 | 31.57 |
| Papua New Guinea | 487.72 | 384.11 | 287.94 | 280.18 | 306.52 | 285.09 | 231.91 | 158.26 | 86.99 | 50.20 | 43.31 | 39.82 | 37.44 | 35.22 | 37.65 | 36.50 | 38.01 |
| Eswatini | 463.57 | 309.90 | 297.13 | 293.46 | 282.67 | 261.78 | 231.01 | 173.19 | 85.18 | 37.28 | 29.83 | 24.48 | 21.26 | 21.49 | 24.94 | 28.58 | 32.38 |
| Djibouti | 292.71 | 392.66 | 304.80 | 289.64 | 297.76 | 275.39 | 230.90 | 167.96 | 84.07 | 41.72 | 41.20 | 40.02 | 38.16 | 38.01 | 39.45 | 41.05 | 43.48 |
| Albania | 353.12 | 358.99 | 308.24 | 294.83 | 283.23 | 262.77 | 230.85 | 171.77 | 83.20 | 38.36 | 37.35 | 34.77 | 30.77 | 32.15 | 38.88 | 47.73 | 59.08 |
| Afghanistan | 234.87 | 287.09 | 231.60 | 236.09 | 280.52 | 278.47 | 230.79 | 165.85 | 83.88 | 38.78 | 32.19 | 29.12 | 29.46 | 30.72 | 32.57 | 35.43 | 40.69 |
| Burkina Faso | 528.64 | 365.13 | 321.33 | 297.23 | 284.52 | 263.01 | 230.03 | 169.75 | 80.96 | 34.10 | 29.32 | 26.12 | 24.53 | 26.47 | 31.81 | 36.37 | 40.24 |
| Russian Federation | 256.48 | 265.79 | 235.11 | 251.05 | 263.55 | 255.02 | 228.74 | 171.47 | 82.09 | 35.97 | 33.16 | 32.53 | 34.10 | 35.42 | 36.49 | 38.02 | 40.02 |
| Ireland | 205.87 | 265.83 | 204.72 | 217.27 | 247.28 | 248.71 | 228.31 | 175.74 | 91.15 | 45.11 | 37.30 | 32.12 | 29.51 | 30.48 | 35.00 | 40.28 | 46.27 |
| Puerto Rico | 324.39 | 233.01 | 226.87 | 255.92 | 274.71 | 265.82 | 228.12 | 163.51 | 77.15 | 31.96 | 27.96 | 26.29 | 27.03 | 28.84 | 31.74 | 34.96 | 38.58 |
| Greenland | 259.89 | 321.66 | 271.25 | 265.51 | 267.40 | 254.34 | 228.09 | 171.34 | 82.73 | 36.73 | 33.62 | 33.59 | 36.62 | 38.18 | 38.52 | 41.31 | 47.62 |
| Republic of Korea | 211.93 | 250.23 | 227.58 | 240.87 | 269.22 | 263.82 | 227.02 | 166.17 | 81.64 | 37.66 | 34.30 | 33.72 | 35.95 | 37.62 | 38.72 | 40.91 | 44.12 |
| Malta | 261.48 | 296.45 | 249.90 | 261.07 | 286.73 | 271.59 | 226.79 | 163.87 | 80.69 | 37.06 | 32.93 | 30.31 | 29.29 | 30.78 | 34.09 | 37.99 | 40.67 |
| Poland | 456.70 | 344.53 | 282.41 | 290.77 | 314.86 | 290.46 | 223.13 | 154.94 | 81.63 | 42.86 | 39.24 | 35.76 | 32.03 | 30.05 | 29.11 | 29.89 | 31.34 |
| United States of America | 214.75 | 261.28 | 234.63 | 247.96 | 282.17 | 273.71 | 223.07 | 157.25 | 77.62 | 35.39 | 30.62 | 29.28 | 31.33 | 33.44 | 35.80 | 40.96 | 48.70 |
| Palestine | 461.59 | 336.80 | 281.27 | 293.51 | 315.92 | 290.86 | 223.06 | 154.05 | 81.25 | 42.93 | 38.43 | 34.62 | 32.48 | 31.50 | 31.93 | 34.95 | 39.52 |
| Nepal | 237.99 | 252.70 | 228.99 | 242.69 | 279.54 | 272.75 | 223.06 | 158.01 | 78.69 | 36.13 | 30.39 | 28.27 | 29.84 | 32.18 | 35.09 | 39.71 | 46.70 |
| Thailand | 318.15 | 313.37 | 257.94 | 264.84 | 280.14 | 262.80 | 222.62 | 161.10 | 77.49 | 33.10 | 27.72 | 24.34 | 22.89 | 24.66 | 29.95 | 36.24 | 43.73 |
| Belarus | 707.30 | 273.71 | 256.64 | 283.98 | 302.43 | 279.94 | 222.09 | 153.06 | 72.59 | 28.63 | 21.12 | 16.25 | 14.12 | 14.94 | 18.81 | 23.00 | 28.38 |
| Bosnia and Herzegovina | 333.49 | 247.26 | 228.57 | 240.76 | 252.32 | 243.94 | 221.71 | 167.85 | 81.46 | 36.51 | 33.20 | 32.13 | 33.26 | 34.58 | 36.11 | 38.18 | 40.80 |
| Marshall Islands | 317.85 | 311.75 | 257.19 | 267.45 | 289.72 | 270.01 | 221.03 | 157.61 | 78.33 | 36.44 | 31.35 | 27.82 | 25.89 | 27.23 | 31.87 | 37.49 | 44.63 |
| El Salvador | 405.66 | 252.23 | 229.95 | 258.93 | 278.68 | 264.92 | 220.42 | 157.12 | 73.50 | 28.99 | 23.61 | 20.65 | 20.10 | 20.79 | 22.62 | 24.90 | 27.67 |
| Ghana | 174.10 | 199.08 | 197.29 | 236.27 | 269.86 | 261.28 | 219.49 | 160.26 | 79.52 | 37.08 | 33.08 | 28.51 | 23.67 | 23.68 | 28.16 | 30.16 | 30.81 |
| Benin | 588.03 | 250.59 | 240.14 | 269.68 | 290.29 | 271.79 | 218.24 | 151.70 | 71.34 | 27.47 | 20.33 | 15.70 | 13.60 | 13.80 | 16.46 | 19.59 | 23.32 |
| Equatorial Guinea | 295.91 | 265.69 | 230.65 | 245.83 | 258.28 | 247.40 | 218.07 | 162.43 | 80.05 | 36.71 | 32.48 | 30.62 | 31.24 | 33.46 | 37.16 | 41.35 | 45.91 |
| Lebanon | 255.98 | 263.20 | 259.07 | 269.12 | 279.89 | 262.91 | 217.53 | 155.19 | 76.31 | 35.20 | 32.15 | 33.54 | 38.92 | 43.85 | 46.96 | 44.64 | 44.02 |
| Sri Lanka | 459.10 | 313.23 | 261.89 | 267.94 | 286.35 | 263.89 | 217.12 | 155.50 | 76.57 | 34.08 | 27.99 | 24.30 | 22.86 | 25.16 | 31.01 | 37.35 | 44.89 |
| Nicaragua | 279.89 | 210.11 | 200.88 | 220.69 | 245.09 | 241.35 | 216.77 | 166.26 | 87.25 | 43.13 | 34.90 | 31.55 | 33.39 | 36.28 | 39.40 | 45.09 | 52.91 |
| Saint Lucia | 486.63 | 401.37 | 295.36 | 272.14 | 272.98 | 253.50 | 216.69 | 160.60 | 82.04 | 38.21 | 29.21 | 22.85 | 19.15 | 19.85 | 24.84 | 30.49 | 36.84 |
| Croatia | 330.89 | 220.87 | 229.68 | 259.01 | 277.60 | 263.43 | 216.14 | 152.45 | 74.84 | 32.65 | 25.81 | 21.32 | 19.19 | 19.85 | 23.21 | 27.01 | 31.26 |
| Montenegro | 269.22 | 184.69 | 190.42 | 220.18 | 248.33 | 247.07 | 215.90 | 158.31 | 79.76 | 37.68 | 31.85 | 28.62 | 27.97 | 28.88 | 31.35 | 34.25 | 37.27 |
| Brazil | 203.31 | 209.14 | 193.28 | 211.96 | 237.12 | 236.91 | 215.53 | 165.52 | 84.21 | 39.96 | 33.80 | 31.75 | 34.05 | 37.30 | 40.26 | 45.99 | 54.07 |
| Iran (Islamic Republic of) | 422.50 | 375.20 | 319.95 | 290.51 | 274.73 | 249.84 | 215.19 | 158.69 | 77.62 | 33.91 | 27.63 | 22.93 | 19.77 | 20.71 | 26.03 | 32.02 | 38.65 |
| Luxembourg | 236.19 | 214.29 | 190.28 | 209.58 | 236.10 | 235.13 | 215.18 | 166.78 | 85.25 | 40.06 | 33.03 | 30.55 | 34.04 | 38.47 | 41.38 | 46.67 | 54.45 |
| Brunei Darussalam | 505.33 | 329.37 | 266.85 | 254.32 | 271.25 | 260.87 | 214.88 | 152.51 | 78.74 | 37.86 | 29.82 | 25.05 | 23.62 | 25.82 | 31.62 | 37.74 | 43.38 |
| Bahamas | 205.61 | 242.37 | 214.70 | 227.12 | 260.30 | 255.87 | 213.54 | 152.51 | 74.86 | 33.38 | 28.01 | 25.73 | 26.86 | 27.85 | 28.85 | 31.81 | 36.98 |
| Kenya | 663.87 | 242.54 | 239.54 | 264.14 | 276.78 | 257.74 | 211.92 | 150.61 | 72.41 | 29.88 | 23.24 | 19.20 | 17.66 | 17.83 | 20.23 | 23.48 | 27.69 |
| Niger | 452.52 | 330.61 | 263.98 | 269.76 | 291.39 | 270.24 | 211.03 | 148.01 | 77.72 | 40.07 | 35.92 | 33.42 | 32.56 | 30.79 | 29.25 | 29.97 | 32.76 |
| Bolivia (Plurinational State of) | 179.57 | 225.01 | 195.39 | 214.96 | 232.76 | 231.44 | 211.00 | 158.85 | 79.25 | 36.73 | 31.19 | 27.22 | 24.84 | 25.75 | 29.86 | 34.08 | 38.23 |
| Ethiopia | 185.44 | 207.84 | 199.54 | 233.87 | 260.28 | 248.60 | 209.92 | 153.88 | 75.35 | 34.16 | 30.23 | 26.36 | 23.45 | 25.02 | 30.58 | 32.39 | 31.52 |
| Ukraine | 451.75 | 326.39 | 267.88 | 276.81 | 296.23 | 272.26 | 209.67 | 145.65 | 77.63 | 40.75 | 36.14 | 32.19 | 29.12 | 27.24 | 27.57 | 27.36 | 30.54 |
| Cyprus | 510.50 | 270.43 | 240.95 | 266.61 | 281.37 | 260.71 | 209.05 | 146.00 | 69.54 | 27.69 | 20.42 | 15.72 | 13.63 | 13.43 | 15.17 | 17.41 | 20.20 |
| Spain | 294.82 | 276.11 | 314.39 | 321.03 | 285.22 | 248.98 | 208.90 | 150.42 | 71.66 | 29.87 | 25.31 | 22.31 | 20.83 | 26.02 | 38.80 | 45.81 | 44.50 |
| Costa Rica | 174.44 | 193.88 | 185.17 | 219.90 | 251.84 | 245.67 | 208.85 | 153.32 | 75.63 | 34.57 | 30.08 | 25.62 | 21.45 | 22.56 | 28.83 | 31.20 | 30.04 |
| Morocco | 453.19 | 330.98 | 261.33 | 264.96 | 285.57 | 265.21 | 208.72 | 147.68 | 78.52 | 42.32 | 38.48 | 34.57 | 31.80 | 31.29 | 31.07 | 32.51 | 36.72 |
| Comoros | 559.10 | 230.59 | 229.10 | 250.52 | 263.41 | 248.09 | 207.47 | 149.70 | 73.19 | 31.59 | 24.88 | 20.63 | 18.82 | 19.01 | 21.25 | 23.91 | 26.94 |
| Bangladesh | 207.87 | 241.25 | 223.57 | 241.00 | 268.38 | 256.12 | 207.16 | 144.96 | 71.32 | 31.69 | 26.06 | 22.91 | 22.11 | 22.04 | 22.91 | 24.81 | 28.29 |
| Iceland | 427.95 | 253.34 | 223.77 | 249.27 | 270.35 | 255.26 | 206.86 | 144.91 | 68.00 | 26.09 | 19.08 | 14.49 | 12.38 | 12.66 | 15.31 | 18.28 | 20.89 |
| Nauru | 213.33 | 236.87 | 217.00 | 241.42 | 253.28 | 242.30 | 206.47 | 148.15 | 71.83 | 30.88 | 25.27 | 21.68 | 20.17 | 21.15 | 24.70 | 28.61 | 33.13 |
| Myanmar | 379.28 | 324.77 | 274.99 | 263.72 | 264.28 | 243.83 | 205.80 | 152.05 | 79.68 | 38.70 | 29.52 | 26.24 | 28.23 | 29.67 | 30.34 | 31.20 | 33.36 |
| Solomon Islands | 704.45 | 294.91 | 252.91 | 257.57 | 278.75 | 259.36 | 205.80 | 147.31 | 78.02 | 41.93 | 39.70 | 37.63 | 36.22 | 35.75 | 35.03 | 36.24 | 39.73 |
| United Arab Emirates | 496.02 | 304.20 | 262.16 | 267.31 | 282.91 | 260.57 | 205.79 | 145.64 | 77.22 | 41.66 | 39.22 | 37.06 | 35.31 | 34.89 | 35.42 | 37.93 | 43.36 |
| Uzbekistan | 350.79 | 418.50 | 314.51 | 280.67 | 272.52 | 246.14 | 204.93 | 148.92 | 75.70 | 35.06 | 27.67 | 23.35 | 22.23 | 23.84 | 28.42 | 37.33 | 52.15 |
| Palau | 189.13 | 218.04 | 204.32 | 222.21 | 252.62 | 246.79 | 204.88 | 146.16 | 71.87 | 32.07 | 26.89 | 24.72 | 25.79 | 27.10 | 28.84 | 32.63 | 39.14 |
| Armenia | 309.58 | 329.14 | 282.13 | 271.66 | 263.41 | 239.90 | 204.12 | 150.22 | 76.36 | 35.59 | 28.25 | 24.32 | 23.77 | 25.76 | 29.80 | 35.01 | 43.27 |
| Panama | 279.75 | 327.87 | 300.39 | 294.05 | 271.90 | 242.05 | 203.32 | 147.24 | 71.87 | 31.00 | 24.76 | 19.87 | 16.25 | 16.47 | 20.31 | 23.59 | 26.47 |
| Azerbaijan | 287.66 | 319.03 | 267.94 | 262.60 | 259.10 | 238.01 | 203.05 | 149.50 | 75.69 | 35.95 | 30.55 | 28.20 | 28.82 | 30.68 | 33.79 | 37.84 | 42.47 |
| Saint Kitts and Nevis | 285.86 | 316.78 | 262.12 | 257.59 | 259.92 | 240.15 | 201.72 | 147.17 | 74.89 | 34.87 | 27.36 | 23.52 | 23.41 | 24.68 | 27.51 | 33.70 | 43.87 |
| Estonia | 227.80 | 206.74 | 203.04 | 224.97 | 237.28 | 230.01 | 201.65 | 148.19 | 71.55 | 31.59 | 28.27 | 27.01 | 27.86 | 29.07 | 30.54 | 32.50 | 35.05 |
| Niue | 350.73 | 370.21 | 305.82 | 302.43 | 305.35 | 268.09 | 201.22 | 136.02 | 71.44 | 35.23 | 27.37 | 21.66 | 18.38 | 25.74 | 45.26 | 66.01 | 89.65 |
| Botswana | 235.70 | 250.23 | 255.95 | 267.83 | 257.28 | 235.53 | 200.75 | 146.04 | 69.02 | 28.10 | 23.52 | 21.37 | 21.77 | 22.38 | 22.95 | 24.61 | 27.37 |
| Sao Tome and Principe | 205.07 | 232.59 | 215.38 | 230.48 | 252.87 | 242.25 | 200.68 | 143.78 | 72.10 | 33.89 | 29.77 | 27.90 | 28.35 | 28.90 | 30.12 | 33.97 | 38.57 |
| Vanuatu | 454.22 | 325.54 | 256.06 | 259.41 | 278.75 | 257.07 | 199.35 | 139.77 | 74.55 | 39.49 | 35.28 | 31.22 | 27.94 | 26.61 | 26.75 | 28.39 | 31.98 |
| Gabon | 437.82 | 306.20 | 244.76 | 250.73 | 267.75 | 249.04 | 199.35 | 142.91 | 76.10 | 39.88 | 34.63 | 30.44 | 27.55 | 26.53 | 26.95 | 29.84 | 33.73 |
| India | 482.79 | 251.20 | 220.84 | 245.55 | 263.78 | 246.70 | 199.32 | 139.34 | 65.22 | 24.87 | 18.29 | 14.15 | 12.46 | 13.04 | 15.85 | 19.13 | 22.99 |
| Cabo Verde | 129.06 | 155.09 | 154.60 | 182.28 | 209.18 | 213.18 | 198.96 | 155.01 | 81.05 | 41.67 | 36.49 | 33.67 | 32.94 | 33.61 | 36.09 | 37.10 | 37.21 |
| Peru | 201.57 | 234.46 | 209.72 | 222.12 | 244.17 | 235.47 | 197.96 | 142.69 | 70.73 | 31.60 | 25.27 | 21.27 | 19.83 | 19.67 | 20.96 | 23.54 | 27.27 |
| Norway | 193.33 | 201.69 | 209.14 | 232.61 | 244.22 | 232.86 | 196.68 | 141.26 | 69.13 | 29.91 | 23.58 | 19.35 | 17.25 | 17.79 | 21.22 | 25.57 | 31.59 |
| Uruguay | 172.33 | 225.95 | 216.62 | 225.70 | 246.51 | 236.54 | 196.23 | 140.14 | 68.93 | 31.51 | 28.05 | 27.22 | 28.96 | 28.96 | 27.21 | 26.81 | 30.34 |
| New Zealand | 329.92 | 290.26 | 235.73 | 242.65 | 254.92 | 235.44 | 196.11 | 141.16 | 68.55 | 29.95 | 25.38 | 22.36 | 20.94 | 22.30 | 26.78 | 33.08 | 42.01 |
| Qatar | 444.46 | 311.27 | 248.51 | 254.25 | 271.78 | 250.43 | 194.83 | 136.31 | 72.38 | 37.65 | 31.92 | 26.30 | 20.97 | 18.63 | 19.38 | 21.45 | 25.08 |
| Tonga | 198.35 | 254.96 | 215.90 | 212.54 | 232.94 | 227.04 | 194.48 | 142.50 | 72.49 | 34.94 | 29.75 | 35.04 | 50.41 | 55.67 | 50.35 | 49.68 | 57.93 |
| Nigeria | 178.98 | 221.31 | 188.32 | 204.31 | 218.67 | 214.51 | 193.87 | 145.70 | 73.09 | 33.53 | 27.07 | 22.18 | 18.87 | 19.36 | 23.68 | 28.25 | 33.11 |
| Jamaica | 218.42 | 233.45 | 211.69 | 220.93 | 246.47 | 237.29 | 193.39 | 137.10 | 69.52 | 32.57 | 26.60 | 24.12 | 25.11 | 26.27 | 27.85 | 31.83 | 38.68 |
| Eritrea | 195.60 | 245.88 | 214.42 | 220.58 | 246.32 | 236.93 | 192.75 | 137.22 | 71.44 | 34.28 | 25.94 | 21.61 | 21.16 | 21.13 | 19.79 | 18.43 | 21.31 |
| Philippines | 189.09 | 236.25 | 184.59 | 194.14 | 216.40 | 213.52 | 190.96 | 146.85 | 78.66 | 40.39 | 32.39 | 26.65 | 23.29 | 24.59 | 30.53 | 37.00 | 44.01 |
| Cook Islands | 399.07 | 303.68 | 241.56 | 244.60 | 261.86 | 242.20 | 190.71 | 135.41 | 72.31 | 38.50 | 34.16 | 29.66 | 25.38 | 23.60 | 24.78 | 27.53 | 32.46 |
| Liberia | 291.22 | 267.09 | 225.85 | 238.80 | 251.86 | 232.32 | 190.48 | 135.45 | 66.10 | 28.78 | 23.59 | 20.04 | 17.85 | 18.64 | 22.65 | 27.97 | 36.08 |
| Tunisia | 284.41 | 314.52 | 260.44 | 252.64 | 247.81 | 225.48 | 189.52 | 138.59 | 71.06 | 33.28 | 25.40 | 20.54 | 18.57 | 18.55 | 19.90 | 22.36 | 26.34 |
| Kuwait | 205.38 | 242.37 | 218.19 | 225.45 | 243.22 | 230.63 | 188.94 | 134.10 | 67.18 | 30.55 | 24.47 | 20.82 | 19.64 | 21.03 | 25.02 | 27.97 | 30.06 |
| Angola | 172.58 | 190.48 | 180.67 | 211.68 | 237.54 | 226.70 | 188.55 | 136.77 | 67.48 | 31.13 | 27.37 | 23.10 | 18.77 | 19.60 | 25.04 | 26.97 | 26.95 |
| Turkey | 392.91 | 398.77 | 274.74 | 242.86 | 250.37 | 228.29 | 188.03 | 135.66 | 69.81 | 33.73 | 27.49 | 22.74 | 19.56 | 19.66 | 23.16 | 27.67 | 32.92 |
| Denmark | 125.76 | 148.41 | 149.41 | 176.82 | 201.98 | 204.52 | 187.56 | 144.06 | 74.02 | 37.24 | 33.32 | 31.17 | 30.62 | 31.36 | 33.55 | 34.74 | 35.37 |
| Portugal | 255.72 | 231.99 | 215.44 | 222.64 | 240.83 | 228.98 | 187.52 | 132.52 | 66.02 | 29.76 | 23.68 | 20.32 | 19.86 | 19.73 | 19.70 | 21.56 | 27.11 |
| Romania | 258.25 | 286.90 | 239.53 | 233.12 | 236.54 | 223.23 | 186.12 | 132.14 | 65.77 | 29.61 | 23.69 | 19.91 | 18.31 | 20.00 | 25.02 | 30.60 | 36.45 |
| Turkmenistan | 312.04 | 296.49 | 237.29 | 239.27 | 247.91 | 226.79 | 185.88 | 132.21 | 65.12 | 29.00 | 23.71 | 19.77 | 17.16 | 17.25 | 20.81 | 24.83 | 30.09 |
| Latvia | 468.62 | 312.44 | 248.91 | 241.57 | 252.83 | 232.07 | 183.33 | 129.67 | 68.48 | 36.12 | 32.37 | 30.34 | 29.98 | 29.04 | 27.49 | 29.47 | 35.88 |
| Canada | 257.13 | 300.66 | 226.85 | 227.01 | 236.08 | 217.68 | 181.13 | 130.23 | 64.10 | 28.70 | 23.74 | 20.01 | 17.65 | 18.32 | 22.16 | 27.45 | 34.62 |
| Republic of Moldova | 126.50 | 150.61 | 150.07 | 175.24 | 197.36 | 196.99 | 179.96 | 138.71 | 71.39 | 35.84 | 32.10 | 30.35 | 30.57 | 32.59 | 36.47 | 38.43 | 38.78 |
| Switzerland | 442.54 | 292.56 | 233.80 | 231.39 | 246.27 | 227.44 | 179.95 | 127.89 | 68.11 | 35.57 | 30.23 | 25.36 | 21.15 | 19.49 | 20.36 | 22.35 | 26.32 |
| South Africa | 131.90 | 159.18 | 154.05 | 175.76 | 195.21 | 194.35 | 178.60 | 137.51 | 70.35 | 34.44 | 29.32 | 26.24 | 24.91 | 26.85 | 32.16 | 33.10 | 28.04 |
| Bermuda | 151.89 | 143.00 | 150.52 | 175.95 | 197.66 | 195.73 | 177.48 | 136.30 | 69.92 | 34.57 | 30.50 | 28.20 | 27.72 | 28.65 | 31.12 | 33.00 | 34.55 |
| Greece | 125.66 | 148.82 | 147.80 | 172.05 | 192.85 | 192.75 | 176.88 | 136.89 | 70.99 | 35.92 | 31.74 | 29.72 | 29.27 | 30.02 | 32.33 | 33.70 | 34.59 |
| South Sudan | 126.63 | 149.12 | 148.66 | 173.71 | 194.88 | 194.12 | 176.37 | 135.36 | 69.94 | 35.28 | 31.45 | 29.53 | 29.32 | 30.78 | 33.97 | 35.68 | 36.52 |
| Guam | 145.16 | 150.42 | 148.49 | 169.84 | 189.16 | 189.40 | 175.95 | 136.21 | 70.86 | 35.72 | 30.14 | 27.11 | 26.37 | 26.83 | 28.79 | 30.31 | 31.54 |
| Uganda | 102.74 | 141.65 | 142.48 | 166.45 | 187.69 | 190.12 | 174.74 | 134.44 | 68.85 | 33.94 | 29.73 | 27.05 | 25.76 | 27.07 | 30.22 | 31.96 | 33.35 |
| Honduras | 121.28 | 141.80 | 145.41 | 171.38 | 191.04 | 190.19 | 173.61 | 134.04 | 69.25 | 34.82 | 31.25 | 29.14 | 28.86 | 29.96 | 32.49 | 34.10 | 35.20 |
| Pakistan | 109.40 | 138.78 | 133.58 | 160.29 | 189.68 | 192.50 | 173.26 | 133.34 | 72.05 | 39.04 | 33.85 | 31.23 | 31.02 | 32.75 | 36.45 | 39.96 | 40.96 |
| Sudan | 126.32 | 149.86 | 148.00 | 171.09 | 190.50 | 188.67 | 171.44 | 131.48 | 67.76 | 34.11 | 30.37 | 28.33 | 28.17 | 29.31 | 32.49 | 34.92 | 36.01 |
| North Macedonia | 172.75 | 154.21 | 148.37 | 171.33 | 191.21 | 189.61 | 171.24 | 131.57 | 67.59 | 34.13 | 29.88 | 27.43 | 26.81 | 28.31 | 32.53 | 33.52 | 34.73 |
| Hungary | 122.81 | 143.53 | 144.08 | 170.17 | 192.70 | 191.42 | 171.18 | 130.13 | 67.00 | 33.53 | 29.84 | 27.58 | 26.79 | 27.72 | 30.61 | 32.88 | 34.39 |
| Dominica | 172.59 | 185.02 | 166.98 | 192.75 | 214.55 | 203.65 | 169.56 | 123.74 | 62.02 | 29.15 | 24.98 | 20.62 | 16.86 | 17.25 | 21.37 | 23.09 | 23.23 |
| Chile | 123.10 | 144.35 | 145.15 | 170.23 | 190.83 | 189.15 | 169.48 | 129.25 | 66.59 | 33.21 | 29.51 | 27.55 | 27.17 | 28.08 | 30.20 | 31.35 | 32.46 |
| Rwanda | 293.18 | 302.82 | 209.09 | 207.35 | 220.64 | 203.10 | 169.00 | 124.56 | 64.69 | 31.49 | 24.99 | 20.79 | 18.97 | 21.08 | 26.72 | 32.33 | 32.63 |
| Burundi | 112.27 | 143.81 | 144.07 | 175.48 | 207.40 | 203.02 | 166.63 | 119.73 | 61.83 | 31.73 | 29.31 | 27.62 | 26.30 | 27.15 | 30.05 | 30.04 | 27.44 |
| Mauritania | 122.56 | 142.45 | 142.38 | 166.31 | 185.25 | 183.59 | 165.93 | 126.40 | 63.79 | 30.67 | 27.10 | 25.21 | 24.97 | 26.47 | 29.90 | 32.77 | 34.30 |
| Israel | 260.25 | 270.06 | 198.20 | 202.30 | 219.18 | 202.54 | 163.19 | 117.00 | 60.53 | 28.75 | 21.76 | 16.72 | 13.81 | 13.67 | 16.54 | 20.70 | 26.33 |
| Chad | 170.79 | 179.85 | 159.22 | 182.70 | 204.66 | 195.03 | 162.40 | 118.28 | 59.30 | 27.53 | 22.88 | 18.56 | 14.88 | 14.91 | 18.37 | 20.15 | 20.20 |
| Netherlands | 194.72 | 156.18 | 161.79 | 197.85 | 236.77 | 223.09 | 159.36 | 106.16 | 59.75 | 35.98 | 33.76 | 30.56 | 26.56 | 24.31 | 24.04 | 21.89 | 18.79 |
| Zambia | 213.27 | 164.25 | 161.21 | 189.59 | 222.32 | 209.91 | 157.46 | 108.61 | 60.28 | 35.36 | 33.87 | 30.84 | 27.20 | 26.65 | 29.16 | 27.18 | 22.22 |
| Oman | 123.93 | 145.02 | 142.70 | 165.37 | 183.87 | 179.00 | 157.04 | 118.44 | 61.08 | 30.27 | 26.06 | 23.51 | 22.94 | 23.98 | 26.96 | 29.55 | 30.69 |
| Micronesia (Federated States of) | 198.88 | 155.82 | 155.79 | 189.91 | 229.73 | 217.10 | 155.45 | 102.94 | 54.64 | 28.38 | 24.43 | 20.84 | 18.41 | 19.08 | 22.28 | 23.09 | 22.41 |
| Tokelau | 161.29 | 149.02 | 151.10 | 181.97 | 216.64 | 206.79 | 154.10 | 105.79 | 57.73 | 32.80 | 31.19 | 29.24 | 26.55 | 26.42 | 28.84 | 26.76 | 21.50 |
| Ecuador | 204.58 | 157.99 | 155.05 | 183.03 | 213.09 | 203.49 | 153.55 | 105.75 | 57.15 | 31.87 | 30.24 | 27.13 | 24.32 | 24.88 | 28.84 | 28.32 | 24.90 |
| Congo | 195.88 | 152.09 | 151.38 | 179.74 | 209.71 | 198.67 | 150.27 | 103.42 | 54.54 | 28.40 | 25.28 | 22.47 | 20.00 | 21.84 | 27.53 | 28.96 | 26.43 |
| Venezuela (Bolivarian Republic of) | 240.83 | 146.93 | 148.39 | 171.51 | 196.15 | 186.29 | 145.09 | 102.40 | 54.51 | 28.76 | 25.23 | 21.91 | 19.38 | 20.37 | 23.91 | 23.06 | 21.42 |
| Maldives | 194.48 | 149.73 | 145.58 | 170.83 | 198.28 | 188.51 | 144.88 | 101.08 | 53.77 | 28.60 | 25.28 | 22.10 | 19.11 | 20.30 | 25.69 | 27.22 | 24.95 |
| Sierra Leone | 281.28 | 140.50 | 146.62 | 170.90 | 195.96 | 184.63 | 144.00 | 101.27 | 52.85 | 27.24 | 24.37 | 21.49 | 19.12 | 20.21 | 24.54 | 24.93 | 23.41 |
| Tajikistan | 198.35 | 149.71 | 145.32 | 168.30 | 192.99 | 184.19 | 143.71 | 100.76 | 53.31 | 28.20 | 25.28 | 22.14 | 20.09 | 21.94 | 28.26 | 29.98 | 26.84 |
| Mali | 195.61 | 150.54 | 144.34 | 167.40 | 192.51 | 183.52 | 141.08 | 98.22 | 52.32 | 28.75 | 27.52 | 25.81 | 23.86 | 23.49 | 24.33 | 23.07 | 21.87 |
| Cambodia | 193.10 | 146.81 | 140.13 | 160.92 | 182.75 | 173.18 | 135.27 | 95.64 | 50.79 | 27.11 | 24.20 | 21.75 | 19.35 | 20.34 | 24.52 | 25.27 | 22.97 |
| France | 190.24 | 168.63 | 134.97 | 156.97 | 185.22 | 174.34 | 130.22 | 89.74 | 49.80 | 28.86 | 27.00 | 26.93 | 27.11 | 27.08 | 26.98 | 23.96 | 18.18 |
| Lao People's Democratic Republic | 150.91 | 148.54 | 126.61 | 148.29 | 173.85 | 165.47 | 126.29 | 88.22 | 48.74 | 27.73 | 25.07 | 23.11 | 21.22 | 19.77 | 19.62 | 17.88 | 15.96 |
| Syrian Arab Republic | 197.15 | 146.67 | 136.57 | 155.00 | 173.65 | 162.83 | 126.12 | 88.75 | 46.19 | 23.16 | 19.61 | 16.56 | 13.82 | 14.49 | 18.33 | 19.88 | 18.92 |
