# Supplementary material for: Global burden of drug use disorders by region and country, 1990–2021
Source: Front Public Health. 2024 Oct 29;12:1470809. doi: 10.3389/fpubh.2024.1470809 (PMC11554507; doi:10.3389/fpubh.2024.1470809)
Supplement: Supplementary file 3 [file Table_2.docx]

**Table 1 Age-standardized DALY rate and ASIR and corresponding EAPC for global DUDs in 1990 and 2021.**

| location | ASIR | | | Age-standardized DALY rate (per 100000) | | |
| --- | --- | --- | --- | --- | --- | --- |
|  | 1990 No.(95%UI) | 2021 No.(95%UI) | 1990-2021 EAPC No.(95%CI) | 1990 No.(95%UI) | 2021 No.(95%UI) | 1990-2021 EAPC No.(95%CI) |
| **Global** |  |  |  |  |  |  |
| male | 222.54(190.47,256.77) | 183.99(159.71,211.51) | -0.26(-5.54,5.31) | 851.58(686.58,1023.05) | 598.14(479.64,722.15) | -1.21(-7.61,5.64) |
| female | 189.27(158.66,221.96) | 154.12(130.49,179.18) | -0.26(-5.37,5.11) | 642.58(519.51,765.26) | 392.51(312.52,473.79) | -1.70(-7.67,4.65) |
| both | 206.18(174.39,238.74) | 169.39(145.14,195.01) | -0.26(-5.46,5.22) | 742.17(597.93,879.88) | 489.81(391.39,593.21) | -1.44(-7.63,5.18) |
| **SDI** |  |  |  |  |  |  |
| High-middle SDI | 213.21(181.57,246.44) | 189.65(161.53,218.09) | -0.58(-5.90,5.04) | 856.77(690.30,1031.82) | 482.12(379.01,594.29) | -2.11(-8.26,4.45) |
| High SDI | 284.65(242.42,330.56) | 350.90(307.36,400.20) | 0.65(-5.34,7.02) | 548.06(430.57,664.63) | 220.40(173.92,266.10) | -3.19(-8.51,2.44) |
| Low-middle SDI | 124.75(105.84,145.45) | 130.55(110.96,151.41) | 0.19(-4.80,5.44) | 794.83(643.62,954.95) | 709.62(559.48,858.77) | -0.32(-6.95,6.79) |
| Low SDI | 107.37(88.96,125.35) | 110.82(92.59,128.79) | 0.15(-4.67,5.21) | 719.83(575.38,878.66) | 597.97(468.77,731.27) | -0.63(-7.08,6.26) |
| Middle SDI | 178.51(152.88,204.74) | 155.19(131.25,179.27) | -0.59(-5.71,4.81) | 750.17(611.72,916.52) | 555.50(440.55,679.88) | -0.86(-7.22,5.93) |
| regions |  |  |  |  |  |  |
| Andean Latin America | 195.61(166.30,225.46) | 147.25(123.64,171.11) | -0.20(-5.27,5.13) | 303.16(238.57,372.33) | 177.54(130.63,230.85) | -1.95(-7.15,3.54) |
| Australasia | 529.52(453.67,616.27) | 425.48(369.38,483.04) | -0.25(-6.36,6.26) | 508.43(383.46,631.96) | 139.51(107.13,171.93) | -4.41(-9.22,0.66) |
| Caribbean | 199.55(166.32,238.29) | 180.09(147.21,220.40) | -0.18(-5.44,5.36) | 639.20(505.32,776.75) | 424.57(332.08,524.68) | -1.32(-7.39,5.15) |
| Central Asia | 173.93(144.74,204.34) | 169.72(143.57,197.09) | 0.18(-5.04,5.68) | 1574.74(1267.28,1868.55) | 1243.96(979.87,1537.34) | -1.26(-8.38,6.42) |
| Central Europe | 197.80(164.95,233.27) | 184.24(155.27,214.63) | 0.16(-5.14,5.75) | 934.23(736.11,1129.34) | 444.98(348.90,543.68) | -2.81(-8.84,3.61) |
| Central Latin America | 162.99(137.28,191.04) | 144.04(121.38,167.38) | 0.04(-5.01,5.35) | 552.50(437.50,663.36) | 417.92(320.09,518.20) | -1.10(-7.16,5.35) |
| Central Sub-Saharan Africa | 128.28(106.92,150.81) | 110.05(91.77,129.82) | -0.10(-4.88,4.91) | 957.08(730.98,1235.06) | 807.46(606.17,1044.96) | -0.76(-7.48,6.45) |
| East Asia | 229.24(194.35,266.18) | 173.93(146.09,204.63) | -0.61(-5.82,4.88) | 631.09(487.49,794.25) | 416.58(317.97,550.67) | -0.89(-6.97,5.59) |
| Eastern Europe | 302.11(257.48,349.99) | 275.72(238.80,312.90) | -0.11(-5.79,5.92) | 1341.94(1052.31,1614.53) | 968.69(755.39,1183.22) | -1.65(-8.51,5.72) |
| Eastern Sub-Saharan Africa | 114.08(95.47,135.50) | 101.09(83.77,119.60) | 0.02(-4.68,4.95) | 481.45(374.63,608.71) | 379.81(288.86,492.14) | -0.93(-6.91,5.44) |
| High-income Asia Pacific | 222.75(180.91,269.46) | 204.38(168.19,247.27) | 0.04(-5.36,5.75) | 366.11(281.03,456.93) | 128.42(100.73,159.17) | -3.44(-8.23,1.60) |
| High-income North America | 436.37(373.43,512.11) | 520.07(454.13,592.82) | 1.02(-5.34,7.81) | 578.69(438.12,709.61) | 291.42(228.20,354.80) | -2.56(-8.18,3.41) |
| North Africa and Middle East | 145.74(119.98,173.60) | 143.52(120.87,169.07) | 0.33(-4.74,5.66) | 1149.62(884.03,1419.31) | 770.51(569.14,975.54) | -1.33(-7.98,5.79) |
| Oceania | 185.47(152.23,230.70) | 173.25(141.60,212.00) | -0.04(-5.27,5.48) | 998.63(782.43,1285.58) | 891.76(686.65,1156.78) | -0.35(-7.20,7.02) |
| South Asia | 139.85(116.75,164.68) | 131.41(109.78,153.28) | 0.23(-4.73,5.46) | 736.91(585.48,911.41) | 678.71(520.99,835.91) | -0.22(-6.82,6.85) |
| Southeast Asia | 155.47(130.90,183.14) | 141.48(116.93,166.01) | -0.01(-5.04,5.28) | 943.06(774.81,1144.82) | 822.39(651.11,1002.89) | -0.43(-7.19,6.82) |
| Southern Latin America | 211.62(175.58,249.36) | 196.13(167.54,227.34) | 0.12(-5.24,5.78) | 432.65(333.62,532.54) | 165.45(126.13,203.86) | -2.84(-7.93,2.53) |
| Southern Sub-Saharan Africa | 260.10(221.76,303.29) | 161.51(137.31,186.47) | -1.27(-6.35,4.08) | 564.09(453.13,678.12) | 620.42(504.67,742.66) | 0.34(-6.22,7.36) |
| Tropical Latin America | 203.95(173.73,242.27) | 180.40(153.43,207.80) | 0.15(-5.15,5.73) | 700.52(576.95,824.96) | 307.55(250.01,364.38) | -2.66(-8.34,3.38) |
| Western Europe | 321.12(272.30,373.02) | 302.00(262.87,348.16) | 0.12(-5.67,6.26) | 491.11(385.72,598.00) | 156.51(123.02,191.55) | -3.90(-8.87,1.33) |
| Western Sub-Saharan Africa | 106.83(89.61,126.14) | 94.68(79.60,111.36) | 0.22(-4.42,5.09) | 769.65(623.31,940.52) | 649.65(523.69,798.64) | -0.57(-7.09,6.42) |
